# Supplementary material for: Exploration of the social determinants of diarrhoea, rotavirus vaccine uptake, and vaccine ‘fatigue’ in Ethiopia, Kenya, and Malawi
Source: PLoS One. 2025 Sep 9;20(9):e0319691. doi: 10.1371/journal.pone.0319691 (PMC12419581; doi:10.1371/journal.pone.0319691)
Supplement: S1 Data — (ZIP) [file pone.0319691.s001.zip › Supporting Information Files/ET_2FGD.docx]

I: Thank you for your participation. Let us proceed to the discussion now: Can you please tell us the illnesses affecting children below the age of five, in your community?

P2: Diarrhea in a compound inhabited by families having children. I know flu and Tonsillitis are the most common illnesses affecting our community and my family; it is sometimes severe in some children. Even though it is as common as tonsillitis, diarrhea is also another common illness that affects the community.

P8: The most common illnesses affecting children in our community are measles and diarrhea. Tonsillitis is also common here; my two children were victims of this illness. One of them is a Fourteen-Year old and the other is Two, the latter had been ill recently, I thought it was just a ጆሮ ደግፍ but managed to identify the disease later after I brought him to the Health Centre. So, flu and ‘kufign’ (the Amharic name to call measles.) The symptom for kufign is red rashes on the body, and it is still a disease affecting children in this community.

P7: The most common illness affecting children in our community is Stomachache. We don’t know the exact cause of this illness, though. I think it is because they usually don’t wash their hand before eating. I don’t know the cause, but Tonsillitis is also common here, and I even have taken my two children to the Health Centre and managed to help them with treatments for the Tonsillitis illness they had suffered with.

P6: Dry cough is the most common illness affecting children in my family and in our community.

I: Is there any different idea? If there is not, which of these illnesses do you consider to be a burden in this community? Why do you say so?

P3: Most of the time, the causes for illnesses they have mentioned as common are the poor drainage system in our community. Sometimes, the illnesses affect children repeatedly, and we usually can’t find the appropriate treatment from the doctors in the health centre. I think it is because the doctors just diagnose them without any stool taste, but just by asking about the symptoms. Children living in this community are vulnerable to diarrhoea and other illnesses like vomiting as they usually eat together. The people living in this area can’t afford private health services, and there is no other option than using the health centres. The health service costs are increasing, as indication, the Health insurance program registration fee is made to be 1,000 birr per person now. We at least need proper health service for our children, and we want you to link the community with these facility systems. Tonsillitis, Diarrhoea, Stomachache, and appetite loss are most common here.

I: So, which of these illnesses do you consider to be a burden in this community?

P6: They have mentioned it all, but let me share some ideas from my experience. I have a child who is suffering from an Intestinal infection, I still don’t know the cause of that. It might be because of constipation or an inability to defecate smoothly.

I: Okay, please let us just discuss the topics raised. We can answer any other questions after the interview.

P7: I think illnesses related to stomach pain are common. My children are usually affected by diarrhea. To treat this, we used to use prescribed medicine every six months, but we can’t get the medicine again before six months. So, when the children get ill, we must treat them with other traditional treatments like taking in ‘Feto’ (an herb which is believed to be a cure for many diseases.) Other health problems are constipation and hemorrhoids. We usually don’t go to hospitals every time these hemorrhoid-related health problems happen, because we believe the health workers may overcomplicate things.

P5: They mentioned it all, but I think Tonsillitis and diarrhea are diseases we consider burdens in this community. We used to treat these illnesses with ‘Feto.’ But, I am taking them to hospitals after you gave us the orientation last time. We thought, that Tonsillitis was a seasonal illness, and it was caused by sun burn during a warm days, but we now know that it is not.

I: You have mentioned illnesses like measles, stomachache, tonsillitis, diarrhea, and other related illnesses. What do people think are the main causes of children in this community?

P6: I think it is related to hygiene.

I: Would you explain it, please?

P2: I think it is because of poor hygiene and contamination of food.

I: Any other?

P1: I think it is because we don’t wash our hands before we serve food to children. In addition to that, they are vulnerable to bacteria as they usually crawl in the ground.

I: How about the causes for Tonsillitis and the other illnesses?

P: I think it is caused by poor drainage and sun burn and poor hygiene followed by the poor drainage system of toilets.

I: Is there anyone who can mention different causes?

P7: I don’t know that much, but I have children here and one of them is Asthmatic. He is allergic to cold air, the health workers usually prescribe him syrup, but that usually don’t heal.

I: Is that Asthmatic infection or is that something like flue?

P4: He is allergic to cold air. My other child’s ear discharges pus. Doctors have prescribed syrup and eye drop those usually don’t cure the illness even they get well for some time after taking those medicines. The illnesses hasn’t been cured, the illnesses stays for three and four days, the medicine hasn’t not been helping them to recover fast.

P6: Children’s immune system is weak, and this made them vulnerable to diseases. There are many causes for Tonsillitis. It can be caused by poor drainage system, or not washing hands before serving children food. Diarrhoea is very common here and I don’t know how we can eradicate that. We have to work with the health Centre for better solution.

I: If you were to rank these illnesses in order of priority, what would you rate as the top three diseases affecting children?

P7: I should rank Tonsillitis and Diarrhea first and second, respectively.

P3: And also coughing.

I: Any other?

P2: There is also sinus disease. This disease were known to affect adults, but it has also been affecting children recently. We should know the cause for this. Diarrhea and Tonsillitis are very common but the sinus illness is also another disease affecting children as I heard from mothers whose children are affected with that.

I: So, what would you rate as the top three diseases affecting children?

P5: It is diarrhea and Tonsillitis, because the diarrhea doesn’t give time to treatment unless we act faster; it drains their energy. The ORS cannot help them like it does for adults. I actually don’t have the information about the availability of ‘ Lemlem’( Amharic name of ORS for children); it was a good solution. We can conclude that diarrhea should be ranked first.

I: Can you tell me the health services/facilities available in this community?

P6: Health Centres.

P7: Tikur Ambessa Hospital, if we are referred to.

P6: It can also be private hospitals.

I: Where do most of the community access health services?

P7: Konen.

I: Is that private clinic or what?

P7: Yes, there is also Teklehaimanot Higher Clinic. There is also a private clinic called Selam, but most people don’t use that.

P6: Many people use the services from Chechela, which is a private health facility. In addition to that, there is a guy called Mekonen who has been giving the service private,, most people get health services from him.

I: So, which health facility is accessed by most people?

P3: It is Mekonen.

I: How much does it cost to access these services?

P7: There was a time I have spent 3,000 birr for health service at Mekonen, a private clinic.

I: What kind of health service you have had?

P3: It was for treatment for Tonsillitis. I spent 3,000 to get tasted to use ultrasound service, and stool tastes in laboratory.

I: Would you please tell me the average amount of money you spend to access the services? The cost may include, the fee for registry card?

P7: I had paid 200 for card.

I: How about the money you spend for card at Health centre?

P4: It is 40 birr, it was 10 birr. The cost to access the services in the health centre is very low when compared to the cost to access the services in the private health facilities. Unless the medicine you want is unavailable in the facility, you can get the health services with only 200 birr. But sometimes, the Kenema( governmental pharmacies) don’t sell some expensive medicines for the people who use the health insurance system to access health services by just showing the receipt that allows you to pay less. This is because they want to sell it for more profit. So, people can access health services with 500 birr, 200birr or sometimes with less than 100 birr.

I: How about you? Would you please tell me the average amount of money you spend to access the services?

P1: Teklehaimanot Higher Clinic charges you higher. I have a neighbour who brought her daughter there for health service, and she spent over 5,000 birr for services laboratory for blood taste, urinary, and stool taste. She also spent more than 1,000 birr on medicines, which was too expensive for only laboratory service, and she didn’t even have injected with glucose fluid.

I: How long do people have to travel to access the services?

P7: The health centre is close to our residents.

I: Do you have to use transportation to travel to the facilities?

P2: No, you need not; they are very close to residents. It is within walking distance and may take 15 minutes of walk.

I: How do you respond when a child has diarrhea at the household level?

P3: Our mothers thought us to feed and treat children with boiled flaxseed.

P6: It is the same here; we also let the child with diarrhea feed the flaxseed after we boiled and distilled it. It is because Feto is too strong for children. We do that and then take them to the health center if we don’t get cured. For your previous question, the health center is the very closest health facility for all of us. We need to take a taxi for Mekonen and Teklehaimanot hospitals, but we can walk for Chechila or the Health Centre.

P4: I have never treated children at home, but I know people who use Abish (a name for the seed of fenugreek) to treat children diarrhea.

I: How about the response made at home for diarrhea, at the community level?

P4: Some say Feto is good, some suggest you Abish like that. But, nowadays, people are understanding these are not good practices as health workers are creating awareness about the harms of these traditional treatments. We may just give them Tenadam (a herb which is believed to cure illness.). We are taking them hospitals now.

I: Where do they go to access treatment?

P2: Most of them go to health centers. I brought my child to the health center yesterday. A health worker, who is not a doctor has diagnosed my child and prescribed a medicine by telling me it was Tonsillitis. I didn’t trust that and tell them that I need to be checked by the doctor. They have told me they, too, could treat that, but I could also get additional treatment by the doctor. The doctor then diagnosed my child and prescribed him with syrup in addition to the medicine. My baby is well now.

I: Any other? You may also tell me where they access antibiotics when children are affected by diarrhea?

P3: We obviously can only get the medicine from health center.

I: Is that always the case?

P7: Yes, we take the medicine from the health centre.

P6: We take the medicine from the health centre if we are prescribed from health centres. I always purchase medicine from the health centre, but I sometimes buy from other pharmacies when the medicines are unavailable here in the health centre.

I: Any other ideas on where to access the medicines?

P2: We get the medicines from pharmacies.

P6: The health center sometimes refers the children to Tikur Ambessa, Ras Desta, or other hospitals for more treatments. If the medicine are available, we get them from the health centre. Otherwise, we must purchase either from Kenema pharmacies or private pharmacies. There are two pharmacies around us, and we purchase from them.

I: You have told me that you use treatments from home. Why do you visit health facilities then?

P7: The treatments we get at home are very backwards and not certain; we just give these traditional treatments until we get treatments from health facilities. But, we need to get more professional medication and visit the health facilities.

I: What are the enablers and challenges for people in this community to access health services for treating diarrhea?

P1: There may be economic challenges; people may not be able to spend for health services unless they are users of the health insurance system. The private health facilities can’t give you the service for free, so a person with low income may be challenged to access the medicine from Kenema, and may need to borrow from others because it is hard to earn money now a days.

I: So, not being able to pay for the service is the major challenge, right?

P6: Yes.

I: How about you?

P? There are challenges to access the service faster. You have told us we can get the service with out waiting for queue for emergency cases like Acute Diarrhoea and Vomiting, but the reality is not like that. You have to wait long, they only consider bleeding and accidents as emergency. I have registered to use the Health insurance system and I am not expected to pay for the treatments.

I: How about the others. What are the enablers and challenges for people in this community to access the health services?

P4: It is not always economic; there are also other causes like ignorance.

I: How about you? Do you have any other ideas on the enablers and challenges for people in this community to access health services?

P7: The health workers always advise us to take children to the facilities as fast as possible; this is considered an enabler to the use of the services.

I: Have you ever had a health education about diarrhea?

P1: They give us training from home to home. They have advised us to let our children play safely and even train us how to make our children a ball. I appreciate the activities of the health centre. I only spent 5 birr to get delivery service when I gave birth to my two children. The health centre is doing great for poor people like us. The only complaint I have about the service of the health centre is the mistreatment of workers on the registry card. The missed treatments from them sometimes made you think it was better to use the services in private facilities. In general, the health services in this health centre are perfect. The doctors even made their phone available to receive our calls any time we need them to remind you of the appointment date; their follow -up for treating children under the age of five is great.

P2: Can I get outside to call? I have to go for my child who is about to come from school?

I: Okay.

P6: The three doctors have treated me very politely. I have given birth later than I was expected, and they made me check up from the health centre after they got me while I came to take medicine for headache. The nurses and doctors had been treating me well, and the janitors had even been cleaning my rooms without any discomfort. I am really grateful for the quality service our health centre provides.

I: So, the quality service from the health centre has been enabler to access health services, right?

P3: Yes.

I: I also want to add this; I had brought my child five o’clock night. The guard received us well and made the workers awoke to give my child the health service. He told us he had been feelinperformser and mekorte.. They were to prescribe him injections, but I have refused. I have refused it because he hadn’t been tasted or diagnosed; the diseases wasn’t identified. The amazing thing was that the health workers accepted my opinion and took them to laboratory for stool taste and dem. Then, they found it was typhoid and Typhus. Sometimes, the health centre performs better than private health facilities, the nurses create awareness and home to home and do measure hypertension, and check for our children's health. We really appreciate that.

I: Thank you. Please relate that with diarrhoea and try to tell me about the enablers and challenges to access the health services?

P? We mentioned it all.

I:What do people think are the main causes of diarrhoea in children in this community?

P? It is caused by sharing toilets and our lifestyle, which made us live very closer. But, the health extension workers have done well to alleviate these problems by creating awareness and giving information about the people who are affected by some transmitting diseases to take care.

I: So, what do people think are the main causes of diarrhoea in children in this community?

P? Not washing hands before having a meal, not washing dishes well, and not keeping personal hygiene.

P? There is no that much prevalence of diarrhoea in our village. I think it is because we have regular sanitary programs in our village.

P? I think feeding packed foods are major causes of transmission of diseases. The children are obsessed with chips and packed foods that are made unsafely.

P? I am on the same page with her; packed foods are very dangerous. My daughter always gets ill whenever I feed her packed noodles.

I: Any other cause you know, you may consider your living style and other causes.

P? We usually are ignorant when it comes to personal hygiene. For example, I sometimes forget to wash my child’s hands with soap after I drop the faces of my baby into the toilet. It is possible the kids touch something dirty when we are not watching. Adults don’t always wash their hands with soap consistently; we usually react only when our children get sick. My daughter keeps her personal hygiene well because the school she is currently in checks up the fingernails of the student. But I have never done the same for my youngest child, this may made him vulnerable for diarrhoea.

P? The situation that we share toilets is very risky. There are four or five households using a single toilet creates accountability of cleaning the toilet regularly. The toilets may be left untidy and may create a suitable situation for flies and germs that cause diseases. Our residential areas are under standard and not suitable for children. I sometimes even wonder how the time during the COVID pandemic was good. I said this because it had enabled us to keep our personal hygiene to prevent the transmission of the pandemic. There were hanging jars to wash our hands with soaps; there was almost no flue and diarrhoea cases at that time.

*All participants:* *[nodding with agreement]*

P? So, we should keep that up and provide methods to wash our hands after using toilets. We should keep the toilet clean to prevent these diseases.

I: What does the community do in the household level to prevent diarrhoea?

P? We should keep our personal hygiene and wash our hands. For example, the cause for my son to be affected by diarrhoea is her interaction with dogs. There have been many times he is affected by diarrhoea, so I have started to wash our hands every time I serve food and eat. I am washing the utensils with Berekina( detergent.) and wash toilets with my son and neighbor, who also have children. These preventions have brought changes now.

I: Very good, how about you? What do you do to prevent diarrhoea?

P6: I think we should cleanse fresh vegetables like tomato and salad well before feeding.

I: Can you elaborate that, please?

P6: We should immerse the fresh salad and tomato to the solution of lemon, salt, and water. Fresh fruits may look good to eat, but very dangerous if fed with out proper cleansing.

I: How about you, you can mention any diarrhoea prevention method you know?

P? By washing hands and cleanse fresh fruit.

I: Any other? What do people do to prevent diarrhoea in children in this community?

P? By keeping personal hygiene, wash our hands and utensils. This is how we have been preventing.

I: Okay, how about in community level?

P? We should create awareness in Bunna Tetu programs( traditional gathering of people, where people drink coffee and chat.) We should have regular discussion meetings to clean our compound, with out that, everyone can’t participate on cleaning programs. The community can share what they know if we teach everyone.

I: Good, what can be done in community level, to prevent diarrhoea?

P?: We should clean…

I: You may tell me what has been done currently?

P: We have a weekly schedule to clean our surrounding which is covered with grasses where people pee on. We clean weekly every Saturday in group of Two, we collect the rubbish from every outlet and put it in organized way, segregating the rubbish accordingly and send for the sanitary workers.

That is how we clean our environment.

I: How many days a week do the sanitary workers collect?

P: Two days a week; Tuesday and Saturday.

I: Do you think that is enough?

P: Yes.

P? I can witness that their compound is very tidy and they clean it well organized and I harmonized manner, I have observed that when I went to their compound to cut Tenadam. It is not the case in our compound, I wouldn’t have started to clean my surrounding if my child wasn’t ill.

P? There is a drainage ditches problem in our village. We can’t access the ditches easily because they are farther from our resident. We have to wait until our storage tanks are full, as we can’t pour our liquid waste now and then.

I: What amount is the storage you are using to store ?

P: It can store 25-liters of liquid waste. If we want to pour the waste in the ditches before the storage contain full, we need to pay 10 birr. I sometimes pay that because the 25 litres are heavy for me to hold it and travel long. We can’t pour dirty water and urine at surrounding so we need to contain to store and travel long to pour it. So, we need ditches that drain liquid wastage from our compound.

I: Haven’t you discussed with health extension workers about the ways to dig ditches from here?

P:We have told them that, but it is expensive to do that.

I: Do you have any other idea over this?

*All participants: [silence]*

I: If you don’t have any, let’s get into to next discussion point.

P? How do people in this community perceive childhood vaccines?

I: We aren’t usually good at that. For example, I only had the vaccine for only year and six months. There are even families who don’t go for vaccine after Nine months and that is what I have done. I don’t know why, but most people recommend me not to take the vaccine after six months, I want to know why.

I: Okay, is there any other additional idea here?

P? I think we are good on this. For example, I have never interrupted the vaccine in five years and I didn’t want to miss that.

I: How about the community, what is the perception of the community about the vaccines?

P: I think, it is good.

P? The perception of the community about the vaccine is good. But as I have observed in my visits to some households with nurses for vaccines like Polio, there are people who are not willing to take vaccines after the Ninth month. We teach them the benefits with nurses and then they change their mind, it is just because of absence of awareness. But it is not a problem now.

P? I think vaccine is very crucial, and they must not interrupt it; that is what I have done for all of my children.

I: Why do you think childhood vaccines are widely accepted?

P? I think it is because the vaccines are directly related to the health of the child. Missing one vaccination is like risking some part of our children for diseases. So everyone urges to take the vaccine even if they are given in institutions like schools.

I: Very good, any other?

P? It is because we are told the vaccines can prevent children from diseases.

I: Okay, you have told me some families are not willing to take vaccines after nine months. What brought this problem? Why are the families not considering these vaccines like Vitamin D as crucial as the other vaccines?

P? It is just the absence of knowledge.

I: Can you elaborate that?

P: The vaccines are important to prevent disease and for growth. Some families don’t know that. But, the health extension workers are creating awareness from home to home and in public meetings.

I: Okay. What else?

P? The causes are usually ignorance of families.

I: What does the health worker teach you about the vaccines given after the ninth month?

P? I also thought the vaccination after the ninth month was not that important. The health worker has wondered how could I didn’t have knowledge about this, given that I have learned up to grade 10. However, the health extension worker has learned about me and arranged a schedule with me for March and tell me to tell the information for others.

I: Any other additional ideas?

P: Now, I have learned that the vaccines given after the ninth month are very important.

I: Any other?

*All participants: [silence]*

I:Let us get to the next important topic. What do people think about rotavirus vaccines?

P? Come again, please?

I: Vaccine for Rotta virus.

P? I have never heard of this vaccination.

*All participants:[laughter]*

P? That is true; I don’t know about that.

I: It is a vaccine given to prevent diarrhoea for children. You have already been taking that. What do you know about that vaccine?

*All participants: [silence]*

I: How often you take vaccination services ?

P? Every three or six months.

I: What have you taken when you came last time?

P? I have observed that they have given vitamins, injections and droplet.

I: That is it, it is the droplet you have mentioned. You just don’t know the name of it.

P? I see.

I: So, what is the perception of the community about this Rotavirus vaccination? Can anybody tell me the purpose?

P? I think it is for growth, and some people have told me we can take fruit like Papaya instead.

I: I didn’t ask about the vitamin, which is given after 9 months. I am asking about the droplets.

*All participants: [Noice, because these start arguing on what the Rota virus vaccination looks like]*

I: Where do they access rota virus vaccine?

P? I haven’t had knowledge about the rota virus before, but we I have been taking that from health centres.

I: Haven’t there been campaigns to give vaccine service?

P?: Yes, they have been providing vaccine services in tents.

I: What concerns do people have with rotavirus vaccines?

P? There is nothing.

P? There should not be any concern, because the workers from health centres are professionals and know what to do.

I: Okay. For example, you told me there are concerns about the vaccines for Vitamin D injections. Are there any other concerns like this?

P? In my experience, children may feel pain after the vaccine. They may even cry all day because of the pain. I don’t know any other concern than this.

I: What are the enablers and challenges for people in this community to access rotavirus vaccines?

P? The pain my child had after the vaccine was given on the 45th day was unimaginable. It was something given through the anus. The vaccine after six months wasn’t that much painful, but I sometimes don’t want that pain and don’t want to take my child for the vaccine, I just feed him fruits instead.

I: How about you, what are the enablers and challenges for people in this community to access Rota virus vaccines?

P? There are health workers who teaches us everything we should do for the vaccine, the schedule we should take the vaccines. So, it is good and I see no challenge to access the services.

I: How about the economic challenge, or the distance you should travel to get the services or like that?

P: The service is given for free, so there is no challenge rated to that.

I: How about the cultural/regional believes that prohibit the vaccine?

P? There are challenges related to cultural believes. Some cultural believers don’t want the vaccination, because they believe that is against the will of the sprit the believe in.

P? Muslim extremists don’t want to take the vaccine, I know that we’ll. Some denomination of Muslims don’t want to take vaccines after the 45th day.

P? I want the vaccine, though.

*All participants: [laugh]*

P? You are right, but I know some.

I: How about the challenge of COVID pandemic? Had that been the challenge for vaccine?

P? Yes. At that time, there was a fear of getting COVID vaccination, so people may thought it is injection for vaccine. The community is still skeptical of COVID vaccination and they tend to resist other vaccine. Because we don’t exactly identify which vaccine is what until we saw the result. That wouldn’t be happened if the vaccine was in form of tablets.

I: How about the effects of the COVID pandemic related to the movement restrictions to limit the transmission of the pandemic? Had not there been a challenge to go for vaccine due to restrictions ?

P: The people had been confusing the COVID vaccines to the normal vaccine. They didn’t want to take the COVID vaccine, and they thought we were providing the covid vaccination in the name of other vaccine.

I: Okay, were the health facilities available and giving the vaccine service as always?

P: Yes, they were open 24/7. There was ambulance service day and night.

I: Could you use the health services for other diseases other than COVID-19?

P: Yes, all you should do was wear your mask.

I: How about the challenge related to the absence of knowledge about the safety of vaccines and it’s curability?

P? There were none.

I: Wasn’t there awareness problems about the vaccination?

P? There wasn’t. But, after the time of the COVID-19 pandemic, the people had become sceptical of all vaccines. It was because her mother died after having the vaccine, which made the people believe the vaccines were risky to take in. There has been no other challenge than this.

I: Okay. Is there anything you want to add ?

*All participants: [silence]*

I: If you don’t, let us finish our discussion here. Thank you all for your participation.
