# Supplementary material for: Exploration of the social determinants of diarrhoea, rotavirus vaccine uptake, and vaccine ‘fatigue’ in Ethiopia, Kenya, and Malawi
Source: PLoS One. 2025 Sep 9;20(9):e0319691. doi: 10.1371/journal.pone.0319691 (PMC12419581; doi:10.1371/journal.pone.0319691)
Supplement: S1 Data — (ZIP) [file pone.0319691.s001.zip › Supporting Information Files/KY_03FGD.docx]

**FOCUS GROUP DISCUSSION 3. MUKURU KWA REUBEN**

**11 PARTICIPANTS, 6FEMALE& 5 MALES**

**1. Can you please tell us some of the illnesses that affect children in your community?**

*P1 - We are affected by diarrhea due to contaminated water from a burst pipeline in our settlement area. It mostly affects our children who play football without paying attention to good hygiene practices and lack proper waste management sites. As a result, our open fields are filled with menstrual hygiene management products and used condoms, which our children use to make traditional balls. We are also affected by malaria since many of us cannot afford to sleep under a mosquito net.

P2 - We are also affected by measles.

P3 - Children are affected by severe headaches and stomach aches because they touch dirty things and do not wash their hands.

P4 - Our children are affected by marasmus and kwashiorkor since we do not provide them with an adequate, balanced diet of food.

P5 - Children are affected by common flu and coughing since they keep touching cold water and due to climate change***.**

**2. Which of these illnesses do you consider to be a burden in this community?**

*P 7 - We consider diarrhea and coughing as a big burden in our community since the bursting pipes might be contaminated with the diarrhea virus from waste disposal. This contamination could come into contact with water pipes, allowing the virus to enter our bodies and affect the entire community. This is particularly concerning when it comes to washing groceries and drinking untreated water.*

3. If you were to rank these illnesses in order of priority, what would you rate as the top three

diseases affecting children

*P3-The most common diseases affecting our children are 1.diarrhea and cough
P 4 Coughing/common flu
P 6Vomiting
P 8 Measles
P 10 Headache and Stomach ache*

4. Can you tell me the health services/facilities available in this community? Where do you

access health services?

*P2 - We have public facilities, namely Maendelo and Mukuru Kwa Ruben facilities. However, they don't have medicine; they usually refer us to buy from an outside/private pharmacy. Ruben center has medicine, but it is relatively expensive, and most of us cannot afford it. Hence, we prefer going to a chemist to buy drugs over the counter.

P5 - I prefer Ruben Center and Mukuru wa Njenga, but neither of them has drugs.

P6 - I prefer Ruben Center, even though it's expensive; they are concerned.

P 11 - We have EF, which is private. It has drugs, but it's expensive due to transportation.*

**5. How do most people respond when a child has diarrhoea in the home?**

*P2 - I usually give my child ORS and zinc when I see he/she has diarrhea, then watch over him/her. If it persists, I take him/her to a health facility.
P5 - I warm water and give it to him to drink, then take the child to the hospital.
P8 - I boil rice or wheat and give the child the soup to drink. If it persists, I give the child wheat porridge in small portions.
P4 - I give the child bread/toast to eat or wheat porridge.
P3 - For 0-6 months, I give the child yogurt or glucose mixed with water.
P10 - For 0-6 months, a child is given drip water or Bonisan, not boiled water with salt, which can cause constipation.* ***At the community level:*** *P8 - At the community level, we have CHVs who refer children who have diarrhea to a health facility and provide drugs to stop diarrhea on the spot.
P11 - I think a lot of people in our community do take antibiotics, especially when they feel unwell. Some of us prefer going to the local health facility or private pharmacy. We trust the expertise of healthcare professionals to prescribe the right antibiotics. On the other hand, there are instances where people might access antibiotics from the local pharmacy without a prescription. This usually happens when we've had a similar illness before and recognize the symptoms. It's convenient for them, and they believe it saves time and money.* ***Preference of private pharmacy:****P5 - We prefer private chemists since they are easily accessible in the locality and there are no huge lines.
P4 - Adequate drugs and good customer relationships.
P9 - I prefer going to a public facility for disease diagnosis and drug prescription but purchase drugs from the private pharmacy.*

**6. Can you tell me some of the enablers and challenges that people experience to access**

**treatment for diarrhoea diseases**?

***Enablers*** *P2-The availability of over-the-counter anti-diarrheal medications is readily available at local pharmacies. This accessibility allows for quick and easy treatment without the need for a prescription.

P5-Proximity to community health centers/clinics is a significant enabler. Since they are easily accessible by community members, it leads to seeking professional medical advice and treatment for diarrhea.****Challenges*** *P3-Poor water and sanitation infrastructure making people more prone to diarrheal diseases.

P5-In some areas, the lack of nearby healthcare facilities poses a significant challenge. Long travel distances/huge lines and transportation issues hinder us from accessing timely medical care for diarrhea.

P6-Even if treatment is available, financial constraints prevent us from seeking medical help. The cost of medications, consultation fees, and other healthcare expenses hinder us from receiving the services.*

7. What do people do to prevent diarrhoea?

***At the HH Level.****P3- Practising good hygiene through boiling drinking/adding chlorine to water,washing hands after visiting latrines/giving child food.
P7-Ensuring our food is properly cooked,drinking water is boiled.
P8-We should avoid streat food since they cooked in ditches where there are housefly and also we should practise wash hands after visiting latrines/changing child diaper at the HH level****.****P7-Cleaning utensils and having a waste disposal at the Compound* ***At the community level:****P1-Having waste disposal sack at the plot level rather throwing waste anywhere at the plot.
P4-Disposing feaces into the pit latrine rather than throwing it above the plot roofs,having improved latrines i.e pit latrines*.

8. How do people in this community perceive childhood vaccines

*P2- In our community, childhood vaccines are generally seen as a crucial preventive measure against diseases. Many parents here believe that vaccines help protect their children from serious illnesses.*

*P 8 Community members perceive vaccine immunizers as people who are not medically qualified to offer the service since they are community health volunteers.*

*P4- The widespread acceptance of childhood vaccines could be attributed to the success stories we've heard. Many families have seen the positive impact of vaccinations – healthier children and a decrease in certain diseases*

**9. How about rotavirus vaccines? What do people think about rotavirus vaccines? Where do**

**they access rotavirus vaccine?**

*P1- Religiously, we have a church that bars members from getting the rotavirus vaccine.

P5- Most of the community members are not willing to get the vaccine since it's done each and every time, i.e., Polio vaccine is done more than thrice a year. Hence, the community has a taboo that it might cause other diseases in the future.

P1- A high number of women make the decision on which vaccine to give to the child. Most males don't even know at what age a child should be given a specific vaccine.

P1- During COVID time, vaccines were offered by appointment only.

P5- During COVID time, most of us didn't take our children to the health facility to get the rotavirus vaccine since it was mandatory to get the COVID vaccine before getting other health services, i.e., the vaccine for the child.*

**10. What are the enablers and challenges for people in this community to access rotavirus**

**vaccines?**

*P2 - One key enabler for accessing rotavirus vaccines in our community is the availability of vaccination clinics at convenient locations.

P3 - The cost of the rotavirus vaccine is low or covered by government programs.

P5 - A positive perception of vaccinations and understanding their importance can facilitate higher vaccine uptake*.
