# Supplementary material for: Exploration of the social determinants of diarrhoea, rotavirus vaccine uptake, and vaccine ‘fatigue’ in Ethiopia, Kenya, and Malawi
Source: PLoS One. 2025 Sep 9;20(9):e0319691. doi: 10.1371/journal.pone.0319691 (PMC12419581; doi:10.1371/journal.pone.0319691)
Supplement: S1 Data — (ZIP) [file pone.0319691.s001.zip › Supporting Information Files/KY_13FGD.docx]

**FOCUSED GROUP DOISCUSSION 13**

**09/05/2024**

**NUMBER OF RESPONDENTS- 10 FEMALES**

**1. Can you please tell us some of the illnesses that affect children in your community?**

**R1-**Diarrhoea.

**R2**-Malaria.

**R3-**Typhoid.

**R4-**Flu.


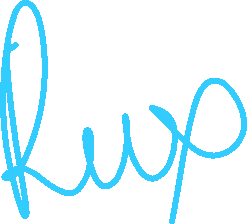


**2. Which of these illnesses do you consider to be a burden in this community? Why do you say**

**so?**

**R4**-Flu and malaria are the most burdensome in the community**.**


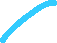

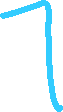


**R5-**Diarrhoea, malaria and flu. Our environment is not that conducive and therefore this causes diarrhoea in the children.


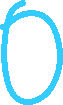


**R6**-There are many mosquitoes in the community hence making malaria burdensome.

**R7-**There are times when the child gets flu we as parents are the reason because we do not take care of the child or protect them. For instance, you might find the child playing outside without a sweater on when it’s raining or they wake up early and go out to play without the parent properly dressing them. Sometimes they get malaria because as parents we have not protected them with mosquito nets when they are sleeping. This is what causes issues and problems.


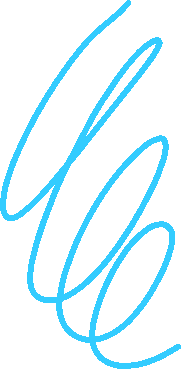

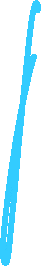


**3. If you were to rank these illnesses in order of priority, what would you rate as the top three**

**diseases affecting children**

**[If diarrhoea is not listed in the top three priority problems -Do you think diarrhoea diseases**

**are a big problem in this community or not? Why do you say so?]**

**R4-**The first is flu followed by malaria and then diarrhoea.

**R8-**The first is diarrhoea then flu and the third is typhoid.

**R9**-Malaria and fever.

**4. Can you tell me the health services/facilities available in this community? Where do you**

**access health services? [Probe: how much does it cost to access these services, how long do**

**people have to travel to access the services.**

**R10-**The hospitals nearby are EF and Maendeleo but my child is usually treated at EF.

**R4**-I also take my child to EF which is like an NGO and a walking distance from my home.

**R6**-I take my child to Mukuru City Council Hospital because the treatment there is not financially constraining and the medicines are free there.

**R2-**I take my child to EF because they never lack medicines as opposed to Maendeleo.

**R5-**I take my child to St. Mary’s which is here in Njenga. I prefer that hospital because I can easily access the medicines prescribed there at a nearby chemist.

**R9-**I take my child to Our Lady because it is nearby and services are free.

**R10**-I take my child to EF because it is next to where I live and services there are fast.

**R8-**The child is taken to EF because it is the nearby hospital and treats everyone in the community.

**5. How do most people respond when a child has diarrhoea in the home? [Probe: What do**

**people do at household level? at community level? Where do they go to access treatment? Do**

**they take antibiotics? Where do they access antibiotics? Why do they access antibiotics?]**

**R9-**The first thing that I do is boil some water, add salt and give the child.

**R4-**I have ORS at home which I was given at Mbagathi Hospital, I give this to the child.

**R6-**I give the child ORS first which I either buy from the chemist or ask from my neighbors, later on I take the child to the hospital.

**R2-**The first aid that I give the child is boiling water, add some salt and sugar and give the child. Later on, I take the child to the hospital.

**Where Antibiotics are accessed.**

**R4-**We get them at the hospital where we are given.

**R6-**We buy them at the chemist.

**R3**-I bought them at the chemist where I explained the child’s condition and was prescribed Amoxil.

**6. Can you tell me some of the enablers and challenges that people experience to access**

**treatment for diarrhoea diseases?**

**R4-**There are times when you are prescribed medicines but you do not have money to buy them.For instance, when you go to buy the prescribed medicine at say Transchem, you find that it costs about 500 to 1000 shillings but when you go to a local chemist, you find a generic version of the medicine at a lower price of about 200 to 300 shillings which is more pocket friendly.

**R3-**Mostly the challenge is finances in buying the prescribed medicines which are not given at the hospital.

**7. What do people do to prevent diarrhoea? [At household level, at community level?]**

**R7**-The first thing is observing cleanliness both inside and outside the home.

**R4**-Boiling the drinking water or treating it and washing vegetables with your own water instead of them being washed by the vendor.

**R9**-Maintaining cleanliness and a clean environment that is surrounding the child.

**8. How do people in this community perceive childhood vaccines [Probe: why do you think**

**childhood vaccines are widely accepted? Why do you think childhood vaccines are widely**

**resisted?**

**R6-**I only see the vaccines that are taken around in the community by community officers though I do not know them.

**R5-**My husband has never been against childhood vaccines and all our children are vaccinated.
