# Supplementary material for: Exploration of the social determinants of diarrhoea, rotavirus vaccine uptake, and vaccine ‘fatigue’ in Ethiopia, Kenya, and Malawi
Source: PLoS One. 2025 Sep 9;20(9):e0319691. doi: 10.1371/journal.pone.0319691 (PMC12419581; doi:10.1371/journal.pone.0319691)
Supplement: S1 Data — (ZIP) [file pone.0319691.s001.zip › Supporting Information Files/MW_7FGD.docx]

**F:** Thank you very much for giving us an opportunity so that we should discuss today. There are few things that I want us to discuss, we are going to discuss about diseases that are common among the children here in Bangwe. We are also going to discuss about Diarrhea, how it is prevented, as well as what you think as the major factors that increases of cases of Diarrhea here in Bangwe. We are also doing to discuss about what people normally do when they are not feeling well. Furthermore, we are also going to discuss about vaccines for children including Rota vaccine. Basically, we are going to discuss about this. First of all, I would like to know the diseases that mostly attacks children here in Bangwe, anyone of us can start.

**00:** Diarrhea, Vomiting.

**F:** Diarrhea, Vomiting, remind me your number.

**P 7**: P 7.

**F:** Please raise your voice, she has mentioned of Diarrhea and Vomiting, what are other diseases that mostly affects children here?

**P 8:** Stunted growth.

**F:** We all have children here or our children do not from any disease?

**P 4:** Skin sores also known as Chicken pox.

**P 10:** Cough.

**P 1:** Persistent stomachache.

**F:** How do you know that children have persistent stomachache?

**P 1:** They mostly cry.

**F:** What name to you call that diseases that makes children to have stomachache?

**P 1:** I think its Diarrhea.

**00:** Yes, it is Diarrhea.

**F:** Diarrhea?

**00:** Yes.

**F:** What else?

**P 9:** Most of the children suffer from Diarrhea and others have difficulties in breathing.

**F:** Difficulties in breathing?

**P 9:** Yes.

**F:** What do you call that disease?

**00:** Silent.

**F:** I just want to know diseases that most of the children suffer from here in Bangwe or let me say in your respective communities? You have mentioned of Diarrhea, Difficulties in breathing, Malaria, Chicken pox, Flu and Cough. Are there other diseases that you are thinking of?

**P 10:** Pneumonia.

**F:** So out of these diseases that you have mentioned, which ones do you consider as burden in this community?

**P 4:** Cholera.

**F:** Mmmh.

**P 9:** The diseases that are common among the children here are Diarrhea and difficulty in breathing.

**P 8:** Malaria.

**F:** It is also a major problem?

**P 8:** Yes.

**F:** Malaria, Cholera, Diarrhea, Pneumonia, Difficult in breathing. Are there other diseases that you consider as a burden here?

**P 8:** Chicken pox.

**F:** We are now trying to look at the diseases which you consider as a burden here in Bangwe? out of those that you have mentioned earlier.

**00:** Diarrhea.

**F:** Why do you consider Diarrhea as the major issue among the children here in Bangwe?

**P 2:** Because there is shortage of clean and safe water here in Bangwe.

**F:** Please raise your voice.

**P 4:** There is scarcity of clean and safe water here, there are no boreholes here and most of the people rely on water that is fetched from the wells that are not covered.

**P 3:** Just to add on what she has said, Diarrhea is an issue here because most of the people who stay close to the rivers where most of people fetch water for domestic purposes the usually use these rivers as disposing sites for used baby diapers and this is the main that I would say that most of the children suffer from Diarrhea here in Bangwe/Mpingwe.

F: Let hear from others, what do you think contributes to more cases of Diarrhea among children here in Bangwe?

**P 9:** Most of the people are not able to practice hygiene in their households, this makes the kitchen utensils as well as those used in feeding the children exposed to germs that are responsible for causing Diarrhea in children.

**F:** You have said that most of the children here suffer from Diarrhea because of shortage of water, right?

**00:** Yes.

**F:** What kind of water do you think is scarce?

**P 4:** Water that is used for drinking and other domestic purposes such as cooking since most of the people use water fetched from the wells for domestic use.

**P 8:** Most of the people eat food before washing their hands after visiting the toilet and this makes one to suffer from Diarrhea easily.

**P 10:** Most of the people who are involved in the business of selling already cooked food don't cover the food appropriately and this makes people who buy and eat such food directly to suffer from Diarrhea.

**P 3:** Shortage of pit latrines, some of the people do not have pit latrines as such they practice open defecation and this places people at the risk of Diarrhea.

**F:** You should raise your voice as you are taking, please P 5 and P 6 please let’s take part in this discussion.

**00:** Alright.

**F:** Where do you fetch water that you consider to be clean and safe?

**P 1:** Water from the boreholes.

**P 10:** Water from the taps that is supplied by Blantyre Water Board is the one that we regard as safe because it is treated before it is to people.

**F:** Why do you think people still use water that they consider to be unsafe while they are aware that safe water is from the boreholes and water board?

**00:** There is shortage of the boreholes here, of course we know that water from the boreholes is safe. There are few boreholes here and we don't have taps here that's why we are still having more cases of Diarrhea here.

**F:** So here you are able to fetch water easily from the boreholes and wells?

**00:** From the wells.

**F:** What kind of wells are found here?

**00:** The ones that people dig.

**00:** The ones that people dig along the rivers and some of the water I these wells come from the pit latrines that are nearby.

**F:** Alright, we also mentioned about Malaria as one of the commonest diseases here, right?

**00:** Yes.

**F:** Why do you think Malaria is an issue here?

**P 1:** Shortage of mosquito nets, most of the people do not sleep under a treated mosquito nets and this exposes them to Mosquito bites.

**P 10:** Malaria is a big problem here in Bangwe because of the presence of stagnant waters and most of the people dispose wastes anyhow and this acts as bleeding grounds for Mosquitos.

**P 8:** Some of the people have mosquito nets but they don’t like sleeping under mosquito nets because most of the people believe that Mosquito nets cause difficulties in breathing as well as skin burns.

**F:** What other issues do most of the people have as a result of using mosquito nets?

**00**: (All) Silent.

**F:** What do most of the people say about the use of mosquito nets here?

**00:** Some of the people complain that Mosquitos nets suck blood.

**F:** Mmmh.

**P 10:** Some of the people do not like to sleep under a Mosquito net because in their houses there are other insects such as bed bucks and they believe that use of these nets encourages the spread of such insects. Furthermore, some of the people believe that they have difficulties in breathing when they sleep under a mosquito net.

**F:** You have said that mosquito net suck blood, how do they suck blood?

**00:** People just say that the chemicals that are applied to the mosquito nets suck blood.

**F:** P 5 and P 6, please it’s like am targeting you but I haven’t heard anything from you.

**00:** Please let's not forget to mention our numbers as we are speaking.

**F:** As you can see most of us are speaking but not P 5 and P 6, some people might think that we have just written these numbers if you will not speak because we have already recorded your numbers and names, just be free to speak when you think you have something to say in whatever we are discussing. Am not angry with you but in a group like this, it is good for each one of us to speak.

**00:** Alright.

**F:** We have highlighted inability of the people to sleep under treated mosquito nets, presence of stagnant waters as main factors behind high cases of Malaria in this community, right?

**00:** Yes.

**F:** Are there other factors do you think contributes to high cases of Malaria?

**P 6:** I agree that presence of stagnant water is the major contributing factor here in Bangwe and that some of the people dispose their wastes anyhow.

**F**: How do stagnant waters contributes to increases of Malaria cases?

**P 6:** The stagnant water acts as the bleeding ground of mosquitos which are responsible for causing Malaria.

**P 3:** Just to add on what my colleague has said, unwillingness of the people to sleep under a treated mosquito nets as well as some of the beliefs that people have contributes to the spread of Malaria here. Some of the people believe that use of mosquito nets causes difficulties in breathing and some believe that the mosquito nets suck blood in our bodies. So, I would say that there are some of the beliefs that restricts people from sleeping under a mosquito net and this exposes them to mosquito bites that leads to Malaria.

**P 1:** Just to add, the government is trying its best to distribute the nets but there are some people who usually engage into business of selling these nets once they have receive the nets for free from the government and on top of that they are some vendors who move around our communities buying nets from the people.

**F:** They buy the nets for what use?

**00:** It is mostly those who sell charcoal.

**00:** Some are fishermen and they use the nets in drying fish.

**00:** Some buy the nets here and sell them to the fishermen at the lakeside.

**F:** Which Lake, do we have any lake in Blantyre? (Laugh)

**00:** Some people come all the way from Mangochi and Salima to buy the mosquito nets here.

**F:** Oooh?

**00:** Yes, that is happening.

**F:** So, there are so many reasons why people are not using the mosquito nets, right?

**00:** Yes.

**F:** Now let's discuss about Pneumonia, why do you think Pneumonia is an issue here?

**P 2:** It’s due to the fact that in most cases most of the parents do not provide their children with warm cloths when it is cold. Some people leave their children naked when it is cold while some people cannot manage to buy cloths that can protect their children from cold.

**F:** Why do some parents fail to provide their children with necessary cloths when it is cold?

**00:** Some cannot manage to buy such cloths and some people usually leave their children with other people when moving out of their houses and those people do not take proper care of the children.

**F:** P 7?

**P 7:** What I wanted to say has been mentioned already.

**F:** I just want each one of us to be open, there is no right or wrong answer here. You just have to give us your views based on what you know because at the end we are not going to mark your responses. We just want to get your experiences as well as what you know. You have mentioned of not providing the children with the cloths that can protect them from cold, what else are you think as another reason why there are more cases of Pneumonia in this community?

**P 5:** Some of the parents do not have beddings for their children,

**F:** Lack of beddings?

**P 5:** Yes.

F: Out of all the disease that you have mentioned, you have said that each and every disease here is dangerous. However, I would like you to give me three diseases that you regard as the most dangerous among the children here in Bangwe?

**P 8:** Pneumonia.

**P 2:** Cholera and Diarrhea.

**P 5:** Chicken Pox.

**P 3:** Asthma.

**F:** Asthma?

**P 9:** I would say that chicken pox is just something that that is new but all these times most of the parents were complaining that their children suffered from Asthma and Diarrhea.

**F**: Are there many children who suffer from Asthma in this community?

**00:** Silent.

**F:** Because if we regard it as a problem, we need to think of how many children are suffering from Asthma here in Bangwe. So can we say the major problem here is Diarrhea?

**00:** (All) Yes.

**F:** Do we all agree with this?

**00:** (Yes).

**F:** What else?

**P 1:** Malaria.

**F:** So, we should take Malaria as number 2?

**00:** (Yes).

**F:** Do we all agree with this?

**00:** (Yes).

**F**: The third one?

**00:** Asthma.

**00:** Is there any medication of Asthma for children available at the hospital for?

**00:** Yes.

**00:** Because when we go to the hospital with this problem, we are always told that they have drugs for adults and not children when it comes to this disease. In most cases, adults are given inhalers but not the children. I have two children and they both suffer from Asthma and I myself I also suffer from Asthma but when I go to the hospital they tell me that there are no drugs for my children but for me.

**F:** What do you mean when you say that there are no drugs for Asthma?

**00**: I wanted to know if there are drugs that cure this disease.

**F:** What do you do on your own? To assist these children when they are having Asthma?

**00:** I usually go and buy Asprin syrup at the pharmacy.

**F:** When you go to the hospital, do they tell you that they have run out of the drugs or this disease has no drugs that can cure it?

**00:** At the hospital they have never disclosed that these children have Asthma.

**F:** Then what do they say?

**00:** They just tell me that it is flu.

**P 9:** When going to the hospital, most of the people know that their children have Asthma but they don’t disclose about this to the health care workers. Most of the parents just say that their children are suffering from cough or flu and the doctors assist the child according to the information that they got from their parents.

**P 3:** I have a question, between the doctor and the parent of a child, who has expertise in knowing diseases that a child is suffering from?

**F:** Our colleague here has asked a question.

**00:** Between the doctor and the parent of a child, who has expertise in knowing diseases that a child is suffering from?

**P 2:** Doctors.

**F:** Mmmh.

**P 9:** I would say as a parent you can go to the hospital knowing that your child is suffering from Asthma but the doctors will not accept that, they just give you medication for flu and cough.

**P 3:** Just want to add, there are two kinds of Asthma, one is hereditary and the other that one can acquire after birth. Like at my home village, Nsanje, when people Asthma they are given some herbs that cure this disease. Cases of Asthma are very common when it is cold and what I know about Asthma is that when one is Asthmatic, his/her lungs are filled with water and at the hospital, they just help one to have enough breath but there are some herbalist who are able to cure Asthma.

**P 4:** Sometimes when we get to the hospital, we are afraid to tell them that our children are having difficult in breathing because we fear that they might tell us that our children are suffering from COVID-19.

**F:** (Laugh).... You see we have reached a point where some are saying that at hospital, they are not assisted while some say that the parents do not reveal truth about their children sickness to the doctors.

**00**:(ALL) Laugh.

**F:** Someone else asked a question that between a doctor and the parents who is an expert when it comes to knowing the diseases?

**00**: Yes.

**F:** And we have answered that it is the doctor, right?

**00:** Yes,

F: I don’t know about this but I just wanted to ask, since we are discussing. I have a question, if you would be suffering from headache and the doctor tell you that you are suffering from stomachache, how would you feel?

**00:** I would believe the doctor since is the one telling me the problem I have.

**00:** It would be very painful because you are having headache and the doctor is telling you that you have stomachache, that would be painful.

**F:** How would it be painful since the doctor has expertise of diseases?

**P 8:** If one can go to the hospital because he/she is having headache and at the hospital they are telling you that you are suffering from stomachache, what drugs are they supposed to give you. Are they going to give you drugs that cure stomachache or headache?

**F:** You see, you are providing answers to the question that was raised on your own.

**P 8:** The problem is that when one goes to the hospital, he/she doesn't go straight to tell the doctors that my child is suffering from Asthma because if that happens the doctors usually tell you that if you knew that your child has Asthma, then you could have just bought drugs at the pharmacy. It is like you are trying to test the doctors since it is like you already know what your child is suffering from before meeting the doctors.

**F:** Is it ok for me to provide answers to that question later? I can forget, please remind me.

**00:** Alright.

**F:** Sure. Let's proceed with our discussion, Here in Bangwe when you are not feeling well, what do you do for you to get better?

**P 8:** We usually go to the hospital.

F: He says he goes to the hospital.

**00**:(All) Silent.

**F:** Why do you decide to go to the hospital?

**00:** At the hospital its where they tell you the real disease that you are suffering from.

**P 4:** We usually go to the hospital to know what we are suffering from and to get appropriate medical assistance.

**F:** They have mentioned that they go to the hospital, what about others?

**00:** (All) Silent.

**F:** Can we say that each and every person here in Bangwe goes to the hospital when he/she is not feeling well?

**00:** No.

**00:** Some just buy painkillers such as Burfen.

**F:** Please be open, just say what you usually do.

**00:** We just buy pain killers in the groceries.

**F:** Some goes to the hospital while some buy drugs, what else?

**P 3:** Just to add, when one fell sick, he/she it is recommended that one should go to the hospital because at the hospital, they do some diagnosis to find your real problem and after that they give you appropriate drugs unlike just going to buy drugs at the grocery store where they just give you the drugs without doing some diagnosis but at the hospital, they are able to do some diagnosis and give you appropriate drugs.

**F:** You have said that it is recommended that when one gets sick, he/she is supposed to go to the hospital. Now what makes some people just to buy drugs when they get sick and not go to the hospital?

**P 4:** Because of distance, some people say very far from the hospitals.

**P 3:** Just to add, some people do not like going to the hospital because now it is a mandatory for each and every person to be tested for HIV before being assisted at the hospital. As a result most of the people go straight to buy drugs the private pharmacy. The problem with the pharmacies is that sometimes they sell us drugs that are expired.

**F:** What else prevents people to go to the hospital when they fell sick, you have mentioned of distance, what else has been mentioned?

**P 3:** people are afraid that they will get tested for HIV at the hospital.

**F:** Why are some people afraid that they are doing to be tested for HIV at the hospital?

**P 3:** Some people are afraid to be tested because they drought themselves of having HIV but for a person like me that is not an issue. Whenever I fell sick, I usually go to the hospital and I am always ready to get teste for HIV but those who have multiple sexual partners they are always afraid.

**00:** This is one of the factors that discourage people from going to public hospitals.

**F:** We were looking at what people normally do when they are not feeling, some say they go to the hospital while some say they just buy drugs, right?

**00:** Yes.

**F:** You mentioned something about distance, now I just want to know. How long do most of the people travel to get to the hospital? How far is the clinic to the areas that are in the boundaries between Bangwe and others townships?

**P 1:** We can't estimate the distance but in terms of the money that we use for transport is K 1, 500.00.

**F:** Go and back or one way?

**00:** One way.

**F:** Is there something that you want to add?

**00:** (All) Silent.

**F:** Alright, now I want us to discuss what you normally do when a child is having Diarrhea?

**00:** (All) Silent.

**F:** Each one of us has a child under 5 years of age, right?

**00**: (All) Yes.

**F:** Have they suffered from Diarrhea before?

**00**: (All) Yes.

**F:** Alright thanks, now just want to know what you usually do when your child is suffering from Diarrhea for him/her to get better?

**00:** We usually make Thanzi ORS (sugar and salt solution) and give them to drink.

**F:** Please raise your voice.

**00:** I was saying that when a child is having Diarrhea, we make a solution of sugar and salt and give him/her to drink.

**P 1:** For those children who are not yet introduced to the hard foods like porridge, it is recommended that the woman should be breast feeding the child excessively for the child who is having Diarrhea.

**P 2:** When a child start having Diarrhea during the night, we need to give him/her a lot of water and he/she should be taken to the hospital the first thing in the morning.

**P 3:** When some people don’t have money to buy ORS, they prepare a solution made up of sugar and salt.

**F:** We should remember that there are some people who stay very far from the hospital and that we don't always go to the hospital in most cases, So I just want to learn what you usually do on own to help a child who is having Diarrhea.

**P 7:** Some boil water and add some sugar and give it to the child.

**F:** You are saying that some, what about you? What do you do?

**P 7:** (Laugh).

**F:** Alright, P 9?

**P 9:** Sometimes we give the child SOBO and it helps in helping the child to stop having Diarrhea.

**P 10**: Mostly we give them porridge.

**F:** You have mentioned that sometimes you buy drugs, right?

**00:** Yes.

**F:** Can you tell me the types of drugs that you buy? Where do you buy these drugs?

**P 4:** When young children are having Diarrhea, we usually buy Caffeino.

**F:** How does it help?

**P 4:** In most cases children have Diarrhea when they start to develop teeth for the first time and at the hospital we are usually given Zinc that helps stopping Diarrhea.

**P 10:** Due to the fact the hospital is very far, there are some diseases that we are required to take our children to the hospital. However, regarding high cost of transportation to the hospital, we just buy drugs at the pharmacy and we normally go to the hospital if the condition of the child becomes worse.

**F:** What kind of drugs do you buy?

**P 10:** Pain killers like Panado, Amoxicillin, Bactrim, Fragile. We usually buy drugs that are used to deal with the disease that our children suffer from frequently. When we get to the hospital we are asked if whether we give the children any drugs before coming to the hospital.

**F:** Are there other drugs that we usually buy for our children apart from the ones that have been mentioned?

**00:** ORS.

**F:** Have we ever used drugs called anti-biotics before or we use them?

**00:** (All) Silent.

**F:** Do we all know what anti-biotics are?

**00:** (All) No.

**00:** I just heard about these drugs but I have never seen them.

**F:** You heard from who?

**00:** From the radios but I have never come across them.

**F:** What messages about these drugs are being said on the radios?

**00:** I forgot (Laughing).

**F:** Aright thanks, you have mentioned about distance, fear of being tested for HIV as some of the reasons that prevents people from going to the hospital. Now I just want to know from you some of the factors that encourage or discourage people from going to the hospital apart from those that have been mentioned already said.

**P 4:** (Not clear).

**F:** Please raise your voice.

**P 4:** in most cases hospitals are congested meaning that one has to wait on the queue for a long period of time before being assisted and this discourages people from going to the hospital.

**F:** Alright is there anything else, you have mentioned of long distance to the hospital, long queues at the hospital, fear of being tested for HIV at the hospital as some of the factors discouraging people from seeking for help at the clinic, right?

**00:** (All) Yes.

**F:** What about factors that encourages people to go to the hospital? What do you think are some of the factors that encourages people to go to the hospital in this community?

**00:** (All) Silent

**F:** You are all quit. Alright, let us proceed with our discussion, we have looked at things that increases cases of Diarrhea in Bangwe, now I just want us to discuss about ways of averting the disease.

**00:** (All) Yes.

**F:** How do we prevent Diarrhea at home or in our communities?

**P 4:** By practicing hygiene. For instance, I always make sure that I wash hands after changing baby diapers, visiting toilet as well as before breast feeding my baby.

**00:** Practicing hygiene and eating food that is clean.

**F:** What do you mean when you say clean food?

**00:** The food that is covered after being prepared.

**F:** P 5 how do you prevent Diarrhea or in your opinion, how can you prevent Diarrhea?

**P 5:** Taking care of the house

**F:** In what way? what do you mean when you say taking care of the house?

**P 5:** Practicing hygiene.

**F:** What do you mean when you say practicing hygiene?

**00:** Maybe she is trying to mean treating water from the wells with Chlorine before it is consumed.

**P 5:** Cleaning the toilet with the water that has been treated with Chlorine.

**00:** Having waste bins at home as well as washing hands after visiting the toilet.

**F:** Do people here in Bangwe have buckets that are used for washing hands after visiting the toilet?

**00:** Yes, most of the Muslima.

**00:** Not only the Muslims.

**00:** Some cover the pit latrine's hole always.

**F:** Do most of the people cover their pit latrine's hole with a lid? because here we are discussing what is being done or what should be done.

**P 9:** Some people do not wash their hands after visiting the toilet deliberately.

**F:** Aright, what you have said are some of the strategies that are followed to prevent Diarrhea at home, are there other strategies that are followed at the community level in preventing Diarrhea?

**P 1:** Sleeping under a treated mosquito net.

**F:** Diarrhea?

**00:** (All) Laugh.

**F:** Maybe you were thinking about ways of preventing Malaria, right?

**00:** Yes.

**F:** I wanted to know what is being done in our communities in order to deal with Diarrhea?

**P 2:** There are HSAs who move from door-to-door distributing Chlorine.

**F:** What else?

**00:** There are also messages that are spread in the communities through community awareness campaigns on ways that people can follow to protect ourselves from Diarrhea.

**F:** It looks like most of you have things to say but you are droughting yourself.

**00:** Yes, some are afraid that when they speak people will laugh at them.

**F:** No, don't be shy, we are here to share one another what we think or know. I have already said that during discussions like these there are no correct or wrong answers. Please be open.

**00:** Meaning that when listening to the what we are saying here at your office you won’t laugh?

**F:** What are we going to benefit by laughing at your responses? that won't happen ma'am, I don’t know everything, had it been that I knew these things, I couldn’t bother to come here.

**00:** Just to add, this is like a normal conversation between people therefore it is impossible for us not to laugh as we are discussing.

**F:** Like we laughed when someone else said one of the ways of preventing Diarrhea is through sleeping under a mosquito nets, we were laughing not because he was wrong but he was answering about the ways of preventing Malaria. Laughing in discussions like these helps in making sure that everybody is able to participate.

**00:** (All) Laugh.

**F:** Alright, we have discussed a lot on this, now let us discuss about the vaccines. I believe we all know vaccines that are administered to children.

**00:** (All) Yes.

**F:** Is there anyone hear whose child has never been vaccinated?

00: (All) No.

**F:** Is there anyone among us who doesn't know vaccines that are administered to children?

**00:** (All) Yes.

**F:** Now I just want to know what people here in Bangwe say about vaccines that are given to children? What do people about these vaccines?

**00:** Doctors or people in the communities?

**F:** People in this community.

**P 9:** I remember this other day I came across some women who were discussing about Cholera vaccine and they were saying that since this vaccine is administered more than once to children, it is responsible for causing Cholera and I would say that this is one of the reasons why most of the people do not allow their children to get vaccinated.

**P 8:** Most of the people are not willing to get their children vaccinated because of what was being said during the time when there was COVID-19 whereby people were speculating that COVID-19 vaccine contained some chemicals that causes death to those who got vaccinated so most of the people are afraid that their children would die if they are given any vaccine.

**P 10:** I can say that here people are fond of speaking a lot of things about any vaccine. For instance, there is a contraceptive vaccine that is administered to women and most of the people say that this vaccine is responsible for causing impotence in men as well as gastric cancer but at hospital we are told that most of the people who prone to gastric cancer are the elderly.

**F:** I just want to know, what factors do you think encourages people here in Bangwe to get their children vaccinated?

**P 1:** Information that is provided to people before they are vaccinated encourages people to get vaccinated. People are told that once they are vaccinated, they will be protected from other diseases.

**P 9:** Most of the people are motivated due to the community awareness campaigns that are conducted by the HSAs and some people get vaccinated when they heard that someone else is suffering from a particular disease that they regarded as an illusion at first.

**F:** P9 has said some people get vaccinated because the health care workers talk of advantages of the vaccine, but you as people from Bangwe do you know any advantage of the vaccines that are administered to children?

**P 3:** Polio is a dangerous disease and the vaccine helps in boosting the immune system of the child so that he/she should not suffer from Polio which has an effective of causing deformed limbs such as arms and regs in children if they are not vaccinated.

**P 4:** Vaccine helps children to grow healthily.

**P 11:** Vaccine is very useful as it protects children from different diseases and for them to grow with good heath like vitamin vaccine helps in supplying essentials vitamins in bodies of children.

**F:** Alright, you have mentioned about Polio vaccine, right?

**00:** (All) Yes.

**F:** What other vaccines that are administered to children are you aware of?

**P 2:** Rota virus vaccine.

**P 3:** Vaccine for Measles.

**F:** You know which vaccine?

**00:** Cholera.

**P 9:** Rubella.

**F:** Mmmh.

**P 9:** Rubella.

**F:** Which vaccine is that?

**P 9:** Vaccine for Rubella disease.

**P 10:** thUmba.

**P 11:** Polio vaccine.

**P 3:** When the HSAs are moving from door to door, some people do not allow their children to get vaccinated. Only those who know the advantages of getting their children vaccinated are the one who accept that their children should be vaccinated but most of the people are rude to the health workers when they come in their homes to vaccinate their children. The fun thing with such people is that they usually take their children to the hospital when they are sick.

**F:** I just want to know, what makes most of the people to be rude to the health care workers when it comes to vaccinating their children?

**00:** Some its due to their churches they are against the vaccines.

**00:** Aaaah will not comment on this (Laugh).

**F:** Now let's look at this issue you raised about COVID-19 vaccine, now I would like to know how the coming of COVID-19 affects how parents perceive vaccines for children here in Bangwe.

**P 8:** The coming of COVID-19 discouraged a lot of parents from getting their children vaccinated like at Bangwe health center people could go when they were looking health and they were pronounced dead while they were at the hospital. So due to this most of the people are afraid to allow their children get vaccinated because they are afraid that they could die after being vaccinated.

**P 8:** The coming of COVID-19 discouraged a lot of parents from getting their children vaccinated like at Bangwe health center people could go when they were looking heath and they were pronounced dead while they were at the hospital. So due to this most of the people are afraid to allow their children get vaccinated because they are afraid that they could die after being vaccinated.

**F:** P 9?

**P 9**: Let someone speak.

**P 3:** [Not clear}

**F:** I just want to know how COVID19 vaccine affected how people perceive other vaccines. Like our colleague here said that most of the people are afraid, right?

**00:** Yes.

F: What makes people to have a lot of fears in as far as vaccines for childre are concerned?

P 9: It is true that people are afraid of the vaccines, i heard came across certain women who were disussing that there was someone who was suffering thinking tahta she was suffering from Malarianbutwhne she got to the hospital, she was told by the doctors that she was suffering from Cholera and due to this, she was given Cholera vaccine and when she got home she died. This made peope to be furious because they were thinking that the person was suffering from Malaria while at the hospital she was given treatment for Cholera and this caused her death. I would say that most of the people are afraid that they are going to die if they get vaccinated.

F: What esle?

00: (All) Silent

F: Someone else mentioned about Rota virus vaccine, right?

00: Yes.

I: What do most of the people say about Rota virus vaccine? How do you perceive it or what is our response to this vaccine?

P 3: Let me just comment on what one of us talked, one of our colleagues mentioned about a certain disease that is locally known as 'Nthomba' and this disease makes one to have a lot of sores on the skin that looks like boxes and to me I feel like vaccines are administered to people to prevent such kinds of diseases. I remember this disease was common long time ago when we were young and most of the people now are not aware about this disease and this one of the main reasons why some people are not willing to get their vaccinated. Another this is that most of the people claim that the government is providing different vaccines to the people so that they should die and the population of the country should be reduced. But to me I think there are several ways that the government can use to kill us apart from these vaccines and what is being speculated on the social media that after getting vaccinated, one would have a deformed body is not true but some of the take this as true.

**00:** Laugh.

**P 3:** I got COVID-19 vaccine but up to date am still arrive without any problem.

00: Some of the people said when those who got COVID-19 vaccine hold a mobile phone in their hands, it switched off automatically.

**00:** Some were saying that it is associated with the concept of 666.

**00:** That's not true, I got vaccinated but am not experiencing any problem.

**00:** Maybe a time for you to experience the side effects is yet to come (laugh).

**F**: Do you think something will happen?

**00**: A time will come.

**00:** Some were saying that men who got COVID-19 vaccine will be eating us in our homes.

**F:** They will be eating who?

**00:** Women and children (Laugh).

**F:** Men eating women as a result of getting vaccinated?

**00:** Yes.

**00:** Maybe they will be acting as dogs with labies (laugh).

**00:** COVID-19 vaccine is the one that has made a lot of people not to allow their children to get vaccinated.

**00:** To say the true COVID-19 vaccine destroyed a lot of things.

**P 9:** I remember in 2012 when Rota virus vaccine was being introduced, there were no rumors or anything bad that was being said by the people about this vaccines and people received it very well the same also with Cholera vaccine.

**00:** Even vaccine for measles but with the coming of COVID-19 vaccine, people started having negative attitude towards any vaccine.

**00**: Some people say after 2 years those who got COVID-19 vaccine will start experiencing some of the side effects of the vaccine.

**F:** What do you think should be done for people to be accepting the vaccines? What should be done?

**P 10:** There is a need for the health workers to conduct awareness campaigns aimed at sensitizing people about the vaccines before they are administered to people. There are some people who does research on various vaccines, there is a need for such people to recommend the vaccines before they are administered to people. These can help in removing negative attitudes and perception that people have towards the vaccines.

**F:** What do you think should be done for people in your communities to accept the vaccines easily?

**00:** (All) Silent.

**F:** P 5.

**00:** (All) Silent.

**F:** Our friend has said that there should be some awareness campaigns so that people should be enlighten about the vaccines, what else do you think should be done?

**00:** It is easy for those who know the advantages of the vaccine to allow their children to get vaccinated but for those people who are ignorant they cannot allow their children to get vaccinated. in addition to this, if one really loves his/her children it can be easy for them to allow their children to get vaccinated regardless of what people say about the vaccines.

**P 9:** The bad thing is that each one of us has his/her own views about the vaccines but if we can all have positive attitude towards the vaccines, it would be easy for the people to get vaccinated. To me what is important is for the people to have adequate information about the vaccine and that we should not get carried away by what most of the people say. I believe if people are taught about the advantages of the vaccine, it would be easy for them to accept the vaccines.

**F:** What else, we are approaching the end of our discussion.

**00:** (All) Silent.

**F:** Alright, that's all that I wanted us to discuss today. I would like to thank you for being open and giving us your views, this is very helpful. Once again thank you for giving us your time.

**00:** Thank you.
