# Supplementary material for: Exploration of the social determinants of diarrhoea, rotavirus vaccine uptake, and vaccine ‘fatigue’ in Ethiopia, Kenya, and Malawi
Source: PLoS One. 2025 Sep 9;20(9):e0319691. doi: 10.1371/journal.pone.0319691 (PMC12419581; doi:10.1371/journal.pone.0319691)
Supplement: S1 Data — (ZIP) [file pone.0319691.s001.zip › Supporting Information Files/KY_12FGD.docx]

**FOCUS GROUP DISCUSSION 12**

**07/05/2024**

**NUMBER OF RESPONDENTS-10 MALES**

**1. Can you please tell us some of the illnesses that affect children in your community?**

**R1-**Malaria and diarrhoea.

**R2-**Diarrhoea and a pimple like disease that removes puss sometimes. It is itchy and makes the child cry since it usually appears around the face. Fever is also another illness that affects us though we do not know what causes it.

**R3**-Another challenge that children face currently is when it rains and the rain is collected in puddles, the children see this as a playing area and this might affect their health.

**R4-**Another challenge that children face is that they play in the rain without proper clothing like jackets which ends up causing pneumonia. Playing with water and walking without shoes also causes them to get affected by a disease that affects their toes and causes whitish patches between the toes.

**R5-**There is a disease that is almost like tuberculosis and the child coughs a lot, sometimes measles is also another disease that affects the child if they are not vaccinated.

**2. Which of these illnesses do you consider to be a burden in this community? Why do you say**

**so?**

**R4-**The most burdensome diseases in this community is diarrhoea, fever and malaria. Diarrhoea affects the children because they do not uphold cleanliness when eating and they might pick up any dirty food like mango peeks and just eat them since they are just kids

**R5**-Diarrhoae and fever. At some point you are not able to understand what the child is suffering from because they are just constantly crying and you are unable to understand them.

**R6**-Therea are a lot of mosquitoes in the community which end up biting the children and making them sick. There are also a lot of sewers in the community that are unsanitary and when the children are out playing they may pick things from these sewers and end up eating them because we are not around them to stop them from doing this.

**R7-**You might find that your child is suffering from stomach aches because of playing with dirty rain water that is collected in pools during the rainy season. They later on put their hands in their mouths ir proceed to eat without washing their hands thus causing the stomach aches.

**3. If you were to rank these illnesses in order of priority, what would you rate as the top three**

**diseases affecting children**

**[If diarrhoea is not listed in the top three priority problems -Do you think diarrhoea diseases**

**are a big problem in this community or not? Why do you say so?]**

**R4-**The first one is diarrhoea and the second is fever because this affects most families in the community. The third is lack of appetite and difficulty in feeding.

**R8-**The first is diarrhoea, the second fever and the third is malaria.

**R9**-The first is diarrhoea because this is very common, the second is allergy o cold and the third is headaches and fever.

**R10-**The order of the priority is similar as previously stated by others only that in my opinion there is also cholera.

**R1-**The first is malaria, the second is diarrhoea, the third is stomach aches and the fourth one is measles.

**4. Can you tell me the health services/facilities available in this community? Where do you**

**access health services? [Probe: how much does it cost to access these services, how long do**

**people have to travel to access the services?**

**R1**-I access health services at the sister’s hospital here in Mukuru.

**R4-**I also access the same sister’s hospital.

**R3-**I take my child to Mukuru kwa Njenga here in Ruben.

**R7**-I take my child to Maendeleo Hospital or Ruben.

**R10-**I take my child to Ruben center which is next to a school.

**R5-**There is Ruben center and Maendeleo both which are close to me and easily accessible.

**R4-**I take my child to Maendeleo which is close to me.

**R8-** I take my child to EF, Ruben center and Maendeleo all which are close to me and easily accessible.

**R2**-I take my child to the sister’s hospital here in Njenga.

**R4-**In our community, Maendeleo is the best hospital it has medicines and the service there is quick.

**R10-**Maendeleo operates on all weekdays unlike EF which doesn’t operate on Saturdays.

**5. How do most people respond when a child has diarrhoea in the home? [Probe: What do**

**people do at household level? at community level? Where do they go to access treatment? Do**

**they take antibiotics? Where do they access antibiotics? Why do they access antibiotics?]**

**R2-**I usually boil water and add some salt and give the child.

**R7-**I usually do the same and thereafter take the child to the hospital.

**R4**-The first aid treatment that I usually do at home is boil water and add some salt and then take them to the hospital.

**R5-**I usually do the same thing and when the situation worsens I take the child to the hospital.

**R10**-When the situation does not look that serious, I go to the chemist buy some ORS, boil some water and then mix it with the water and give the baby.

**R8**-I usually boil some water, add some salt and give the baby. When the baby’s condition worsens is when I take them to the hospital.

**6. Can you tell me some of the enablers and challenges that people experience to access**

**treatment for diarrhoea diseases?**

**R5-**In case the child becomes sick at night, it is difficult to take the child to the hospital because f the security around here.

**R4**-Security, financial constraints and long ques at the hospital.

**R7**-Sometimes the doctors at the hospital are rude and difficult to work with.

**7. What do people do to prevent diarrhoea? [At household level, at community level?]**

**8. How do people in this community perceive childhood vaccines [Probe: why do you think**

**childhood vaccines are widely accepted? Why do you think childhood vaccines are widely**

**resisted?**

**R8-**Always washing fruits before giving the child, ensuring that their drinking water is boiled and upholding cleanliness at home.

**R4-**Washing fruits after you buy them and before giving the child to eat them, ensuring that the child does not drink borehole water and instead drinks the tap water and ensuring that the child’s environment is clean both at home and in the plot.

**R5-**Generally upholding cleanliness even though at some point this is hard because of where we live and our environment.

**R6-**Cleanliness is very important. When preparing the child’s food, cleanliness should be observed and ensuring that the food is well cooked so that the germs in the food are killed.

**R3**-The first thing to do is to ensure that cleanliness is observed this includes where they sleep, the food they eat and treating their drinking water.

**9. How about rotavirus vaccines? What do people think about rotavirus vaccines? Where do**

**they access rotavirus vaccine? [Probe: What do they think are the benefits of rotavirus**

**vaccines? What concerns do people have with rotavirus vaccines?**

**R6-**I remember when my child was nine months old they were given a vaccine called rotavirus to protect the child from diarrhoea.

**10. What are the enablers and challenges for people in this community to access rotavirus**

**vaccines? [Prove: cost, distance to access services, cultural/religious beliefs, impact of**

**COVID-19, perception of vaccine safety]**
