# Supplementary material for: Exploration of the social determinants of diarrhoea, rotavirus vaccine uptake, and vaccine ‘fatigue’ in Ethiopia, Kenya, and Malawi
Source: PLoS One. 2025 Sep 9;20(9):e0319691. doi: 10.1371/journal.pone.0319691 (PMC12419581; doi:10.1371/journal.pone.0319691)
Supplement: S1 Data — (ZIP) [file pone.0319691.s001.zip › Supporting Information Files/KY_02FGD.docx]

**FOCUS GROUP DISCUSSION 2. MUKURU KWA REUBEN**

**10 PARTICIPANTS, 5 FEMALE& 5 MALES**

**Moderator: Tell us some of the illnesses that affect children in your community.**

**Respondent 1**: The disease that mostly affects our kids is diarrhoea. We live near drainages, there are plots and open sewers everywhere, all the plots drain dirty water into open sewers. Most of our kid's playing items always fall into the open sewers and they still pick them from the sewer and play with it. Some of them come with sweets, we don’t follow up on their sources, we usually say it is God who protects us. Another thing, where we live there is no proper waste management, so there is rubbish all over. There are improperly disposed off especially used condoms, and some of the kids may want to play with balloons, they end up using the used condoms as balloons, I have personally found several of them using the condoms as balloons.

**Respondent 2**: Another disease mostly affecting children is measles..., measles.

**Respondent 3:** Mostly our kids are affected by headaches and diarrhoea. Their activities mostly involve coming in contact with dirt. Like playing balls, and playing around the sewers. They are involved in many activities that we can’t control as they like moving around even trying to discipline them doesn’t work as they are still just kids and they are forgetful. We have no otherwise as we live here, these are our homes and we can’t move away, and we have them.

**Respondent 4:** I have a kid, 3 and a half years old, the most prevalent problem where we stay, is our kids have marasmus or kwashiorkor caused by inadequately nutritious foods. We usually repetitively feed them rice and this affects their health as we can’t provide proper food mostly due to poverty.

**Respondent 5:** Another disease you find in kids is diarrhoea, you find kids picking things from the ground and starting to eat them and they later experience diarrhoea, that is one of the things that is a problem for kids.

**Respondent 6:** How are you all? I stay in Pipeline, and where am from, obviously diarrhoea and vomiting is the most prevalent problem with kids.

**Respondent 7:** Our kids mostly have cold flu’s because of playing around with cold water.

**Respondent 8:** The diseases mostly affecting our kids are diarrhoea and vomiting. Kids usually eat food that we don’t know where they get it from. When we buy the fruits we usually don’t wash them, especially bananas we don’t wash them, we give them to children without considering if they are clean or not. Others still pick things from the ground and still eat them after they drop them. They start to have diarrhoea and cough a lot. They cough because of the cold as they play with cold water, and they cough and experience running noses. Because kids love to eat, and they eat everything. You give them fruit but you haven’t washed it for them, and right now we have a water shortage everywhere, as they have closed the pipeline water outlet. Right now the water we are getting we are not sure if it is clean or not. If you use the water either I drink or my kid takes it can cause problems. We have stayed without water for 2 weeks. I would like to add and say that our children are suffering because our water pipes pass under toilets and it may be contaminated.

**Respondent 1:** I would like to comment on cold flu. We live in Reuben and Njenga, Cold flu is common where there is a high population of children in one area like the Church. In that church you buy your kid a lollipop and mine doesn’t have one, they will share that lollipop. As the kids share those sweets they get the cold flu.

**Moderator : Tell us the Three priority high-burden diseases in this area**.

R1 For those diseases that we have mentioned, one is diarrhoea, malaria, cold flu, and measles, another is vomiting and kwashiorkor.

**R2.** One of the diseases that are very common and harmful to my children is diarrhoea, cold flu, and abdominal pain. Thank you.

**R3.** Cold flu, diarrhoea and vomiting.

**R4.** Diarrhea, headache and vomiting.

**R5**. At my home the common disease is measles

**R6**. At my homestead, I have two diseases, like my older child is a headache and the smallest is cold flu.

**R7**. We live near a river. So, we get a lot of mosquitoes. Even though they say that there are no mosquitoes in Nairobi, but where I live there are a lot of mosquitoes and it is difficult to get mosquito nets.

**Moderator: Can you tell me the health services /Facilities available in this community?**

**RI.** There are a lot of chemists, there are more chemists than people in this area. As you know, we have one public hospital, Maendeleo, and there is another one, Ruben Center. Here in Maendeleo, I don't know if the government have denied them access to drugs. You get a prescription and you buy from the chemists. Chemists give anything that resembles the prescribed drugs since they want money.

**R2.** Reuben Center, yes, has drugs, but it is expensive. All of us we are not on the same level when it comes to financial status. For me if my child gets sick, I will go to the chemist and tell him, this one has been experiencing diarrhoea for two days or one day. How can you help me? By the way, here in Maendeleo, I am happy that they are now carrying out tests. Even If they dont give you drugs. But now what they need to do is to bring drugs. If you go to the public hospital, you get prescription then they stamp it, you are told to go to town. Town is still money. Thank you.

**R1**. I go to EF. I normally take my child EF in Reuben Center because there is a child doctor who pays for me. It’s expensive to go there but I don’t feel pain now that they pay for me. The other child I take him to a chemist

**R2**. The one I prefer to go to is Ruben or Mukuru. There near the chief. Yes. private hospitals their drugs are expensive. You have to look at your pocket carefully. You know that the work we do is not easy. Today we can it but tomorrow we won't. And if the child gets sick might die in the house, that is the problem.

**R3**. Reuben Center.

**R4**. I prefer the Ruben Center. In fact, even if it's a clinic, even if it's taking weight measurements, I prefer Ruben Center. They have good services and they are more concerned than public hospitals. So I love Ruben Center even if I have to pay, I love it. Thank you.

**R5**. I love Mukuru health center near the chief camp.

**R6**. I would like to say that I was happy that Maendaleo has been opened unfortunately, I have gone there several times but I have never gotten any medicine from there. The medicine you have been prescribed is the same as the one that was banned by the government. I prefer going to Ruben Center and get a prescription because going to Maendeleo is like a waste of time long queue without medication. Reuben Center is far and their prescribed drugs are expensive to buy.

**R7**. For me, a good hospital is Reuben Center. In the other hospital, if you go, the best you can get is these cheap drugs that cost around 10 shillings.

**R8.** My hospital is called Our Lady of Nazareth, but I don't like to go to the hospital. Now, like this morning, he woke up and told me that he had diarhea. Now, what I normally do is I buy a lot of metronidazole drugs. When he tells me that he has diarrhoea, I give him and he goes to school.

**R9.** My hospital is Reuben Center

**Moderator : How do most people respond when a child has diarrhoea in the home**?

1. **At the household level?**

**R1.** I usually get ORS mix it with water and give it to my child. I add this medicine called Zinc. When I see my kid is not getting well, I take him to the hospital.

**R2.** The first thing I did, when he started to Diarrhea, I gave him a glass of warm water and took him to hospital.

**R3.** When my child starts to have diarrhoea I give her boiled rice water when it doesn’t stop, I prepare wheat flour porridge. It helps so much. If it persists in now take her to hospital.

**R4.** When my child has diarrhea I prepare wheat flour porridge or one slice of bread you give when it is dry. To help the kid swallow the bread you can put it slightly into the water to soften a little bit. And when he has constipation you use soap, it helps.

**R5** . For a 6-month-old child with diarrhoea, I just boil water and add some glucose then give to take.

**R6.** We also give yoghurt it helps diarrhoea in both young and older children.

**R7**. For a 6-month-old kid, there is a drug called drip water since the kid has not started to eat. There are those kids who are born prematurely and those who are born maturely. The more you give to the child salty water that you have boiled on the gas. The next day that kid will not be able to respond to long calls because salt affects the digestion of the kid since the kid is used to breastfeeding. Giving salty water to that kid is like introducing illness to that child. The only medicine is drip water and there is a drug called Bonisan its found in hospital. Salty water causes constipation in children. Salt is salt irrespective of the amount; it tastes the same. But generally do not copy anyone in doing this it is only God who protects children.

**What do you do in the community to treat diarrhoea?**

**R1.** Tradition healer knows traditional medication. They normally use soda mint and ashtone to treat teething in children.

**R2.** We also use pawpaw to treat diarrhoea because it's available and its cheap.

**R3.** When it comes to the community, this is where cultural beliefs come in. Different tribes differ like Luhya and Kisii.

**R4.** There are Community Health volunteers, like there is one in our area who takes care of a unit that comprises like 10 women when you have a problem, we tell her that she might have some drugs. You can call her when you are stuck in a log que tell, you get attended to as faster as possible and when there is delays in the hospital she comes and ensures you are attended faster.

**Why do you prefer to go the chemist?**

**R1.** We usually get our medication from the chemist. They have amount proportional to the amount of money we have. Some are in powder form and others in the bottle

**R1.** They understand our specific drugs of interest. In public hospital, you queue and there are no drugs and they will prescribe and sent you the chemist. So, it’s better you just go to the chemist once.

**R2.** I Like going to the chemist since the money I will pay at the reception at Reuben Center is enough to buy my drugs.

**R3.** For me chemists and hospital is just the same. The chemist people cleaned for long time in hospital until they until they knew drugs. But what I can say it important to get tested. It’s expensive yes but at Maendeleo you will be tested free of charge and you buy drugs. The chemist personnel have the history of medication they gave you earlier on and they are flexible to change them.

**R4.** The good thing with chemist is you cannot miss drugs there. But when you to the hospital you be tested and prescribed drugs and go back to the chemist that you left. When you go to the chemist that you are well known to, they will serve you pretty well since they need you again and again.

**R5.** It’s good to go to the chemist but it’s very important for the child be tested and prescribed since the drugs that are prescribed at the hospital are in line with the child’s illness. Because, when you are used to going to the chemist for the child’s cough one day it can backfire. For Maendeleo its good despite long queue and lack of drugs, from there you get exact prescription that will treat your child.
