# Supplementary material for: Exploration of the social determinants of diarrhoea, rotavirus vaccine uptake, and vaccine ‘fatigue’ in Ethiopia, Kenya, and Malawi
Source: PLoS One. 2025 Sep 9;20(9):e0319691. doi: 10.1371/journal.pone.0319691 (PMC12419581; doi:10.1371/journal.pone.0319691)
Supplement: S1 Data — (ZIP) [file pone.0319691.s001.zip › Supporting Information Files/MW_6FGD.docx]

**Facilitator:** thank you for giving us this opportunity for discussion, for you to hear what we are doing and to take part. We would like to discuss several issues, and one of them is about health issues that affect children here in Bangwe. We are going to discuss diarrhoea and what we do when we have diarrhoea, both children and adults. We will discuss about the vaccination and where people go when they get sick, is this clear?

**All:** mmh (yes)

**Facilitator:** we will also discuss diarrhoea prevention if we do it and the treatment, so we will be going here and there but these are the main themes. Let’s start with health issues that affect children here in Bangwe, what are they?

**03:** as you have explained, it’s diarrhoea

**02:** malaria

**00:** high body temperature in children, it’s good for a small child to receive that vaccine to protect a child from polio or stroke, this is a good vaccine

**06:** children are affected by difficulty breathing, if such a vaccine is introduced, it can help children to prevent difficulty breathing

**Facilitator:** mmh, difficult breathing, what disease is that?

**05:** It’s pneumonia, when it’s cold, children have difficulty breathing

**04:** another health problem is a sore throat that comes as a result of flu. If it’s a little child he/she has sores in the stomach

**Facilitator:** we have represented everyone here in Bangwe, so it can be health challenges that affect other children and the ones that affect our children, so look into that as well. Is there any other health condition apart from pneumonia, malaria, diarrhoea, stomach sores, flu and cough?

**05:** there are skin rashes that affect children, especially in their heads, they are common as well

**00:** others call it chicken pox

**05:** no, chicken pox affects the whole body, this one affects the heads only, people then go to the barbershop and shave, and it’s an issue here. Some children’s hair is affected

**04:** there is another one that affects a two-year-old child, they develop sores in the ears, and the ears are damaged. That’s what number 5 said but these ones affect ears, so we don’t know what it is

**Facilitator:** okay, is there anything else to add or these are common ones?

**All:** Silent

**Facilitator:** if there are some, you are going to remember. Amongst the health problems that you have mentioned, what do you think is the biggest health challenge here in Bangwe?

**05:** it’s malaria and diarrhoea because diarrhoea is dangerous to a small child. When it affects a child, we become restless

**Facilitator:** maybe you should explain how dangerous it is to children

**05:** when a child has diarrhoea, he/she is dehydrated, and the time you are taking a child to the hospital, he/she is weak (not clear)

**00:** in addition, diarrhoea puts us at risk because if a child has diarrhoea and is vomiting, when taking that child to the hospital, with our health facilities that have challenges, if we are taking a child to Namatapa clinic, we are referred to Queens and when we go to Queens, we are told there are no drugs, ‘’go buy from the pharmacy’’ and this disease (not clear 5:54) so, facilities like ours should have sufficient treatment for us to be receiving the treatment as fast as possible so that we can prevent such problems

**05:** I would like to add to this issue of diarrhoea. When a child is born and is growing up when he/she has diarrhoea, we think it is teething, and in so doing, we are delaying taking a child to the hospital. The time we take a child to the hospital, we are told that there is no medication, and go to Queens, the time you get to Queens, too late, you have lost a child, yet it was a treatable condition, and you have failed it

**06:** I would like to add, that we all know diarrhoea is very dangerous and everyone is at risk, more especially to small children because their bodies are not mature, their bodies are still fresher than the adults. When such children are affected by this disease, they have diarrhoea and vomit, they lose a lot of water from the body compared with adults and by the time we take the child to the hospital, he/she is weak, and we lose many things. We may give them fluids to improve their conditions but that does not work, we find it problematic, so it’s a dangerous disease to children because they cannot manage on their own. Sometimes parents may be away, leaving children alone, and diarrhoea can start amongst them, fellow children don’t know what to do, so it is difficult, and diarrhoea is an issue here in Bangwe. When talking of malaria, is an issue too because you may find children fainted, and what people do is fan a child or seek some medicine yet they are delaying a child. Instead of taking a child to the hospital to be examined, it is always too late. When they go to the hospital, they are told malaria has rushed to the brain and disrupts a child’s brain in a short period of time, these 2 diseases are risky to the children, and that’s why, when such children recover, their brain doesn’t function well because the time is shorter, at least if it was an adult, it can be okay

**Facilitator:** let’s hear from others

**04:** regarding stomach sores, when the children are affected, there is a problem because once a child is affected, he/she may cry without you knowing the reason why, our health facility here in Bnagwe has no drugs, and they were finished long ago. They just prescribe the treatment for us to buy from the pharmacy, it is expensive such that we cannot afford them. When we came back, we just went to the herbalist, so it was difficult

**Facilitator:** we have talked about health problems that affect children in this area, so we have talked about malaria and diarrhoea, is there another health problem apart from what you have mentioned earlier?

**00:** pneumonia is another big problem here

**0:** pneumonia affects a newborn baby and health workers should help us on that one. When a child is born, they should help too (10:31 not clear) because you find that the child is not well taken care of and the result may be that the child has pneumonia, and unfortunately, the child may die whilst there because doctors at that time were busy with their phones instead of attending the patient, that should be improved as well

**04:** I think health workers should be telling us what to do when we have an emergency considering that there is no medicine at the hospital, so they should just tell us in advance that when there is an emergency, we should be doing what’s right for us not to lose the children

**Facilitator:** diarrhoea, malaria and pneumonia health problems here in Bangwe

**05:** also epilepsy, I have seen several children with this sickness

**Facilitator:** since we are discussing diseases that affect children and we have discussed diseases that we think are more dangerous to children, isn’t it?

**All:** mmh (yes)

**Facilitator:** if we are to select the top 3 diseases, what can be they among what you have said?

**03:** malaria, diarrhoea

**00:** difficulty breathing because a child cannot last for hours

**03:** if a child has any of these diseases for 30 minutes, he/she dies. Let’s start with pneumonia or difficulty breathing, if you cannot rush to the hospital within 15 minutes, the child may die as you think of going to the hospital. Second is diarrhoea, a child dies within 15 minutes because of lack of oxygen and the child is dehydrated, blood circulation is affected too, and the child dies too. Similarly, with malaria, this is what I can explain, within 15 minutes a child may die, but for an adult, 15 minutes can pass, the time he/she goes to the hospital and receives the treatment, something may show up and that’s why we go to the hospital as number 01 said, we may go to the hospital and find staff is busy with phones, it is difficult, they see this patient is seriously sick and tell you too late that there is no medicine, then they give you a referral letter to Queens. When you get to Queens, they tell you there are drugs, then they give a letter for you to go to the pharmacy, and in the process, the patient is dying. Such things are troubling us, we will be worrying because of those civil servants because we think for the lives of children to be taken care of, we should work hand in hand

**Facilitator:** Alright, I would like to know, when someone gets sick here in Bangwe or the areas where you coming from, where does he/she access medication?

**04:** most of the time when someone is sick, for him/her to get quick treatment, he/she has to go to a private clinic and not the public clinic because it does not have medicine, so they just prescribe medication and tell you to go to the pharmacy, it’s better for you to go the private because they assist you fast and medicines are available daily, so when people get sick, they go to private clinics

**02:** some people go to the churches for prayer because they are not assisted when they go to the hospital, so others started trusting their religious beliefs that when we go to a prophet, he/she will pray for the child and will be okay

**01:** I think what number 04 said is true, hospitals delay us because when we go there, they do things totally differently, so, we just take a child for a malaria test. All we want is an examination, and if a child is diagnosed with malaria, we go to a private clinic if we have money, so we go to the private clinic for a child to get better

**Facilitator:** don’t private clinics test?

**01:** they do of course, but it depends on the (16:43) because if you go the hospital, they will still tell you, there is no medicine here, go to Queens, so we think this is near, so they do test but we just try in case we are going to receive the treatment

**05:** when a child gets sick, the problem with the public hospitals is that when you go there, ‘’the child is unwell’’ so they examine a child, then they give you panado and you come back home with panado, you see that the illness continues. Then you think of going to the private clinic because you are not assisted. When you go to the private, they give you a lot of medicine, and when you start administering it to a child, he/she is recovering. So people don’t trust public hospitals fully, they just go there for a child to be examined, that’s the advantage of public hospitals, but for you to receive medicine, there are challenges

**Facilitator:** don’t focus on children only, include adults as well

**05:** Everyone (cross talk)

**04:** when an adult gets sick and goes to the hospital, they just tell you, ‘’We have diagnosed you with malaria, go to the pharmacy and buy LA.’’ You have to go to the pharmacy to buy LA, next time you just think I should not go to the hospital, you just go to the private and if they don’t have testing tools, they refer you to a public facility, ‘’go to a public facility for examination, from there you will come back.’’ There are some private clinics that have adequate resources, you just go there, get treatment and come back

**06:** I would like to add, that usually when you are going to a public hospital, you go with the hope that you will get treatment, but, it’s unfortunate to go there in the morning up until the afternoon and be told there are no medicines. This has happened to me 6 times. I got sick and went there. Medicines were supplied the day before, the following morning I went there, I was told there were no medicines

**Facilitator:** how did you know drugs were supplied yesterday?

**06:** Okay, when the truck carrying the medicines comes, we know it. There are people working there, who tell us there are medicines, so we rush there when we get sick, hoping we will receive enough medicines. When you go there in the morning, you will be told there are no drugs, so you are surprised, ‘’were there many patients last night more than 1000 and they have finished the medicines?’’ it’s questionable, and when they tell us like that, we think that we will stop going to the public facilities, we will go to the private to access treatment. Just like what number 04 said, they also do examinations, for those whose pockets are insufficient, we just go to a public facility for testing and prescription, and then we go to the private clinics. If we have enough, they do everything and we get home better, public facilities are killing us

**Facilitator:** if someone gets sick, but he/she has no money, what happens?

**01:** I would like to comment on the issue of public facilities, if a child has difficulty breathing, and you are not working, for you to buy LA if a child has malaria, it’s K3000. That K3000, yesterday you did not eat from the morning, where are going to get the money? We are giving you this message to pass it to the government. Another thing is that public hospitals should at least be assisting us because not all of us are happy with how this country is governed

**Facilitator:** what happens when we are sick, but have no money?

**01:** we lose lives

**Facilitator:** what do you do to get treatment?

**04:** sometimes we use herbs, based on the beliefs, we use herbs like (21:47 not clear), administered to a child or an adult

**Facilitator:** How long do people travel to get to the clinic? We may think of areas that are far from the clinic

**04:** from Naboya to Bangwe clinic, it’s about 500 kilometres

**Facilitator:** 500 kilometres

**04:** yes

**Facilitator:** not 500

**00:** maybe 4 kilometres

**All:** laughing

**00:** 500 you can get to Mozambique

**Facilitator:** how much money do people use?

00: maybe 1000 to 1, 500 for two people

0: K3000 for two people, which means 6000 go and back, two people on one motorbike, the car cannot reach there (cross-talk)

**Facilitator:** do such areas have private clinics?

00: no

**Facilitator:** how do they get medical treatment if (23:28 not clear)?

00: that’s the way, they travel on foot (cross-talk) even 7000

**Co-Facilitator:** I have people from BCA do come, how much is it from BCA?

All: cross-talk

0: it’s costly as well from BCA using a motorbike, they will tell you 1.5 or 1000 (cross-talk) BCA area has robbers, so people get afraid of going there and cars don’t go there at night (cross-talk). During the day, it’s 500 motorbikes

**Co-Facilitator:** if it’s two people, how much does it cost?

0: 1000

**Facilitator:** apart from herbs, private clinics and prayers, I would like to know if people get biomedical drugs on their own.

**04:** those who work in companies, do have health facilities within so that when they get sick, they access the facilities

**00:** they just refer them to the health facilities

**04:** exactly, but they are referred to private hospitals, such people are better than business people

**Facilitator:** where do people around Bangwe access drugs?

**04:** they buy from the shops, drugs like panado, parapain and penicillin

**Facilitator:** mmh

**00:** just relieve pain as we are waiting to go the hospital the following morning

**Facilitator:** what about drugs for cough and flue

**04:** they buy drugs like cough stop, parapain, panado, Conjex and aspirin

**00:** not aspirin, it only helps (not clear) but brufen

**Facilitator:** do people here use antibiotics?

**00:** anti what?

**Facilitator:** antibiotics, have you ever heard of this?

**00:** no

**0:** they are available in the pharmacies but we don’t know how they are used

**Facilitator:** like what?

**00:** antibiotics are available in the pharmacies but, I don’t how they are used

**Facilitator:** what type of such drugs do people use here, if you remember the names?

**00:** people who use such drugs?

**Facilitator:** not people but names for such antibiotics that you saw people buying from the pharmacies

**00:** what I meant was that I don’t know what antibiotics mean

**Facilitator:** have you ever heard of Green-Green?

**All:** yeah

**0:** drugs to stop diarrhoea

**04:** when you have diarrhoea and take Green-Green, it stops instantly

**Facilitator:** where do you get it?

**04:** we access it from the pharmacies

**0:** this one is available in pharmacies, drugs like Flagyl

**00:** Flagyl is available in the pharmacies too, it is an instant drug, if your child is very sick, just buy Flagyl, diarrhoea stops in less than 30 minutes

**Co-Facilitator:** is Bumulo available here?

**00:** yes it’s available here,

**Co-Facilitator:** I heard it from one of the villages

**04:** they are blue capsules (cross-talk)

**0:** That’s Green-Green, some call it Bumulo, and some Green-Green (cross-talk)

**00:** Green-Green is blue one, Bumulo is like Brono, I once bought it (cross-talk) and people love it too much

**Facilitator:** Alright, we have discussed that when a child has diarrhoea, he/she is taken to the hospital, but what do you do at home when a child has diarrhoea?

**04:** when a child has diarrhoea, we rush to buy Thanzi, some parents give such child Thobwa, it stops diarrhoea, and that’s what happens

**Facilitator:** please continue, I interrupted you

**04:** as I have said, when a child has diarrhoea, we rush to the pharmacy to buy Thanzi or Thobwa, it helps stop diarrhoea

**01:** when the child has diarrhoea, we do home remedies like mixing salt and sugar, and homemade Thanzi. When taking a child to the hospital, he/she is given Thanzi

**02:** healthcare workers say when a child or an adult has diarrhoea, should be given water every now and then

**Facilitator:** you all have under-5 children and at one point, that child had diarrhoea. I would love to hear from what you did at home before you took a child to the hospital

**04:** when my child had diarrhoea for more than two days, I was giving him/her Thobwa. I was advised by a doctor that just give him/her Thobwa every now and then, diarrhoea stopped

**Facilitator:** For how long did it take for diarrhoea to stop?

**04:** aaah, the whole day

**Facilitator:** did diarrhoea completely stop after taking Thobwa?

**04:** After taking Thobwa, the diarrhoea continues, but here and then. I gave him/her Thobwa in the morning, he/she had diarrhoea at around 3. I gave him/her another one until evening, he never stopped up until morning when the child was okay

**Facilitator:** what did you mix Thobwa with?

**04:** nothing, it was just Thobwa

**Facilitator:** even Sugar

**04:** sugar was there of course

**All:** laughing

**06:** I remember when my child had diarrhoea, what I did was to mix sugar, salt and water, just like what number 1 said. Then I administered it to the child, and within 30 minutes the child recovered completely

**03:** when my child had diarrhoea, I gave him/her ashes, the condition improved but not completely

**Facilitator:** how old is a child?

**03:** 3 years old

**Facilitator:** did you (add water) and just stir it?

**03:** I just (added water) and stirred it, we learned home remedies from our church

**Facilitator:** Brother, you have found us discussing diarrhoea and we have started a bit earlier, but you are not very late, if you have an opinion, you come in, but we have numbers, so yours is 7. When you want to speak, mention your number before expressing your opinion

**07:** mmh (okay)

**Facilitator:** so, feel free

**0:** my point is what this one said about mixing salt and sugar to make Thanzi ORS

**Facilitator:** is there something different from what others have said?

**00:** I think there is none, these are what we do

**Facilitator:** Alright, what do you think causes diarrhoea?

**01:** it’s caused by the food that our bodies don’t accept like unclean foods, when we eat such foods, they affect our lives, even mangos, I remember sometimes back in Ndirande, people had diarrhoea, during cholera time, it was popular then because of unclean water, so it depends on water and the food that we are eating. We should make sure the food that we are eating is clean or the water sources that we use

**Facilitator:** we have said diarrhoea is one of the health concerns here, in addition, what do you think is happening with water for this area to have this problem of diarrhoea?

**06:** in other words, we rivers that people wash from, and some people urinate in such rivers. After that, there are microorganisms, when someone contacts them, they cause problems in his/her body, and after that, the person has diarrhoea. Some defecate in the river and someone drinks water from the same river, that person contacts the germs that affect him/her. Children may play with such water and drink without their parents’ knowledge, and that child may drink together with germs, so we can say many people here have no toilets, they just defecate anywhere, and then houseflies transmit germs to the foods, and someone eats food without washing hands, adding germs in the body and that’s what causes this problem in this area. We have a city in Ntopwa if you pass by there, you will be disappointed because of the environment, just across this bridge, there is a stream where people dispose of their waste, there is a very bad smell, and the waters are black. Dirty water from that stream is passing through your house, you are eating nsima, and there is a very bad smell, and there can be something bad contacting your food and you see no problem with that. After two to three days, they start affecting you. Most people often get sick from it, and diarrhoea is very common in there

**Facilitator:** Ntopwa

**06:** yes, even water from bathrooms passes through your house, and children play with them, then they eat food without washing their hands.

**04:** just to add what number 6 said, because of water problems here in Bangwe, our wives draw water for baths from the rivers, and we get germs from bathing waters because people dump diapers in the rivers, so they contaminate the same river that they use for bathing, this is a very big problem

**Facilitator:** people here bath water from the river

**04:** yes, when there is a water problem that week, we use water from the river for bathing

**01:** to be honest, based on our community here in Bangwe, we cannot buy water just for washing, in short, in October, we use water from the river, and rivers like Namatapa started up there, there are sewage systems from Mpingwe, that’s what we use, it looks clean but it’s dirty because people dump wastes, so don’t protect ourselves due to the setup of this area

**Co-Facilitator:** what can you say about piped water?

**01:** to be honest, if you travel from Ntopwa to here, you will only find a few tap water, where there are MASAF, areas like here in Namatapa we use a borehole which is at a Lutheran church, others use water from the wells, others wise clean water is available in the fenced houses and it’s for themselves, but that borehole at the church, even people from the clinic use water from the borehole. It is difficult, therefore, to treat that water because it’s coming from beneath, all waste deposits go there as well, so we don’t use piped water

**04:** in other words, we have 3 or 4 boreholes here

**Facilitator:** the whole Bangwe?

**04:** around this area, Namatapa and Ntopwa, we have two boreholes in ETG but another one is at a mosque, and another one after that. We have this side a borehole which is at the church, which means 3 boreholes in total, another one at school, 4 in total

**00:** when piped water stops, those from hilly areas come down to access water, so we cannot say we will use the water for bathing, we just say Aah I will use water from the river for bathing, this one should be used for drinking

**Facilitator:** if you estimate the number of people who use these boreholes, how many are there?

**00:** it’s many because we can have water for 2 days and 5 days without water, so people depend on that water from the boreholes

**0:** even one week

**Co-Facilitator:** estimate the number of people

**Facilitator:** just an estimation

**0:**  maybe 3. Something

**Facilitator:** million?

**0:** thousand

**All:** both laughing

**00:** they cannot reach that number (cross-talk) Ntopwa uses Chiwe and water from the mosque, they don’t use any other borehole

**Facilitator:** if those 4 boreholes were one, how many people use that borehole?

**00:** There are many because it’s this whole area

**Facilitator:** how many people approximately?

**0:** maybe 4000 (cross-talk)

**04:** it’s more than that because that clinic uses it (cross-talk) including those living in the fenced house

**Facilitator:** you said this area has an erratic water supply, many times per week did you say?

**04:** 2 days or one day (cross-talk) after 2 days, it will disappear

**0:** just one hour or 30 minutes, they stop. Sometimes they start very early in the morning, that’s all

**Facilitator:** next will be when?

**All:** cross-talk

**04:** This area has a water problem, and that’s why diarrhoea is common here than in other areas because it doesn’t matter if you live in a fenced house, everyone goes out to fetch water from the river and after there, we don’t even boil the water, we bath as it is, together with the germs and in two days, you start getting sick, that’s what happens here

**Facilitator:** we have talked about what causes diarrhoea, hygiene, water problems and toilets. How do you prevent diarrhoea?

**01:** according to our financial status, we cannot just apply water overnight, that’s not possible. We just accept that if it happens, we will receive it because we are suffering

**Facilitator:** mmh

**01:** if there can be donors who can help us with boreholes or water from the water board, at least we can prevent, on our own it’s difficult

**04:** there are preventable problems and there are un prevented problems such as hygiene issues, it is difficult for us because water is a problem here and the water board one only available in the very early morning, and if you delay, you will not find it, you will draw from the borehole and maybe two pails only depending on the distance, most of people depend on the water from the rivers, bathing, maybe he has 5 children, all of them will use the same water from the river

**Co-Facilitator:** you draw water from the river or borehole and you already know they have germs, what do you do on your own knowing that the water has germs?

**0:** we boil them before using it

**05:** it is not possible to boil water because it needs charcoal

**All:** laughing

**04:** (cross-talk) We just accept it because most people use water from the rivers and if you observe, people dig wells along the river, for them to have water to drink

**Facilitator:** what have they done?

**04:** people dig wells along the rivers, and water comes from beneath, so they drink such water (laughing)

**02:** people dig wells behind the rivers, do you think that is good water, do we know what happens in these wells?

**01:** we have 4 boreholes, but our friends from Mondiwa, and Masala, use water from the wells only, and you cannot find boreholes anyhow. Water board and a few water sources that are functional, and such people are at risk, I know one day you will meet them as well, and they will tell you what I am saying. At least we have boreholes here but down there, I once lived there

**00:** Mondiwa is worse, it’s down there, a very remote and busy area

**01:** even when you want to buy relish, you walk a very long distance, it’s hard to reach the area, and you cannot find piped water, this area is better than theirs because in that area, you can only find water from the wells only

**02:** in the recent past, they were distributing chlorine, but because cholera cases were reduced, they stopped distributing it, so we were using that chlorine

**00:** when they were distributing chlorine, it was helping us prevent cholera, when they stopped, this area was at risk

**Facilitator:** how were they distributing it?

**00:** they were going to the communities

**Facilitator:** in communities

**00:** yes, like Namatapa, they could just announce, ‘’Come to Namatapa school tomorrow for chlorine!!’’ then we could just send the children, ‘’Go receive chlorine.’’

**Co-Facilitator:** which means every household had chlorine

**All:** yes

**00:** we were receiving like that size, then we could use over a month or two, but they stopped because cholera cases had reduced

**Facilitator:** we have talked of diarrhoea, let’s now discuss vaccination because we are going towards the end of our discussion. What do people say about the under-5 vaccines?

**04:** most of the time, depending on how famous is that vaccine, people think it makes children barren. Based on where this world is going when healthcare workers go out for under-5 vaccination campaigns, even mine, I refused

**01:** I just want to comment on what he has said. Previously, we were receiving the vaccine from schools, but according to this world today, if they say we are going to vaccinate children tomorrow, some people tell their children not to go to school, ‘’I don’t want my child to receive the vaccine’’ Vaccines protect children, but because someone damaged it, people think vaccines are bad, but some of them are beneficial to children in future

**06:** the coming in of COVID vaccines has disrupted peoples’ lives here in Bangwe, they think every vaccine is for COVID because they think when COVID minus 19 comes, children are destroyed, because of that, most people refuse their children's vaccinations, they think that we had no such things all along, we had no COVID-19, what is this vaccine for, that’s what happens. People are not receiving the vaccines now because of the COVID-19 vaccine, it has discouraged people so that they think, ‘’Let children be, God will be taking care of them.’’

**05:** how the vaccines are introduced in this country, our parents were telling us how previously vaccination issues used to be, it was only once

**00:** measles vaccine

**05:** it was only once, it’s surprising now, today you receive the vaccine, tomorrow another one, up to 5 times, people are afraid. I received the vaccine once, so it’s surprising that they are coming, ‘’eeh receive the top one’’ Such things make parents tell their children not to receive the vaccine

**Facilitator:** do you have anything to say, we are discussing what people say about the under -5 vaccines, your number is 8, when you are speaking, mention that number

**08:** I have a comment on what the brother has said. It is surprising because they are giving out 7000. So they are giving out cash, which used to be free and once, so they are leaving their offices and come here to vaccinate children and give them 7000, bathing soap and pail, so you wonder, what’s going on. They came to my house and I found them, I told them’’ I don’t take part in these childhood things, please go back.’’ So, tell us what is going on

**Facilitator:** can we answer that question at the end, we would like to hear what people think about the vaccines, we are thankful for your opinion

**08:** Okay

**Facilitator:** I would like to know, you said that you refused your child to receive the vaccine as well, what was that vaccine?

**04:** it was polio vaccine. They brought the first one, the oral vaccine, so they put some drops in his/her mouth, when a child was born, he/she received the polio vaccine as well. After some months, he/she received the polio vaccine, the past 3 months, nurses came with the same polio vaccine and this time around, they came with the fourth one and I said Aah, another jab, it’s enough for polio vaccine, I told them you should not vaccinate my child because he/she should not be receiving polio vaccines until when

**Facilitator:** what about you, can you remember the vaccine?

**08:** it was the polio vaccine, after sending them back, the child received the vaccine at the health facility

**Facilitator:** after sending the back or not?

**08:** when they got me home, told them and after two weeks, the child got sick and when he/she was taken to the hospital, they were asked, ‘’Did the child receive the vaccine?’’ they said no, so they were sent back. We then received the vaccine because we wanted to access the treatment but for me, I was not happy

**Facilitator:** what prompted you to receive the vaccine?

**08:** when I was young

**Facilitator:** no, for your child to receive the vaccine, for you to be satisfied, was there anything?

**08:** for me, I was thinking, all these vaccines in one month, aah according to my studies, maybe they want to make children barren, that’s what I was thinking

**All:** laughing

**08:** they want to make children barren because this is not the first vaccine, during the days of King Herod, he was killing male children that time, and Pharaoh was killing male children to protect his throne, he was using the vaccine, and this is not strange for us who read books, and we follow Quran and the Bible very well, and we say ‘’ooh what is coming now, happened before, they want to reduce black peoples’ population, so they should remain only the white people,’’ There is another emerging issue of men marrying fellow men, and that’s why I said aah my child should not receive the vaccine

**Facilitator:** (laughing) Let’s now hear from others, what do you think about the coming of more vaccines, he has spoken his views regarding what he thinks, let's hear from others

**07:** I can comment on what this gentleman has said. My thoughts are similar to his, they want children not to overpopulate in future, and that’s why they are introducing the vaccines so that black people should go, they should remain the white people only so that what they want to do here in Africa, they should do it freely

**Co-Facilitator:** we are going to talk about it at the end

**Facilitator:** mmh, let’s hear from others

**All:** Silent

**Facilitator:** you have said this has been happening since the introduction of the COVID-19 vaccine, what did people say regarding the under-5 vaccines previously?

**04:** there had been vaccines before and our parents were not refusing us to receive the vaccines. There was a vaccine for bilharzia that was supplied in schools. They were pills, not vaccines. So there was the measles vaccine, there was the polio vaccine which I never received, but the measles one, I received when I was in standard 5. During those days, parents had no concerns. From that time until this time of Chakwera, it’s difficult for someone to allow their children to receive the vaccine because of the issue of overpopulation. People think if a child receives more vaccines, he/she will die, will be barren or will be demonic. Some children are not obedient, maybe it’s because of the vaccines

**All:** laughing

**Facilitator:** some children are not obedient

**04:** yes

**Facilitator:** what obedience?

**04:** underrating parents (cross-talk)

**00:** when a 5-year child is wrong and you advise them, they tell you, ‘’Your age mates had gone

**Facilitator:** 5 years

**00:** 5 years and you ask yourself, why is it like this?

**Facilitator:** ‘’is it because of the vaccine?’’ laughing

**0:** I have a child who is less than 2 years old. He tells me, ‘’You are rubbish’’ when I go home empty-handed without Kamba puffs, he/she asks, ‘’Where is Kamba?’’ ‘’I haven’t bought one’’ ‘’ you are rubbish!’’

**All:** laughing

**0:** where is he/she getting all these?

**04:** it’s because of the vaccines

**0:** some vaccines are suspicious, they are demons because….

**04:** (previously, polio vaccines were received once at the hospital only, but this year, the same polio vaccine is received up to 8 times by the same children, and not others, it’s surprising. The children start receiving the polio vaccine right from the hospital, then the second one is received from their homes, the third one, the fourth one and the fifth one. If it were you, what would you think?

**Facilitator:** we have talked of concerns that parents have, besides all these, what motivates parents to have their children vaccinated?

**04:** what motivates children to receive the vaccine is that when a child gets sick, it worries parents because vaccines help children to prevent diseases, and children grow well because they don’t get sick every now and then, but that depends on the type of vaccine

**05:** what happens to parents is that when a child is born, we know all vaccines that should be received by a child, so parents follow the vaccine schedules

**Facilitator:** why previously were you taking children for vaccination?

**05:** to prevent diseases, that’s what we were following, and not what’s happening now, children are receiving the vaccines up to 10 times, which discourages us

**0:** after all, there was only one vaccine at that time

**Facilitator:** apart from too many vaccines, are there other reasons that prevent people from receiving the vaccines?

**02:** religious beliefs, some people don’t believe in going to the hospital to receive medication

**05:** some people don’t believe in biomedical drugs, they believe in herbs, they don’t pray, but consulting traditional doctors

**04:** there is a religious group called Jehovah’s Witnesss, they don’t believe hospitals have answers for their illnesses, they believe in God so much. A child is born up to growing up without going to the hospital

**08:** similar to the apostolic people, their things work better than us who receive the vaccines, we get sick often, so we are surprised. Apostolic people don’t go to any hospital or traditional doctors, they just stay, growing up the same how chicken does, but they don’t get sick. For us who receive the vaccine do sick or what number 7 said, maybe white people have plans for the future, for example of a museum, you can raise chickens, you want them to multiply, then you kill them. Do the white people take us like that? God created us, but white people have more wisdom such that they test their weapons on us, we don’t know. We used to go to the hospital but today they come to our homes, what’s the problem, maybe there is something that they want to get in future

**06:** another risk that makes people have doubts about vaccines is that healthcare workers were forced to receive the vaccine, otherwise they were not allowed to work. Malawians are now civilized, they say healthcare workers are working and there are many vaccines been introduced, what are the benefits of this vaccine? I we study it deeply, as number 8 said, they want to reduce our population because we are overpopulated. In the near future, people will not have children because there are more vaccines in their bodies and these vaccines are not futile, they have benefits. You can believe me, if you have eaten more food in a day, you may be constipated or discharge undigested food at the toilet because the food is too much. As with the vaccines, this can happen in future. People get encouraged of course but some have discovered that the vaccines are too much, they are still coming with the same people, repeating the same vaccines, If the vaccines had been given to other people, and not the same people. Some people are afraid and they say let it be since it has already happened, some say, when the vaccine team comes, they chase it away, ‘’We don’t want you here.’’ When some people go to the hospital, they are told not to access services, because of fears, they receive vaccines which bring problems to their health, people have no peace, they are worrying and such problems and people think these are the last days, obviously this is heading to something. If these people continue with these, this is their aim, and that’s why some people discovered that white people have their plans, they can have the plans of course, but people have discovered it quickly

**Co-Facilitator:** what do you think the government should do for more people to participate when the new vaccine is introduced? What can be done?

**01:** it should not be a must, it should be on a voluntary basis. The risk is that when our wives go to the hospital, they are asked, ‘’Did you receive the polio vaccine?’’ ‘’no’’ ‘’for a child to receive treatment, he/she has to receive polio vaccine.’’ Such things put us at risk, ‘’why are you forcing it?’’ If a child dies, it will be by God’s grace, but we should not be forced on it, you should just feel it, ‘’vaccinate my child’’ we should not be forced

**02:** they should train us before the vaccine, they should tell us the advantages and disadvantages of that vaccine so that when people speak about the disadvantages of the vaccine, we should already know them, and then we can decide to go and receive it

**07:** as I am speaking now, most women have received cholera or polio vaccine out of their will because they had gone to the under-5 clinics with children, so they were told, ‘’if you don’t receive the vaccine, we will not assist this child’’ even if you has just send a girl to go to the under-5 clinic with your child, she was told right there, ‘’if you did not receive the vaccine, I will vaccinate you, if not, this child will not be assisted.‘’ if the child gets sick, and you take him/her to the hospital, the child was not assisted, so people started accepting the vaccine so that their children should access the health services

**08:** the vaccines women are receiving nowadays, are not as sweet in bed as they used to be

**All:** laughing (laughing)

**0:** in the near future women will be forced to receive family planning injections, ‘’were you injected?’’ ‘’no’’ ‘’you are going to receive one’’ (cross talk)

**Co-Facilitator:** you have said children are receiving the vaccines when they go to the hospital, even if you send someone

**0:** exactly, they are just asked, ‘’Did you receive the polio or cholera vaccine?’’ ‘’I did not receive it’’ ‘’for the child to access medical services, you should receive these vaccines.’’ So, if you hesitate to receive the vaccine, you face it when you go to the hospital and you cannot run away

**Co-Facilitator:** when they are giving the vaccine, do they tell people the advantages or disadvantages of the vaccines?

**0:** women are told the advantages and disadvantages and we know them, but because of the bad approach, we think women are not true to us

**06:** according to the situation we are in, things are bad. Even if they come with the truth, nobody will believe in them because if we can look at the time we are in, according to the scriptures, things are going toward the end because it is written somewhere that ‘’a time will come, young and old, poor or free will be forced to receive a mark on their hands or forehead, and if anyone will not receive, he/she will not be allowed in hospitals, buy or selling anything in the markets.’’ If we look at the situations that are happening, if someone is going to the hospital and is told they are not going to be assisted because they did not receive this, it means there is something that will be happening and is creeping. Our eyes are seeing but another spirit tells us, ‘’What is happening now is such a thing, so we are having a revelation in our lives. As number 2 said, if they could teach us first, maybe people would have been flexible because once you have taught someone, he/she has a choice to make, just like what you have said, you said we can choose to take part in research or not, but you have sensitized us, then it will be up to a person to decide whether to proceed or not, it’s not a must. Similar to this situation, if they came with sensitization to people, whether it’s not true, people may decide what to do, but because it is a must, most people are discouraged. Even if they come today, people will just listen to them but will have battles in their hearts. If you ask anyone, will tell you, what this one is teaching us, I wish I would have beaten him/her up, but because he/she is teaching, let him/her finish, afterwards, when getting out of that gate, ‘’these stupid people from government come all the way to teach us, they think we don’t know.’’ As we are here, people can stay quiet listening and talk as if all is okay, once they go out, what they will talk out there, you may be disappointed, that’s honest. People are tired when they see the situation in the world, they say ‘’eeh gentlemen, these people should not bother us.’’ So, everything you tell them will be regarded as bad even if it’s good, that’s why people today have no hope, they have fears because of such things. If they tell us this from the hospital, expect to be told that you are not allowed here at the market, those who have a mark. Let’s not beat about the bush, they are setting up a foundation so that these children who are vaccinated, will be free tomorrow, for we are done, we will be persecuted, but children will be free because, through these vaccines, they are benefiting something, these children are in their systems and these systems will make them honourable and we will be like the wastes

**Facilitator:** thank you very much. Let’s discuss the Rotavirus vaccine. Who heard of this vaccine?

**00:** I heard of it

**Facilitator:** who else? Or the vaccine that protects against diarrhoea

**08:** I heard it

**Facilitator:** what did you hear about this vaccine?

**08:** they say this vaccine protects from the microorganisms so that if a child is affected, microorganisms should be weaker so that when you go to the hospital and receive the treatment, these microorganisms should already be weak and you should not take longer if you have diarrhoea. That’s what they explained. You should take longer if you have diarrhoea, so if you have received the vaccine, and there is medication in your body, that microorganism is weaker, so you just go to the clinic for additional help

**Facilitator:** mmh, what do people say about COVID vaccine? They are not called by this name, we have talked about the polio vaccine

**08:** when people are coming, they come with good news and there is no one who can say this is a bad vaccine, they are like business people. If you ask them, ‘’What this kanyenya is made from?’’ they will never tell you it’s from pork, they will say it’s from chicken because everyone likes chicken, so they cannot say this vaccine will damage your bodies, that’s not possible. They will say this is a good vaccine, it helps a lot, and they say things that will make you fall in love with that, but they cannot say this vaccine is damaged

**Facilitator:** that’s what they say

**08:** yes

**Facilitator:** what do you see regarding this vaccine?

**08:** we cannot say we are seeing anything now, but maybe in future we will see. If you receive the vaccine today, it cannot show in three months. They give you many years, we even watch these things from Indian movies, they are educative, so we just don’t watch them. Once you receive the vaccine today, it will take 8 years for it to start showing, and I believe these children will be barren in future

**Facilitator:** Okay, they are saying that this vaccine is protecting from diarrhoea, so when the children have received the vaccine, what do you observe regarding diarrhoea?

**01:** he has already said that at that time, it is okay, but these things have a specific period of 8 years or 10 years before they start showing up. At the time of receiving it, it can be good

**Facilitator:** what current advantages do you see with this Rotavirus vaccine?

**01:** (not clear) But I don’t know what will happen in future

**Facilitator:** I wanted to know if this vaccine has any advantage regarding diarrhoea, is it reduced or not?

**0:** I would like to ask (1:17:20 nor clear)

**Facilitator:** yes, you want to tell your wife not to go?

**0:** no

**All:** laughing

**08:** if we say this vaccine has a problem now, that’s not true, but our eyes are in future because an adult like me to receive a vaccine me to be barren, or to kill something in my body, it cannot work because my body is well mature. For children, it can be possible because they have started from the underground. They say up to 5 years, they give an overage of 10 years, when a child reaches 10, he/she has something wrong. If they vaccinate children of this age, they are already civilized, ‘’let’s go to Kachere,’’ they see that their penis doesn’t erect the way they supposed to be, ‘’can I see it, it does not erect.’’ They think it’s another problem yet it is an effect of the vaccine that he received, we have these thoughts for the future, and for us vaccine cannot make us barren, our problem is that when we are in bed, we just do it once, then we sleep with our hand touching it (vegina) it is weakening us

**Facilitator:** how many times were you doing it before?

**08:** iih! We do it my brother

**All:** laughing

**Facilitator:** that’s good news, we have reached the end of our discussion, but before finishing up, there might be something that we did not ask, and it’s your time to raise it, everyone who has it can start

**00:** let me ask, what benefits do you get when you are sent to have a discussion with people like us, considering that you reimburse our transport money?

**Facilitator:** that’s a very good question. When a government would like to come up with a policy, there should be evidence. It is different from what was happening previously whereby ministers would just discuss, ‘’This is how healthy issues will be done, agriculture will be done this way, ‘’ then they could tell people, from today on, this is what will be happening. It was then discovered that such policies don’t work because people don’t receive them, they infringe others' rights, or such policies are not in line with people’s wishes. To ensure policies are benefiting people, they go to the beneficiaries and hear their views. ‘’we would like to this and that, what do you think?’’ the advantage of this Rotavirus vaccine is that when the government gives out this vaccine, maybe people don’t receive it. When we know the reason why people don’t receive it, it will help improve the approaches. There was an issue of lack of awareness, which will help the government to reach out to people. Government cannot know if it does not approach people to hear their thoughts, so this is helping, it’s like a doctor and a patient are discussing, ‘’What should we do with your problem of headache?’’ patient is giving out his/her reasons, ‘’for me, do this and that for the drugs to be effective’’ doctor should also explain from his/her side, ‘’if we do this way, this will happen.’’ So, the two will reach a consensus and come up with something beneficial to both sides. The advantages of this discussion will help during policy formulation

**02:** just an opinion, if the government had given a chance to the traditional birth attendants for them to work freely like before, the issue of vaccine would have not been a problem, but because traditional birth attendants don’t work here in Malawi, people only access health services from the hospitals only and I think there are rumours to with hospitals, we have nowhere to access health services, so with all these going on, like bareness as a result of vaccination, if it were like those days, people could go to the traditional birth attendants and after that, they could go to the hospital for vaccination because they had the option to choose where they want to deliver their babies. So, there should be many healthcare workers in the communities

**Facilitator:** Okay, people should have the right

**08:** commenting on what number 2 said. Government is clever, previously people were going to the traditional birth attendants, and then traditional birth attendants were banned. ‘’if anyone goes to a traditional birth attendant, that birth attendant will be prosecuted.’’ So all the people go to the hospital. They knew if we gave the right to the traditional birth attendant, people would no longer go to the hospitals, so they locked up that one first so that people should be going to the hospitals. These are their future plans

**Facilitator:** if these vaccines are offered by the traditional birth attendants, will people receive them?

**08:** that will depend on how sensitized people will be because what happens is that there might be something that the hospital gets from the woman who has given birth, and we know it, but we cannot stand here and explain. So when they see that traditional birth attendants are benefiting, let’s lock him/her up and everything should be done at the hospital, so they benefit. When a woman has miscarried, healthcare workers ask, ‘’Are you going to dump it yourself or we should do, they want to see if you are civilized or not, but for men like us, the Adams, we know this is deliberately, they want to pack it in a carton and send it to China, this is a business (not clear) so they decided to lock up traditional birth attendants, everyone should go to the hospital. If you have injured your fingernail, you are told to receive the vaccine, get injected and the like, how is this possible

**Facilitator:** Okay, any other words, we have discussed it enough. There are church people coming

**All:** Silent

**Facilitator:** allow me to take the photos

**All:** mmh (yes)

**End of interview**
