# Supplementary material for: Exploration of the social determinants of diarrhoea, rotavirus vaccine uptake, and vaccine ‘fatigue’ in Ethiopia, Kenya, and Malawi
Source: PLoS One. 2025 Sep 9;20(9):e0319691. doi: 10.1371/journal.pone.0319691 (PMC12419581; doi:10.1371/journal.pone.0319691)
Supplement: S1 Data — (ZIP) [file pone.0319691.s001.zip › Supporting Information Files/ET_3FGD.docx]

I: Thank you for your participation. What are the common diseases affecting infants in your village?

P1: I have children below the age of five. My children and other infants in this village are usually affected by bacterial diseases, fever, and vomiting.

I: Okay, how about you?

P5: Tonsillitis, common cold and rash are common diseases here. Rash on different parts of the body has also been a common illness among the children.

I: How about the other of you? What are the common diseases affecting infants in your village?

P2: I had to visit the health center recently. I have observed that many children have been affected by fever, and it is like pandemic. The symptoms of this flu are fever and vomit. I learned how fast the transmission has been from the doctor who had been giving me treatment and medicine, as I was also affected by that flu.

P2: There are a lot of dirty ditches in our village. These ditches can cause diseases in children and adults. They usually cough, affected with fever, vomiting and acne. The ditches have become a major cause of diseases affecting children and even adults.

I: So, what are the diseases caused by this unhygienic usage of ditches?

P2: Flu.

I: Okay. What else? What are the common diseases affecting children in this village?

P1: I want to add some; the children in our village live in a very compact way that made them vulnerable to transmission of disease. I usually found out that the children are having a fever when I check their head by touching. Rash, vomiting, severe cough, and loss of energy are what I observe on them.

I: Is there any different idea on this matter?

P3: There are many unhygienic ditches in the village. My child checks up every month or twice; he is Autistic and also affected with heart attack. The absence of hygiene in this area is making his conditions worse. He has come here in Tikur Ambessa five times. The ditches are very unpleasant and so the toilets. Some people use the ditches as a toilet; living in such conditions is not good at all.

I: You have mentioned that the children have been affected by bacterial disease, right?

*[All of them]: Nodded to show agreement.*

I: What are the symptoms of the bacterial diseases you have mentioned?

P3: Their faces become jelly, stomachache, fever, and sometimes there can be acne, loss of appetite. Somehow, it is similar to the flu.

P10: My child is a year and three months old. He is often affected with bacterial diseases and he usually loses appetite and hates to breast it. The symptoms are loss of appetite to cry now and then diarrhea and vomit.

I: Very good. You have mentioned many like diarrhea, vomiting, fever, rash and coughing. Which one of this is more common among the children?

P1: It is the fever.

I: Fever is a symptom, which disease has been the cause for that?

P4: It is because of the unpleasant smell from the ditches and the flu caused by that. There happen a high fever on them when they come home, after staying to those ditches.

I: What other disease are the most causes of the symptoms you have mentioned?

P6: Children pass their time together and they interact more closely. This made the transmission of the disease easy. I prohibited other children not to come in to home. It is not that I hate children, I have done that to block the transmission and the vulnerability of being affected with flu. It is for the sake of the health of the adults and children. I have told my wife not to let any child to enter to the house.

I: So, what has been the most common disease?

P3: It is the flu

I: How about you, the other of you?

P4: Most of the time, it is the flu. There are also vomiting and diarrhea.

I: Which diseases has been more dangerous for the infants living in your village?

P3: Most of the time, both flu and bacterial diseases are caused by absence of hygiene and bad system of sanitary. There is a system of disposing sewage but it is implemented in a way that let the people affected with spilled dirt here and there, there should be well confined black plastic bags.

I: Before discussing about the solutions, could you tell me the most dangerous disease that has been affecting infants in this village?

P3: It is flu, I mean it is the bacterial disease. The flu is the second most dangerous. It is because the bad smell coming out while disposing the sewage, our children smell that when we brought them from schools and that is one way of being affected by the flu.

I: Can you rank the danger of those diseases?

P3: Bacterial disease are the most dangerous of all, Flu is second. The flu has been affecting not only the children, but also the adults. There is high transmission of flu now, there are only some who has not been affected.

I: Okay. You may also need to consider the situation in general, not only the current situation. Can you rank the danger of these diseases in order?

P10: I think bacterial disease is more dangerous. We can treat the flu by breastfeeding and eating oranges that doesn’t work for bacterial diseases. We must get the medicine for bacterial disease. So, the bacterial disease, and the flu, respectively. Tonsillitis is the third most dangerous disease; it is caused by a bad smell that irritates the tonsils.

I: Any other?

P6: It is like they said. But, the diarrhea caused by the bacteria made them lose energy and it is incurable. There is a cultural problem; we live in a society living densely. I live in a single compound consisting of many households, there are children between the ages of five and six, and also with the age of just a year like mine. The immunity of those older children is higher, so I need to protect my child and quarantine him at home. People in Our compound don’t understand this and think you do that because of hatred. There is a child that is often affected by tonsillitis; my child is usually get affected by tonsillitis transmitted from this child. My child stays sick for fifteen and twenty days, sometimes even long after the other child gets better. My child don’t get better just after he takes the medicine, as he is very young to gurgle the solution of salt, he swallows it, so it can’t cure him. Taking too many drugs is also not good for children, which may cause diarrhea.

I: Is there a different disease you want to rank as dangerous?

*All participants: [No answer]*

I: If there is none, are these diarrhoeas’ diseases very dangerous in your society? You may have come across these answers earlier. Please explain this more?

P9: Diarrhea is something that drain their energy, so it is dangerous without a doubt.

I: If that has been that dangerous, how?

P10: It is something that affects the children often times.

I: Any other?

P2: This is caused by not keeping hygiene, so we need…

I: We will discuss the solutions and measures to take later. Let’s now discuss whether diarrhea has been the problem here or not? And how?

P8: It has not been a problem in my village.

P3: It has been a problem in my neighbors. I have been observing many cases like that. The acute diarrhea have been affecting children; it includes an Eight-Month child.

I: Has it been affecting children?

P3: Yes, it has currently been affecting children within the age range of Eight month to the age they sent to class.

I: Is there any other? Has diarrhea been affecting children in your village?

P10: Yes, it has been a problem in our village. It is a problem that often affects children, infants, or not. I know a woman who had taken her child to the hospital after the child had been having diarrhea for seven days. She had learned it was caused by contaminated Indomine *[*a ready-made packed noodles brand*].*  Not only that but my child has also been affected by it; it has been about fifteen days. He had been ill last night. I had treated that and the stomachache had been stopped, but the bacterial disease may still have a chance to retrieve. So, I can say diarrhea is a current problem here.

I: You have been raising good ideas. I want the participation of participants who have not participated yet.

P3: Something has popped into my mind; there has also been an infection that I have been observing.

I: What kind of infection?

P3: Urinary tract infection that has been affecting females starting from the age of 9 months.

I: What are the health facilities you use in your village that provide health services?

P3: Private hospitals and health centers.

I: Okay.

P9: Sometimes, they refer you to Zewditu and Ghandi if that is severe and can’t be treated in health centers. Ambulances take you to these hospitals for serious cases. Ghandi Hospital is the closest of all. It is usually a matter of ability to afford.

I: So, there are private clinics and health centers.

P9: Yes.

I: Where do you get the health services most of the time? Where does society go for health services more?

P9: It is to the health centers.

P10: The health centers were very crowded. Currently, I use a health Centre called Asra Simint. The quality of service is better now; my daughter was born there, and it has been a week since I took my daughter for a check-up. As per my observation, there are improvements in the services.

I: So, you usually go to health centers for health services?

P7: Yes, that is the case most of the time.

I: Is there any different idea?

P1: Most of us go to health centers for health services. Kirkos Health Centre is the closest facility, and that is where we usually get the service. There was also a time when I went to the Zowi Medium clinic to get the treatment for bacterial disease. We also use Zewditu Hospital if we are referred to.

I: Most of you have told me that you use health services at health centers, right?

*All participants: [ nodded with agreement]*

I: If that is so, how much do you pay for the services?

P10: As I heard from the health worker at the health center, we have to pay 30 birrs for the registry card at health centers and 30 birrs for the laboratory service. Now, I am using health insurance for free. Health centers are giving better health services now. People with diarrhea and vomiting are getting better service on time. I use the health center of Kirkos Sub-city; they give first to infants, and that is good.

I: How about the payments in private health facilities? How much do they charge you for the services? You may also include payments for the registry card.

P7: Sometimes we pay 300 birr only for the registry card, with no other service. It is too expensive to get medical treatments here. Unless for rich people, no one can afford this if they don’t borrow. The payments at health centers are fair.

I: Do you get the health services and health workers you want in the health center?

P3: Honestly speaking, we don’t. The services at private health facilities are very expensive but we sometimes have to borrow from others or spend our entire savings to get services with better health workers and with no crowd of patients in reception. Private hospitals may charge you 500 or 600 birr only for registry cards, and the total amount you pay may reach 3,000 birr. In health centers, the doctors don’t usually get on the workplace on time and they mistreat you. That may be caused because of the workload.

I: How far is the health facility from your residence?

P6: I had not been using the health Centre facility at kirkos Sub City. I used to go to private clinics rather than go to health centers even if they were expensive, and I had to pay 300 only for a registry card. This is because the doctors usually get grouchy when giving the service. But, on the 22nd day of the month, my blood pressure raised significantly, and I couldn’t even stand straight, feeling very ill. It takes me 3 or 5 minutes to reach the Heath Center from home. My son had brought me here and I could get a surprisingly satisfying service, I didn’t know there is such improvements on the service quality. I get back home healthy, my six years old son hadn’t expected me to use the health center as he heard me criticizing the service quality. He knew I prefer to get the service from private health facilities and used to borrow money for that if necessary. But that day, I went to the health center and get back healthy. I also saw when the six-month old baby is cured that day. I didn’t like the service before that I don’t even want to see the gate of the health center, but it is improved now. There is still weakness there. It only takes five minutes to get there. Sometimes, the doctors in the night shift come late for the work. That is what happened to me when I came for delivery. I had to wait for two hours, laboring. That was very reckless; some of the workers' attention was on their cellphone, some of them were sleeping. I was crying because of that ignorance, and that is why I hated the service of health centers. Finally, they have referred me to Ghandi Hospital. The service quality is better now.

I: So, the service quality is improved now, and the facility is close to your residence?

P3: Yes.

I: How about you? How far is the health facility from your residence? I am asking you this to know if the distance of the facility from home is one factor not to use the services, okay. The health workers’ performance may be another factor. So, please tell me the distance between yourhome and the facility?

P10: It is within walking distance for me and takes me 10 minutes to reach. Honestly speaking, the service quality is improved now. The health workers were ignorant, and some of them didn’t have good work ethics and all their attention was on their cell phones. We used not to get the medicine we wanted, and we had been purchasing medicine from private pharmacies. I have been getting the medicine available this year. My and within child had never been made to wait longer in reception now.

I: Good, any other?

P2: The services from the health center have been improved now. The health center is close to me and takes 15 minutes of walking for me. Most of the time, they examine you wrong. I used to bring my child and mother for health services to this health center. The results I received from there in Zero Zetegn Health Center were not as accurate as Asrasimint Hospital and Feres Bet.

I: Are those private hospitals?

*All of them: [No, they are health centers]*

P2: They are not private, but are better than Zero Zetegn health center. They provide better service and check up.

I: Any other?

P1: The health center is very close to me and it take me 10 minute of walking from home to health center. I have something I need to comment, I actually have commented this on the meeting arranged by the health center staffs. I have commented them to receive and help women with hypertension when they go upstairs for the service. I have told them they should treat staffs well as the wereda staffs do, I see no change now. I recently brought a women with hypertension and tried to help her when she was trying to go upstairs, the health worker was very ignorant of that poor women’s pain, and was even grouching on her. I criticized her and told her she was there to help such people, the women were asthmatic, and they should show some sympathy. Finally, the assistant had interfered between that rude health worker and me, and things settled. Feres meda health centre is also found in 20 minutes walking distance, but we are registered to use health insurance service at Kirkos Sub City Health Center. That is why we usually go to Kirkos, even though the Feres Meda and Asra simint are better health centres in providing quality service.

I: Thank you. How do you treat your child at home when they get diarrhea?

P7: I treat my child with the water boiled with rice. I also treat with salt and water solutions with lemonade. But, we should bring them to the hospital if that doesn’t help. Children may lose energy and be challenged, so we need to react fast and bring them to the hospital.

I: Any other?

P6: I give him more fluid and whatever he wants, let him drink low concentrated lemonade. If this doesn’t work, I will take him to the health center. I also give a call to the health extension worker at Kirkos sub city, wereda 11 kebele 23. She is a hardworking extension worker and she helps me and my child, she is always there me to tell me whenever I feel sick and cough. She was the one who made me know I was anemic; I had mistaken the symptom of anemia for a headache. Now, I know the symptoms, and I also brought my children when there was diarrhea for over 24 hours.

I: How about you, guys?

P10: In the time of diarrhea, I bought ORS and fed them after I made a solution of it with 1 liter of water.

I: How long do you usually wait before going to take that measure?

P5: If the diarrhea stays for more than two days, I treat them with this solution. I will bring them to the hospital if these all don’t give a cure.

P2: You are asking me about the cultural treatments we give when our children are affected by diarrhea, right?

I: Yes, something you do at home.

P2: I usually treat them with black caraway, biscuits, and also coke, and brought them to the hospital if that doesn’t work and see no change for two days.

I: Any other?

P1: I also know people do what they have mentioned earlier. I usually treat my child with cup of lemonade. I also have been treating my child with yoghurt and boiled powdered potato. This usually works for me.

I: Any other?

P10: I don’t underestimate diarrhea if there is a diarrheal for three time a day. I usually give them holy water first and brought them to hospital if there is no change and do what the doctors tell me to do.

I: Any other? Starting from which age you give them these treatments?

P3: My daughter has been her first time for having diarrhea condition. She is 3 years and seven months now. I treat her with the rice and brought her to hospital after two episode of diarrhea. They have told me it was caused by bacteria. I then started to send her to kindergarten with her own baby pot to minimize the risk of transmission of the bacteria.

I: How about for the infant with the age of less than six month. Do you treat them the same way, or what? Some mothers take treatments them selves, assuming the baby will take in the nutrients through breast feeding.

P1: I have never done that. Some mothers treat them with Tenadam [The leaf uses for cultural treatment method] but I don’t feed anything for babies less than the age of six month.

I: Any other?

P6: There was a trend of giving some kind of candy for their baby to treat them. My neighbor also advised me to drink milk with አብሽ and to feed my breast so as to let him get the solution through the breast feeding. The surprising thing is he is cured of the stomachache, it may be because I beloved. I always suggest mothers not to feed their baby under the age of six months anything. That is what I do for diarrhea, I feed them nothing before the age of 1 year. As the children grow, they develop the ability to survive and their appetite grows. So, we can treat them with different treatments like Tanadam [The leaf uses for cultural treatment method].

I: You have been participating well, thank you. Now, tell me if you measures taken by the society as a treatment for the diarrhea?

P2: For flu…

I: For diarrhea.

P3: As they have mentioned, mothers feed them rice as a treatment.

I: Any other?

P: They say honey and tenadam with water is good for health. But, most of the time it is mothers who believe it works and mothers who live with their mothers use these kind of treatments. Most people in the society believe solution of coffee with honey can be a good cure.

I: Any other?

P3: There was a time I treat my Two-year daughter with honey-mineral water solution and that stopped the diarrhea. I didn’t know it was that curing.

I: You knew that before or you learned that from the society?

P3: My neighbor had told me that and I applied that.

I: You have mentioned that you go to health centers when your children are having diarrhea. Were do you go to other than that to treat the diarrhea?

P4: There is a health facility at Jemo, it is called Enat ena Hitsan. I went there and had gotten registry card and my child has gotten the service. I was treated there and was prescribed medicine and bought the medicine from Kenema (governmental pharmacy). By doing so, the diarrhea is stopped, God is the cause but that was how it was cured.

I: Any other, participate please?

P3: When it is for the health service for my children, I usually use private clinics.

I: Is that the same for diarrhea?

P9: Yes, I use the service of health centers or higher hospitals when it is for me. Unless for the vaccination I have never brought my children to health centers. There is a private hospital called Biham nearby, I brought my children there for treatments.

I: Any other idea?

P4: There is also a trend of taking children to churches to take holy communion and take holy water for the cure. As a believer, it usually works.

I: Do you use medicine for treatment? If so, what are those medicines? Do you have a trend of taking medicine without out prescription?

P3: I let my children take medicine for bacteria every three months.

I: What kind of medicine?

P3: Medicine for worms, the medicine that we take in as a shot. But I always say that I have done that. I also buy medicine for tonsillitis from the pharmacy.

I: You buy that from pharmacies?

P3: Yes, it is from pharmacy.

I: You all must have experience on this matter, what medicine do you use for treatments, and how? Please participate.

P1: I bought the medicines from Kenema after the doctor’s prescription.

I: Okay, from Kenema?

P6: Yes, she told me I could get medicine from Kenema and Red Cross, stadium branch. The prices of medicine are higher at private pharmacies so I had to buy from Red Cross pharmacies, the price difference between the private pharmacy I asked to buy from and governmental pharmacies was 400 birr.

I: Any other? Where do you get the medicines from? Some of you have said, from pharmacies, traditional places and like that. Where do you and the community purchases medicine for diarrhea?

P1: The most common health problem occurring on my child is bacteria. *[ sorry for my voice, it is because I am having flu]*

I: It’s okay.

P1: So, I buy the medicines from the health insurance system. If I don’t get it from there, I buy from Kenema pharmacy.

I: You buy from Kenema when prescribed, right?

P1: Yes.

I: Any other?

P10: I usually buy medicines from the health center. But I know neighbors who buy medicine many times, by just using the prescription they have received for one time. They also recommend for others, with out any diagnosis, I always tell them that is wrong. Some people even share the medicine prescribed for them. People shouldn’t share medicine prescribed for them and shouldn’t buy with out being treated and diagnosed.

I: Any other?

P5: My wife had been taking the prenatal check-ups here in Tikur Ambessa Hospital. We were learned our baby had no limbs at all after the ultrasonic examination. We were told the baby wouldn’t survive for more than a month and it will die. He is still living and he was born with limbs, unlike their prediction. The only problem he have had is heart disease, we buy the medicine from here. If we don’t get it here, we show the prescription and receipt from here buy from Kenema. But the person who has cash can get the medicine easily from kenema, people who brought the receipt will get the medicine last. I want this problem to be solved.

I: Okay. Is there any other idea on this?

P6: Most of the time, the pharmacies don’t sell you the medicine without prescription, especially the medicine for children. The pharmacist in Kirkos don’t usually sell medicines for worm and others with

Out prescription of doctors. When I was at kazanchis, before 9 years, they used to sell us medicine without prescription. Currently, pharmacies don’t do that especially for children. I really liked this, people should be prescribed by doctors before purchasing the medicines. I usually heard people complaining about not buying with out prescription.

I: Thank you. What are the good opportunities and challenges people get when bringing your children having diarrhea?

P6: Most of the time the workers miss-treat the diarrhea patients unlike for the treatment given for flu or other disease. The other challenge is, children react different when they come to the health facilities. Sometimes they become incapable of pooping even they had been pooping now and then at home. It is a major challenge for mothers, after facing all those treatments and get to the doctors finally they sometimes can’t poop, that is very challenging.

I: What about the good opportunities that made you go for the services?

P6: When that happen, some good doctors tell you to come back 5 minutes after the baby poops at home and solve the problems. Some doctors are as good as this and even tell us we need not to re-register for service. This is the good opportunity.

I: So, these are the good opportunities and challenges you have been facing?

P6: Yes.

I: Any other? Isn’t the question clear?

P3: Sometimes we may be urged to bring our children to health facilities at night. It is expected to the doctor to fell asleep at night. But, they at least have to wakeup faster when called for the service. We have patients in our hand, the health of patient should be given priority than the comfort of the worker. I had brought my child some night and my child was crying so bad, the health workers made us to wait long and they finally diagnosis her with pneumonia. I had to pay 500 birr and I couldn’t get that at that time and I had to wait till morning. Later, they rechecked her and found it wasn’t pneumonia. I bought medicine to get ride of that flu like disease and my child is still taking it in.

I: How about the good opportunities that made you to use the health service more?

P7: There could not be good opportunities from that illness.

I: I think you didn’t get me.

P10: I went to the health center eight years ago, the good opportunity was the fact they react fast and take a stool taste. The challenge was, that there was no power and they couldn’t use the laboratory. That was the challenge I can mention.

I: So, not having a reserve generator for power is one challenge, right?

P10: Yes.

I: You also have mentioned the unavailability of health workers at night as a challenge and the integrity of some health workers as a good opportunity, right?

P10: Yes.

P3: I always remember the day I brought my neighbor for treating acute diarrhea at Yekatit 12 hospital. Even though there was a high crowd of patients, she could get the service fast at night and get back home in the morning. That was a good thing I always remember that day.

I: What are the causes of diarrhea disease for infants? You were mentioning it earlier.

P8: The bacteria from the pollution of the environment and contamination of foods are major causes of diarrhea.

I: Okay, any other?

P3: As per my experience feeding vegetables like cabbage is also one cause for diarrhea affecting my children. I fed my children vegetables and they had affected them with diarrhea. Ignorance of family for the health of the children and environmental pollution are some of the causes. May be it is because mothers are busy.

I: Any other?

P3: Expired and ill handled juices and candies, and chocolate are also causes. I know a neighbor who used to fed those and got diarrhea. I could observe that from neighbors.

I: Any other?

P1: The other cause is the habit of children to take in anything they get on the floor. This made children below the age of two, to be vulnerable for bacterial disease.

I: Any other?

P6: We usually don’t wash our hands and breasts before feeding the infants below the age of two. This make them vulnerable. Even they always urge us to feed them, we have to make sure we washed our hands before feeding them. The other cause is ignorance of mothers when the children below the age of two cry with pain, thinking that is just caused when milk teethes grow. That is not always the case, as that is the time babies start crawling they become vulnerable for diseases. Once they become ill, their immunity declines. We should be ware of the signs the children show, diseases caused by water contamination is more severe than disease caused by food contamination of food. After they get sick for the first time, they will be infected frequently as the bacteria can’t get out of the body easily.

I: How about the measures you have been taking as a solution? Please mention not suggestions, but measures you have been taking or you have learned from others to prevent diarrhea?

P2: As most people here are poor the time people like me put waters outside of home with tots. Children drink this water who is vulnerable to disease-causing organisms and sunlight. So, I always tell my family, to put the water-holding tots inside the home inside to prevent the water from the sunlight and chemicals from the plastic tots. As a poor family, we always eat a Shiro stew that is made for three or four days. That is not good for the health of children. So, I usually insist my children shouldn’t eat like that and let always them be fed fresh.

I: Okay. What other prevention methods you have implemented or heard of?

P3: My daughter was born at eight months and her skin was sensitive. For this reason, I have been using Dettol for washing starting from the time she was in the incubation room. I also have been washing the floor with fluid detergents when she started crawling. In addition to those, I have been using methods like inserting disinfectant called Wuha Agar, bottled water and boiling and cooling drinking water, the boiled water doesn’t taste good though.

I: So, you have been preventing by using Dettol and Wuha Agar?

P3: Yes.

I: Any other?

P5: I am informed that , the cause for the bacterial disease my child affected with, is contamination of water. So I have been boiling and cooling drinking water since my child was sick. I used to let him drink water from tap before that.

I: Okay. How about the prevention methods we observe from the society?

P6: We usually ignore the fact that finger nails can accumulate dirt and create suitable situation for contamination. So, we need to trim their nail, provide eating plates only dedicated for them, and keep our personal hygiene before cooking.

I: Okay, what should the society do to prevent diarrhea?

P3: I think, creating awareness for the community in the weredas could brought good changes. We need to eat fresh and the health workers should teach this.

I: What measure the society been doing to prevent diarrhea? You have been mentioning some earlier, right?

P1: The playing grounds of children should kept clean, because children may eat bread and other there.

I: I am asking measures those have already been taken by the community?

P2: We shall discuss about the methods to keep our environment clean.

I: Tell me if the society has done things like that?

P1: The health extension workers had been distributing water disinfectant called Wuha agar. I have been participated on activity as I was one of the facilitators. There was a pandemic here and we had been teaching how to use this disinfectant with boiled and cooled water. I also believe we should keep personal hygiene of children by providing those neat clothes trim their nails regularly and I have been doing that. The big problem is the transmission. Even you keep your children’s personal hygiene, the children make contact with each other. So, I have been giving advises for neighbors who have just buy sweets and chips for their children from shops with low hygiene. I sometimes tell the neighbors “Chips are fried with oil made of donkey fat.” Kids may love sweets, but we should not give them for the sake of their health. I have never bought those for my child and he is healthy now, Allhamdulila!

P2: In my village, the people have a schedule to clean their environment twice a week in wereda level. People around here also clean their surroundings on Sunday. This is all to protect the children from the disease.

I: Thank you. Let’s now discuss vaccines, what is the understanding of society about vaccines?

P2: We get vaccination services at health center for our children below the age of five. Vaccines are very crucial and mothers should always bring their children for vaccination and better not to miss one.

I: What is the awareness of society about vaccines?

P6: As a culture, we always ask mothers how she is doing and the facility where she was getting antenatal check-up from. We also discuss the vaccines and exchange information every time we see pregnant women. It has become a trend to share vaccine information now. The health extension workers also provide awareness-creating programs, and people’s awareness of vaccination has increased in these 16 years.

I: Any other ideas on this matter?

P3: We may be able to follow up and bring our children for vaccination for three years. That is because the health extension always reminds you to. But, we usually don’t do that well after the third year, we usually forget to take the vaccine after the third year. I want to appreciate the health extension workers because they work hard to follow us and take the vaccination service regularly.

P9: We mothers, young or old, are very serious about vaccination. I wish we all do the same for the other measures taken for health. To share my experience with you, my son is a year and three months years old and I know he should get injections for nine months and then vitamins after that. The health workers had to write for me to receive vitamins in a year and three months time. But they didn’t, I was confused and asked other sisters even people were telling me they are not prescribing that on year and three month time any more. Then, I have asked the sister and she told me I should receive the vitamin even they don’t write it for me. She have told me, I had to remember my self and get the service. So, we should take care on the schedules. The society has good awareness on vaccine now.

I: Why do you think the society is well aware of it and accept it well?

P3: It is because we do it for the sake of children’s health.

P9: It is because we know from reading, the vaccine boost children's immunity and prevent them from a diseases that cause their foot to be clubbed. That is why society accepted it well.

I: Do you all have the same opinion on this?

*All participants: [nodded with agreement]*

I: If that is so. Please tell me what you know about Rotavirus. As you know, it is given to prevent diarrhoea. It has been included in vaccination programs.

P3: What is that, is that like ... አክታ? Or polio?

I: It is the vaccine type you are given in three rounds. They don’t tell you the name?

*All of them: [Yes]*

I: You don’t know its name but you identified what it is, right?

*All of them: [Yes ]*

I: They don’t tell you the purposes of each vaccine you take.

*All of them: [No]*

I: Okay, it was better if you ask the purpose of each vaccination and know what is what.

P3: I think the vaccination name is also on the card.

I: You are right.

P2: We just come in scheduled and take it, we don’t specifically know the purposes of each vaccine.

I: Okay. What is the understanding of the society about this vaccine?

P5: Even though we are unaware of it, we are taking the vaccination well. We weren’t learned the purposes though.

I: Okay. The purpose of this study is to know the cause for the spreading Rota virus regardless of the vaccination that has been given for the people. What do you think is the cause for that? For example, people may still get infected with TB even they had had the BCG vaccination on their 45 day after given birth, it actually can be less severe than the person who wasn’t vaccinated. In addition to we want inputs to produce vaccines called shigela and E.coli. The health workers have been identifying the shigela and E.coli bacteria when patients come for diarrhoea treatment. There has been orientation programs given and I think one of you has participated in wereda. So, I want to inform you that the health workers in Kirkos and Teklehaimanot health centres are now trying to identify diarrheal cases caused by these bacteria types when people come after three consecutive diarrhoea. You also need to share this information for others. I explained you this for more clarification, now you need to participate more, okay?

I think are well aware of it now, how aware is the people about this rota virus? What is your experience and the understanding of the society on this rota virus and the symptoms you observe on your children?

P1: It is obvious the society share information when new kind of diseases spread because we all need the health of our children.

I: By the way it has been while since the rota virus vaccination has started, it was since 2000 E.C. The study purpose is to produce modified vaccines. As it was started after 2000EC, most parents don’t take it as a child. That is why we are asking the awareness of the society?

P4: We have never told such kind of things there in health centres, we just take it and go home.

P2: You are informing us about this, so we will share the information with others, and that continues to spread. It is very important information. I also take my daughter myself and see the vaccination process.

I: There is a vaccine they injected around here *[locating her* thigh*?*

P9: Yes, they take about 5 vaccines, two here and the other three here *[* showing left hand and on their thigh respectively *]*. We just let them take all vaccinations but don’t know which is given for what.

I: You have been taking three types of vaccines right?

P2: Yes.

I: You have also been taking the vaccine in the form of a drop, right? Since 2000 E.C. they introduced vaccines given as drops, other than the Polio vaccine. It is given in three rounds.

P3: Oh...really! We have no clue about that.

I: So, you all have no awareness about that?

*All of them: [No]*

I: Where do you get these vaccinations?

P1: At health centres.

P2: Some rich families may bring their children to private clinics for vaccination.

P3: But, health centres are more reliable than private clinics in the case of vaccines, governmental facilities are more responsible.

I: Okay, if there is some who is aware of the vaccine, please tell me if there is different answer?

P3: The health workers don’t usually explain us the purposes of the vaccine. They urge us to get vaccinated and go home

P1: They used to teach our mothers about the vaccine type and purposes while they are in waiting line. My mother was well aware of the small pox and polio vaccine. But they are not doing that now, they just vaccinate and certify. There were good sisters in kirkos health canter who always teach mothers about the vaccines. But, we educated persons are not even aware of this.

I: I already have told you about the vaccine. So, what is your concern when you get back to home, after vaccination?

P6: We usually ask them about the vaccines you have told us. They don’t explain us anything and it would be nice if they explain the purposes of each vaccines given in any form. In that cases, I can inform others and the society would be well aware of it. They have to explain about the vaccinations.

I: They write the type of the vaccine on your cards, right?

P1: They do.

P2: You can’t read it, though.

I: Maybe it’s because it is written in abbreviations.

P3: The pharmacist may tell you about the vaccines, but the others don’t.

I: Okay. What are the good opportunities and challenges to get this vaccination services?

P3: We are only given two days per week to get vaccination services. It is not enough because mothers are busy with jobs, the service should at least be given 5 days a week. There should also be awareness creation at schools and at home.

I: Very good, any other?

P6: There have been relentless efforts made to persuade people to get vaccination services in the past. Vaccination types are increasing in type now, the vaccine certificate I have received is very different with the current one. As long as we accepted their benefit, it would be better if the health workers showed integrity to explain the vaccines. I usually ask about the symptoms and the purposes of vaccines, but they just vaccinate and register you. There should be awareness creations given regularly. If we keep giving the service like this, someday we may lose cause for taking our children for vaccination. It is shameful for a mother not to know why he exactly brought her child for vaccination. There should be better explanation about the purposes to give us trust on vaccination and transfer the knowledge through generations.

I: Okay. You may also mention the good opportunity that made you go to vaccination service.

P6: We are usually busy, so there may not be a way we could gather for education. So, we can provide brochures on which the details of the vaccines are written, and we can learn from that. I don’t think 90 per cent of mothers can be gathered and learn with patience about the purposes of the vaccines.

I: Very good. Any other?

P3: I have given birth to my two children at Gandhi Hospital. They usually show us on plasma what we should do after six months or like that. So, it is not necessary to teach mothers by gathering them. They can just teach us by videos and cartoons. The good thing about Gandi hospital is that we can get vaccination services from where we get postnatal services, instead of coming back for vaccination. I don’t know how the health services work on this though.

I: Any other please?

P2: I think the government should consider providing medical related programs about this matter on TV. The Tvs are full of programs of sport, they should also inform about this.

I: Okay. The result of study is given for policy makers and they will consider filling the gaps. We will also work on media coverage largely.

Are there any cultural or religious believes related to vaccination?

P2: In Gurage culture, children get stabbed on their back. There is believe such act can increase your performance in education.

I: How about cultural and religious believes that prohibits the vaccination?

P2: The priests teach us to take the vaccines and necessary medications side to side with Holy Communion and other religious solutions like holy water when we get sick.

P10: As a people living in Addis Ababa city, we tend to use more modern solution. We only go to health centres for health services rather than applying those cultural solutions.

P6: We can observe that, both Christians and Muslims in our neighbourhood use these modern ways of preventing disease side to side with Dua (prayer) or Holy water. There is a better understanding now even there are still gaps. Some people used to advise me not to go for vaccination, but people are well aware of it now. The priests may give us Kiba Kidus to cure on Sunday, but if they see no change they tell us to go to doctors on Monday. If the health workers give more health educations, we would have more awareness and always choose the appropriate solutions.

P2: Sometimes the religious fathers advise us to go to health facilities if we try the religious solutions and don’t work.

I: There is actually no covid pandemic currently. But try to recall the time when the covid pandemic was at its peak and tell me how did it impacts the process of vaccination ?

P3: Oh my God, the covid 19 pandemic has made us to forget the vaccination at all. We couldn’t even get outside of home that time.

P2: The covid pandemic has made vaccination impossible, both adults and children weren’t taking the vaccine.

I: Any other?

P6: I were very frightened for my child and didn’t want to go for vaccination. I was sceptical because I thought they may accidentally made mistake on vaccination. But my older son was giving me advises to go for the vaccination.

I: Do you have a doubt and concern on the safety of the rota virus?

P2: We weren’t affected by it, but we were concerned.

I: I was asking about the vaccine for the children?

P3: Yes, sometimes we are concerned. We sometimes hear that they vaccinate wrong type or something like that you hear from many people and you tubers.

I: Any other concerns you have on the vaccination?

P6: Sometimes, there may happen coincidence. Rash and headache may happen on children at the same day they get vaccinated. This made you believe it was caused by the vaccine. I always have concerns every time they get vaccinated. It is always a lot of worries especially within three days after the vaccine.

I: They don’t tell you the side effects?

P6: They tell you on the first vaccination, but don’t tell us after that. They first tell us there are side effects like fever and swellings and not to worry.

P3: They tell us to bring them back if the side effects stay longer.

I: Okay. Even if you believe the vaccine is a crucial side, you still have concerns about the vaccine. Is there any other idea?

If not, please add if you have anything to add.

*All participants: [Silent]*

I: If not, I want to thank you all for your participation. The discussion will be used as input for the study made at Kirkos and Teklehaimanot health centres. This contribution will greatly help your children and the society.
