# Supplementary material for: Exploration of the social determinants of diarrhoea, rotavirus vaccine uptake, and vaccine ‘fatigue’ in Ethiopia, Kenya, and Malawi
Source: PLoS One. 2025 Sep 9;20(9):e0319691. doi: 10.1371/journal.pone.0319691 (PMC12419581; doi:10.1371/journal.pone.0319691)
Supplement: S1 Data — (ZIP) [file pone.0319691.s001.zip › Supporting Information Files/MW_4FGD.docx]

**Facilitator:** we are now starting, okay

**All:** yes

**Facilitator:**  as I said, we are going to discuss about diarrhea and the rotavirus vaccine, before that, I would like us to discuss diseases that affect children under 5 here at Bangwe or the communities where you come from, you will tell us your number before telling us your opinion

**02:** I think the season that we are in, the disease that is common in children is diarrhea, and because of hunger in this country, diarrhea, and malnutrition are affecting children

**04:** other diseases that are affecting children here in Bangwe are asthma and trachoma

**08:** I think malaria is also affecting us here in Bangwe, it is affecting children mostly

**09:** here in Bangwe, children are affected by cough, this disease is really affecting children

**01:** I just want to add on diseases that my colleagues mentioned (not clear)

**03:** to add on that, skin rash is also affecting children

**Facilitator:** is it common here?

**03:** yes

**05:** there is another disease that most people don’t know the name of, some people just say skin rash, and maybe you can tell us

**Facilitator:** What does it look like?

**05:** the patient normally has a burning sensation

**Facilitator:** Is it like skin rash?

**05:**  yes

**07:** I think diarrhea is affecting children here in Bnangwe because of poor hygiene

**Facilitator:** That’s what you think is causing diarrhea here in Bangwe

**07:** yes

**Facilitator:** Okay, I believe every disease is a human problem isn’t it?

**All:** yes

**Facilitator:** what are the main health concerns here in Bangwe?

**02:** It’s diarrhea and the skin diseases that are now common. There is a new one called chicken pox, children are affected

**09:** I think every disease, because when someone gets sick, he/she feels pain, and I cannot say if I could have gotten sick from this disease (not clear)

**Facilitator:** What you are saying is very true, and we are not belittling any disease here, but if the government would like to intervene or there is that capability for treatment, which disease do you think they should start with?

**ALL:** (cross-talk)

**3:** it’s diarrhea in children because you hear parents saying ‘’The child is teething!’’ eeh so they got used to the disease, so diarrhea is a big problem for under-5 children

**Facilitator:** what else apart from diarrhea?

**05:** malaria poses a risk to children, we should take part in helping our leaders

**01:** (not clear)

**Facilitator:** Okay, is there any other disease? We are not denying what you have mentioned but, if we can discuss all diseases, it will take us the whole day. We will focus on diarrhea, why is it a health concern? This one talked about hygiene, he should tell us what he meant

**07:** I should start with water, once water is brought home, you notice it’s neither covered nor treated with chlorine. A cup that we are using is not even cleaned, that’s what we mean when we say lack of hygiene practices

**Facilitator:** So, household hygiene

**07:** yes

**09:** apart from household hygiene, let’s look at our markets and towns. We can buy a mango and start eating without washing it and once we have finished, we just litter everywhere, then throw it in a bin, so there are houseflies everywhere. Such things worsen the problem instead of sorting out the problem

**Facilitator:** community hygiene practices as well

**09:** yes

**06:** we should have proper toilets in our homes for us to prevent cholera

**Facilitator:** what are these proper toilets?

**06:** some people just leave a toilet uncovered, and when houseflies land on it, it also land on plates, so we end up getting sick

**04:** what happens to children of 2 to 3 years old, for them to have diarrhea, they just pick soil and eat, and that causes diarrhea to them

**Facilitator:** on community hygiene, where are your sources of drinking water?

**03:** for us, we are living in a rural area though it’s Bangwe, so we use water from boreholes, if it is functional, some drink water from the wells

**Facilitator:** a dug one

**03:** eeh (yes)

**Facilitator:** how are these wells cleaned?

**03:** Aah when it is dug, women remove the water and clean it, then we start drinking from the well, it’s not even cemented, if there is a cemented one, ‘’Is it yours?’’

**All:** (cross-talk)

**02:** to add on the issue of wells, we rely on the wells, and they can be here, and toilets up there. During the rainy season, runoff water can start coming here (cross-talk)

**Facilitator:** what about rivers? You may talk about it, though this is a rural area but, Bangwe is semi-urban, there are rivers and sometimes people rely on waters from these rivers

**09:** we use water from the rivers for washing and cleaning the utensils, and not drinking (not clear)

**07:** we wash plates, and before they are dry, we use them and maybe there are sewage systems up there, they all end up in that river (cross-talk). There are things that women are using nowadays….

**00:** (pampers

**07:** instead of throwing used pampers in a toilet or in a pit, they throw them in the river, and someone will clean her/his vegetables in the same river

**00:** those vegetables may be taken to a market and somebody buys them, this contributes to diarrhea as well

**Facilitator:** Okay, let’s discuss malaria. Why is malaria a health concern in this community?

**07:** because of the lack of bed nets, most children don’t sleep under the nets, it’s easy to catch malaria

**Facilitator:** do parents sleep under the nets

**All:** (cross-talk)

**07:** Bed net is important, it protects us from malaria

**05:** Bed nets that were distributed back then are worn out, most of us have no bed nets. So if we say only children don’t sleep under the bed nets

**All:** laughing

**09:** most people don’t believe in bed nets now because when they have hanged a net, a few weeks later, there are bed bugs at the corners of the bed net, so most people don’t sleep under the bed net because they think the bed bugs have come because of the bed nets

**Facilitator:** when they stop using the bed nets, do bed bugs….

**09:** (they go away

**All:** cross-talk

**00:** it’s just an opinion if there are bed bugs in the house, even if you remove the bed nets, bed bugs will hide somewhere and come at night, and the bed net is treated, it is difficult for it to have bed bugs

**Facilitator:** Okay

**00:** when using a bed net, we prevent mosquitos that are already there, the question is where do the mosquitos come from? We need to fix where mosquitos breed from. So, we should clear the bushes around homes and stagnant waters. Some keep chickens and ducks, and we keep stagnant waters and mosquitos breed there. We have bushes that are not cleared, we can find a way how mosquitos come about

**0:** before mosquitoes come to the bed nets

**00:** Sure

**Facilitator:** mmh, when you look at stagnant waters that are in our homes, are there many so they can contribute to mosquito breeding?

**00:** stagnant waters are normally available when the rainy season is going toward the end (not clear)

**Facilitator:** mmh

**00:** stagnant waters that are available are from the toilets because that’s where the mosquitoes hide as well

**Facilitator:** ooh, which months is malaria most common?

**05:** In summer, because summer is friendly to mosquitoes when there is no wind. When it’s windy, mosquitoes go in that direction (not clear) we sleep without covering our heads with a blanket, and we find that all mosquitos are in the house attacking you

**ALL:** laughing (cross-talk)

**Facilitator:** Alright. You mentioned another disease which is also a health concern here, what was it?

**02:** skin rashes

**Facilitator:** why is it a common health problem here?

**02:** fluids from the wounds, so, diseases like scabies, once those fluids contact someone, it is transmitted, so it is about hygiene as well, we should be practicing hygiene (not clear) and I should make sure I don’t spread the disease to others. For example, if I am bathing from the river and someone is bathing in the same river downwards, I can transmit it. Sharing clothes amongst children of the same household due to poverty, ‘’wear your this clothes of your younger sister/brother’’ in the process, you transmit it within that house, so it is about hygiene issue. Once we have realized this problem, we should rush to the hospital because when we go there, health workers will tell us what to do, they can give us medication, and instead of getting sick, we can realize that we are okay, rushing to the hospital

**Facilitator:** let’s hear from others, why do you think skin disease is a healthy problem here?

**05:** the issue is what he has said, the thing is, when you get sick from this disease, the main thing is to rush to the hospital and see the doctor because when you just stay, the disease will also attack other family members, but, when we go to the hospital, you protect yourself and the family members, that’s what I think

**01:** the issue is, that we share soap and we use one sponge as well sharing of bathing towel

**Facilitator:** mmh

**01:**  this makes it hard to defeat it at a household level because of poor economic sources that result in sharing bathing materials

**Facilitator:** Okay, any additional thoughts

**2:** on that issue of poverty, most people don’t use the necessary soap that can reduce infections, the soap that we are using is contributing to sickness, and there are different kinds of soap, so we go by what is cheaper

**All:** laughing

**Facilitator:** which soap is mostly used due to poor economic sources?

**2:** soap that has no names

**All:** laughing

**2:** some washing soap when you use them, clothes get dirty and you wonder what happens

**All:** laughing

**00:** you find soap at the market sold at K200 and you go by that because it is relatively cheaper (cross-talk)

**Facilitator:** Alright, we have talked of rushing to the hospital when we get sick of course, we are not talking about children only but everyone, where do you go when you get sick?

**04:** when I get sick, I buy drugs from the grocery or pharmacy but, the medication that I take may be irrelevant to my illness, so I just want to relieve pain, that’s the first treatment. When the condition gets worse, that’s now where some people tell you, ‘’let’s go to the hospital to seek medical treatment’’

**01:** gentlemen, when someone gets sick, the first thing is to go to the hospital, that’s my opinion

**Facilitator:** Okay, we are supposed to go but, let’s talk about what we do. We are going to discuss how important is it for us to go to the hospital but, let’s focus on what we do

**01:** the issue is what has been said that when you feel a headache, you buy Panado but, when you notice that the condition is not improving, you go to the necessary people who know these things because you can think it’s a headache and you buy panado yet, it’s not relevant to headache, so it may worsen the condition

**09:** most people go to the church prophets to be prayed for, so they were told not to go to the hospital because biomedical drugs are Satanism, ‘’just come for prayers.’’ Some people have their traditional doctors based on their beliefs

**00:** This is common because some people think they are bewitched, ‘’You should not waste time going to the hospital’’ (cross-talk)

**0:** ‘’They will hit you’’

**00:** ‘’With a hummer, they (witches) play with your head at night.’’ That’s what happens

**Facilitator:** let’s focus on the issue of buying drugs from the groceries, is it everyone who does this, or how common is this?

**04:** to be honest, this is common and most of us do this practice of buying drugs from the groceries or consulting traditional doctors. For example, a certain woman was in Thyolo, her home village, and her child got sick, it seemed like malaria, another one had malaria too and the mother got sick too. Some people told her it was because of water, ‘’you need to mix water from Thyolo and water from here, then you should mix them with traditional medicine, then you should take it.’’ If she started getting better, it was these days because she went to the hospital, but when her friends assisted her, they told her it was because of the weather from Thekerani and now she had to get acclimated and for her to adapt, she had to take a mixture of water from here and the traditional medicine

**Facilitator:** let’s hear from others…..among the women, men, and children, which group commonly uses….

**04:** (drugs from the groceries

**Facilitator:** yes

**04:** it is men, most men don’t go to the hospital, and they just buy drugs from groceries or pharmacies. When they feel a headache, they buy drugs

**All:** laughing

**03:** if ever there is a person who is afraid of going to the hospital, it is men

**Facilitator:** mmh

**03:** women go to the hospital but not men, men do persevere and for them to go to the hospital, they should be carried because he is done, so they cannot go to the hospital by themselves, he takes it as a burden

**Facilitator:** we are all men, if we were speaking for someone, (24:14 not clear). When you are chatting with women, they say the same things that men are weaker, we would like to hear, why is it so difficult for men to go to the hospital?

**2:** it is true that most men don’t go to the hospital, they hesitate, even myself, I just buy drugs (cross-talk) I feel too busy to go to the hospital and stand in the queue, I feel it is something

**00:** you feel it is a waste of time, ‘’with this condition, should I bother going to the hospital? I will be okay’’

**09:** the problem is that we underrate the illness, so when you go to the hospital and see someone crying bitterly and you only have a toothache, you start undermining your illness. When you see a patient rolling over a bed, you say, ‘’This is a patient and I am not, how have I managed to walk up to here? The patient is this one on the bed’’ you haven’t met with a doctor, you go back home. So, you think the one on the bed is a patient, and not you. You also think that you will waste your time at the hospital, instead of crafting your hoe handles, and doing some things, you think, ‘’Should I go there?’’ so you send a child, ‘’Go buy panado, I going to dambo, you will get me there, please bring water as well’’ It’s done

**01:** most men fear going to the hospital because they are afraid of being tested for blood

**All:** laughing

**01:** my friend got sick and I told him to go to the hospital, he told me, ‘’I cannot go to the hospital because I was told that whenever you go to the hospital, the first thing is a blood test, I don’t want to be blood tested’’

**All:** laughing

**01:** most men don’t want to test blood

**00:** most of the time men have self-doubt because of our behavior when we go out, so we tell ourselves, ‘’I am not behaving well and when I go there (hospital) I will be diagnosed with it, when this one knows my status, things will not be okay with me, so let me stay,’’ forgetting that we are spreading the diseases and when we would have gone to the hospital, we would have been assisted and the disease would have been reduced, so we just worsen the condition because of shy and thinking that we are going to waste of our time (cross-talk) it is this who is supposed to be there and not me, isolating ourselves yet, we are making a wrong decision

**Facilitator:** you wanted to speak

**0:** I will talk about why men don’t go to the hospital, for example, a man can go to the hospital, and a doctor may tell him to buy drugs from the pharmacy or is given panado, so he asks himself, ‘’I have come here all the way from home, only to be given panado, I have K50 or K100, I could have bought panado.’’ That also discourages a man not to go to the hospital, they just buy panado. Most men don’t just stay and buy panado because the treatment they get from the hospital and pharmacy is different, this discourages men. Sometimes they go to the hospital earlier, just like someone said, you can have a toothache and to the hospital earlier and stand in a queue. If the facility opens at 8, they open at 10 instead, so he thinks, up to now doctor has not yet come, it also discourages a man from going to the hospital, and he thinks of going to the private or traditional doctor to have his tooth removed, so what happens at the hospital makes men not to go to the hospital

**Facilitator:** Okay, let me give this example, he said that he had a toothache and he consulted a prophet

**All:** cross-talk

**Facilitator:** what do men do when they have diarrhea?

**0:** on the issue of….

**Facilitator:** (knowing that we don’t go to the hospital

**0:** my thoughts are that when men have diarrhea, we have our beliefs, we may cut apiece from a mango tree or aloe vera and take it, that’s medication for diarrhea, and such things help men. When they have diarrhea, they use natural herbs like aloe vera, mango, or avocado pear tree when someone has anemia, so they use such things

**Facilitator:** when they use these things, does the condition improve or it’s just a belief?

**0:** to some, these things improve their conditions to some don’t depending on the suitability of the medication

**00:** some groceries have medication for diarrhea

**Facilitator:**  like what

**00:** fragile (cross-talk) clear clear

**All:** (laughing)

**0:** water mixed with salt and sugar

**00:** if there is diarrhea that frustrates people, it’s cholera

**Facilitator:** ooh

**00:** (not clear) has its medication, people use herbs and they get cured but not cholera, it is difficult. In the past, people could know very late, they could try this and that, trying a mango tree

**All:** cross-talk

**00:** you know when the condition has deteriorated

**Facilitator:** we are saying, that men don’t go to the hospital, and instead, they just buy drugs to relieve pain. I would like to know, do you think it’s possible for a man to use health services?

**00:** it is possible

**Facilitator:** what can make it possible?

**00:** it can be possible through the meetings, our leaders and healthcare workers should initiate the meetings with community members, sensitizing them to the importance of going to the hospital first when you get sick. People do some things as usual, they take things for granted. Just like some peoples’ allegiance to a certain church, ‘’My father was born in this church and I cannot go to any other church,’’

**Facilitator:** How can this practice come to an end?

**05:** just like this meeting, we have got a responsibility to take part, because where we come from, there are people who practice this behavior. After this meeting, if we have learned something when we get back home, we can pass it on to them (not clear) from today on, we can start another step

**00:** sometimes have a meeting with men as you have done today, don’t just say men are stubborn, you have given a certain power for us to take a step (not clear). Healthcare workers try, they say when a woman starts an ante-natal clinic, her husband should accompany her, some men go, and some don’t, but, it’s necessary when a woman is going to the ante-natal clinic, her husband should accompany her (not clear) but, health care workers prioritize men because they are the ones who stay closer with children, so men are left behind. About what this one said, you can go to the hospital earlier and stay in the queue, go to the doctor's room (consultation room), they know they have no medication for what you are complaining about, they leave you to go to the doctor’s room, registration up to the pharmacy yet they know we have no medication for this illness. You stay there up until 10 O’clock and you get to the pharmacy, ‘’there are no drugs for your disease, buy from the pharmacy.’’ As someone who looks for money so that children can have food, you feel your time has been wasted (cross-talk)

**Facilitator:** what you are saying is very important, so one should speak after another

**00:** please, as a government, you should improve on that so that when someone gets to the hospital, should be sure that he/she is going to be assisted, and where possible they should be coming in the morning and tell patients, ‘’Those who have such and such a condition, we don’t have medication for that.’’ This is why people go to private hospitals because when you go to private hospitals, you are assisted instantly but those who are poor cannot afford private hospitals, so what can they do

**Facilitator:** hospitals that you are saying have no medication are where most women go. Why there is this difference? If there are no drugs, what motivates women to go to the hospital?

**00:** it’s their practice, they say when I get sick, I should go to the hospital while a man thinks that for me to go the hospital, the condition should be worse, the illness that everyone knows there is a patient there, but not sickness which I can get sick and still be able to walk. I may get sick and come here like this, but that one is not regarded as a sickness, undermining the illness

**01:** most men force themselves themselves when they are sick and they say, ‘’I am sick of course but, who is going to provide food if I don’t go to work?’’ (cross-talk) as my colleague said they go to the hospital when the condition worsens, so, they are forced to go to work because some work with Indians, and they are difficult bosses, when you tell them I went to the hospital, they cannot take it, so they go to work when they are not feeling okay. They go to the hospital when their condition is worse

**00:** they are carried

**Facilitator:** how long do people travel to get to the hospital?

**0:** from Mbalame to Bangwe clinic, is a long distance. To use a bicycle, we pay 2 to 3000

**Facilitator:** one way?

**0:** (cross-talk) Yes, if it’s night, you are in trouble because you wait until morning because that time, no bicycle operator works

**00:** they refuse to travel at night (cross-talk)

**0:** last week I went to the hospital when my child got sick at night from where I stay to a clinic, I paid K4000 because he took advantage of my situation, so he could not receive 2000

**0:** during cholera outbreaks, my nephew/niece had cholera. We traveled up to number one on foot. We then asked a bicycle operator how much from there to the clinic, and he told us to pay K1000. We were supposed to pay 300 or 400 but, he charged us 1000 and we paid him, so there is a long distance that makes also us not to go to the hospital when we get sick

**08:** we think of this long distance, as my colleague said, you go through all stages of the clinic and they are told there are no prescribed drugs

**Facilitator:** please remember your numbers

**09:** on top of the long distance, there are poor road networks. If the patient is very serious and cannot sit on a bicycle, the car cannot reach the area because of the poor bridges, they were damaged during the rainy season and no one is coming open to say he/she will fix them, they are waiting for the campaign periods, when they will nail their things (posters, campaign material (not clear)

**All:** cross-talk

**00:** if you had come to that side today, what he is saying, you would have been the first person to say about it because if you come by car, you have gotten out of the car and travel on foot

**Facilitator:** Okay

**07:** most of the time when you get sick, you are well assisted when you go to the hospital than buying drugs from the shop because, at the hospital, they know what exactly you are suffering from

**09:** sometimes when you go to the hospital, and you are explaining to the doctor how you are feeling, they just prescribe the medication without examining you, such things discourage others from going to the hospital, ‘’I have just explained how I am feeling but, they have not examined me to see if what I think is true’’ most people then think, if I explain this to a vendor, ‘’I feel headache, what can I do?’’ ‘’buy panado, it helps’’ so you cannot go to the hospital

**00:** I want to explain what I have noticed about the treatment that we receive when we go to the hospital. For example, you have called us here, and we have come here. When we go to the hospital and are diagnosed with a disease, then you refer us to Queens yet you have no money, you then start thinking, ‘’Why did I come here? They haven’t provided me with transport (not clear) such things discourage us not to going to the hospital. When people come to the hospital to access services, they are told, ‘’According to your condition, go to Queens’’ yet the patient has no means of transport. So you think, ‘’should I go to Queens on foot, let me just stay.’’ You just stay because you did plan for transport

**Facilitator:** Transport

**00:** yes

**Facilitator:** when we arrived, we discussed diarrhea in children, and discussed diarrhea in adults, and the use of herbs. I would like to know, what do you do when the under 5 children have diarrhea?

**05:** when a child has diarrhea, we rush to the hospital because a child who is under 5, cannot speak for him/herself, it is us parents who observe, maybe a child is lethargic or has diarrhea, maybe malaria. We just assume we should take a child to the hospital so that doctors can treat the child

**04:** there is conduct that mothers do to their children when they have diarrhea, just like what number 3 said, they think the child is teething, and some people say just take administer some diarrhea medication, so we administer the drugs to a 6 or 7-month little child, so we just dilute the same drugs to stop diarrhea and so, they think it’s teething yet it’s not

**Facilitator:** what kind of drugs are commonly used?

**04:** mostly is Flagyl

**Facilitator:** mmh, let’s hear from others

**00:** I think for a child to have diarrhea, it’s because of lack of hygiene practice at that household

**Facilitator:** is there anyone here whose child had diarrhea before?

**02:** that’s what I have already said when a child has diarrhoea, parents think it’s teething

**Facilitator:** has it ever happened to you that your child had diarrhea?

**All:** yes

**Facilitator:** everyone should tell me what he does because we should differentiate what we should be doing because you are informed and what you do, so let’s start with what you did when your child had diarrhea.

**05:** what I did do when my child had diarrhea, because of the condition of the child, people did not know that our child had diarrhoea, it was we parents who knew. I was then preparing to go to work and I said to my wife, ‘’Have you seen the child?’’ ‘’yes’’, ‘’ he/she has diarrhoea,’’ that time it was not even a teething period. That time she was trying, that issue of prayers, ‘’aah let me take her/him to a pastor,’’ I asked, ‘’Did you go to the hospital?’’ ‘’Yes, I did.’’ But she consulted a pastor. When the condition worsened, the child died, and after I investigated and noted that she went to a certain pastor I said, ‘’Why did you not tell me?’’ the time you went to a pastor was a time you could have gone to the hospital for him/her to be diagnosed because pastor cannot know the problem for this child, what a pastor can tell you is to lay a hand on a child for prayers. As men, let’s take part in the welfare of a child because we are the ones to look after children, if we don’t do it, we end up losing children’s lives, so we are losing the lives of those who could have helped in the future. Once I have realized a child is sick and I am doing something, I should stop, then I should carry the child on the back and rush to the hospital, the mother will be following me

**Facilitator:** I am sorry for the death of a child, let’s hear from others

**02:** when a child gets sick, I take him/her to the hospital but still, some people don’t go to the hospital because of the beliefs as he has said about the religious beliefs. There are others as well who don’t go to the hospital because they don’t take any medication

**Facilitator:** I am not here to judge or deal with you but, let’s look at what we do. I am not an angel either

**All:** laughing

**Facilitator:** I may be doing what you are doing but, let’s know what you do before taking a child to the hospital

**02:** we take a child to the hospital because that time, the child hasn’t eaten anything

**01:** when my child had diarrhea, I noted that I could not manage to take the child to the hospital, I told my wife to dilute glucose, or they call it thanzi, sugar and salt plus water so that the child should get hydrated that came as a result of diarrhoea. The following morning, I went to the hospital where he/she received treatment and was okay

**Facilitator:** Okay

**09:** what I did, when the child had diarrhea, I told his/her granny, ‘’the child has this condition,’’ the granny then said, this child needs to be given a bark from the mango tree, it helps. Then I told my friend, ‘’my child has diarrhoea and the granny said this and that.’’ The friend then said not to waste time with that because the granny cannot know. He/she is not a medical personnel, take a child to the hospital to be examined and they are the ones who can give you the right treatment. I then told my wife, ‘’don’t waste time here, got to the hospital to get the right treatment’’, and when she went, it worked well

**08:** my child had diarrhea and when we told our friends, they told us to get glycerine from the shop and give the child one spoon to drink. When I heard about it, I consulted my parents that time, and they said no, glycerine feels hot when applied to the skin, should we give it to a child who is not mature, tell your wife to take the child to the hospital? When the child was taken to the hospital, he/she was examined and the doctor said that it was not teething but, the child was removing the wastes from the stomach. The child was given treatment, and in a few days, the condition improved

**07:** when a child has diarrhea, it is good to take him/her to the hospital to be seen by the doctors

**Facilitator:** what do you do on your own? Or what have you been doing or ever done?

**07:** what I did was buy drugs from the shop before taking the child to the hospital

**Facilitator:** was kind of drug did you buy?

**07:** it was this green-green

**All:** laughing

**Facilitator:** I would like to know the kind of drugs children are using, like green-green

**07:** shops have limits on drugs that they can sell

**Facilitator:** are there drugs that they cannot have them?

**07:** they have painkillers only

**Facilitator:** is that true that vendors only have painkillers?

**07:** they have them but illegally and you can observe them

**Facilitator:** ooh

**07:** they first see who wants to buy

**All:** (cross-talk)

**Facilitator:** what kind of drugs do they not sell to any other person they don’t know?

**00:** Bactrim, indomethacin, amoxicillin, and the like, are sold illegally

**Facilitator:**  do you know drugs called antibiotics?

**00:** I think Bactrim and penicillin are included, they are regarded as antibiotics

**Facilitator:** why do you buy such drugs that are not sold to everyone?

**07:** to protect a child

**Facilitator:** we are looking at everyone, child or adult

**07:** to protect the patients before taking them to the hospital, we take them to the hospital

**03:** partly it’s like first aid, ‘*’pafupi mpomwe wafika’’* (you don’t go far when you have an option nearby). As we are thinking of taking a child to the hospital, we want to reduce the problem, so we go to the shop because it where you close to

**09:** drugs that are illegally sold are more powerful, you may buy them according to your budget but, they cannot know the limit (dose), maybe you were supposed to take 1 tablet, but you are taking 3 tablets at once

**All:** (laughing) cross-talk

**00**: vendors don’t know the dosage, for example, if you have malaria, they don’t know how much LA you take or how much Bacrim you take. You can ask, ‘’how much is Bactrim?’’ ‘’K100’’ you buy 2 and you think after taking it, you will be okay. You may relieve the pain but, after some time, the disease resurfaces because you are told the dosage

**Facilitator:** Alright, on what we do when a child has diarrhea, the main thing is that a woman should take a child to the hospital

**All:** laughing-cross-talk

**Facilitator:** do men take any responsibility?

**00:** the responsibility is to encourage a woman to take a child to the hospital so that the child should be treated as we are looking for food

**08:** we do take responsibility, as we have said, there are poor road networks where we come from, and so we ensure we have found means of transport for a woman to get to the hospital

**Facilitator:** why don’t you use those means?

08: during that time, we do things that when she is coming back from the hospital before the child uses the medication, they should eat. Most of the time men are hunters

**Facilitator:** mmh

**08:** That’s why we in most cases send women so that we can handle other things

**Facilitator:** Okay, the discussion will be back and forth so that we follow the issue. As we discussed diarrhea, you talked about what causes it and what we do to deal with it but, on prevention, how do you prevent diarrhea in the community or household?

**05:** as others said, it is all about hygiene because it cannot happen for a household that lacks hygiene, unclean utensils, and eating at that household, which means you are keeping certain diseases in that household. Even the issue of toilets, houseflies can collect waste things while you are eating, which means you are getting diseases, and when diseases are entering, we don’t know, all we know is food. We then realize when the symptoms are shown and we wonder what happens, forgetting that we don’t practice hygiene. So the place where we are eating should be cleaned

**06:** we should avoid drawing water from the wells, we should boil them before we use them, and if we have chlorine, we should use it before using that water, I think we can prevent

**02:** we should have a toilet which has a cover and we should have a garbage pit. We should encourage children to always wash their hands (not clear) in other words, hygiene practices

**00:** we have talked about washing utensils in rivers, it is necessary because we don’t know what’s in that river. Maybe others have defecated in that river, and we use that water for washing plates and before they get dry, we use them. That is one way of getting diseases and we should improve on that

**Facilitator:** we are going toward the end of our discussion and we would like to talk about the vaccine for under-5 children. What do people say here in Bangwe regarding the under-5 vaccines?

**02:** I just don’t know what’s going on in our country because every vaccine that is introduced, people are talking but, for me, I do believe in vaccines because for us to be born, we start with the vaccines, so the vaccine started long ago. I don’t know if it is politics or what but when every vaccine is introduced, people talk badly about it

**05:** recently, 2 vaccines have been introduced if I am not mistaken. I was working somewhere with my fellow men and they were saying, ‘’My child should not receive this vaccine!’’ Those who go to the villages (healthcare workers) can be my witness, they have faced many challenges verbally. Others tell them, ‘’Don’t come here or I will beat you up! Don’t give my child that vaccine.’’

**All:** laughing (cross-talk)

**05:** when they see healthcare workers coming, they hide their children, ‘’come here until they have gone,’’ so we know it’s not good because if they have introduced the under 5 vaccine, they foresee what’s coming tomorrow and the person who does that doesn’t know what will come tomorrow, that’s why he says that my children cannot receive the vaccine. When we heard that we were wanted by the medical personnel who wanted to discuss health issues with us, we had the courage to come so that we could take part in that issue, and when we get back home, we will meet these men and tell them, gentlemen, maybe we speak these out of childishness but, let’s see think about it because for us to grow up, he received the vaccine that time, and that means vaccine started long ago. When health care workers say let’s vaccinate children, ‘’aah no, last month children were vaccinated, this month as well (not clear) if you don’t vaccinate your child when the child will have a problem and go to the hospital when he/she will be examined, they will know that the child did not receive the vaccine, you will blame the health workers because the child hasn’t been treated yet, health workers had been coming to your village for the vaccine. You didn’t even use your transport, they come to your household to vaccinate your child, but you have refused them, ‘they should not come’’ ‘’Okay, you leave like that because you think that you are a doctor on your own’’ that’s what happens

**01:** the issue of vaccines in the communities is a threat. We have grown up, and we hear our parents that there were different vaccines in the past and we had received different vaccines, but now, it is a threat. I heard others say, ‘’vaccine people are coming!’’ (not clear)

**All:** laughing

**01:** it is a threat, someone may have said, ‘’Vaccine people will come to our school tomorrow, don’t go please’’ It is a threat because someone may come with rumours about the vaccine that is not true, such things affect some people in the communities, and they are having some mind-set that vaccine is bad. They say, ‘’Maybe we are overpopulated and they want to reduce us.’’ The issue of vaccines is a threat to the communities

**00:** this came after COVID-19

**0:** yeah

**00:** that’s when the hating of vaccines came in, it was okay in the past, they could go door-by-door, they could just stay at one place and people were going but, but when COVID-19 came, there were a lot of rumours that people believe in, some of them when you hear, you ask yourself, ‘’can this happen?’’

**Facilitator:** mmh, please share such rumours

**00:** rumors say it’s Satanism, some say it’s 666

**0:** some politicians destroy the reputation of fellow politicians (cross-talk)

**00:** even religious people, tell their followers not to receive the vaccine

**01:** that’s why the vaccine is a threat in the communities because of what people are hearing

**Facilitator:** you have said that in the past, people were just receiving the vaccines without any problem and I believe the vaccines that were offered in the past are similar to the present ones

**01:** they are the same

**Facilitator:** what makes people to be suspicious about this vaccine during COVID-19?

**01:** the issue is, just like the polio vaccine he talked about, two doses of the vaccine have been offered, and two doses are remaining to finish

**Facilitator:** mmh

**01:** If the vaccine is offered today, and health workers go to that community tomorrow, people were asking, ‘’A child had received the vaccine yesterday, should he/she receive another one today? What government want to do to us?’’

**All:** laughing

**01:** some say, they want to reduce the population or they want us not to give birth

**0:** Malawians are over-populated, so you should be reduced

**00:** during COVID-19, there was mandatory vaccination, and everyone had to receive the vaccine by compulsory, that time people had questions, ‘’you don’t want to get vaccinated, should you do it because you are forced to do so? Why compulsory?’’ some people were receiving money, and some didn’t, but the same vaccine and you wonder, what do they want (cross-talk)

**0:** it’s questionable because IHV was discovered long ago, why COVID-19 vaccine was available instantly other than HIV which was discovered long ago? There is a lot that people think about and for them to get to normal, they should be civically educated

**09:** on the issue of COVID-19, between those who were vaccinated and those who did not, it was those who were vaccinated that were contracting COVID-19, and people were surprised, why do you have the virus yet you received the vaccine? This one did not receive the vaccine but is okay, should we receive the vaccine still?’’ no, let us stay

**00:** that’s why there was a rumour that this is not a vaccine, they want to give us a virus in order to reduce the population here in Malawi

**04:** I want to speak about this vaccine. In the past, the vaccine had no problems. There was a polio vaccine and measles vaccine and people were receiving the vaccine without any worry, but when it comes to the COVID vaccine, there was Pfizer one, another was Johnson-Johnson, and the like. Another type said people should receive it once, and another one said people should receive it twice, and later, it was communicated that everyone could receive how they want, people were worried because of such things. COVID-19 vaccine, we heard that some people died after they had received the vaccine, such people were afraid to receive such a vaccine. For vaccines such as measles and polio, mothers followed up from the time a child was born up to the time a child finishes all the vaccines, and now, someone comes with another vaccine

**Facilitator:** the vaccine that they finished receiving it

**04:** yes, that’s what discourages women, they say we finished all the vaccines and now, we are told to go with children to receive the vaccine as well. For example, some children received the vaccine at school and followed the procedure, but they were told to go back to school for another vaccine, just like the diarrhea vaccine, as number 3 said, there were different vaccines but, the vaccines had no problem but, because the vaccines are now coming, people get vaccinated and go, they get vaccinated and go, because of that, parent and guardians are not accepting their children to receive the vaccine because it has threats. Previously, there was no vaccine for diarrhea but now, the vaccine has been introduced, so people are afraid, they say diarrhea vaccine as well, they are afraid and hence, they don’t allow their children to receive the vaccine

**Facilitator:** what are peoples’ fears of the diarrhea vaccine?

**02:** as number 3 said already, peoples’ fear started with the COVID-19 vaccine and all these come from there. If it were not that things, people would have been receiving the vaccines but COVID disrupted all relationships

**Facilitator:** what else do people say about the diarrhea vaccine apart from what he said?

**02:** on top of the diarrhea vaccine, all under-5 vaccines that are introduced nowadays, people are saying they want this generation to be castrated so that it should not overpopulated

**All:** laughing (cross-talk)

**00:** just like what happened with circumcision, people said the aim is to reduce the population, people were asking, ‘’What are you going to do with the foreskins (cross-talk)

**Facilitator:** let us get back to the issue this was talked about, he said when the healthcare workers go to the communities, you tell children to go and hide. What do we say when women take little children to the hospital where the vaccines are offered?

**02:** the vaccines children receive at birth, those vaccines are followed, even the vaccines that are received at under-5 clinics, they are okay with that but, not this one for door-to-door

**All:** (laughing)

**02:** even the polio vaccine, if it is received from the clinic, is okay, you will never hear anyone talking

**00:** but, if they take the vaccine to their homes, they think a lot

**Facilitator:** people have problems with door-to-door vaccine

**00:** as I have said, for example, today is an under-5 clinic day, whether it is an outreach or static, the health care workers then come the following day and tell people, ‘’we offering polio vaccine’’ Then someone says, ‘’my child received the vaccine yesterday.’’ And the health care workers say, ‘’We are giving the vaccine to those who received the vaccine yesterday’’ so people get surprised and ask, what is happening because they know vaccines start from this point up to this point, ‘’What about this one?’’ For them to understand it, it's difficult, so they ask why the government giving the vaccines to every child in the community, there is misunderstanding there and that’s why, this one said maybe they want to reduce population, it’s Satanism or they want us not give birth

**Facilitator:** Alright let’s get back a little bit. This one said the vaccines are good despite all these, that’s what number 2 said

**02:** maybe……

**Facilitator:** let’s talk about advantages of the vaccines

**02:** the advantage of the vaccines is that the researchers foresee something ahead, they want to protect before that disease arrives. I believe that if the government wants to harm us, it can do so because there are many ways that we don’t know….

**00:** (these women who hide their children, you see them asking for family planning if the government wants to use family planning methods, is it not going to be done

**02:** it can happen

**All:** laughing

**Facilitator:** I understand you, the government might conduct research and the vaccine cannot harm us but, as humans, we have a right to think what we can, those who are thinking that way, we are not judging them but as parents, we have children who received the vaccines, have you seen the benefits of receiving the vaccines?

**04:** vaccines have benefits, my children have been receiving the vaccines since birth, like polio and measles vaccines, so vaccines have benefits because children don’t get sick when the disease comes, that’s the benefits I noticed when the children get vaccinated. Even with this rotavirus vaccine, if my child gets vaccinated, the child is protected from diarrhea in so doing, I have saved the money that I could use to take my child to a private clinic or hire a bike to the hospital, that’s the benefits of getting the child vaccinated

**Facilitator:** let’s hear from others, we going towards the end

**09:** sometimes because of ignorance, you don’t differentiate whether the vaccine has worked or not because the child does not get sick or he/she has no infection, so you think maybe the child received the vaccine for the sake of getting vaccinated because the child never gets sick, the vaccine should protect the child before getting sick but, I believe if a child gets vaccinated when he/she already gets sick, most parents would have known the benefits of the vaccine but, they just take it for granted and maybe think the disease doesn’t exist

**08:** most people ask why they are coming to vaccinate me as if I went to the hospital or invited them. I am not sick, but they are coming to vaccinate me, what’s wrong with this vaccine, is it good or bad, that’s why people hide their children, thinking that maybe they want me to suffer, as you know when a child receives the vaccine, he cries at night, so they say they want me just to suffer, but my child is not sick

**Facilitator:** mmh

**08:** just like what he has said maybe we will realize when the child gets sick and gets vaccinated, that’s when we will know the importance

**Facilitator:** he has raised a good issue of what children experience after receiving the vaccine like crying at night, is there anything that we observe when the children receive the vaccine?

**00:** the vaccines protect children from death. When the children receive the vaccines, you protect them from death

**Facilitator:** when the children receive the vaccine whether from a clinic or right from the village, he said children cry at night, I am asking if there is anything else apart from crying.

**09:** a child can receive a vaccine and the vaccinated area may get swollen (not clear) but it cannot go on, it’s just for that moment

**07:** I can say the vaccine is important because it protects from illness before one gets sick. When someone is seriously sick and receives the treatment, it does not function properly

**Facilitator:** Okay, let’s finish with this question. For people to receive the vaccine amidst all these rumours, what can be done?

**02:** people should be sensitized to the importance of receiving the vaccine and ignore the rumours. Once someone hears something, especially men at the working sites, ‘’gentlemen, what do you think of an injectable family planning method?’’ ‘’it reduces manpower’’

**All:** laughing

**02:** ‘’What do you think is happening on the ground?’’ someone will take that, and tell someone (cross-talk)

**Facilitator:** This is mostly mentioned, how do you connect issues of manpower and….

**02:** (not clear) They say it reduces manpower. There is a need for civic education, we should not accept without investigating (cross-talk) we should be asking, ‘’How do you get these issues?’’

**00:** the issue is, before a clinic, a health talk should be done covering such things, as civic education to people, ‘’Ladies and gentlemen, our responsibility is to protect people and we cannot come here to kill people.’’ Maybe with time, people will understand because, amidst all these, there are some who rush to these things

**Facilitator:** Okay, is there anything to add?

**All:** Silent

**Facilitator:** This is what I prepared for us to discuss, there can be something that is important, this is your chance to comment before we finish

**02:** the issue is what I have said, people should be well addressed. If the government wanted to do something for its people, it could do so without anyone knowing it, just like what others said about injectable family planning, the government can use such ways. These (vaccines) are aimed at protecting the citizens

**Facilitator:** Okay

**02:** people should know these things or if we avoid all these, if the government wants to do it, it can do it but, every vaccine is good

**01:** On that issue of civic education, people should be taught what the vaccine is and they should know the meaning of the vaccine, most things will be avoided, and people will be able to say let me not follow these are mere rumours, let’s get vaccinated

**Facilitator:** Thank you very much, this max the end of our discussion I think you for your attention, thank you.

**All:** thank you.

**END OF DISCUSSION**
