# Supplementary material for: Exploration of the social determinants of diarrhoea, rotavirus vaccine uptake, and vaccine ‘fatigue’ in Ethiopia, Kenya, and Malawi
Source: PLoS One. 2025 Sep 9;20(9):e0319691. doi: 10.1371/journal.pone.0319691 (PMC12419581; doi:10.1371/journal.pone.0319691)
Supplement: S1 Data — (ZIP) [file pone.0319691.s001.zip › Supporting Information Files/KY_10FGD.docx]

**FOCUS GROUP DISCUSSION 10**


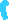


**15/03/20204**

**NUMBER OF RESPONDENTS-( 8 MALES,2FEMALES)**

**1. Can you please tell us some of the illnesses that affect children in your community?**

**R1-**You might find a child has diarrhoea, is vomiting or has measles.

**R2-**Children suffer from fever, they are unable to eat sometimes and diarrhoea and common flu.

**R3-**Fever, vomiting, high levels of body fever and diarrhoea.

**R4-**A child might have rashes on their body. This mostly occurs when there is a lot if heat and the rashes may appear on the neck, face and even sometimes on their rear ends.

**R5-**My child’s stomach swelled and became stiff, their breath became smelly and thereafter they started diarrhoea.

**2. Which of these illnesses do you consider to be a burden in this community? Why do you say**

**so?**

**R5-**Diarrhoea.

**R2-**Fever.A child’s body becomes hot all over and they struggle to breath. This causes you to tense because you do not know what the cause of that fever is and at times when you take them to the hospital to be tested you are told that they do not have malaria. What causes this?

**R6**-Coughing.My child has been coughing for like a week now. I have taken them to the hospital and they have been given medicine but they still cough for about an hour in the morning when they wake up before they cool down.

**R7-**Sometimes when a child has diarrhoea, they cough a lot until they vomit.

**3. If you were to rank these illnesses in order of priority, what would you rate as the top three**

**diseases affecting children**

**[If diarrhoea is not listed in the top three priority problems -Do you think diarrhoea diseases**

**are a big problem in this community or not? Why do you say so?]**

**R1**-I would say that the first one is diarrhoea because most of the times we do not understand what exactly is the cause. You might find that your child is three to four months and has diarrhoea. This child is very young and has not yet started eating or even crawling so you fail to understand what might be the cause of the diarrhoea because most of the times it is said to have been caused by eating dirty things. This causes us a lot of worry because the child’s progress and milestones start regressing. The second illness is vomiting because when you feed a child something they immediately vomit it out and the third is coughing. Our children cough a lot and even after treating it the cough is recurrent.

**R8-**Sometimess a child’s a lot of fever and when you take them to the hospital you are told that they don’t have malaria or any illness. That baffles me a lot because I am unable to understand what is happening to the child. The second illness that affects children is vomiting and diarrhoea during teething and when they start crawling. The third illness is lot of coughing which happens mostly with young children.

**R4**-During the early stages of growth, if a child has worms in their stomach, they tend to eat sand. This is very challenging because it causes diarrhoea. The second illness is body temperature changes and fever, and the third is sores that heal and then leave a spot and start forming once again on the same place as the previous sore.

**4. Can you tell me the health services/facilities available in this community? Where do you**

**access health services? [Probe: how much does it cost to access these services, how long do**

**people have to travel to access the services?**

**R9-**There is Ruben Center and Maendeleo Hospital. I go to Maendeleo which is free for treatments. The only thing that you pay for is a clinical book or sometimes if the medicines are unavailable there then you get prescriptions to buy them from outside. It is not far from where I live so I go there by foot.

**R4-**I can access two hospitals at any time which are Ruben Center and Maendeleo. I like going to Maendeleo because the treatments there are free except times when there are no medicines and you have to buy them from outside. I access it by foot because it is near my home.

**R10-**There are two hospitals which are Ruben Center and Maendeleo. I prefer Maendeleo because their services are good and if there are no medicines you get a prescription to buy them from outside. I walk in order there because it is close to my home.

**R5-**There is Mukuru Health Center or Our Lady of Nazarene. I go to both and I am served well at both. I have never been charged for treatments there and I walk to get there.

**R3-**I live in Njenga and we have two hospitals; Mukuru Health center and Our lady of Nazarene. I go to Mukuru Health care because it is close by and the treatment is free there. The only thing you will buy is a hospital booklet and I walk to get there.

**R4**-I go to MMM Mukuru kwa Njenga. I like going there because when I get there I am served and do not pay for treatment. I just go with my hospital booklet and am not charged anything apart from twenty shillings to pay for a receipt.

**R2**-I live in Ruben and there are two hospitals here, Maendeleo and Ruben Center. I mostly go to Ruben Center because it is well equipped and has everything that I need. There is a lab and medicines are available there even though I have to pay for it. I go there by boda-boda.

**5. How do most people respond when a child has diarrhoea in the home? [Probe: What do**

**people do at household level? at community level? Where do they go to access treatment? Do**

**they take antibiotics? Where do they access antibiotics? Why do they access antibiotics?]**

**HOUSEHOLD LEVEL**

**R6-**The first aid that I would do is give the child zinc and ORS to stop the diarrhoea and then I would take the child to the hospital for further tests.

**R1-**The first thing I would do is take the child to the hospital to be diagnosed and directed by the doctor on which medicines that I should give the child.

**R7-**I used to go to the chemist and ask for medicines that would stop the diarrhoea and sometimes the diarrhoea would still continue. I later came to learn that I should take the child to the hospital to be tested in order for the cause of diarrhoea to be identified.

**R2-**The first thing that I would do if I noticed that my child had diarrhoea was take them to the hospital instead of giving them medicines. There, they would be tested for the doctor to guide me on which medicines to buy for the child.

**R5-**When my child has diarrhoea, I make sure that I give them a lot of water because it mostly happens at night. Then, in the morning I take them to the hospital.

**R3-**When my child has diarrhoea and I am unable to take them to the hospital, I boil some water and add some sugar and salt to it. I then let it cool down and give it to the child. After that, I take the child to the hospital.

**COMMUNITY LEVEL.**

**R6-**There are some people who give their children water mixed with a bit of salt and sugar. There are also others who give the child ORS or Zinc depending on their knowledge an ability. They buy the two from a chemist.

**R8**-There are some mothers who crush sodamint (Sodamint is an antacid used to treat heart burn) or ashton (Ashton is a teething gel for infants) thinking that it is medicine for the teeth and give the child, others rush the child to the hospital while others give the child boiled water mixed with salt and sugar.

**R3-**In our community we mostly believe that diarrhoea is caused by plastic teeth in children. When they get diarrhoea, we take them to a man in our community who rubs the teeth of the child and it stops. Old women and mothers in the community believe that you cannot give the child to someone else to hold them after this because they might die.

**R9-**Many people in the community run to the chemist or look for someone with ORS and give the child.

**R7-**There are others who give the child dry bread to dry up the diarrhoea and there is one person who told me to mix flour with water and give the child.

**R6-**There are others who mix millet flour with some cassava, cook porridge with it and give it to the child. This hardens the stool and stops it from having the consistency of diarrhoea.

**WHERE DO YOU GET ANTIBIOTICS?**

**R4-**When I need the antibiotics I go buy them from the chemist. There are times when you feel unwell and that you need the antibiotics, that is when I go and buy them from the chemist.

**R2**-I always have antibiotics at home. I never lack Amoxil or paracetamol because of my small children. For instance, I give my child Calpol if they have fever before taking them to the hospital.

**R8-**I mostly get my child’s medicine from the hospital, I never buy them from the chemist because of fear. I know of a parent who bought their child medicine from the chemist when they had diarrhoea. The medicine did not help the child and the child ended up dying.

**R3-**Most of the times I go to the hospital because you might go and buy medicines from the chemist and they have expired, this cannot help me.

**R7-**My neighbor has ever borrowed antibiotics from me because their child was given some vaccination and was crying a lot. As neighbors we felt affected and I decided to help the mother with Calpol which helped to sooth the child and thereafter the child slept.

**6. Can you tell me some of the enablers and challenges that people experience to access**

**treatment for diarrhoea diseases?**

**CHALLENGES**

**R1-**There are dirty sewers in our community and our children play outside and this might expose them to the dirty sewers. We ensure that the children wash their hands after playing and that we boil their drinking water.

**R3-**The first challenge is that there are times when you take the child to the hospital, get tested and prescribed some medications to buy from outside when you do not have money. You might delay getting those medicines as you wait for maybe your husband to come home and give you the money to buy those medicines.

**R6**-You might go to the hospital and find a long que there and maybe the child is getting worse, this might compel you to go and look for services from private hospitals. The ques are long at the public hospitals because the doctors there are experienced and will prescribe you medicines that will help your child.

**R4-**A child might continue suffering as you look for money to cater for their treatment because of financial constraints.

**R8-**Sometimes the medicines at the hospitals are not there and you have to buy them from outside when you do not have the money to do that.

**R7**-There are times when maybe your child does not have oxygen so you are sent to some where like Mama Lucy Hospital as an emergency and you do not have the fare for transport. There are services that are not there in our hospitals.

**R2-**The long ques are challenging because a child might be in pain and some doctors are not understanding and harass you. This demoralizes you and you might be having an emergency.

**R7-**The doctors at the hospital are inconsiderate and take long breaks during their lunch hours. They might not even be eating but making stories while your child is suffering.

**R5-**There was a day when my child was sick and I was far. By the time I got to the hospital it was about half past one. I gave out the booklet and was queued. When I reached the consultation room, the doctors told me they were closed and that I was the last and I had to come back again. We wonder what the point of public hospital is when we are getting this kind if treatment from them while we are running away from private hospitals because of incompetency.

**ENABLERS**

**R3-**CHVs in our community go around giving vaccinations to children when they suspect cholera outbreaks.

**R5-**There was a time when we were given some water buckets at Mukuru which we filled with water and placed them on a stool near the toilets so that it becomes easy for a child to wash their hands after using the toilet.

**R6-**There is away that the hospital gives you first aid before referring you to another hospital

**R7-**If you rush your child to the hospital during emergencies they test your child before giving your child any medicine as opposed to the chemist where they will just guess.

**R10-**There are CHVs who give you a referral letter to go get your child treated for free when you do not have money.

**R5**-The doctors at the government hospitals explain and change medications that you got from the private hospitals and were not helping you to ones that will help you.

**7. What do people do to prevent diarrhoea? [At household level, at community level?]**

**R10-**I make sure that the food my children consume is safe by doing things like washing hands before cooking.

**R5**-I ensure that the environment we live in is clean and that we always wash our hands after visiting the toilet.

**R6-**I make sure that I wash their hands and fruits that I buy before the child eats them.

**R8-**I make sure that when I buy water I always treat it first. There are some water treatment tablets that we are given at Mukuru Hospital and I make sure we treat our water before drinking it.

**R3-**I always boil our drinking water and make sure that I wash my hands if they are dirty before I touch my child. I also wash my breast before breastfeeding my child.

**R7-**I ensure that I practice cleanliness at home and give my child a balanced diet. I also ensure that I wash my hands before leaving the toilet, that I wash fruits before eating and ensure cleanliness during the cooking process.

**R9**-I make sure that I am clean before handling a child and that the environment is always clean.

**R2-**As a mother if you are breastfeeding you ensure that you wash your breast before breastfeeding.

**R8-**We make sure that our living surroundings are clean because there are many children playing outside.

**R6-**Improving on hygiene by doing things like washing your child’s toys, washing your breast before breastfeeding a child and washing your hands after visiting the toilet. You should also avoid buying a child street food because you do not know where or how the food was prepared. As the mother of the child, you should be the cleanest in order for you take care for your child.

**8. How do people in this community perceive childhood vaccines [Probe: why do you think**

**childhood vaccines are widely accepted? Why do you think childhood vaccines are widely**

**resisted?**

**R5-**In our plot, I have not seen any mother who does not get their child vaccinated but there are many who refuse the vaccines from the mobilizers and people who go around in the community giving out vaccinations. There was one person who told me that the reason they refused their child to receive the recent polio vaccine was that it was a coverup for family planning.

**R3-**A lot of people are contemptuous about the vaccines that are taken around in the community and prefer going to the hospital even though a lot of the times these vaccines help us.

**R1-**The first thing for me is that I like vaccines because they protect a child from many illnesses.

**R4-**Where I live the people like vaccines and are happy when the CHVs bring vaccines to our community because they help people who are unable to access these vaccines because of time as the CHVs bring the vaccines to you.

**R9-**The men in our community are accepting toward vaccines.

**R1-**In our area people accept vaccines because they are important for young children and protect them from diseases.

**R6**-I know the polio and measles vaccine.

**R10**-I know the polio vaccine.

**R7**-I know the polio, measles and rotavirus vaccines.

**9. How about rotavirus vaccines? What do people think about rotavirus vaccines? Where do**

**they access rotavirus vaccine? [Probe: What do they think are the benefits of rotavirus**

**vaccines? What concerns do people have with rotavirus vaccines?**

**R4-**The rotavirus vaccine protects children from illnesses but I do not know which.

*Respondents do not know what vaccines are for, their names or benefits as they do not ask the doctors or healthcare staff what they are for. They mostly just trust the doctors *

**10. What are the enablers and challenges for people in this community to access rotavirus**

**vaccines? [Prove: cost, distance to access services, cultural/religious beliefs, impact of**

**COVID-19, perception of vaccine safety]**

**R7-**I have never experienced challenges in accessing vaccines. I just take the child to be vaccinated and go back home.

**CHALLENGES.**

**R8-**My friend’s child was vaccinated and when they got home they started reacting to the vaccine by swelling. When the child was taken back to the hospital, they were given some medicine to apply. This resulted to a sore and the child had to be taken to Kenyatta Hospital.

**R5-**The challenge is that as mothers we know that the child will cry during vaccination so we give them some medicine to cool that down before and after being vaccinated without the doctor’s knowledge.

**R7-**Another challenge is that sometimes you are told that the vaccine that your child is supposed to be vaccinated on that day will not be opened on that day and so you have to come another day.

**R6-**IThere are some single mothers who are employed and take their children to day care. They are unable to take their children to be vaccinated because they are employed and have a lot of responsibilities.

**R6-**Reluctance of our male partners in helping out when getting our children vaccinated is a challenge because this is overwhelming for one parent to carry the burden alone. They are culturally indoctrinated in thinking that they are only supposed to provide for a family and nothing else.

**ENABLERS**

**R5**-It is easy to access the hospitals and they are free.

**R7-**The attitudes of care givers varies from one to another. There are some who are welcoming and good and others who are rude.
