# Supplementary material for: Exploration of the social determinants of diarrhoea, rotavirus vaccine uptake, and vaccine ‘fatigue’ in Ethiopia, Kenya, and Malawi
Source: PLoS One. 2025 Sep 9;20(9):e0319691. doi: 10.1371/journal.pone.0319691 (PMC12419581; doi:10.1371/journal.pone.0319691)
Supplement: S1 Data — (ZIP) [file pone.0319691.s001.zip › Supporting Information Files/KY_09FGD.docx]

**FOCUS GROUP DISCUSSION 9. MUKURU**

**9 PARTICIPANTS, 5 FEMALE& 4 MALES**

1.Can you please tell us some of the illnesses that affect children in your community?

*R 1 - We are affected by diarrhoea here in our location since we have open ditches and we buy/eat street food that is not properly cooked and cleaned.
R 2 - We are affected by malaria.
R 3 - We are affected by the common flu, coughing, and diarrhoea.
R 4 - We are affected by diarrhoea due to the vegetables we buy from the market, where hygiene practices are poor.
R 4 - We are affected by skin rashes in our community since we have open sewage, resulting in children falling inside*

2. Which of these illnesses do you consider to be a burden in this community? Why do you say

R3**-**Cholera and diarrhoea are the diseases that are most burdensome in this community. This is because when a child has diarrhoea, they become weak and dehydrated very quickly. As a parent, if you don’t have any knowledge on helping the child then there is a high chance of you losing them.

3. If you were to rank these illnesses in order of priority, what would you rate as the top three diseases affecting children.

*R 1 - Diarrhea and stomach ache
R 5 - Stomach ache
R 6 - Common flu and diarrhea*

4.How does the mentioned diseases affects one/you.

R 1-It's costly in treatment.
R 3-The disease affects us psychologically in an event when we don't have money to seek treatment.
R 5-I am affected since I stopped going to work to attend to a sick child at home.

4.Importance of Policies on health

*R 3-Policies contribute to enhancing the quality of healthcare services by establishing standards and guidelines.
R 4-Policies help in formulating and implementing strategies for preventing and controlling diseases. This includes vaccination programs and health education.
R 7-They set the direction for healthcare delivery, ensuring that efforts are focused on key areas and priorities*.

5.Challenges encountered going in accessing service at the public health facility

*R 3- Lack of drugs, hence referring you to private pharmacies outside.
R 5- Long queues and poor customer service.
R 6- We have a lot of congestion in the public health facility.
R 7- Public facilities do not have enough laboratory equipment, hence referring to private facilities which are too expensive*.

6. Can you tell me the health services/facilities available in this community? Where do you access health services.

**Health facilities available**
R4-We have mukuru kwa ruben.
R 3-We have maendeleo.
R 2-We have private facilities
**Services available.**

R 8 Vaccination.

R 7 Laboratory test

7.How do most people respond when a child has diarrhoea in the home? [Probe: What do people do at household level? at community level? Where do they go to access treatment? Do they take antibiotics? Where do they access antibiotics? Why do they access antibiotics?]

*R 3- When the child is sick, I take him to the child.
R 3- I warm water and add salt and sugar for the child to drink, but if it persists, I take him to the hospital.
R 5- I go to the chemist and buy ORS over the counter*.

**6. Can you tell me some of the enablers and challenges that people experience to access treatment for diarrhoea diseases?**

**Enablers
R1-**Government initiatives and policies that focus on improving healthcare infrastructure and accessibilit**y
R6-**strong community network that encourages seeking healthcare and supports affected individuals
R7-We are well-informed about the importance of seeking treatment early.
**Challenges
R 3-**Inadequate awareness and education about diarrheal diseases contribute to delayed treatment.
R 5- Many individuals, particularly in our community, may hesitate to seek treatment due to concerns about the cost of medical care and medications.

**Rota virus vaccine and other childhood vaccines**

R4-I don’t know the vaccines given to the child, I always give the doctor the child card and the kid gets the vaccine.
R 2-I accept the rotavirus since I have seen how it work on other people and after been told the side effects of the vaccine.
R 7-I won’t just accept the vaccines since i don’t know how it works.

*R 8* In our community most parents who have children usually get their children vaccinated because the Government instructs them to do so
