# Supplementary material for: Exploration of the social determinants of diarrhoea, rotavirus vaccine uptake, and vaccine ‘fatigue’ in Ethiopia, Kenya, and Malawi
Source: PLoS One. 2025 Sep 9;20(9):e0319691. doi: 10.1371/journal.pone.0319691 (PMC12419581; doi:10.1371/journal.pone.0319691)
Supplement: S1 Data — (ZIP) [file pone.0319691.s001.zip › Supporting Information Files/ET_1FGD.docx]

I: Thank you for participating in the interview. You may answer my question voluntarily. What are the diseases that occur frequently on children living here?

P1: Most frequently occurring diseases on children around our area are Flu [common cold] and Diarrhea.

I: Is there any different answer, are there any other diseases occurring in your area, on children?

P6: I usually observe headache and stomachache.

I: Any other diseases?

P4: I observe Vomiting; I think it is because the children living here are vulnerable of Vomiting, because of the environment here. There have also been bacterial diseases.

P2: I have observed that, there has been acute vomiting and diarrhea on children and it usually turns out to be typhoid, typhus and other diseases like these.

I: You have mentioned diseases like flu, diarrhea, vomiting, and typhus. Which of these diseases is very burden?

P3: It is diarrhea and fever, and they usually prescribed ORS and syrup when they come for treatment.

I: Are there any other concerning diseases than those?

P3: The Vomiting is also very concerning and doesn’t give you the time to react.

I: Why do you think it is concerning?

P3: I think it is because our community had low level of living standard and there is low personal and public hygiene status and we live in compact residential places. Our sanitary system is poor and it made them to be vulnerable for diseases like TB.

I: Any other reason to label them as concerning?

P4: On addition to what has been said, the cause for that is because our village in this Kirkos Sub City is full of inhabitants with low standards of living. Our way of keeping personal hygiene is poor and that is because the people living there are poor. The children may feed something from the garbage, so the major cause is low living standard and personal hygiene.

I: What can be the consequence on the children?

P5: Based on my observations, the children pass their day with animals like dog and cats. They might be affected with disease caused and transmitted by animals. That can bring different diseases on them and their families.

P3: That can brought death on children. That is the biggest lose it can brought. In addition, it can cause them to severe diseases.

P6: There has been two days of weeks for the sanitary workers to collect garbage from home according to the governmental program, so the children can be vulnerable for diseases as they have high chance of getting in contact with the dirt. In addition to that, there is a bad drainage system that is polluting rivers across Tekle Haimamot to Immigration road and around Teklehaimanot hospital. In summertime, there is a flood that brings this dirt to the city, and the children get affected with it.

I: What else?

*All participants:* Silence.

I: From the diseases you have listed, please list as per their level of concerning, from one to three?

P6: Especially for children less than the age of 5, it is first Diarrhea. The second is bacterial diseases, and it is flu on the third.

I: Any other?

P2: The Diarrhea and Vomit are the first and second respectively. There may also be fever, but it is diarrhea and vomit as first and second most concerning.

I: Any other?

P1: Most of the time, the TB is dangerous as it can lead them to death as we usually ignorance, thinking it is just a flu or Tonsillitis. The second is diarrhea and vomiting because it lowers their resistance to diseases. I think these are the first and second dangerous diseases.

I: Very good.

P3: There is also food contamination and water contamination.

I: So, on which rank should we put that?

P3: Third or fourth.

I: How about the diseases on first - and second-ranked diseases?

P2: It is food contamination.

I: We put that third. How about the first and second?

P3: It is diarrhea and fever, respectively. Those diseases are common among children here.

I: What kind of health institution do the communities prefer to visit when they are sick?

P1: They usually go to health centers. The community prefers to go to health centers, especially for children. People with better financial ability may use others.

I: What other options of health institutions are there in this area?

P2: There are medium level clinics. There are also private, high-level hospitals, but our community can usually use health centers. Most of the time, children with low living standards are vulnerable so the people with high living standards are not that much vulnerable. Most people prefer health centers anyway.

P5: In addition to the health center, the people also visit hospitals when things get serious. People may get referred to Zewditu hospital, which is under Kirkos sub-city. It is because the health center and the hospital are interrelated governmental institutions where the community can find services easier.

I: They went there when they referred to there [at Zewdtu hospital]?

P5: Yes.

I: For serious cases?

P6: Yes.

I: What are the other health institutions for the community to be served other than health centers?

P2: Other than health center, the person with better financial ability may bring their children to private health institutions to get better medicine. Most of the time, there is no medicine in health centers. Sometimes, the hospitals may refer to other private hospitals for better treatments as they are interrelated.

P6: In addition to health and governmental hospitals, there are two hospitals supported by NGOs. The first is Zawi in kebele 25 and at Feres Meda; their service are given for fair prices. We can get laboratory services for only 700 or 600 birr. They are supported by NGOs.

I: What are the situations in Teklehaimanot?

P2: Here in Teklehaimanot Health center, there may be a referral system to Ras Desta and Tikur Ambessa hospital, Yekatit Asra hulet, Menilik hospital, or Zewditu. There is also a private high-level clinic in Teklehaimanot. There is also Cecilia. In addition to that, the people use health insurance services provided by the government and that has created the opportunity for the people to use services including inpatient services.

I: Where do the communities usually prefer to go when they get sick?

P1: It is usually health centers.

I: How is the distance between the health posts from residential areas and how much do they have to pay?

P3: As Kirkos Health Center, most of the villages are around the center of the wereda, so they only take fifteen or twenty minutes. They only pay 30 birr to registration card fee, by savings. They may need to lay for laboratory and other services; the price is very low though, when compared with private institutions. There is free service for people with disabilities and autistic children with the sponsor of NGOs. The health center also provides services for children in critical cases.

P1: People in critical cases can also use ride service, and sometimes ambulance to get to facilities faster.

P5: The health centers are best for children, in my observation and others opinion. The Asra Simint Health Center is a very good health center with laboratory standard that equals a hospital. The health workers are also good there.

I: Is there any different opinion here in Teklehaimanot?

P2: We, as Teklehaimanot health center, we work for 24 hours. The health center is closer to residential area and people are not complaining about that as they can also use ambulances to take them to Tikur Ambessa hospital in case of emergency.

I: What measures do you take at home when the children living in your area get diarrhea?

P3: I have children, when they get fever, I advise my neighbor working at health center and take them to health center even it is night. I have health insurance card and I get services and get treated with syrup and other medications.

I: How about you, the others? What do you do before taking them to hospital?

P5: Mothers may try to treat them before going to hospitals. It can be, by treating with water-salt solution, water-sugar solution and like that. They may feed them a spoon with sugar solution.

I: What else?

P4: As Mr. Habte mentioned, we may give them Asprin or something like Vitamin Complex. They treat them by giving quarter of the tablet with solution of water. Sometimes, they also treat it with Tena Adam or Bahir Zaf traditionally, [Traditional leafs uses for treatment] if they think it is ‘Mich’ [Which is called Herps Smplex virus]

I: How about for the diarrhea?

P2: They do the same; they also treat them by feeding them with Rice-water, after boiling the rice with water.

I: What else? You can also mention treatments other than traditional.

P5: We also feed them with honey and mineral water to treat diarrhea. In addition to that, we put butter on their head and neck when they get high fever, which gives relief.

I: You have been mentioning that you give a quarter of a tablet, where do you get the medicines from,

P1: We get the medicine from the health centers.

I: You get them from the health institutions?

P1: Yes, we take that from the health institutions. We take that according to the prescription.

I: Very good. You also have told me you take traditional treatments, right?

P3: Yes.

I: Who decided on this, and who brought this idea up?

P5: I don’t know their source of information, but it is usually mothers who brought such kinds of solutions like taking a spoon of salt-water solutions to treat the children with diarrhea.

P4: In the case of diarrhea, children are given a known dose of ORS and sometimes injection through anus. It is advisable to get services from health centers as the traditional health treatments are not that much effective and have been the cause for death of children with diseases like measles, which could be treated well at health institutions. There is a tendency to use Ashes on the head to treat the Tonsillitis traditionally. So, we need to take those traditional medicines in a way that don’t contradict the modern medical approaches. I think, as my father here said, the ORS has the content of salt and sugar, and they feed sugar and salt because of this.

I: Very good, any other opinion?

P6: It is all the same.

I: Why do you think people take the medicine like Antibiotics by them selves?

P3: I think it happened when they are prescribed once, and they take that medicine when they observe the same symptoms another time. That is because people don’t know they need to get diagnosed before any prescription.

I: Do you have any other opinions?

P2: Family may get worried and urge to give medicine, thinking their children may die before treatment.

I: Okay, any other opinion.

P5: I know a child who has died because of that improper taking of the Amoxicillin tablet. They use over and over after they have prescribed once. Families should take care of it as the medicine is not given the same for all age groups. There is type 5, 20, and 25 of the medicine. There is concerning issue about this in countryside; they sometimes put the children under blanket and let them under the smoke of sugar poured coal, thinking the disease will be healed after the cause get out of their body, in the form of sweat.

I: Thank you. You have been giving us good intentions. What challenges can there be for children to take medical treatments for diarrhea?

P2: I think the parents don’t usually want to go to health institutions. They tend to think they can’t get the services at night or they fail to take the child to health institutions because of ignorance, and this leads to death or physical or mental disability of their children.

I: What other obstacles or good opportunities are there to take medical treatments at health institutions?

P3: The obstacles are the miss- information from neighbors. They inform the community, and the health center doesn’t treat at all and the traditional way is better. In addition to that, the health workers are not that much collaborative when come for service. When you want urgent services like, for diarrhea or vomiting, they sometimes tell you to have a registry card first before service, and you may not get the worker who shall give the registration card. These are the obstacles in Health center.

P4: On addition to that, there is also financial inability to use the health service at health center and that made them to use the traditional services or not to use the service at all. The second challenge is the ignorance of the family. I personally prefer to use the services at health institutions.

I: Okay, you can also tell us the good opportunities that enable people to go to health services at health institutions.

P1: My brother here has mentioned something good. Sometimes, there is a tendency to treat the child at home; there are some people creating these problems. We take them to health center or to hospitals if they are referred to.

I: What are the situations that create good opportunities for people to get health services at health institutions?

P4: We have observed that families become ignorant of diseases like diarrhea as families focus on making a living. But, nowadays, the health extension system has brought a good system of registering the health conditions of children, their weight, temperature, and other necessary information. This has been a good trend now on children under the age of five and for pregnancy. But most of the time, it is educated people who are using this opportunity. It is better nowadays.

P3: Mothers hadn’t been getting information from media, but this generation has access for media and can easily get the health information about Polio and others, from media and take their children to health institutions.

P6: I think the best thing done on the community is the measure taken by the government that demands the children to have birth-certification to be registered at schools. This has made every family to have the birth certificate, the health follow-up and the vaccination every month, six months, and annually as recommended by WHO. I think this has been creating a good opportunity for people to go for health services at health institutions.

I: What do you think is the main cause of diarrhea on children? You may consider cases in your area or in general?

P2: I think bacteria are the main cause of this. Our hygienic system and system of drainage of latrines are backward. The way we purchase foods in markets has been creating situations for infections. The second reason is the living style we live in; the way our residents built had been made us vulnerable for the transmission of viral disease.

P1: The major causes in children disease have been the packed foods we purchase from shops. Most shop owners don’t even know the expiration date of the foods they sell. The milks we buy from shops are infected most of the time, and EFMHACA [food, medicine, and health care administration and control authority of Ethiopia] don’t control this mechanism of selling milk door to door. This mechanism of selling unqualified milk and juices is more risky than the other causes, like purchasing vegetables.

P3: Even all the mentioned causes are appropriate. I think the most cause of the disease is the way we live and feed; where we feed and our latrine is very close. In addition to that, we live in very scattered residential areas and that create situations to more infections.

P2: It may have been mentioned, but there is a major cause; that is the drainage of latrines. Most of the time, the flood sweeps the toilet and leaves the dirt behind. This is all because of our poor living standard; there may be two households living in a place that is for one. The children usually get vulnerable to bacterial disease in summertime, as the flood sweeps the toilets and leaves the dirt behind.

I: What does the community do to prevent diarrhea?

P5: It is the same most of the time. I usually take my children to health institutions. But, my wife tends to try traditional methods like salt-water solutions. I don’t think that works, though.

I: How about to prevent it, not to cure it?

P4: We have to prevent it by personal and communal hygiene. We have to advocate about the necessity of hand wash

I: What else?

P2: We have to enable families to wash the hands of their children before meal, to eat fresh, to eat, and drink clean water. The major transmission means the food, so we need to make all children wash their hands before a meal. We have to teach children and families to prevent washing hands and eating fresh meals.

I: Do you know anything that has been implemented in your area, to prevent the disease?

P4: In our area, we have programs like ‘Buna Tetu’ [Coffee ceremony] to create awareness about sanitary systems for better hygiene, preventing such diseases, to drain the toilets properly on time, to clean their utensils properly and even diarrhea at home. There is training for families to take care of children below the age of five. We have campfire programs and cleaning campaigns to clean our environment and closed drainage ditches. But, the sustainable way we are planning is to create better standards of living and live in better places that a human deserves, with dedicated living areas farther from industrial areas. Condominium residential areas are better in this regard.

I: Let us now discuss other issues than Diarrhea and discuss vaccination. What is the attitude and understanding of the community about the vaccination for the children?

P3: I think the community has enough understanding of the vaccine now. I have never heard of people saying they’ve missed the vaccine, but there may be a delay sometime and go to health centers. They share the information with each other. I think the community has a good understanding of the vaccine.

P2: I think the community is well aware of the vaccination and more concerned than the health workers. The schools demand the vaccine paper so the people know they have to have vaccine papers. I think there has been excellent job done here, and there is good understanding on this.

I: Is there any different opinion here?

P6: There has been a good promotion on the necessity of the vaccine even delayed. The community has been bringing good changes on taking their children for vaccine and for following-up their health status and their weight. No one has been ignorant in our community.

I: Is that the case in all of your community? Isn’t there any reluctance from them?

P5: I have observed that the orphans living on the street don’t know what vaccine means. As my observation on the life skill training for them, they only know children need food to grow, there is no responsible for them and we need to take them to take the vaccines for Mengaga Kolf [Meningitis] and Polio. They just give birth and go away and never take the follow-up. We need to support them, and the community is well prepared for that without any advocacy. The youths living there give birth one on another, and they need to be given special attention.

I: You have raised a good point of reminding us about the people with no awareness about that. Why do you think the vacation is well accepted? Is that because it is mandatory for school registration? Or why

P2: I think it is because of the advocacy done by the media and the disability they saw in person. The generation can see the evil of the polio in person and even the people in the countryside know vaccine is necessary for pregnant women on Eight month of the pregnancy; the people collaborate and help the pregnant women to take the vaccine. This is because the community has seen the disabilities caused by the absence of the vaccine in the past.

I: Is there any other cause?

P1: The people in the past know the harms by the transmitted disease like Small pox and ‘Pertussis’. The people has learned from the harms caused by them and has been taking the children for vaccine services.

I: Very good. What is your understanding on Rota virus? I think it is known recently right?

P5: Yes.

I: I think it is introduced after 2,000 E.C. What is the people’s understanding on that?

P5: The vaccine for Rota virus is given for children with the symptom of fever and diarrhea. This can affect their digestion system and that can be changed to infections leading g to death, we have leaned that from the health extension workers. It was Polio and other diseases, but the mothers are discussing about the vaccination for the Rota virus. As my friends have mentioned before, people has learned from the consequences of the polio and have been taking their children to vaccine at all cost. The same is true here for the Rota virus, but we need to do more on this. Thank you

I: Is there any related opinion this? Do you know where the vaccine has been given? Do you have the information?

P4: I don’t have any idea; I know some vaccines that have been given home to home. If that is the Rota virus’s vaccine, it is done by health extension workers. There is something like refrigerated boxes for holding vaccines, and they give it at health institutions.

I: Do they give the vaccine at home or health institutions?

P1: It is given at health institutions.

I: Have you had the chance to observe when this vaccine is given home to home, as a campaign?

P3: I have seen when they measure the weight of the children and when they measure the mothers after delivery at home. But I think they give the vaccines at Health centers dedicated room. Unless it is for the polio vaccine, they give the vaccine at health institutions.

I: Okay. How about you, the others? Haven’t you heard about the vaccine for Rota virus? What is the purpose of the vaccine?

P2: It is as Negash has mentioned; it is to prevent diarrhea. I have heard this from health workers when they give awareness on that. I have never heard specifically for ‘Rota virus’ though.

I: What is the concern of people towards this vaccination? Some people might not like t or like that?

P5: The main purpose of the vaccine is to prevent infection inside the children body. If they don’t take the vaccine, there may be infection inside their intestine, anus and bladder. We can prevent that by the vaccine. The vaccine is given at the health center. Their weight, temperature measured, and their eye and throat are diagnosed. The medication is given as per their age, in health institutions but not in villages.

I: What are the concerns about the community about this vaccine?

P5: Most of the time, the community wants to take children to health institutions when they get sick; sometimes the people tend to learn from something they see practically. The people know more about Polio as they already saw the consequences. We need to advocate more about the Rota virus as the media is not working that much on it. Most people, especially fathers don’t have awareness about the diseases. The government has signed agreements to work on it but it has not been implemented well. The vaccine is very important but we need to aware more people about the virus.

I: What are the obstacles and good opportunities to give the vaccine services here? You all may not exactly know about it, but what would be the obstacles for the vaccination held on?

P4: Unless there is ignorance, I don’t think there would be no obstacles. I think the community knows well about benefit of vaccine from experience, health education from health center and extension workers.

I: Okay, what else? It could also be other problem.

P5: There are obstacles like the inability of mothers to take the children to health institutions because of illness or because of being busy taking their children for vaccination. As I have told you earlier, the orphans doesn’t know about vaccine at all as they don’t access media, and we need to do a lot for them. But, there is good awareness of the benefit of vaccine.

I: What are the other challenges that made people not go for vaccination?

P2: I think there should be done more on awareness creation. For example, there has been a TV program called “Tenawo be betwo” [Which is the health focused program] It has been giving better awareness for people. There should be the same program for children and adults. There have been governmental bodies’ discussions on cervical cancer and others, but not for this vaccination. In addition to that, we have to understand that the biggest problem is the Inflation in the country; a mother must be on work to feed her child and not have attention for the baby and don’t take to vaccination unless for emergency cases like diarrhea, fever, or vomiting. If there weren’t inflation, parents would take their children regularly as they could have more time to take their children to vaccination. There is no neighborhood giving advice not to go for vaccines, so the most common obstacle is an economic problem.

I: What kind of economic problem is that? Is there a problem to pay for the service, or is there a problem of traveling to distant health institutions or problems of paying transportation?

P2: The people can at least walk to health facilities if the facilities are nearby, but sometimes there may be emergency cases that lead to the death of children while taking to the children. So, economic problems are a major problem here. The parents must take care of their children and take their children and spend the health of their child as much as possible.

P4: Children miss vaccination for two reasons. One is because of the distant health facility from residents, and the second is not to have time to take their children to vaccine as they have to make a living.

I: How about traditional beliefs and religious causes that prohibit vaccination?

P2: There have been good measures taken for the children vaccination. But, there were challenges during the COVID 19 vaccination; the people have been relating the vaccine with 666 [Relate it with devil] and as something related to the USAs’ way of killing people. But, the people haven’t been relating the vaccination for Polio, Rota virus, and other vaccines for religion, with religious beliefs.

I: Has the COVID 19 become the obstacle and affect the vaccination for the Rota virus?

P6: There had been rumors and miss information about the Covid 19, and this inconsistent information made the people confused. But, in this case, there has been common awareness and information from health workers and specialists in the field. The other cause for the unacceptably of the covid 19 vaccination is the situation in which it was mandatory.

I: Okay, but has COVID 19 affected the vaccination for the Rotavirus?

P1: Yes, there were restrictions on movement during that time, and mothers had not been taking their children to health facilities not to be vulnerable to the disease.

I: How about the peoples’ belief about the cure by the vaccine?

P6: There had been misinformation from the media about COVID. So, we need to work on media to get the same results on vaccination.

I: So, has the COVID pandemic affected the other vaccinations?

P3: Yes, media is powerful here, and we learned that from the pandemic.

I: Okay, how about the opinion of people towards the vaccine? How about their opinion on the cure for the disease?

P2: I have never heard people saying the vaccine is very low in standard and in quality. The health workers give the services with great care and professionalism, so I have never observed people complaining over this.

I: Very good. Are there any other additional ideas you have?

P5: There were four types of vaccines from different countries, and people ask the difference between the qualities of vaccines before taking the Covid 19 vaccine. Some people relate it with some political conspiracies, but health workers have been trying to create better awareness even though there were low results.

I: How about the attitude of people for vaccination of children?

P3: There have been great results in the vaccination for the children, and the mothers always brought their children on time. I wonder why the people weren’t like this for Covid 19.

I: Is there anything you want to add?

*All participants: Silence*

I: Okay, if you don’t have something to add, I have finished the discussion point. Thank you all for participation.
