# Supplementary material for: Exploration of the social determinants of diarrhoea, rotavirus vaccine uptake, and vaccine ‘fatigue’ in Ethiopia, Kenya, and Malawi
Source: PLoS One. 2025 Sep 9;20(9):e0319691. doi: 10.1371/journal.pone.0319691 (PMC12419581; doi:10.1371/journal.pone.0319691)
Supplement: S1 Data — (ZIP) [file pone.0319691.s001.zip › Supporting Information Files/KY_08FGD.docx]

**FOCUS GROUP DISCUSSION 8**

**NUMBER OF RESPONDENTS-10 [ 8 FEMALES,2 MALES]**

**1.Can you please tell us some of the illnesses that affect children in your community?**

**R1-**A child may suffer from fever or even diarrhoea during their lifetime

**R2-**A child may have coughing episodes that do not end. It comes and goes. Then there is a way during the teething process they may suffer from diarrhoea and febrile episodes that do not end.


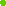


**R3-**They may suffer from fever and diarrhoea.


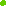

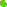


**R4-**You may find the child has fever and when you go for the fever medication and give them, they faint.

**R2**-You see these small children, the may have these scabies like symptoms or swelling especially on the neck or back which causes fever

**R5-**The body becomes cold, you might find the child vomits. Their legs become very cold and their body starts shivering.

**R6-**Stomache pains

**R2-**The navel does not heal fast so there’s something that remains which goes outwards then returns back inwards, so we as parents we are wondering where that thing comes from because if their navel is tied it do not heal fast.

**2. Which of these illnesses do you consider to be a burden in this community? Why do you say**

**so?**

**R7-**Fever.Mostly fever. A lot of fever, sometimes it may be in excess even when you give the child medication, it doesn’t cool down the fever. It may reach a point where they convulse because of the high fever

**R2-**Others may have wheezing breathing sounds and you can hear the chest is as if has something placed in it then the fever settles at the back of the head. This troubles the child and when that fever settles there, you see that the child becomes dehydrated. This is a small baby of around five years and they might faint.

**R8-**You might find others when they cough they nose bleed because of the cough. And when they cough their cough sounds are drum like.

**R2-**Another that I remember is fast breathing. The child breathes fast until you see their stomach rising and falling and another breathes from deep inside.

**R9-**Mostly diarrhoea. Let’s say there are two types of diseases, one which the child diarrhoeas a lot. Then there is this other one which is having high fever. When you go to the hospital you are told the child has pneumonia and malaria. Mostly small babies are affected by diarrhoea and pneumonia.

**3. If you were to rank these illnesses in order of priority, what would you rate as the top three**

**diseases affecting children**

**R10-**The first one is mostly high fever. When you take the child to hospital you are told that they have malaria

**R3-**The first one is fever then diarrhoea.

**R4-**The first one is fever, the second is diarrhoea and the third is vomiting.

**R2-**The first is mainly fever. When the fever is a lot, you are told they have malaria. When they have this high fever they diarrhoea and vomit simultaneously. During this vomiting and diarrhea episode ,their breathing pattern becomes bad

**R6-**The first one is fever, the second is diarrhoea and the third is common flu.

**4. Can you tell me the health services/facilities available in this community? Where do you**

**access health services? [Probe: how much does it cost to access these services, how long do**

**people have to travel to access the services**

**R5-**I access health services in Njenga and Code Care. I go by foot and am not charged anything

**R4-**I access health services in Njenga.The other facility is Mukuru Health Center. There is also St. Mary. For private health facilities there is Code Care. I usually go to Njenga but when there are some certain tests that are unavailable there I go to a private facility or when there is a long que and your condition is unfavorable you may opt to leave.

**R4-**You are not charged anything when you go to Njenga but there was once I went there and there was a test that was needed, I was told to pay 200 shillings. I go to Njenga by foot and also access the private facilities by foot too since the distance from my home is not long.

**R3**-I live at Ruben. The hospitals around me are Maendeleo and Njenga. I take my child to Maendeleo Hospital. I am not charged anything there and everything is free. The distance between my home and the hospital is not big so I access it by foot.

**R10**-I live in Njenga.The hospitals around me are Our Lady, Mukuru and Code Care. When my child is sick I take them to Our Lady which is a public hospital. I am not charged anything and everything is free. Sometimes though, when the child is sick and the hospital staff send you to their pharmacy you find some medication unavailable there so they write down the missing medication for you and tell you to purchase it from other pharmacies outside the hospital. I walk to the hospital since its not far from my home.

**R7**-There is Mukuru Health Center,St.Mary and Kware.For clinics I go to Kware,for treatment I go to St. Mary or Mukuru.I chose these hospitals because they are cheap and you do not pay anything although at St. Mary you pay for the consultation fee and if you are sent to the lab you don’t have to pay. Medication is available and on instances it is not you go and buy from other pharmacies outside the hospital. At Mukuru Health the services are also free but the lab tests you are sent outside the hospital then you take the results back to them. The medication available there you can access and the missing ones you source from outside pharmacies. I walk to all these facilities.

**R2**-There is Maendeleo which is in Ruben. Then there is Kwa Njenga, Our Lady Of Nazareth and Mukuru.I prefer Maendeleo because it is close to my home. Sometimes when the child is overwhelmed, I rush them there on a motorcycle but if the fever is mild I go there by foot. I am not charged anything at the hospital, you are given the medication you need unless it is unavailable and you are sent to buy it from outside. I pay either one hundred shillings or one hundred and fifty shillings for the motorcycle ride to the hospital.

**R3**-In Ruben there is Maendeleo Ruben Center and Better Living Health. I take my child to Ruben Center. I am not charged anything, everything is free. There are times where medication is missing and I have to purchase it from outside. I access these facilities by foot.

**R8**-I live in Ruben. The hospitals around me are Maendeleo and Our Lady. I go to Our Lady which is a public hospital. I am not charged anything there everything is free apart from the unavailable medicines at the facilities which I am sent to buy from outside. The lab tests are also free. I access these facilities by foot

**5. How do most people respond when a child has diarrhoea in the home? [Probe: What do**

**people do at household level? at community level? Where do they go to access treatment? Do**

**they take antibiotics? Where do they access antibiotics? Why do they access antibiotics?]**

**HOUSEHOLD LEVEL**

**R2-** Because you do not know what caused the baby’s diarrhoea, you are supposed to rush them to the hospital. In the past we would rush them to the nearest facility and they would tell us the child has a fever. When you go to the hospital directly, the proper tests are taken and they are given the right medication. I used to stay with more than one child and there is no way you will decide to mix sugar and water or tea leaves to curb the diarrhoea, you will have to rush them to the hospital because if you give them the sugar and water mixture and they stop diarrhoearing but continue vomiting you haven’t treated anything.

**R10**-The option is the same, you rush the child to the hospital

**R7-**Some types of diarrhoea are not for observation. There are some people who tend to observe their children first for some days if they have diarrhoea but what I know is if a child has diarrhoea they are supposed to drink a lot of water as you take them to the hospital to know what is causing their diarrhoea

**R6-**You rush the child to the hospital.

**COMMUNITY LEVEL**

**R2**-According to other mothers who help us in the community, you take some sugar and salt and mix it with warm water then give it to the child. Then you observe the child for the day and the day after. If the diarrhoea doesn’t stop, you take them to hospital or you are told to rush them to the clinic which is bad because you buy zinc or you are given ORS which does not help and the diarrhoea It.

**R4**- Some people give their children wheat flour. They mix it with water like porridge and give the child. The child then stops diarrhoearing.

**R10-**Some people give their children yoghurt so they stop diarrhoearing. I don’t think it helps. If they see that it has not stopped they rush to the chemist and buy medication of which if given fever medication the child’s fever will reduce but their diarrhoea will not, it will still continue.

**R2**-Some people boil plantains without oil, it is let to cook down and then stick it on the child’s stomach. Then the child gets better.

**R8**-I have heard of the wheat mixture method to cure diarrhoea in my community.

**Do they take antibiotics? Where do they access antibiotics? Why do they access antibiotics?**

**R4**-When you take the child to the hospital, you are given the antibiotics.

**R9**-Similarly, when you take the child to the hospital you are either given the antibiotics or sent to purchase them from a chemist, but you take the child to the hospital prior to buying the antibiotics from the chemist.

**R7**-I once took the child to a chemist for the first time when they had diarrhea. They were given ORS, they weren’t given anything else by the way Just ORS medicines for fever and cetirizine. I gave the medicines to the child and they did not stop to diarrhoea. To make matters worse, the following day the child begun vomiting so I had to take them to the hospital. From there on I have never tried taking the child to a chemist I just take them to the chemist. I would rather be given a description by the doctor and if the medicines are unavailable at the hospital, I go and purchase them from the chemist.

**R2-**The good thing about taking a child to hospital when they have diarrhea, they are given a complete dose of medication for diarrhea which we as mothers used to assume is clear water. We were wondering why we are given water when we have it in our homes, later on we were explained to that it was medication. When you are given the medication in a small bottle the child sucks it and it boosts their immunity so the diarrhea stops and the child gains some strength.

**6. Can you tell me some of the enablers and challenges that people experience to access**

**treatment for diarrhoea diseases?**

**CHALLENGES**

**R2-**Some challenges that we face when we go to the hospital is finding many children at the hospital because diarrhea is a challenge that affects almost all mothers and children.You find the patients at the hospital are a lot and before your turn to see the doctor gets to you, your child’s condition worsens. That is one major challenge we face.

**R4-** Another challenge we face is when we get to the hospital to see the doctor and are sent to the lab for some tests, you find out that the tests you were supposed to take for the child are unavailable at the hospital. Because it is missing there, they end up giving you some prescriptions without the tests.

**R3**-I agree with what she has said. The challenges that we face is that you do not get some services when you go to the hospital like lab tests.

**R7**-The challenges that are there is when you go to the hospital you find some tests missing there and to be honest you might have gone there because of financial constraints. You are sent outside for the tests and you have no money to cater for those tests outside. Another thing is medication. These hospitals have pain killers and if you were prescribed other medicines than that, you are forced to look for them outside and we avoid that because of financial constraints. You cannot go to a private facility because their charges are high so you opt for the public ones because you know the most that you will be charged will not exceed five hundred shillings, and maybe that is what you have. At the private facility, the lab tests are over six hundred shillings, you need to purchase the medicines and you have no money. Those are is the challenges that we face.

**R 5**-Transport.At night you cannot go by foot to the hospital so you are forced to look for transportation because of fear. Sometimes you find there are no doctors at night in the hospital because I think these hospitals mostly operate during the day time and close at night so you might go there at night and not find a doctor.

**ENABLERS**

**R2**-Sometimes when you go to these hospitals they refer you to another one where you will not miss medication and will be able to see a doctor. That is a big advantage we face. Even if the child’s medication was missing you are referred to a bigger hospital and are directed to where it is.Then it is up to you to know the transportation costs and how you will get there.

**R2**-The help that we have on our side are frontiers. If you approach them and tell them you are going through something they give you a way out and you are able to get help.

**R4**-On my side, I do not have any enablers. Even the NHIF card that would have helped I do not have it because I am unemployed and unable to pay for it.

**R5**-I also do not have the NHIF card.

**R8**-I also do not have a NHIF card.I pay for treatments out of my pocket. When we become sick in my household we sort ourselves out. If we go to the hospital and get prescriptions and we don’t have money, we just come back home.

**R7**-You might go to the hospital and be told that yes you have a card and it would have taken care of the service charges but you are told the card is not working. This card issue has become problematic, you go to the hospital and expect the card to cater for the charges but you are told the card does not work while you were expecting it to.

**7. What do people do to prevent diarrhoea? [At household level, at community level?**

**R11-**Checking the child’s environment and ensuring that they do not eat dirt.

**R8**-Cleanliness .The child’s environment should be clean, the utensils that they use should also be clean.

**R4**-Cleanliness.The first thing is looking after a child’s cleanliness. They should not eat dirt and the food that you give them should also be clean.

**R6**-Cleanliness.You should not leave the child in a dirty environment.

**R5**-Cleanliness is the first thing that you should consider. This means that the environment you are in should be clean. For food you should make sure the utensils you use are clean and the water you use should be clean. You should boil your drinking water, wash your hands after visiting the toilet and wash the fruits and vegetables that you buy from the market before eating them.

**R7**-As she has described, cleanliness should be observed. You cannot stop a child from playing outside but you should make sure that when they get home the food that you give them should be cooked with clean water. Make sure the child washes their hands before they do anything and make sure your toilet is clean because most of the time dirt comes from the toilet. You should also ensure that you boil the water and if not, you should treat the water instead.

**R7**-Keeping toilets clean is a big challenge because we share the toilets. There are some people who have plans in the plots and make sure that this week one person washes the toilet, the next week someone else washes it and they make sure the toilet is okay.

**R2**-You should ensure that you maintain a child’s cleanliness and whatever the child comes across even if it is a ball for playing it is clean, their utensils are clean because for children of ages five and below there are some we must feed them with spoons. Their utensils should be cleaned and covered because there are cockroaches and flies that spread germs and when they come from playing the first thing you should do is clean their hands before giving them something to eat or drink

**R2**-It is hard to maintain cleanliness in our environment because where we live there are sewers next to us and a child may place their feet inside or their sweets may fall into the sewer but we need to teach them and guide them that if their sweets fall inside their sewer it is dirty and not safe for consuming. We should also at least monitor them because if we do not we are the ones who will end up suffering.

**8. How do people in this community perceive childhood vaccines [Probe: why do you think**

**childhood vaccines are widely accepted? Why do you think childhood vaccines are widely**

**resisted?**

**R7-**The vaccines that I know of are BCG which they are given at birth.

**R5**-I also know of Measles.

**R10-**Polio.

**R2-**Clear water. We used to think that it is just water. Sometimes as a parent you are forced to ask what it is.When the child is given medication and they are vaccinated on both of their thighs that is when they are given that to suck it.

**R3**- Rotavirus

**PERCEPTION OF THE VACCINES**

**R5**-They accept the vaccines because they are told if a disease emerges the child might be affected by it they might fall sick and die. We are told that vaccines are important.

**R5-**People in the community are accepting of the vaccines. A lot of them accept them because they are told if they do not vaccinate the children they might fall sick. Mostly the polio vaccine, which they are very open and accepting to because it is advertised on tv and people state the effects of not being vaccinated so there is a fear instilled in people hence encouraging them to be vaccinated. Also awareness is a contributor.

**R2**-People accept these vaccines because the Government directs and educates us well as mothers. When mothers become pregnant, they attend the clinics are educated so that the moment they give birth they follow these instructions because they do not want their child to suffer.

**R5**-I take my child to all their clinics because I love them and I care about their wellbeing. I do not want to miss their clinics when they are young as this might affect their health later on in life as they grow older and I do not want that. I want the best life for them.

**R2-**There are some who refused the polio vaccine because you do not take the child to hospital rather the vaccinators come to your home from plot to plot. They are afraid of these vaccinations because they do not look like they come from the hospital or are doctors but if they are explained to and educated they later accept the vaccines. These are trust issues for the vaccination personnel.

**R10**-For some it is their churches that influence them. They do not believe in going to the hospital, vaccinations or going to the hospital for medication. They don’t trust that.

**9. How about rotavirus vaccines? What do people think about rotavirus vaccines? Where do**

**they access rotavirus vaccine? [Probe: What do they think are the benefits of rotavirus**

**vaccines? What concerns do people have with rotavirus vaccines?**

**R3-**Rotavirus prevents diarrhoea.

**R10**-You ask the doctor about the benefits of Rotavirus. When the doctor gives the child the vaccine you ask them what it is and its benefits so the doctor explains it to you.

**R5**-In the past the nurses would shout at mothers for asking questions about Rotavirus but these days they don’t.

R8-These days the nurses are better. Before you would get up early and go to the hospital so you can come back early to continue with your other activities. You might go there and miss them so you sit there waiting for them and there are a lot of people. When the nurses come, they waste time greeting each other while the patients are still waiting for them. They then rush calling out your names and if you miss your chance they shout at you when they are the ones who came late. You do not have time to ask them questions. These days they are better though.

**R2**-These days the nurses are better, they even ask you if you have any questions and they explain them to you.Yes,these are public hospitals. These days people have become better because they know people have suffered they become frontiers and they help you.

**10. What are the enablers and challenges for people in this community to access rotavirus**

**vaccines? [Prove: cost, distance to access services, cultural/religious beliefs, impact of**

**COVID-19, perception of vaccine safety]**

**R4-**There are no challenges in accessing these vaccines because they are always there, they are never missing.

**R4-**When you go to the public hospitals for the vaccines you are not charged anything.

**R2**-The Corona virus vaccine made some people worried because you would here people saying that they were vaccinated and their body became weak and they were unable to eat or do anything when vaccinated at work.

**R2**-Also when students were vaccinated at school they were unable to continue studying because they did not have strength, so when we heard those rumors we would become afraid. The good thing is that small children were not vaccinated, only the high schoolers an university students.

**R10**-It never affected a parents decision to take their child to get other types of vaccinations because we knew that the corona vaccine was for corona and these other vaccines had no other complications
