# Supplementary material for: Exploration of the social determinants of diarrhoea, rotavirus vaccine uptake, and vaccine ‘fatigue’ in Ethiopia, Kenya, and Malawi
Source: PLoS One. 2025 Sep 9;20(9):e0319691. doi: 10.1371/journal.pone.0319691 (PMC12419581; doi:10.1371/journal.pone.0319691)
Supplement: S1 Data — (ZIP) [file pone.0319691.s001.zip › Supporting Information Files/MW_5FGD.docx]

**Facilitator:** we are stating, that the noise is over now, but you will raise your voice to be well-recorded. There are several issues that we would like to discuss here, we are going to discuss the diseases that affect children here in Bangwe, we are going to discuss what you do here to deal with diarrhea, prevention, and curing, we are going to discuss also about under-5 vaccination, what do people say about the vaccines. Finally, we are going to discuss the rotavirus vaccine, so there are several topics that we would like to discuss, but firstly, let’s start with diseases that affect children here in Bangwe, what are they? Everyone can start

**00:** every disease or…

**Facilitator: (**every disease that affects children

**02:** I am a parent, I have children. Diseases that affect my children are diarrhea and malaria. I think they get sick from diarrhea because they eat without washing their hands. They get sick from malaria because they don’t sleep under the mosquito net or when you have hanged a net, they remove it, and mosquitos bite them, so they end up getting sick from malaria, to me that’s what I can say

**Facilitator:** mmh, diarrhea, and malaria, is there any other disease?

**04:** I have observed that most children, especially in my household, have diseases that affect children, such as influenza and coughing. What I have noticed is that when children are with their friends, they can transmit diseases because these diseases are communicable. Malaria is there as well, they have diarrhea as well but not often because it depends on the food they have eaten at that time, and whether that food is suitable for a child or not. Let’s say we have bought treated beans, and all of us have eaten, everyone has diarrhea, so diseases that are common are flue, cough, and diarrhea

**05:** I want to comment on what number 2 said. Diarrhea is common in children because it is difficult to control a little child when playing with friends or controlling what to eat. When the child has diarrhea, as a parent, you need to help that child quickly by taking him/her to the hospital. The main thing is that hygiene is an issue for us. We just litter everywhere. A child is different from an adult, you can control an adult, but not a child just do it, you see them putting something in his/her mouth, ‘’Don’t do that!!’’ so this disease is difficult to understand because we just litter everywhere

**Facilitator:** what happens with garbage here in Bangwe?

**05:** (not clear) If you go to the street, you will note that some have swept and some just throw away masks anyhow, so a child just picks such things and eats yet there are germs on such things, so germs affect the immune system for a child because the child’s immune system is different from our

**03:** just to add to the issue of hygiene here in Bangwe Township. Hygiene is an issue because, for example, we have children who wear diapers, and most parents don’t have a pit to throw the used diapers, they just throw them everywhere as long it’s done for them. When children see such things, they start playing with them and catch some germs, they will have diarrhea too, and maybe flue and cough plus maybe skin rashes that are affecting children nowadays, so I wanted to comment on the issue of hygiene

**Facilitator:** mothers are just throwing diapers anyhow, do you have a place of disposal of the diapers?

**00:** we don’t have it, it’s only at the bin, I can tell you to walk on foot, I and this one come from the same area, once you pass the Seventh Day and cross this river, you will see they have littered them everywhere and some are throwing into the rivers and dilute, some use the same water, they are affected. There is no special place for disposing of such things or pit to throw such things, it can be good to go door-by-door telling people to have pits for throwing such things and once it’s full, cover it, and you can avoid many things

**01:** hygiene is an issue here (not clear) another thing on this same issue about water, water is a problem here. Only a few people use treated water most people use water from river sources or wells, and as you can see, this area is hilly. Someone can build a house up there, and have a toilet there, another one can have a well down here, that’s why diarrhea is an issue, for under-5 children, diarrhea is linked to the beliefs, ‘’Aah they are flashing out bad things’’ (not clear) but diarrhea is common here

**06:** on the issue of diarrhea in children, some of us live in railway houses and we have flash toilets, some adults use the toilet and never flash out and a child may go there and do something without our knowledge, and when the child is back, can just start eating, and at the end, the child may have diarrhea. Secondly, on the issue of diapers, it I true about our rivers, there are different diseases. You find a child bathing in unprotected water and the result is that children are affected by several diseases apart from diarrhea but, there is a skin rash that is affecting children, (not clear) if they help us to have a place for disposal, we can prevent the disease

**Facilitator:** on the prevention, how do you prevent diarrhea on your own?

**04:** the prevention of diarrhea, most of the time we men or families don’t manage to prevent it because we just watch our children instead of telling them this is bad, let’s say there is research going on, there is a vaccine that people are receiving. We are the ones who say, ‘’Don’t take my child for vaccination’’ yet the child will be unprotected and everything that the child will touch or us, will cause a problem to his/her health because we have refused a child to receive the vaccine. As that one said, we have the toilets that we use, when we are not all available, a child may go to the toilet. The broom that we use for cleaning a toilet, a child cannot manage to use it. He/she can see a toilet tissue but, maybe because we did not tell a child how to use a tissue, a child cannot use it and after the toilet, he/she can start eating without washing hands, the child cannot be protected. The child should be protected from the beginning, if the child has received the vaccine and is doing something wrong, we can talk to him/her that you are doing wrong, even if a child is doing that, because he is protected already, diseases cannot function well and the result, a child survives. So, we are advisors to our children, telling them what’s good or bad when they are young, so even when we are away, a child will still do that which is good, but if we don’t advise them, a child cannot know what to he/she is doing

**Facilitator:** Okay, I understand you, and you have focused on what you should do isn’t it?

**04:** yes

**Facilitator:** but, let’s focus on what you are doing, you said that you are failing, which means you are not preventing isn’t it?

**04:** mmh (yes)

**Facilitator:** you have also said it for parents who refuse their children to receive the vaccine, we are going to proceed with that issue but for now, let’s hear from others, about how you prevent diarrhea in the household

**07:** to prevent cholera, we should wash our hands with soap after the toilet. When a child has gone to a toilet, follow him/her after the toilet and wash his/her hands with soap (not clear)

**Facilitator:** you are representing the people of Bangwe, there are 7 of you but, you have represented the people of Bangwe, and so, when discussing, we should be thinking if what we are talking about is what is happening in Bangwe. You have talked of hand washing using soap, is it true people in Bangwe do have soap for hand wash after the toilet

**04:** That’s not true, if you go to every household, you will never get water for washing hands, this is not happening and that’s why I am saying, we are failing in our responsibilities because we should be leading by putting water somewhere so that a child should know this water is for hand washing, the child will get used to that even if he/she goes elsewhere. During COVID-19, it was started, ‘’wash your hands with soap after you do everything.’’ Soap helps in many ways and we are failing to buy soap and put it in the toilet. You will never find soap in every household including me, I have no soap, we are failing in our responsibilities, so we should not be saying something to back ourselves up that we have soap, that’s not true and that's why, this disease is worsening, because we are not fulfilling

**Facilitator:** Okay, you are not opposing what this one said but, I want you to differentiate what we do and what we are supposed to do, these are two different things, I thank him for what he said that if we would be doing this, we can prevent, but, what are you doing currently, so let’s look at both sides. If you tell us that you have soap, it will give us the picture that everyone in Bangwe manages soap and there can never be any diseases in Bangwe

**00:** Okay, on prevention, parents should be the leaders for everything, we should control what a child is using in the toilet. What I observed is that some scholars said (not clear) different activities, and different steps. Most of us live in rented houses. If you go to the communities, you will notice that most people are living in rented houses, so we have that mentality of saying everyone should mind their business. If you sweep the waste and leave it there, someone will proceed with it, and so, if I control my child’s behavior, diseases will still come because of the environment. The diapers we are talking about, are throwaway everywhere and the astray dogs just pick them, that’s also contributes to this problem, so there is a need for guidance for door-to-door sensitization because there is no one who can do anything only if you are trained, to you, you think it’s correct yet it is destructive. The main issue is that most of the people stay in rented houses and everyone minds their business. This problem is easy to overcome, it all depends on the counseling in these households, and otherwise, the percentages will go up every year, if you go to Banagwe and Makheta, you will observe that this problem is common in highly populated areas. For example, I am dropping my child to school (not clear) in short I will be hearing everything, if my child diarrhea, you will hear, ‘’This is this development’’ If we are to deal with this problem, counseling should go through that and I believe we can deal with this problem fast, that’s what I think

**Facilitator:** Okay, we have talked of prevention, what do you think is contributing to this issue of diarrhea, let’s now talk about what you do when a child has diarrhea, what do you do?

**02:** when a child has diarrhea, like in my family, let’s say it has started at around 7 in the evening, the hospital is far away, what we do firstly is to dilute the sugar and warm water and give the child because when a child has diarrhea, he/she loses water from the body, so, sugar and water replace the lost water. If we have an opportunity to take a child to the health facility, we do so but after giving the child warm water mixed with sugar, we rush to the hospital so that health workers can do their part

**04:** the problem that I observed with diarrhea is that we hesitate, and that’s why it becomes worse. It is not often for a child to get sick today and be taken to the hospital, it’s not possible. What happens is, ‘’A child is sick, what can we do?’’ some give a child aloe vela, that’s a problem. We take a child to the hospital when the condition worsens when eyes change, and when the child is seriously sick, such things. We take a child to the hospital when we see that the condition is worse, otherwise when we notice that the child has diarrhea, ‘’Aah let me do this first, ‘’ and the mother is leaving as well. ‘’Aah just carry the child on your back’’ and when the condition continues, ‘’aah let’s try this and that,’’ we are delaying child.’’ We are taking the child to the hospital when the condition is serious. Maybe my wife is doing a business, I am doing some peace work, and there is no one to take a child to the hospital. When it comes to worse, that’s when we take a child to the hospital

**03:** there are some things that make us lose children because of diarrhea. Just like what he said about hesitation, some do believe that once a child has diarrhea, hasn’t finished teething, and we delay dealing with the disease in the process. There are some diseases that need medical attention, like diarrhea because the patient is dehydrated but some go to the religious leaders for prayer, yet the child is dehydrated. It’s a big problem because we hesitate. Others because of believe, that disease needs medical attention, and the child should receive the treatment, what my colleagues have said, there should be door-to-door sensitization especially on the issue of diarrhea in children and not only children, adults also because if the household doesn’t practice hygiene, not only children are affected, even adults are affected, so people should be sensitized

**00:** okay, for me (cross-talk)

**Facilitator:** let him finish

**00:** the question is what we do when a child has diarrhea, (not clear) when the child has diarrhea, all parents want is for the diarrhea to stop, we are not really interested to know why a child has diarrhea. It can be possible that the diarrhea is because the child has a problem in his/her body or something has happened to his/her health, most people just want diarrhea to stop, so most people do what he said, diluting sugar and warm water, so if a child has diarrhea, rush to the hospital, maybe the issue is how access to the hospital, so we have a hope that diarrhea will stop after giving these to a child, but we have to take a child to the hospital

**0:** (not clear)

**Facilitator:** laughing, ooh

**0:** as this gentleman said, I believe once a woman is pregnant, she needs to be well organized in that house because you know that in this situation, you have carried Jesus’s cross. When a child is born (not clear) I will give an example of a person who is here, (not clear) like this man, one day I visited him at home I found him drinking and I asked, ‘’What are you drinking?’’ I thought he was going to tell me, ‘’I have stomach-ache, so I am drinking thanzi .’’ which means he had a problem and has decided not to hesitate, the first thing as a man, or a family, you should have things for emergency use, you are not a doctor of course but, first aid just like what these ones have said (not clear)

**Facilitator:** what you have said, and what this one has said about what we can do on our own like buying aloe vela, attending prayers, but what I want to understand is that why do you think what you do is a problem as we are dealing with diarrhea in children?

**04:** it is a problem because we inherited it from our parents. We are abandoning our culture because for us to grow up and get married, it means we are established. What is common now is that ‘’our parents told us to do this when this happened!’’ that’s what is common to us. If we abandon this and start our own things as a family, it cannot be what that one said about prayers. It’s okay of course but, we should first go to the hospital for treatment and if the condition does not improve, we go back to the doctor and explain, ‘’I came with this problem but, maybe the treatment is not suitable for this child,’’ they can change the prescription

**07:** I just want to comment, we hear that once a child gets sick, we should have first aid. Diseases like diarrhea, we should take 6 spoons of sugar and one spoon of salt and dilute, then we give the solution to a child, then we take the child to the hospital, even at the hospital, they do ask, ‘’Did you give a child anything?’’ (not clear) first is to know the problem, and if we have known the problem, whether it is stomach-ache or headache, (not clear)

**03:** the main problem that makes us fail is that this disease does not end. If we have failed, we need to be taught so that we can correct and when make corrections, the problem will end. We are losing many children because of diarrhoea

**Facilitator:** number 04 talked about the culture that we inherited from parents, do you agree with that?

**03:** I somewhat agree with him but, let me say this (not clear) where one was born and grew up shapes you. For example, I am used to eating Kondowole nsima, and if I travel to Karonga or elsewhere, they like rice, it will be difficult for me to assimilate, so there are three things that make us play with life like a ball. Diarrhea is a dangerous disease. Norms from parents misled us. Another thing that I have noted is that our religious friends are contributing very much to this, and for a man, you need to become like a mad person if you want to do something. That should help you. If I am not mistaken, my colleagues talked about prayers. If a child has diarrhea, you cannot take him/her to a pastor and say please help my child, then you are just worsening the condition because if that’s a problem, go to the hospital. The problem in the families is because account number one is a wife, even if you check with the issues of prayer, it is women who do that and they come home with something, and if a man says, ‘’My wife, we should not do this.’’ Then you start quarreling, so these two things are disrupting us, and worsening this problem. I am not saying people should not prayers are wrong but, I think me I tell my wife, ‘’Do it, but when you come here, once you cross this door frame in, know you are married, respect this marriage but, if you take rules from church and bring them here, it will not work.’’ So, that’s true about norms from parents because things are now changing, as we move forward, we have to look forward as well and find a solution for this problem

**06:** on this of norms from parents. As we are leaving, we should be looking at where we are going and where we are coming from. Previously, parents had their beliefs even if a child is sick but where we are and where we are going (is not clear). The issue is that different diseases are coming and for us to do what our parents were doing (not clear). When we have a problem like diarrhea, we should rush to the hospital

**Facilitator:** we are discussing how you prevent, and there is the issue of Thanzi ORS, prayers, norms from parents, and number 05 talked about pharmacy

**05:** eeh (yes) I was saying….

**00:** another thing I want to add is that there was an organization that was distributing medicines in the communities and that medicine was for us to treat water to prevent cholera. If there is that opportunity, with diarrhea, you cannot start from morning up to evening, you die. If there can be free medication because we have different financial status. If we go to Mtopwa, we will find someone with money who is able just to buy flour for nsima, but, cannot have money to buy medication for a child. So, is there an opportunity for free medication that prevents diarrhea

**Facilitator:** what is that?

**00:** Thanzi ORS

**03:** even chlorine

**00:** exactly!! People will be trained and sensitized, ‘’ladies and gentlemen, when you notice that your child has diarrhea’’, because there is diarrhea which is normal, and there is another one that shows a child is sick, you train people, ‘’when a child has this kind of diarrhea, give this to the child,’’ they will not bother taking money and buy, you have protected that child and the child will be a president or a minister tomorrow, so there is a need for a free assistance

**Facilitator:** you have talked of normal diarrhea and the one as a result of illness, please explain to me, how you differentiate these two.

**03:** as an adult, there is diarrhoea, and there another one (pali kutsegula, ndiye pali kutsakula)

**All:** laughing

**03:** from the time we started discussing…..

**00:** (you have gone (to the toilet) every now and then

**03:** diarrhea that shows you are sick, that’s a message telling you, ‘’gentleman, you are not okay, ‘’ on the same spot, you need to go

**Facilitator:** that’s sickness one

**03:** yes, sickness one

**Facilitator:** mmh

**03:** there is another diarrhea which is a result of what you had eaten, you just go twice (to the toilet) then you are okay, but the diarrhea that we are referring to is one that even the time you invited us, we would have said ‘’there is no toilet here so let me stay’’ so going to the toilet every now and then

**00:** there is normal diarrhea and the one that shows something is wrong with my body. So, it is the degree of the diarrhea, let’s say someone, once he/she drinks milk, they have open bowels, and if you continue, it means something is wrong in your body, unlike when I eat today and tomorrow I go to the toilet, I cannot say I have diarrhea, that’s normal one (not clear) not that when you go to the toilet and then ‘’free, phree!!’’ No, that means you are not okay. Whether you do it much or not, but you are not okay

**O:** diarrhea is just diarrhea, once you wait, that’s when you worsen a condition because every diarrhea, you are dehydrated, please research this, you will find out that every diarrhea you are dehydrated, if you are waiting, what are you waiting for?

**Facilitator:** to see whether you will come again

**0:** then you are waiting for an accident and that day, he and I would be busy carrying a patient to the hospital, and everyone in the community (not clear) this and that…

**000:** I believe in what this man said about free medicine distribution (not clear), people did it but that time, when a health worker comes to a household ‘’I don’t want this in my house’’ whether it was good or bad, it will never be used at that household and the health workers got tired to go to the communities. If it is the best medication to stop diarrhea, even if someone is given Thanzi, he/she cannot refuse it, if they bring chlorine, our parents will not accept it even our wives will not, ‘’chlorine for what?’’ That means the health worker has just left chlorine which will never be used. We are even failing to use chlorine in a toilet, we cannot treat water using it

**Facilitator:** do people refuse to use chlorine in the toilets?

**000:** Ask if there is a toilet in which chlorine is used

**00:** there are different kinds of people, there are some people, what is difficult for us is awareness. If you are ignorant, you cannot refuse to use chlorine in your toilet, you will know that chlorine kills a smell and germs. When they give you a water guard to treat water, that water is protected and it kills germs in the water, that’s someone who is aware, and those who are ignorant should be told, ‘’You refuse these things, they have these advantages’’ that’s the main issue that there is a need for counseling

**Facilitator:** on this same issue chlorine or water guard, I want to understand, what differentiates those who are ignorant from those who have knowledge.

**00:** let’s answer it this way, what is difficult is that health workers talk a lot of things. They come with this today, before the month ends they come with other things, and people don’t understand them and they don’t know which is which. What I am saying here is even if you are with community members, they may chase you out, saying, ‘’Aah we are tired of these things!’’ I am talking these through experience because parents or those people, most of them I know and I have worked as a health worker before, but not like you. I was just a civilian to say, and when we go to this household and talk to them, they would agree, but when you go there again, you will hear them say, ‘’What is she saying, don’t come here again.’’ The medication that people can use for cholera is Thanzi, and not treating water, ask anyone here, which toilet chlorine was used, and none. You will never smell chlorine in any toilet

**0:** they say chemicals to lower the toilet level

**Facilitator:** you have said healthcare workers bring many things at once, what does that mean to people in the communities?

**00:** it brings the threats

**Facilitator:** what threats?

**00:** Recently there was COVID-19, and vaccine was introduced. There were many types of vaccines, ‘’when you receive this one, we are okay, when you receive this one, you will wait for some months and after that, you will come for another jab’’ ‘’Where did we meet? At the office, what were you doing?’’

**All:** laughing

**Facilitator:** he is afraid

**0:** I went for a vaccination

**Facilitator:** what vaccine did we receive there?

**0:** COVID vaccine

**00:** there came a cholera vaccine that needed us to too, and there be another one coming, which is which?

**000:** let me add that, these people don’t want your lives, but according to the prevalence of the disease, they are protecting you because when they say ‘’Malawi’’ they mean you

**00:** then I am saying, this one and I went and we do go, but there are some people here who did not go, why?

**000:** I can say according to their beliefs

**00:** accepting is difficult

**000:** understanding is important because these diseases….

**00:** (you and I do understand, and because we are with these people, we can be faithful listening to them because we want them to have that picture and say the people that I met with do understand yet we are not, and after here, we will speak different thing…

**00:** (to be honest….

**000:** (what I want to say is that we are many

**Facilitator:** what you are saying and what he is saying are different things. One is talking what is supposed to be done while another one talks about what is happening, so there is not that much difference but, we should look at both sides. It’s very good news because I thought we would discuss this later but, you have introduced it and previously, we talked about diarrhea prevention and you said ‘’it is us parents who refuse the vaccine.’’ Let’s continue from there. You have said people have different beliefs regarding the vaccines. What are the beliefs people have regarding the vaccines?

**00:** hearsays

**Facilitator:** what do they say about the vaccines?

**00:** even when the children run away from schools, it’s because of vaccines. When they see a health worker with the vaccine, the issue is, because I went to a certain hospital and someone died, then we think I will die if I get vaccinated, that’s how people conclude, ‘’Don’t do this,’’ you understand

**Facilitator:** mmh

**00:** the vaccine is bringing confusion, we were hearing it was weakening some, others fainted, we have different blood types and because of that, others were saying, ‘’Once you have received the vaccine, after some time you would die.’’ Such things made people not get vaccinated because even those who go door-to-door, there are many people in the communities but you will note that those who get vaccinated are less than 200 because these hearsays people are hearing them in their own understanding, not as doctors but in some groups, like in bawo groups, drinking joints, that’s where such things are discussed and there are disagreements, that’s where you say, ‘oooh! If that’s the case, that’s okay’’ then if you go home and something happens, you just tell them, ‘’okay, I have noted it’’ because you have heard that someone took that medication and never got healed, he/she died, ‘’should I die?’’

**Facilitator:** laughing

**00:** it’s difficult for people to believe in something as true, we pretend to understand, just like what my colleague said, when you are in groups, it’s difficult. You may discuss and someone may say, ‘’You will never give birth to children with these injections’’ (cross-talk)

**00:** (not clear) Do you have evidence that someone is not giving birth to children, you will hear them say ‘no’ but you have just believed it. Some people even say it’s 666, once you are vaccinated, that’s 666, it’s Satanism

**Facilitator:** which vaccine do they refer to?

**00:** COVID vaccine, they say once you receive that vaccine, you have received the 666 mark

**0:** in short, it’s everyone, my child who came here (not clear) so they connect

**Facilitator:** what do they connect vaccination with?

**0:** Satanism for example

**00:** you and that group are connected (cross-talk)

**0:** that child of mine who came here, one day he/she came home, ‘’Come here, vaccine team came to our school, wanted to give me polio vaccine, and they said if you are not vaccinated, you face this and that’’ we said that’s how it is. Some vaccine teams came to our communities, they were at Nthemba

**Facilitator:** mm

**0:** so they came and found that child home, ‘’We are giving the vaccine to children of this age range’’ the child said, ‘’Give me the vaccine’’, and the child told us, ‘’They gave me some drops of the vaccine in my mouth’’ we did not shout the child, we said, that’s good decision because you would have faced problem on your own. We can encourage children as well, but, when the children are going to school, they already know what to do, ‘’when the vaccine team comes, please run away’’

**Facilitator:** you are saying people talk much about the COVID-19 vaccine, what do people say about the vaccines that have been there before? Have we started receiving the vaccine yesterday r during COVID?

**All:** no

**02:** I want to testify and I can show you here. I received the vaccine for smallpox. I was born in 1985, and previously, were receiving the smallpox vaccine and my age mate maybe these ones. We were receiving the measles vaccine and other diseases. This has not started today…

**0:**  (there was polio 1, polio 2

**02:** now, we have got the wrong mentality but, vaccines started a long time

**Facilitator:** where is this wrong mentality coming from? And how does it affect the recent vaccines?

**000:** we are in these days, there are many hearsays that make people go mad, so it is difficult to understand these things because there are a lot of hearsays

**Facilitator:** we would like to hear your views on what people say regarding the vaccines. I think children were not receiving the COVID-19 vaccine, what do people say about under-5 vaccines?

**04:** the issue is what this one said regarding children's vaccination. They say the problem is that we are overpopulated…

**0:** (talk it properly

**04:** I am going there

**All:** laughing

**04:** when the children receive this vaccine, they will not give birth to children, that’s what parents think

**0:** us, we are parents

**04:** once a child receives a jab, that child will never give birth, so we don’t know the issue of death, the issue is, when the time comes for a child to get married, he/she will never get pregnant and the male one will never make his wife pregnant….

**000: (**before he proceeds, you gave birth but, is what parents think true or not?

**Facilitator:** let me plead with you, if possible answer him at the end because at the beginning we said that there is no right or wrong answer. Research is like a net in the lake, when they throw a net, it brings everything, and you have time for separating stones and fish. Even when we are harvesting rice, the same applies. So we will have time at the end but now, let’s discuss everything whether it’s true or not

**04:** this one said he has a vaccination mark, I have too, and it’s true that we are age mates. What I want to say is that, previously, we didn’t know that we were receiving the vaccine. Our parents were just carrying us on their back and going, ‘’It’s today!’’ they were going in groups, ‘’This is a third one, and there is the last one! And after that, we will never go to the under-5 clinic.’’ Parents were doing that, but today

**All:** cross-talk

**03:** I am taking what this one has said, he is the one who said we should abandon norms from parents. Aren’t they the same parents who were following the vaccination, taking a child to the clinic for polio vaccination?

**All:** laughing

**03:** he was agreeing right?

**00:** everything has its advantages and disadvantages, we have said parents had their medication and they were saying this sickness, I should give this to the child (cross-talk)

**Facilitator:** let’s speak one at a time, this is interesting

**00:** do we have such things today?

**0:** no

**00:** who ended it? The same parents

**0:** eeh

**00:** didn’t your parents tell you that we don’t want that girl

**0:** they did

**00:** didn’t you not insist and have a problem with that woman?

**0:** it has happened, parents have a problem, if we follow them there are good things they were doing, they were taking us to the hospital up to the stage he is saying, we have finished, ‘’I am done with you’’ they are focusing on another child. The same parents had another habit when I had a headache, they had their own medication, can you do that today?

**00:** aah no

**0:** are you abandoning norms from parents?

**00:** yes

**0:** why?

**00:** it’s bad

**0:** aah, I am saying let’s not leave everything, let’s take the good ones, you don’t abandon everything, you take the good ones and leave those that are not good

**Facilitator:** okay, this is an interesting debate, I want to watch it like traditional dance

**All:** laughing

**02:** let me ask him, things are changing daily and every day, the hospital laboratory is busy researching medication for certain diseases. Our parents then just say, ‘’You have a headache, this is a herb for headache’’ that’s all. Things are now changing daily, so we can abandon some of the things and see where we are going

**Co-Facilitator:** This one talked about social media, how does it affect vaccine information? You just started it

**0:** you know what

**Co-Facilitator:** mmh

**0:** (not clear) Even these research issues, have been announced on the radio, ‘’people will come to your community to tell you or are doing such a research,’’ and there are social media, this issue we are discussing, ‘’what do you think?’’ his opinion (not clear) that’s how social media is working. There is another social media which is a trap (not clear)

**Facilitator:** Alright, on the issue he was talking about, that the vaccines aim at making children not to give birth to children. I would like to know, is there a specific vaccine that they refer to or every vaccine?

**00:** if you have heard him, he said that it is a threat for a child to receive the vaccine or injected than those who should receive the vaccine of their age (not clear) that’s what makes people think, ‘’Aah they want us to be like them, if you have 2 children, that’s enough’’ government is saying people are overpopulating, we just speculate, M’dala is done, he is not continuing

**Facilitator:** before COVID came, how people were receiving the vaccines here in Bangwe before COVID?

**0:** people were receiving the vaccines depending on what that vaccine was all about, for example, before COVID (not clear) when we talk about COVID

**Facilitator:** mmh

**0:** it was a must, whether you wanted it or not, that’s what made people talk

**Facilitator:** what was motivating people to go receive the vaccines previously?

**04:** what motivated people to receive the vaccine was that pregnant mothers were going to the clinics, and when they went there, they were receiving counseling on what to receive during that period up to the time of deliverance. When a child was born, the same procedures were followed until they finished that, so it was not difficult to let’s say someone said, ‘’When you go to the hospital, the doctor will refer you to us when he/she is done with you so that you will hear what we would like to tell you and it will be up to you to agree or not, you have a right to accept or not.’’ Then I went to the hospital and I was referred to you, ‘’I had diarrhea and I am referred to you to deal with this problem, ‘’ then you are telling me, ‘’for you to get rid of this illness permanently even if you eat something bad, you need to do this, ‘’ then I say ‘’aah no, that’s not true’’ or, ‘’that’s true’’ it is only you and me who know this, so you inject me or I have refused and go. What was happening previously, when people went to the hospital, everything ended there, they were injected and go

**Facilitator:** I would like us to focus on accepting or refusing, so I want to know, did people previously accept the vaccine or not? If they were accepting it, why?

**00:** people previously were receiving it, what came later as you know, a father is the one who is stubborn in the household because he feels that he is a leader. I should agree with that one, when that campaign came, that husbands should accompany their wives to the ante-natal clinics, it helped a lot because when a man goes there and received counseling, there was a mind-set change, he was able to say, ‘’what I was hearing in bawo was not true, let’s follow this. ‘’ that was helping (not clear) you will notice that previously, people could give birth to a disabled child and were kept indoors, because of counseling that had no threats like what you are doing, people were able to receive the vaccines. You also know that black people, seek medication when the condition is worse

**All:** laughing

**Facilitator:** the issue of awareness has been coming, this one is repeating the same issue. I would like to know, what awareness do you think is lacking on the issue of vaccines that you think those who are ignorant should remain behind on this issue of vaccines?

**02:** I think there was an answer to this question even though you are asking it. There was a need for a door-to-door sensitization for people to be told, ‘’Ladies and gentlemen, we would like to protect the newly born babies, they are presidents for tomorrow. There will be such and such things coming, don’t get afraid, receive and accept them so that children live longer. These vaccines don’t aim at killing children when they are 5 years old or make them barren, it’s not like that. ‘’people should be sensitized and that’s why, long ago Kamuzu used to say school never ends even if you reach form 4 and go to the college, you still need to learn something, someone from standard 8 will come to you a form 4 student, ‘’we have come for this reason to deal with such disease, please listen to us and follow this advice,’’ that person will get something

**Facilitator:** mmh

**02:** that’s it

**00:** for example with the family planning vaccine, women were hard but in the end, men accepted it. When our parents were giving birth, they were thinking, ‘’I have a plot, maybe these plots will be taken by such a person or I am giving birth because I have food and the like’’ yet this time, if you take that into account, it will be difficult because that advice came through radios and social media, it was good. For someone to change his/her mind, there should be a good approach, and not a political approach, it is received well and the person receives it well. Just like if I have made a mistake and you ask me when you are angry, I cannot accept it, because I will know that if this one is angry, let it be. So there should be a very good approach, I think we will not be surprised with some of the things

**Facilitator:** you agree with what he said about the door-to-door sensitization campaign

**00:** yes, there was someone who I cannot mention his/her name. When we went for family planning, he/she said no, this is not good method, use the natural one, we haven’t do that yet, he/she is the one receiving money, telling people to do this and that yet he/she is not practicing it

**Facilitator:** what natural methods was he/she talking about?

**00:** for example, you are talking here but behind, you say no to this, I said because of was at work (cross-talk) laughing

**Facilitator:** I am just been sent

**00:** yes, for my children to have food, it’s because of this, I have no option but what I can tell you is that follow this one. You want to show that even if you have said that but (not clear) I have evidence but I cannot tell you who said this but it is happening, and that’s why these things are difficult

**Facilitator:** mmh like you are giving them two different messages

**00:** Yes, like telling someone, this is medication for madness, then you say Aah my friend (cross-talk) same as when HIV just came, I remember my uncle, we used to call it ‘’kanyera’’ sitting in the sun and the like, many people died in the years of 2000s. once a patient had diarrhea, he/she had gone, there were many coffin-selling points along Bangwe road but, when the message came, people started going and receiving the drugs and the like, and that reduced, even (not clear) told me, ‘’the patients refuse but, tell the patients that this is your life and if you refuse these, you better die, so choose’’ you could control a patient, so the counseling should not be political as I said, telling us that I am doing this because it’s my job

**Facilitator:** ooh that’s what you meant when you said it should not be political

**00:** exactly, because you are employed and your job is going on, your children say my father has gone to work, In he is in Bangwe, after you we will say, gentlemen, be careful

**All:** laughing

**Facilitator:** number 6 had something to say

**06:** as parents, we have responsibilities, if we are learning here, we can go encourage women who don’t know to say, ‘’Let’s do this and that’’ If they understand, they will tell their friends

**Facilitator:** we have discussed many things about the vaccines, let’s get back to where we started from, we are about to finish, sorry it’s been a long time. When people get sick, where do people seek treatment here in Bangwe?

**02:** they go to a clinic, we only have one clinic, Mpingwe, Naizi, BCA, Railways, and Namatapa, we have one clinic and that’s why, you asked what we can do if someone has diarrhea, first of all, we give first aid at home and after that, take a patient to a clinic because the clinic is far, for example, someone stays in BCA, they cannot just take the patient and walk all the way to a clinic without water, you need to have a solution of water and sugar like glucose and administer it along the way to a clinic, so we have one clinic

**Facilitator:** apart from the clinic, is there anywhere people go to access help?

**0:** we have a pharmacy at Shares in Railways, if you experience some symptoms, you go there and explain. There are doctors there, so they ask, ‘’Did you go to the hospital?’’ ‘’I did not go’’ ‘’okay I will help you’’ so they help you accordingly

**Facilitator:** pharmacy and hospital, is there anywhere else?

**All:** Silent

**Facilitator:** we hear some buy from the shops, doesn’t it happen here in Bangwe?

**0:** it happens but, only those who have money manage to do it, but if you don’t have something in your pocket, you cannot go to the pharmacy, you will wait until morning and go to the public hospital, so it’s a big challenge

**06:** we rush to buy drugs as you are saying but, we can buy drugs only to notice that we are not healed, then you go to the hospital and ask, ‘’Did you take any medication?’’ when we say yes, we are wrong, we should not take medication, we have to go the hospital for treatment. When they examine us, they don’t diagnose any illness because of the drugs that we took

**Facilitator:** you have talked about rushing to buy drugs, how do you decide that you should go to the clinic or you should buy drugs?

**0:** situation (cross-talk)

**00:** let’s say you once got sick and you went to the clinic and were told, we have no medication for that stomach-ache, go to the pharmacy to buy, so if you get sick again, you think, ‘’Last time I was told there was no medication, ‘’ I better go the pharmacy and buy.’’ (Not clear)

**Facilitator:** what distance do people travel especially those who live far away, how long do they travel to get to the Bangwe clinic?

**00:** for us who live in Railways, we go to Limbe or Queens because for you to travel by railways….

**0:** talk of BCA, there is no clinic in BCA and Namiyango, they come here (cross-talk) people from BCA go to Limbe, some go to Queens, those who live in Mpingwe go to St. Patricks in Nzedi but those who live at number 2 and number 3, I have been meeting them at this clinic

**Facilitator:** maybe on distance you can think of kilometres, but we can think of transport one may use

**0:** if you use a bicycle, it’s 1.5. it used to be K1000 but, it’s now K1.5

**Facilitator:** is it one way?

**0:** yes

**00:** K3000 go and back

**0:** it also depends on the situation

**000:** There will be 2 of you with the patient

**0:** carrying a patient is expensive, that price is not enough (cross-talk), or am at easy, I will use a minibus

**00:** if I am sick, but I don’t have money for transport, it’s not possible to walk from there to a clinic

**Facilitator:** what do you do if you don’t have transport and you cannot walk?

**00:** that’s what he said, if you have 1,500 you go to the pharmacy (not clear)

**000:** what happens in our hospitals is that we can go there and be told, ‘’this condition, go to Queens’’ yet you have no money for transport to Queens, you then go back home

**Facilitator:** drug shortage, lack of money, and long distance, are things that make you fail to go to the hospital

**000:** Exactly

**00:** in some areas, there are health workers. We are considered city residents, if we had health care workers closer, people would have been going there to access treatment when they get sick, but we are considered city residents, if you go to Namatapa, with wind that has just passed, there is flue, but you will find many patients, but you will find that there is no medication, ‘’go to the pharmacy’’ (cross talk)

**0:** the problem is that we rush to a new things, like the newly diseases, you can be easily assisted, if you can help us, it is difficult for people in the villages to go to the hospital, as health workers, you know the diseases, if you do like what Covid team does, going to schools and announce, ‘’those who have such conditions, we are meeting at a such place for medication

**00:** Previously, healthcare workers used to conduct a census, some will be here, another one there

**0:** these things started way back (cross-talk) they stopped

**00:** they were many, but now they stopped (cross-talk) not clear..that makes a patient go to the pharmacy

**All:** laughing

**0:** you go to the pharmacy if you have money

**00:** After discussion, you hear them telling you, ‘’Tomorrow bring that lady you were with!’’

**All:** laughing (cross-talk)

**0:** it cannot come to the public yet you discussed it privately, I can sue him/her

**000:** when you go there, they tell you to go with your partner

**00:** gentlemen, we are bringing more issues here, whether a doctor is male or female when the doctor helps you privately, cannot come here to tell you, ‘’Gentleman, it’s tomorrow!’’

**0:** I went to do circumcision, I was injected, but he/she passed by and said, ‘’Don’t forget, it’s tomorrow!’’

**All:** laughing

**Facilitator:** I cannot refuse that because we meet with different scenarios, what somebody faced, we have learned that it can happen. I want us to finish with the drugs that we buy on our own after prescription. What are they?

**02:** when the child had a cough, I took her/him to a clinic and told them I should buy amoxicillin, and when I went to buy, there was no amoxicillin, so I bought Bactrim. I accepted because it was a doctor who told me and after 3 days, the child was okay

**00:** I took my daughter to the hospital, I was told she had insufficient water….

**Facilitator:** (not only prescription, include that you decided on your own, like what he said, ‘’When I got sick, treatment was prescribed and the next time I had the same symptoms, I just bought the drugs.’’

**000:** when my child has flue and cough, I buy drugs on my own

**Facilitator:** what kind of drugs do you buy?

000: penicillin

**Co-Facilitator:** do you know antibiotics?

**Facilitator:** ever heard the word antibiotics

000: I know soap like Protex when my child has skin rashes, I buy a black Protex

**Facilitator:** do you know the drugs called antibiotics?

00: we just heard about it, but we have never seen it

000: there is another one we mix with water

0: Delto

000: yes, I have been buying it

**Facilitator:**  you bought Delto for what reasons?

000: skin rashes

**Facilitator:** which means many of you don’t know the antibiotics

000: yes

0000: I had skin rashes, and they still itching me up to now, I went to the hospital, (not clear)

**Facilitator:** what do you do on your own?

0000: I just use soap, so I use soap when bathing, but I have been receiving different medication from the hospital including those that are applied on the skin, it never improved

**Facilitator:** Let’s finish our discussion, it’s been a long ago and we thank you for your attention and flexibility to contribute, Are there any last comments?

**Co-Facilitator:** it can be a question or anything else

**Facilitator:** I will respond to that question you asked me, but I want to give you a chance

**00:** we are thankful, I have added knowledge through this one and that one in this discussion. Secondly, I don’t what the end of this training

**Facilitator:** ooh, you have called this training?

**00:** we have learned

**Co-Facilitator:** you had come late (not clear)

00: thank you

**Facilitator:** anyone who is remembering about the end of this discussion should answer

**0:** (not clear) They are conducting research on diarrhea in children (not clear) Mostly they were asking what we do as parents, (not clear)

**00:** mmh

**0:** I thank you, as he said, it is research yes, but we have learned something, and wherever, we will be explaining here and there (not clear)

**Facilitator:** thank you, just to add what he said, this is research, so the group discussion is aimed at knowing what people know, what they do, or what they would like to see happening as far as diarrhea is concerned. The aim is to enlighten each other (not clear) it is a government that has a responsibility to ensure health issues are going well here in Malawi. The government believes that peoples’ views should be followed when making health policies, so we were discussing diarrhea, how it is caused, and the current vaccine. If there is a problem, what should be done, so you have expressed your views that may encourage others to go receive the vaccine? On the issue of purpose for this research, this discussion that has taken 2 hours has ended here but, the research is gin on because we will discuss it with other groups, with you today, we are done. It can be possible to come back after listening to the discussion and note there is something that we did not understand, so we can make a phone call, ‘’Let’s meet, there is this issue that came out of that discussion,’’ that can happen, for today, we have finished. Have I answered the question?

**00:** you have answered it, but let me speak on my own behalf. Aside from that, I am not employed, if there is something to do, include me so that I can accompany you

**All:** laughing

**Facilitator:** thank you

**00:** I have expressed my views

**Facilitator:** good opinion, thank you

**00:** I am looking for an employment

**Facilitator:** thank you, this one asked a question and he said, when a child is receiving the vaccine, the aim is to make that child barren, so you asked, ‘’Is what he is saying true?’’ was it a question?

**00:** yes

**Facilitator:** In this research, we are conducting, we believe that what someone is saying is true, understand me on this. We say there is no wrong or correct answer because we don’t know why the person is talking like that. For the vaccine to be given out to people, there are procedures that are followed. In that paper I have given to your friends, there are organizations, here we have the Ministry of Health, the World Health Organisation, we have Centre for Disease Control from America, and we have another one in Africa, the Centre for Disease Control Africa and the like. It is the duty of these organizations to make sure everything concerning health is going on accordingly. On the issues of vaccines, they monitor it, vaccines have stages, there are those who manufacture the vaccine being it the laboratory or, after the manufacturing, before it is given to everyone, and they test it this happens in stages as well. It can be tested on animals, and from there, the vaccine is taken to people, that’s why you see research. Before the Rotavirus vaccine was given to anyone, they took a sample population for a test, to see how this vaccine is functioning in children’s bodies. After running that test, the results are analyzed by the organizations to see what happens when the vaccine enters the body of a child. If they see there is nothing wrong with it other than protection, they say this vaccine is good. If they are satisfied with the procedures, from manufacturing through to the pilot stage, when everything is okay, they recommend the vaccine to be given to people. Then we say every procedure was followed, and there is an assurance that it is good. What someone is saying is that when you give this vaccine to a child, it will make that child barren, as a researcher I don’t have an answer to whether it’s true or not, it is good to hear that opinion however because whether it is true or not, it affects someone’s decision making on the vaccine, which means we need to do something to approach that person with such opinion and hear where such opinions come from, that’s why we have come here for discussion, it helps government to say, ‘ooh there are some people who think about this vaccine, let’s approach them and ask.’’

**Co-Facilitator:** some just read from the Facebook, some just wrote it

0: people can just write things

END OF INTERVIEW
