# Supplementary material for: Exploration of the social determinants of diarrhoea, rotavirus vaccine uptake, and vaccine ‘fatigue’ in Ethiopia, Kenya, and Malawi
Source: PLoS One. 2025 Sep 9;20(9):e0319691. doi: 10.1371/journal.pone.0319691 (PMC12419581; doi:10.1371/journal.pone.0319691)
Supplement: S1 Data — (ZIP) [file pone.0319691.s001.zip › Supporting Information Files/KY_11FGD.docx]

**FOCUS GROUP DISCUSSION 11**


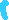


**30/04/2024**

**NUMBER OF RESPONDENTS-10 FEMALES.**

**1. Can you please tell us some of the illnesses that affect children in your community?**

R1-Cholera or diarrhoea.

R2-Pneumonia

R3-Malaria.

R4-Skin diseases.

R5-High fevers.

R6-Coughing and vomiting.

R7-Sometimes the children have loss of appetite.

R8-Kwashiakor, sometimes parents are not able to feed their children a balanced diet and feed them whatever they have. This leads to diseases like kwashiorkor.

**2. Which of these illnesses do you consider to be a burden in this community? Why do you say**

**so?**

**R3-**Diarrhoea.When a child has diarrhoea and is vomiting a lot they are likely going to become weak in a short while.

R2-Diarrhoea.

R8-Common flu, the child secretes a lot of mucus and sometimes tears too.

R7-There are some children with congested chests and have difficulty breathing because of the cold especially now during the cold season, the cold affects them and makes it difficult for them to breath.

R2- Flu and congested chests which increases their fever.

**3. If you were to rank these illnesses in order of priority, what would you rate as the top three**

**diseases affecting children**

**[If diarrhoea is not listed in the top three priority problems -Do you think diarrhoea diseases**

**is a big problem in this community or not? Why do you say so?]**

**R3-**The first is diarrhoea and vomiting and the second is chest congestion and flu and the third is pneumonia.

R6-The first is cholera and diarrhoea, the second is pneumonia and the third is flu.

R4-Diarrhoea is very common, then there is coughing. Diarrhoea in children is very common in the community and we do not know the causes of it.

R2-Diarrhoea in the community may be caused by lack of cleanliness in the community. The children are exposed to dirty environment when playing and this causes their diarrhoea.

**4. Can you tell me the health services/facilities available in this community? Where do you**

**access health services? [Probe: how much does it cost to access these services, how long do**

**people have to travel to access the services?**

**R6-**I take my child to EF because the services there are free and affordable.

**R1-**I take my child to Maendeleo because I don’t live far from it and their services are free and good. For instance, if you take a child who has diarrhoea there they are given priority in treatment.

**R8**-I take my child to Prisons in Remand because the services there are good and they never lack medicines.

**R9-**I take my child to Maendeleo Hospital or Our Lady of Nazareth.

**R7-**I take my child to Our Lady of Nazareth in Njenga because the services there are free and treatment there is quick.

**R10-**I go to Kware Dispensary because the services are good and they attend to the child quickly especially if it is a case of diarrhoea.

**R2-**I prefer taking my child to Maendeleo Hospital because their services are good and free especially if the child has diarrhoea.

**R3-**I take my child to Kware because the services there are great.

**R4**-I take my child to Maendeleo because it is close to where I live and the hospital has medicines and there are laboratory services so one is able to know their cause of cholera.

**5. How do most people respond when a child has diarrhoea in the home? [Probe: What do**

**people do at household level? at community level? Where do they go to access treatment? Do**

**they take antibiotics? Where do they access antibiotics? Why do they access antibiotics?]**

**R6**-Sometimes we rush to the chemist because it is during the night and you cannot go to the hospital. There we are given some medicines to help us through the night so that in the morning we are able to go the hospital.

**What do people do at household level? at community level?**

**R5-**I boil water with some sugar and salt, cool it and then give it to the child.

**R7-**I give the child water in plenty because the more they diarrhoea, the more water they lose.

**R1-**I usually cook a little porridge with wheat flour and give them and give it to the child with water.

**R10-**If it happens at night, I boil some water, sugar and salt and give it to the child and then take them to the hospital in the morning.

**R3-**I cook some wheat flour porridge and give the child or give them any medicine that I have in the house.

**R2**-I was told by my parent that the diarrhoea is sometimes caused by teething so I scrub them with a plant called Nderema.

**6. Can you tell me some of the enablers and challenges that people experience to access**

**treatment for diarrhoea diseases?**

**R10-**We are not sure whether we might get any income everyday as we are casual laborers so finances become a challenge.

**R5**-Lack of employment and income becomes a challenge and so accessing treatment for the child becomes challenging.

**R9-**Finances are a challenge most of the time because our income is insufficient.

**R6-**Security is a challenge because you might be attacked on the road at night.

**R4-**The challenge is finances because of our low income and so we do home remedies in times like this.


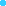


**7. What do people do to prevent diarrhoea? [At household level, at community level?]**

**8. How do people in this community perceive childhood vaccines [Probe: why do you think**

**childhood vaccines are widely accepted? Why do you think childhood vaccines are widely**

**resisted?**

**R7-**I have never asked the doctors or nurses what vaccine they are giving my child but follow whatever is written in the child’s clinical book.

**R8-**I asked the nurses about these vaccines and they told me that it protects the child from various diseases. For instance, the rotavirus vaccine protects children from zero to two years from diarrhoea.

**9. How about rotavirus vaccines? What do people think about rotavirus vaccines? Where do**

**they access rotavirus vaccine? [Probe: What do they think are the benefits of rotavirus**

**vaccines? What concerns do people have with rotavirus vaccines?**

**R6-**It is a vaccine that protects the child from diarrhoea.

**10. What are the enablers and challenges for people in this community to access rotavirus**

**vaccines? [Prove: cost, distance to access services, cultural/religious beliefs, impact of**

**COVID-19, perception of vaccine safety]**

**R2**-I received the COVID-19 Vaccine.

**R8-**There are people in the community who told us that once you receive the COVID-19 Vaccine you turn into a zombie.
