# Supplementary material for: Exploration of the social determinants of diarrhoea, rotavirus vaccine uptake, and vaccine ‘fatigue’ in Ethiopia, Kenya, and Malawi
Source: PLoS One. 2025 Sep 9;20(9):e0319691. doi: 10.1371/journal.pone.0319691 (PMC12419581; doi:10.1371/journal.pone.0319691)
Supplement: S1 Data — (ZIP) [file pone.0319691.s001.zip › Supporting Information Files/KY_01FGD.docx]

**FOCUS GROUP DISCUSSION 1. VENUE :MUKURU KWA NJENGA**

**9 PARTICIPANTS, 6 FEMALE& 3MALE**

**Moderator: Tell us some of the illnesses that affect children in your community.**

1. R1. Malaria
2. R2. Cholera
3. R3. Flu/Cold
4. R4. Pneumonia
5. R5, R1. TB
6. R2. Typhoid
7. R6. Diarrhea
8. R7. Asthma
9. R4. Meningitis
10. R1. Cough

**Moderator: Tell us the Three priority high-burden diseases in this area**

**R1**. Cholera

I consider cholera as the most burdening disease in Mukuru informal settlements. The water we use for drinking is not clean and my child suffered from cholera just the other day. I had to rush her child to the hospital after several episodes of diarrhea.

**R2.** Malaria

It took 7 days for my child to be fully treated because I took my child first to the nearby chemist and was given the wrong medication. I had such a bad experience and I cannot advise any parent to take their child to the chemist to buy medications without consulting a doctor first.

**R3.** Diarrhea

For me, it is Diarrhea because, Mukuru informal settlement has poor water and sanitation, there are open sewers everywhere and that is still the play area for children. There is a recurrence of diarrhea among children, especially under 5 years.

**R4**. Pneumonia

My child developed a breathing problem, a headache, and a cough. It took so long to be fully treated

**Moderator: Can you tell me the health services /Facilities available in this community?**

**R1.** I go to NMS Maendeleo Hospital because it is closer to where I live and the services are free. They do not have medication. After consultation, you are sent to the nearby chemist to buy medication but I prefer to go to town because it is cheaper there inclusive of transport

**R2.** I prefer EF Hospitasl because all services are available including laboratory services and medication. The only challenge is their services are charged meaning you cannot access the services if you don’t have money.

EF Hospital is not far from where I live (Diamond) so I just walk to the hospital. I don’t incur transportation fees.

**R3**. I go to either Quarry Hospital or MMM Hospital, I discovered the Quarry Hospital recently but previously I used to go to MMM.

I visit Quarry Dispensary only for immunization, most of the time it is very congested meaning you take so long to get the services. Healthcare services are available and free of charge.For MMM Hospital, you will pay but you will get all the required services.

**R4.** I also prefer MMM Hospital because patients are not many therefore faster service delivery. All services are available and it is near my home

**R5.** I take my child to Mukuru Health Centre. It is a bit far but it’s the nearest health facility. If I choose to use a motorbike it will cost me one hundred shillings. I don’t have that one hundred shillings so I do walk to the hospital.

Drugs are not available most of the time, you have to buy them from chemists that are adjacent to the hospital. Same case for laboratory services. It is like the clinicians have a deal with the private owners of the chemists.

You can take 45 minutes to walk to the hospital (MCC)

**R6.** My child once suffered from pneumonia and I took the child to MMM where is got treatment and she is now well. I did not pay for transport since I walked to the hospital.

**R7.** During outbreaks, there are outreach programs for door-to-door vaccination.

**Moderator: How long do people have to travel to access the services?**

**R1**. I walk for like 20 minutes to get to the hospital

**R2**. I walk for 5 minutes, it is not far

**Moderator: How do most people respond when a child has diarrhea in the home**?

1. **At the household level?**

**R1**. If my child has a high fever, I will take him/her to a cold place

**R2.** I saw my grandmother massaging the baby’s tummy, and since it is not safe to go outside at night we had to wait till the next day to take the baby to the hospital.

**R3**. You mix warm water with salt and sugar then give the mixture to the baby

**R4**. You boil water and give it to the baby while it is still warm

**R5**. I went to the hospital and I was advised that ORS and zinc are the first lines of treatment for diarrhea to avoid dehydration. I always keep them as first aid.

**Moderator: Can you tell me some of the enablers and challenges that people experience to access treatment for diarrhea diseases?**

1. **Challenges**

**R1.** My child had a chest infection, I took her to the hospital and I was just given the prescription so that I can purchase it from a private chemist. One was going for 800 shillings and I am supposed to buy 3 totaling to 2400 shillings. I did not have such an amount of money. Sometimes, you are given an alternative to that drug which will be cheaper but again you might compromise on the effectiveness if you choose the cheaper alternative.

**Moderator. What do people do to prevent diarrhea**

**At household level**

**R1.** Always wash your hands after visiting the toilet.

**R2.** By boiling water.

**R3**. We wash our vegetables before cooking.

**At community level**

**R1.** At my church, on some Sundays we have CHVs coming to our church to talk about vaccines and diarrhea prevention

**R2**. In my church also, we are taught the importance of boiling water and eating healthy foods

**Moderator:** **How do people in this community perceive childhood vaccines**

**R1**(a father) I know there are childhood vaccines given to children but I don’t even know them.

**R2**. I know about polio and sometimes during an outbreak there is door to door vaccination campaigns.

**Moderator. Why do you think childhood vaccines are widely accepted?**

R1. They are accepted because it is a preventive measure put in place by the government hence we trust the government and hospitals.

1. **why do you think childhood vaccines are widely resisted?**

R1. Due to myths and misconceptions attached to the vaccines

**How about rotavirus vaccines?**

1. **What do people think about rotavirus vaccines?**

(No one responded)

1. **Where do they access rotavirus vaccines**?

**R1**. During the antenatal clinics, we are taught about the vaccine and where to access them. We get them from the hospital.

**What do they think are the benefits of rotavirus vaccines?**

**R1**. Since the rotavirus vaccination, my child did not experience diarrhea at all.

**R2.** My child has been just fine since the rotavirus vaccination

**R3**. I did not see any effect it had on my child

1. **What concerns do people have with the rotavirus vaccine?**

**R1.** After vaccination, the child might have a fever

**R2**. My child did not have any side effects not even fever.

**R3**. If you find out your neighbor’s child had side effects as a result of rotavirus vaccination you will refuse to vaccinate your child

**Moderator: What are the enablers and challenges for people in this community to access the rotavirus vaccine**

**R1.** We live near these facilities, which are just a walking distance

**R2**. Very few people still hold on to those beliefs, especially the ones from a rural area

**R3**. There are concerns that expired vaccines are given.

**R4**. COVID-19 did not affect our perceptions towards the KEPI vaccines

**R1.** Since the vaccines are given at the hospital we are guaranteed the safety.

**R2.** There are concerns about safety because we were told those vaccines are expired and not safe for us
