# Supplementary material for: Exploration of the social determinants of diarrhoea, rotavirus vaccine uptake, and vaccine ‘fatigue’ in Ethiopia, Kenya, and Malawi
Source: PLoS One. 2025 Sep 9;20(9):e0319691. doi: 10.1371/journal.pone.0319691 (PMC12419581; doi:10.1371/journal.pone.0319691)
Supplement: S1 Data — (ZIP) [file pone.0319691.s001.zip › Supporting Information Files/KY_04FGD.docx]

**FOCUS GROUP DISCUSSION 4. MUKURU**

**10 PARTICIPANTS 6 FEMALE& 4 MALES**

**What are the top health concerns or priorities among under 5 children in your community?**

1. R1. Malaria
2. R2. Cholera
3. R3. Flu/Cold
4. R4. Pneumonia
5. R5, R1. TB
6. R2. Typhoid
7. R6. Diarrhea
8. R7. Asthma
9. R4. Meningitis
10. R1. Cough

**How would you rank gastrointestinal infections in children compared to other health-related issues?**

**R 1) Cholera, Malaria, Diarrhea, Pneumonia**

**What do you think about introducing a new vaccine for other gastrointestinal infections**?

**What information do you need to consider before the introduction of these new vaccines?**

**R1.** I need to know the vaccine manufacturer

**R2.** I need information on the purpose of the vaccine

**R3**. I need to know the safety of the vaccine before my child is given.

**R4.** I would like to know if the vaccine has been accepted by the government

**Q 2.1 Have your children or other children you know been directly affected by gastro infections?**

**R1.** Yes, my child had diarrhea. I took her to the hospital and was given ORS and Zinc.

**R2**. My child had diarrhea and a fever at night; we couldn’t take her to the hospital at that time. We need to wait

*R 3. Mostly, we take the sick child to a health facility. But at times we take them to a nearby private chemist to get over-the-counter prescription because at night it is not very safe. Or we give home remedies and if the sickness persists, we take the child to a health facility*

**Q.2 .2 What challenges have you faced in accessing health care services related to gastrointestinal infections?**

**R2**. I prefer EF Hospital because all services are available including laboratory services and medication. The only challenge is their services are charged meaning you cannot access the services if you don’t have money.

**R5.** I take my child to MCC Hospital. It is a bit far but it’s the nearest health facility. If I choose to use a motorbike it will cost me one hundred shillings. I don’t have that one hundred shillings so I do walk to the hospital.

Drugs are not available most of the time, you have to buy them from chemists that are adjacent to the hospital. Same case for laboratory services. It is like the clinicians have a deal with the private owners of the chemists.

You can take 45 minutes to walk to the hospital (MCC)

**R3.** I visit Quarry Hospital only for immunization, most of the time it is very congested meaning you take so long to get the services.

**Q 3.1 How involved are you in health-related decision-making processes at the community or policy level?**

**R1.** I haven’t been involved in any decision-making processes but I know there are people in the community who have been engaged in such processes.

**Q 4.1 Have you participated in any public consultations or community forums related to health policy changes?**

(all of them said they did not participate in any community forums before.)

**Q 5.1 Are there community organizations or groups working on health-related issues, including vaccine-preventable gastrointestinal infections**

**R1.** Community Health Workers

**R2.** Church groups that are assigned the responsibility of training members on how to prevent diarrhea

**Q 7.1 Do you have any feedback or suggestion for improving the current health policy related to vaccine-preventable gastrointestinal infections**

**R1.** We need to have more forums that can involve as many people in the community.
