# Supplementary material for: Exploration of the social determinants of diarrhoea, rotavirus vaccine uptake, and vaccine ‘fatigue’ in Ethiopia, Kenya, and Malawi
Source: PLoS One. 2025 Sep 9;20(9):e0319691. doi: 10.1371/journal.pone.0319691 (PMC12419581; doi:10.1371/journal.pone.0319691)
Supplement: S1 Data — (ZIP) [file pone.0319691.s001.zip › Supporting Information Files/KY_14FGD.docx]

**FOCUS GROUP DISCUSSION 14**


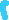


**NUMBER OF RESPONDENTS-5 FEMALES, 4 MALES**

**1.Can you please tell us some of the illnesses that affect children in your community?**

R 1-my child suffered from diarrhoea many times because of the issue of cleanliness and the child not being paid attention to.

R 2. For instance, last week he was suffering from diarrhea, vomiting and had a fever.

R3-Sometimes, they have fever, crying and refusing to eat. As a mother, you become concerned and do not have peace when you see these signs in your child.

R4 My child suffered from diarrhea five months ago

2. How do most people respond when a child has diarrhoea in the home?

R1 -I make a salt and water mixture and give them immediately. Sometimes it might happen at night like an emergency so you give him this remedy and take him to the hospital in the morning. You cannot take him to the chemist because you can’t know what they are suffering from.

R2-I took him to the hospital. First, I took him to a chemist like hospital in a place called Kwa Dennis, then after sometime I realized that he was getting worse so I took him to Mbagathi Hospital. I gave him hot water as first aid at home before taking him to the hospital.

R3. I give my baby Bonisan water and also wheat flour porridge to stop diarrhea

3.Can you tell me some of the enablers and challenges that people experience to access treatment for diarrhoea diseases?

*Enablers
R 1-Government initiatives and policies that focus on improving healthcare infrastructure and accessibility
R 6-A strong community network that encourages seeking healthcare and supports affected individuals
R 7-We are well-informed about the importance of seeking treatment early.

Challenges
R 3-Inadequate awareness and education about diarrheal diseases contributes to delayed treatment.
R 7-Our people may be hesitant to openly discuss symptoms or seek treatment due to societal attitudes or fear of being ostracized.
R 5- Many individuals, particularly in our community, may hesitate to seek treatment due to concerns about the cost of medical care and medications*.

2.Preference method for on Vaccine(Injection or ORal)

*R 1-I prefer injection than oral
R 4-I prefer oral one since the injection one is painful to the child more so at night hour.
R 3-I prefer injection since cultural the injection makes the child strong.*

4.What type of information you need before getting vaccine

*R 4-We need information on the age required before getting a vaccine.
R 5-We need information on the advantages of the vaccine and the side effects of the vaccine.
R 2-Community awareness should be raised before launching the vaccine and should be done by community people whom we trust and who are fully prepared, such as CHVs and medics we know.
R 7-The government should publicly announce the availability of the vaccine, which should have been tested elsewhere and proven successful.*

5.What do people do to prevent diarrhoea? [At household level, at community level?]

*R 3- When the child is sick, I take him to the child.
R 3- I warm water and add salt and sugar for the child to drink, but if it persists, I take him to the hospital*.

6.How about rotavirus vaccines? What do people think about rotavirus vaccines? Where do they access rotavirus vaccine?

*R 4- I don't know the vaccines given to the child; I always give the doctor the child's card, and the kid gets the vaccine.
R 8- I accept the rotavirus vaccine since I have seen how it works on other people and have been told about the side effects of the vaccine.
R 7- I won't accept the vaccine since I don't know how it works*.

7.What type of information you need before getting vaccine

*R 4- We need information on the age required before getting a vaccine.
R 5- We need information on the advantages of the vaccine and the side effects of the vaccine.
R 2- Community awareness should be raised before the launch of the vaccine, and it should be done by community people whom we trust and who are fully prepared, such as CHVs and medics we know.
R 7- The government should publicly announce the availability of the vaccine, and the vaccine should have been tested elsewhere and proven successful.*

8. Are you being Involved in Health related issues

R 1-At times, women get involved specifically during PLW sessions and in making decisions on health.
R 4-I think health-related issues are crucial, and everyone should be actively involved. Personally, I try to stay informed about current health trends and issues, whether it's through news, social media, or attending community health events. I believe that being proactive about our health is not only essential for personal well-being but also contributes to the overall well-being of the community.
R7-I've been involved in a few local initiatives that focus on specific health issues. Recently, I joined a community gardening project that aims to increase access to fresh produce and promote a healthier diet.

9.Can you change the government's decision to recommend vaccination against diarhea

*R 8 - Yes, we can, but at times our views are not taken into consideration.
R 3 - I believe it's possible to influence the government's decision on recommending vaccination against diarrhea through a comprehensive advocacy strategy. We need to gather compelling evidence on the effectiveness and safety of the vaccine, engage with key stakeholders, and present a strong case that emphasizes the long-term benefits for public health*.

10. Channels preferred to use to raise issues on health.

R 1- Working within professional networks is crucial. As a healthcare professional, I often use CHV.
*R 4- We use social media as my go-to channel for raising health-related issues. It allows me to share information.
R 5- We can communicate at the chiefs baraza.
R 7- We can use local radios to communicate.
R 8- We can participate in picketing and peaceful demonstrations.
R 1- Direct reporting to the public health facility.*

12.Have ever participated in health related forums*(Giving opinions)*

*R 8 - I haven't participated much in health forums, but I've been involved in local health community events.
R 5 - We will start going since from this discussion we have gained awareness that our voices matter in such meetings.
11. Reasons for not participating in health meetings.
R 1 - I have never been invited.
R 3 - Usually the meetings occur during weekdays and I am at work.
R 5 - I used to think the meetings were a waste of time*

13.Are you aware of any organization doing health interventions around you.

*R 1-NGOs i.e Amref which does health related program.
R 3-SHOFCO which also does vaccine and health.
R 8-Churches which does health talks during Sunday school*.

13.Changes you wish to be made in health system in relation with Vaccination

***Health facility*** *R 1-Government to add more drugs at the public health facility.
R 3-The Government to secure medicines at the facility i.e security.****Vaccines****R 3-Raise awareness on various vaccines.****How to measures changes from above.****R 3-If there is a meeting and community members have attended fully
R 4****-****Availability drugs at the facility.*

14. Recommendations for the government in policies related to childhood diarrhea

*R2 - The government should provide aqua tabs for free.*

*R3 - The government should monitor water vendors to ensure that the water is of high quality.4*
