# Supplementary material for: Exploration of the social determinants of diarrhoea, rotavirus vaccine uptake, and vaccine ‘fatigue’ in Ethiopia, Kenya, and Malawi
Source: PLoS One. 2025 Sep 9;20(9):e0319691. doi: 10.1371/journal.pone.0319691 (PMC12419581; doi:10.1371/journal.pone.0319691)
Supplement: S1 Data — (ZIP) [file pone.0319691.s001.zip › Supporting Information Files/ET_5FGD.docx]

I: Thank you for your participation. What are the most common diseases affecting children under five in your village?

P6: As we live in condominium with common toilet, there is a problem of hygiene. We have limited access to drinking water and we have to use a toilet for seven or six households, this is why children get vulnerable for diseases.

I: Any other?

P7: Children in our village don’t get their meal at schools in hygienic ways.

I: Tell me the diseases.

P7: Diarrhoea, flu and other. I think the people don’t have that much understanding about the causes and how the children get affected while playing on the field.

I: Any other disease?

P3: There is also tonsillitis, measles and mumps.

I: Okay. Which of these diseases are dangerous for the community?

P5: It is flu. It is because the sewerage system is poor here, people also urinate open. So, the smell coming out affects people.

I: How about in your community?

P6: The most common dangerous diseases in most villages is flu.

I: Please, participate. Can you rank them in order of their danger?

P1: Flu is the first, the second is diarrhoea, and the third is ‘bird’.

I: Any other.

P7: Flu should be ranked first.

P1: Bacteria and stomach-ache.

I: Would you please tell us the health facilities nearby?

P4: Teklehaimanot health center, Churchill health center.

I: How about private health facilities?

P4: Teklehaimanot Higher Clinic.

I: From which health facility you get the health services?

P2: We go to Churchil.

I: Is that because, it is closer to you?

P2: No, Teklehaimanot is closer to us but we just prefer Churchill.

I: Any other?

P7: Teklehaimanot health center.

I: You may also need to list private health facilities.

All participants: *[Silence]*

I: How much do you pay for the services of health facilities you have mentioned, to your children?

P5: We pay around two hundred.

P4: We use health insurance, we go to Teklehaimanot health center.

I: You don’t pay to the services as you are members of health insurance?

P4: We just pay annually.

P7: Most people are currently signed up for health insurance service, so we can buy medicines from there if available. Most of us go to Teklehaimanot health centre for h health services as it is closer for us and we know the stuff members there.

I: Okay, how far is the health facility from your residence? How much time it takes to reach the health facilities?

P1: Teklehaimanot health centre is just located behind my residence.

P5: Churchill is a ten minutes walking distance from home.

P4: It takes 30 minutes from our residence.

I: We asked you these because we want to know the factors, okay. Let’s now get in to new discussions point. What do you do for children under five at home in time of diarrhea?

P4: We took them to health centers as fast as possible.

I: How do you treat them at home, before taking them to health facilities?

P4: We treat them with ‘ Tenadam’ and ‘Yemich medanit’ [cultural leaf uses for Herpes simplex virus [ treatment].

P3: I usually treat her with lemonade, and water from boiled rice, and take her to the health centre if these all don’t work or buy her medicine from the pharmacy.

I: Any other, what do you do at home to treat your children with diarrhoea?

P6: The honey coffee solution is good, most people recommend it.

I: Starting from which age do you do this?

P6: If they are newly born, we treat them with a ‘ Tenadam’ solution with water.

I: Any other?

All participants*: [ silence]*

I: You have told me what you do at home to treat children under five in case of diarrhoea. What do the people in the community do?

P7: Most people prefer to use traditional treatments unless things get worse.

I: Where do you get the medicines from?

P7: From Fitun pharmacy and Desalegn pharmacy.

I: You buy without a prescription?

P7: They just sold us after we described the details.

I: How about you, do you buy medicines without a prescription?

P6: Pharmacies need prescription papers to sell you medicines.

I: When you brought children to health facilities in case of diarrhoea, what were the e good opportunities that made you to go to the facilities and what were the things as a challenges that made you not to go and use the services from the health facilities

P5: What made me not to bring them to health facilities is, the absence of discipline and welcoming reception in Teklehaimanot health center. Teklehaimanot health center is closer to me, but I prefer Churchill as they treat you well and work with discipline. The workers in Teklehaimanot don’t respect patients and even don’t want to talk to you when you go for health services for children.

P1: There is nothing as obstacles to go for health services at health facilities. All I need is just to go for it.

I: Aren’t there good opportunity that made you go for it, or something you don’t like and became obstacles for you not to use the services?

All participants: *[silence]*

I: You have been mentioning the causes of diarrhoea in your village, can you tell me the causes now?

P4: Poor environmental hygiene, not having enough number of toilets and like that.

P5: My child had never been in this situation, but children under a year old use baby bottle, and that creates an opportunity for diseases to have occurred.

I: How about the others?

P6: I think, the major cause is the absence of personal hygiene.

I: What should be done to prevent diseases?

P2: We need to work on personal hygiene.

I: How about methods to prevent it at home?

P7: We need to keep the hygiene.

I: Can you elaborate on that?

P7: We need to keep personal hygiene by washing hands and cleansing utensils.

I: How about at the community level, what we should do to prevent diarrhoea at the community level?

P4: The major problem is the absence of standard way of living and quality houses. We live in scattered way and use toilets in common. We should all east decrease the number of households using a single toilet.

I: Let us focus on mentioning what we can do as a community. We will discuss on the solutions from the third body.

P2: We shall keep cleaning our environment and disposing the trashes well and cleaning ditches.

I: What are your really experience on preventing the diarrhoea, what are you doing?

P6: Health extensions used to teach about that home to home. But, they are not doing that anymore. That was crucial as they used to go home to home to teach and collaborate cleaning sessions. I don’t think they are doing this nowadays. This is a burden of Safety nate program now. I think we should continue this home to home extension works and teach mothers about this matter.

I: How do the community perceive the vaccines, what is their attitude on vaccination?

P4: I think they all have better understanding. All mothers and fathers want to have their children vaccinated.

I: Is there any other different idea?

All participants: *[silence]*

I: Why do you think it is well accepted?

P6: It is because the vaccines are useful go grow a healthy baby since pregnancy.

I: What kind of health benefit does vaccination bring? What are diseases prevented by vaccination?

P4: Polio.

P5: Measles, worms and meningitis.

I: Good. What do you know about the Rota virus vaccine? Have you heard of this type of vaccine?

All participants: *[Silence]*

I: It is the kind of the vaccine you took in three rounds in form of droplets. What is the attitude of the people living in the community about this vaccine?

P6: We just bring them to the vaccine, we don’t know what is what.

I: What do you know about this vaccine? What were the side effects of this vaccine?

P5: I remember the vaccine for measles had the side effect of high fever. Then, after she took the vitamin they prescribed me here, she became better. The rushes on her face were all recovered to normal.

I: OK. How about the vaccine given in droplet form? Where was the vaccine given?

P6: They have been given home to home.

I: The vaccine given in droplet form?

P6: Yes.

I: It would be nice if you asked the name and purposes of the vaccines. Anyways, what do you think are the challenges of this vaccination for the community? It could be cultural or religious teachings against the vaccine.

All participants: *[Silence]*

I: How about the COVID pandemic, wasn’t that a challenge to the vaccination?

All participants: *[Silence]*

I: What do the communities think about the safety of the vaccine?

P6: People may not accept it the first time as it was new, but the safety wasn’t that much questionable.

I: Thank you, I am done asking. I don’t get that much satisfying discussion but you can add if you have something. Have a good time.
