# Supplementary material for: Exploration of the social determinants of diarrhoea, rotavirus vaccine uptake, and vaccine ‘fatigue’ in Ethiopia, Kenya, and Malawi
Source: PLoS One. 2025 Sep 9;20(9):e0319691. doi: 10.1371/journal.pone.0319691 (PMC12419581; doi:10.1371/journal.pone.0319691)
Supplement: S1 Data — (ZIP) [file pone.0319691.s001.zip › Supporting Information Files/MW_9FGD.docx]

**Facilitator:** as I said earlier, there are several issues that I would like us to discuss. We are going to discuss health problems for the under-5 children here in Bangwe. We are going to discuss diarrhoea as I said, how it is caused and how we prevent and treat it. We will discuss where people access treatment when they get sick. We are going to discuss the vaccination as well. First of all, let me start with health challenges that affect people here in Bangwe, what are they? Everyone can start

**09:** diarrhoea

**02:** most children are affected by malaria and headache

**05:** pneumonia

**Facilitator:** if you lower your voice, it will not be recorded

**05:** I am lowering it because I have a louder voice

**Facilitator:** Okay

**07:** body pain

**06:** they get sick from stomach-ache due to lack of hygiene at the household and the poor toilets that are available in our homes

**Facilitator:** so you are telling us the reason why children suffer from diarrhoea in this area

**06:** let’s now focus on the diseases that affect children commonly here in Bangwe

**09:** different diseases?

**Facilitator:** every disease

**09:** flue, most children are affected by flue

**04:** there was an outbreak of scabies in the recent past that affected children

**10:** measles, does not affect most children, but it affects some

**09:** Organ failure as a result of lack of vaccination

**Facilitator:** is that common here?

**09:** no, it’s not common here

**Facilitator:** is it a condition that can affect children?

**All:** yes

**09:** it affects children if they do not receive the vaccine properly

**02:** another disease that has come back after a break is scabies, it is so common

**00:** I would like to add on the issue of scabies, they are available when the children get sick, we should rush to the hospital

**Facilitator:** you have mentioned many health problems like diarrhoea, malaria, body pain, fever, pneumonia and scabies

**0:** skin rashes as well

**00:** chicken pox

**All:** cross-talk

**09:** skin rashes also affect adults, they itch too much, so they are transmitted to children

**Facilitator:** Okay

**00:** chicken pox is an issue here

**Facilitator:** I know that every disease is **a** health problem, but I would like us to mention the health problems that are very serious here in Bangwe, what are they?

**0:** chicken pox

**09:** diarrhoea

**02:** I can put them in three categories, diarrhoea, flu and malaria

**0:** and ringworms

**Facilitator:** this one has grouped them into 3; diarrhoea, malaria and flu. Let’s hear from others

**11:**  scabies

**Facilitator:** Among the three diseases that were mentioned, which can you substitute with scabies?

**11:** I have just added

**09:** she has just added because malaria and flue are very common, sometimes a child may get examined, and not diagnosed with malaria, what is common now is an infection

**Facilitator:** I want us to agree, that if these conditions may be grouped into three, what can be they? We are not denouncing other ones, but the most important ones, let’s say the government wants to intervene today

**03:** I am in support of diarrhoea

**07:** malaria as well, malaria is dangerous to children

**00:** flue as well

**Facilitator:** let’s start with diarrhoea, what makes it a serious health concern? This one started, what did you say?

**06:** lack of hygiene practices and toilets (not clear)

**Facilitator:** mmh what else do you think is contributing to diarrhoea issues here in Bangwe?

**03:** just to add to what he has said, it seems this issue has focused on hygiene practices. Here in Bangwe, diarrhoea is common because we are overpopulated. In the past, houses here in Bangwe were scattered, now if you go to Ntopwa and Namatapa and see how congested houses are, it shows that if a child has diarrhoea, it is simple to be transmitted. Apart from it being a disease that started long ago, it is very common now because once it starts, it affects a large area because we are overpopulated and hygiene practice is an issue already, that’s the first part that I would like to comment on the issue of diarrhoea here in Bangwe. We are overpopulated, houses are closer apart and lack of hygiene practices. Another issue is the lack of advice on how to prevent such a disease, we have seen healthcare workers' community sensitization on how to prevent diarrhoea and the like but, most of the time they reach out to a small number of people. I was listening to what people were saying on diarrhoea prevention, they were saying I do this and that, which means when the healthcare workers are doing community sensitization, most people are not available in their homes, busy with their businesses, ‘’I sell charcoals, flitters, ‘’ the time healthcare workers are coming for the sensitization, they will not find me, they will not find this one too and the result is that the messages reach out to a few people, so there is a need for the counselling on how we can handle this disease, that’s what makes it spreading

**02:** I think this disease is common because of what my colleagues have said, but they did not go straight. The issue is about our water sources, where we get water and what kind of water is it. You will find out that the water that we use is not protected, they are from wells or from the boreholes that are near the toilets or a bin. Let’s talk of food, most people just buy food and start eating. Some foods are not covered, and some are not washed, like fruits. When it comes to cooking, because of poor financial status, most of the foods are not cooked sufficiently, which means what causes diarrhoea is still there. This makes the disease to be there forever. Some people have a habit of cooking a lot of food and keep the remaining one uncovered

**All:** laughing

**0:** children may just pick and start eating

**02:** there are many things that are causing diarrhoea amongst us

**09:** in our rivers, dirty things are dumped in there like pampers. People are advised not to dump things in the rivers, and that they should have toilets, but we look at it, some peoples’ toilets are along these rivers, they use something and dump it in the river. They wait until night so they can dump waste in the rivers, and when wash from the rivers, we may get sick easily. If we stop dumping garbage into the rivers, it can be good because many people use the rivers, there can be more diarrhoea cases as well

**Facilitator:** what counselling do you need for dumping waste and pampers in the rivers?

**09:** as this one said, healthcare workers may come but, they find only a few people, if they say on such a day, everyone should come, to have a meeting like this one and discuss how we should protect our rivers or there should be a punishment for everyone dumping the waste in the rivers, our rivers can be protected

**00:** just to add on the same, you have asked how to deal with it. This has been happening in this community, through heart care workers people have been advised on how to take care of diapers after been used, they should be burned or dumped in a toilet, we have been hearing these, but I think there is hesitation, it looks like there is no ownership. If we can just go near that bridge, we will notice that people have turned that area into a bin, yet there are no healthcare workers, we are alone. It shows we have no sense of responsibility. If healthcare workers come up with a by-law for people not to dump things under there, it all depends on the community’s coordination because health workers may do their part but as owners, we have no sense of responsibility. If we had a meeting like this with the concerned parents and ask, what is our responsibility? If we ask each other here, everyone has a child, so each one of us has a mechanism on how we handle the used pampers, we cannot have similar mechanisms, you will hear some saying we do this and that, if we could be exemplary as we are implementing it by telling people, we should be telling people what we do. That’s why I was saying civic education should be given to people in the communities because there are some things…..

**Facilitator:** (how civic education should be given?

**00:** I have seen community members forming organisations like home-based care, such groups look at the welfare of the community members as far as hygiene issues are concerned. If we had such groups, it is us who are suffering, and we cannot wait for the health care workers to come and tell us to clean the rubbish yet it’s us who get sick. So, I think it could be a very good initiative, training people just like this meeting to say, ‘’Ladies and gentlemen, can’t we follow what we were taught and implement them in our respective areas, we should call for the meetings and tell people.’’ I think with time, such problems will end. During the day, you find someone with a pail full of pampers, he/she is going to dump them into the river and we are just watching him/her. We are sitting here, there is a pit there, houseflies are all over, we don’t manage our responsibility and when get sick tomorrow, we blame the hospital, ‘’They are not helping us, look, I have diarrhoea.’’ So I think we lack counselling because it is we who should protect ourselves, we should start ourselves, that’s the first part

**04:** I would like to add that, I summarize what these two have said. Most of the time during the funeral, we hear the headman telling the women to be taking care of pampers but, we just take it for granted, we don’t think of taking responsibility. Most things lack ownership, you should be telling yourself this is wrong. There are things that someone may be discouraged to say, brother or sister, what you are doing is wrong because they can be asked, ‘’Who are you? Is this yours?’’

**Facilitator:** ‘’is this river yours?’’

**04:** like that, we are discouraged ourselves but we are the ones living in that community, so you will see that where people dump the waste, under-5 children play with such waters in the same river. You can tell them, ‘’Don’t play here!’’ but their parents don’t mind, but you have that conscious to say ‘’Children should not play with this water,’’ and for you to speak it out, you will be blamed, ‘’who is she?’’ So, what he said is that if there is an organisation that would like to monitor us, we should be taking part as well so that in the process, we will learn. It should be our culture to do that

**00:** If you ask these people who are talking, you will hear that they have no rubbish dump. So, when they want to dump something, they do it through someone (cross-talk) they don’t mind if they dump them behind the toilet, as long as they remove them from their home

**All:** laughing

**00:** the first thing is that everyone should have a rubbish dump and if you have one, you cannot hire someone to dump the rubbish for you because when that pit is full, we excavate it, and you can turn the waste into manure, so if you ask all these people, they have a rubbish dump. So, you want to start hygiene practice because someone is dumping into the river, if you want to show something, ‘’umagwira madzira a njoka m’manja, koma ukalekelera kuti taswa, tating’ono ting’ono tomwe tija, sungatigwire tikuluma.’’ (It is good to start an intervention earlier than later). So if you ask whether they have garbage dumps in their homes, they will tell you that they don’t have, so how can they tell someone not to dump things into the river?

**Facilitator:** why do they have no rubbish dump? Or let me ask, do you have a rubbish dump yourself?

**00:** I can have it

**09:** I can have it too. Sometimes you can be staying in a rented house, you cannot dig a pit, the owner can do it or give you a bin but, it’s not a garbage dump only, people use that as a toilet, when it’s hot, you go out with your food or give food to the children. They house flies from there contact the same and when the children eat the food, they have diarrhoea

**10:** I want to comment on what this gentleman said regarding the congestion. Because of the highly congested houses, there is no space even for a toilet, so there cannot be a space for the rubbish pit. You cannot even have a space on the front of your house, so it is difficulty

**Facilitator:** so, you don’t have garbage dumps, you are living in congested areas and people don’t follow the counselling. What do you think can help reduce the risk of diarrhoea?

**05:** Blantyre City Council does not pay attention to its people, they don’t visit them. They can bring a bin, for example, they brought a bin at a clinic and it was full

**Facilitator:** is there a bin?

**05:** it was removed

**Facilitator:** was it removed?

**09:** yes, after it overflowed (cross-talk)

**04:** I would like to respond to this gentleman, but I will ask him. The government is failing to provide us with important things, and we are saying we need a bin yet the government does not provide us with it, what can we do? There should be counselling for parents to control the under-5 children. They should be trained to wash their hands before eating. We should tell them to eat hot food. We are few here, most people out there don’t mind where they keep food for their children. If everyone in the community learns that a child should only eat hot and covered foods. Children should be trained not to drink water from unknown sources, they should drink water which is covered, that can help us. We cannot depend on the government to provide us with a bin, I don’t know

**09:** we are focusing on diarrhoea, for you the trainers, train us on things that we should be following so that together we can reach out to other community members to tell them the prevention of diarrhoea

**Facilitator:** are we the trainers?

**All:** laughing

**10:** I would like to talk to all these people, first of all, let me agree with my brother here. I once lived in an area where every week city council collected the garbage. When the council brought a bin here, it was its responsibility, why, do people here in Bangwe pay city rates, and the use of that city rate is to help people in that particular area, are we together? If we are not receiving that service, it means our money is not used. So it is the city’s responsibility to do that. Let me come to what you said they should help us. It’s not about helping us because the mindset that we have is damaging us. When we talk of development, Malawians wait for what the government should do for us not what we can do to help ourselves, and because of that, we just wait for someone to help us, that’s why we are worrying that the government is not intervening on this diarrhoea. My colleague said that we don’t have garbage dumps in our homes, is it a government that can do that for us? Pampers that we dump, does the government know we are doing this? So, you can see all these things are up for us to take care of ourselves. So there is a need for us to come together because it’s our community, our children, and we should protect them ourselves when we do that, we can form our organization for hygiene sensitization, telling people that hygiene practices don’t benefit the government fully, it’s us because if the children will live, they will be ours if they die, the burden will be upon us.

**Facilitator:** we have discussed it at length, that one asked if you have a garbage dump, it was noted that there isn’t due to different reasons, maybe lack of space for that or we have no desire for that. Is there anything that you can do as Bangwe community members on our own? The government has a responsibility, of course, the city council has a responsibility too. On your own, what can you do to deal with this threat? If you agree not to dump wastes into the river, for people to follow, is there anything that can be done?

**All:** yes

**Facilitator:** what can be done?

**10:** I think the first thing cannot be space, we can collect the waste in a sack, making sure there is no water contact so that when the bag is full, we can burn it. I remember when I stayed at a certain plot….

**00:** (but for these wastes to get dry (cross-talk)

**10:** Someone said that you can put them on the roof for quick drying, then you can crush them and add them to the garden as manure, that’s what he was doing. We cannot put water in that rubbish pit, we only dump dry waste and when it’s full, we burn it. That was happening in the same locations

**08:** There are few

**Facilitator:** let’s proceed, yes number 8

**08:** if we don’t cover drinking water, smaller houseflies may contact it. Or you did not cover the food, the child just eats the food, it is a risk as well

**Facilitator:** Let’s now talk of malaria as one of the serious health problems here, what makes malaria a serious health problem?

**04:** too much mosquito breeding due to stagnant waters in homes and there are no bed nets in homes, children don’t sleep under the bed nets and when they are bitten by the mosquitos, they get sick from malaria

**Facilitator:** lack of bed nets and stagnant waters where mosquitos breed

**05:** if we say we are lacking the bed nets, we are dishonest to the health care workers. They distribute the bed nets….

**09:** (aah….

**Facilitator:** (let him finish

**05:** they distribute the bed nets and chickens sleep under the bed nets

**All:** laughing

**05:** you can visit homes and see how many chickens sleep under the nets (cross-talk) so what is difficult is that we don’t use our responsibilities. People have bed nets in their homes and they hanged them long ago, but they don’t sleep under them and they say, ‘’I don’t breathe,’’ they say a lot of things, ‘’I dream people chasing me.’’ Such things

**All:** laughing

**0:** some people are afraid of bed bugs that come in the bed nets, and that’s why they don’t use the bed nets

**05:** bed bugs do not get in the net no matter what

**Facilitator:** does it not get inside?

**05:** no, it just moves outside the net and you see them

**09:** I should testify on the issue of the bed nets. There are people who buy bed nets for fish catching, so they buy them at 200. Bed nets were distributed, we should not lie, it depends on our role of taking care of the bed net

**Facilitator:** those who buy the bed nets, how do they get the bed nets?

**09:** they go to the households, ‘’We are buying the worn out nets!’’ some people may damage it intentionally for them to get cash (cross-talk)

**Facilitator:** how much do they buy?

**09:** I am not sure, it can be 200, and that can cause a child or an adult to get sick from malaria because they don’t sleep under the bed nets, mosquitos are breeding too much

**Facilitator:** why do people decide to sell bed nets when they know they are stagnant waters and there is that risk?

**00:** poverty and lack of sensitization, I believe when they are giving out the bed nets, they give advice regarding the bed nets, so it is out of poverty and ignorance

**02:** I want to comment on the stagnant water where mosquito breeds. Mosquitos breed in the toilets as well, if you look carefully at these pit latrines, if you go in the evening, you will see a lot of mosquitos in there

**Facilitator:** how do you prevent it?

**02:** obviously we should be getting a mosquito coil and putting them in such toilets, you will see that a mosquito is killed before it comes out

**00:** let me comment, I think we are just biting about the bush, the issue is hygiene, as this brother said toilet is one of the breeding sites for mosquitos, we cannot say every toilet that happens, only the toilet that is not taken care of. We talked of diarrhoea, despite having a toilet in a household, we have noticed that toilet may contribute to diarrhoea, how do we use the toilets that we have the under-5 children, when such children want to defecate, we just send them to the toilet without monitoring them. They defecate behind the hole and maybe the household has many people, so everyone uses a toilet the way they want. Rubbish is outside or behind, and houseflies are everywhere, yet you are eating utaka outside there, these houseflies are contacting the food, so it seems like we lack hygiene practices for both malaria and diarrhoea, in other words, we should have a sensitization campaign to tell people what to do. Though people have the bed nets, there is negligence because when the health care workers are coming, we don’t even mind, but when we hear they are coming with maize to distribute, everyone leaves their things and rushes there, they prioritize free things, and not their lives, this issue should be discussed in our communities

**Facilitator:** what approach can the healthcare workers use to sensitize people?

**00:** this lady said that village headmen plea with people at the funeral, and we just take it for granted when they say, ’’ We are worried as a tribe’’ There are some who really get worried. When a mad person dies, ‘’as a clan we are worried,’’ people just take it usually, so when we see a village headman speaking at the funeral, we say that’s how he/she speaks always, for people to take what is been said seriously, there is a need for further step. ‘’when the village headman speaks, is there anything happens? There is nothing that happens, that’s why in the villages, there are selected people to out at night (community policing), but you will hear the same people are stealing. At the beginning, you said there is an allowance, this is that. Is that a payment? People take it another way, so if we say we should give counselling, it is the duty of the health care workers and they are happy with what they do, so we lack the advice for us as a community to have such groups, if we have the groups, village headmen should come to the top, they should come up with a policy, ‘’we have empowered you to go out in the communities for monitoring, and if you find such things happening, do this.’’ We visited the funeral, and during the speeches, a coffin was taken somewhere there because of the congestion, and people built everywhere. When the village speaks it out and ends there, if someone wants to build a house, can he/she go to the village headman and ask where to build a house? Not at all, so there is a need for monitoring, if we want to deal with this, we should not speak only. He has asked, ‘’Do we have rubbish pits?’’ we can speak out here, but there should be a group of people to monitor such things, ‘’what is happening here? What did you do? When we come back after 2 days and get the same, action will be taken.’’ It will be sort of a pressure and when such things happen often, they say, ‘’mphini yobwereza imawala.’’ (it’s good doing things repeatedly) when you visit homes often, ‘’We said this and that, why are you doing this?’’ follow-up is necessary

**0:** including a punishment

**00:** yes, including punishment. Once the rules are established, there should be follow-up, if nothing changes, punishment should follow, you will see that with time, people will be scared of doing something wrong. People don’t think when they get sick, it will be the suffering

**Facilitator:** we have discussed malaria, what you are supposed to do and what happens for malaria to be common. Let’s discuss the third health condition, what was it?

**00:** flue

**Facilitator:** why is it common here in Bangwe?

**04:** besides windy weather and the dust, sometimes we should be monitoring what our children drink. There are different types of drinks coming, some are diluted ones, so we don’t know what they are. Some drinks worsen flue in children because of the sugar concentration. Hygiene practices to their beddings, maybe they have taken days before washed, beddings can cause flue and cough besides dust which is out there

**0:** just to comment on the issue of hygiene, we have been to homes and heard a mother saying, ‘’Take your blanket out!’’ she is going to work, she is going to buy the charcoals for sale, ‘’not you of course.’’

**All:** laughing

**00:** she is going to buy the charcoals for sale, she is leaving the child behind, and telling the child to take the blanket out, and after all the mother knows that the child has urinated on a blanket. We see blankets beside the houses, and we then ask, do parents see this blanket?’’ which means parents don’t show responsibility because a child cannot say this blanket is dirty, wash it. Flue starts instantly. So, parents should be at the forefront because some of these diseases are due to a lack of hygiene practices. This one has talked of the food we are eating nowadays, we have been doing such things with St. Jones because I do first aid work. There are some foods that are not suitable for children, we just give them yoghurt, this and that, how do you know that this has caused flue in my child, we have to monitor things like these, ‘’how a child has reacted after eating this one?’’ there are some drinks that have an impact, so you just give them to the children and they cough, and then take them to the hospital, they cough throughout because we are not monitoring the foods they eat, we should not just be giving them drinks

**Facilitator:** did you say St. Johns?

**00:** yes

**Facilitator:** what is your work?

**00:** we do many things, so we look at different areas like motherhood, emergencies and the like. I belong to a group that deals with accidents, what we should do if someone gets wounded, and what we do if we get the person on the spot, so we sensitize people. Sometimes at the under-5, what people can do if a maize or pigeon pea gets in the child’s nose? People do rush to the hospital, not knowing there is moisture in the noise that will cause the maize grain or pigeon pea to expand, by the time you get to the clinic, the child has fainted. So we train people on what they can do when the child has an accident. This is summer season and flue is common, most children have a nose bleed and when a child has nose bleeding, it’s not time to rush to the hospital, do this and that, and you will see an improvement

**Facilitator:** can you train us how you at the end, how to hand a person who has (43;00 not clear) were you aware of it?

**09:** no, I wanted to ask as well. That issue regarding the flu, what is needed a guidance. Some people keep things under a bed, there are dusty areas, and they need to be cleaned daily. We have spices that we add to the relish, but they may cause allergies and flue, we should be responsible as well knowing what children dislike, and things like perfumes, we should follow these up to say if I do this, will my child not get sick? So we should be following up with such things, they help to prevent flue and protect a child from flue

**02:** I should agree with what my friend said about children’s blankets. You will notice that the blanket is bigger so that the child is failing to carry it, he/she is just dragging it on the floor. One day you will notice that the rope that we use for hanging clothes is full, it is a blanket that is removed from there and put them on the dusty floor, such things cause flue. I give an example of my neighbour. His/her child was playing with dust, and eating it. I was then speaking to him/her, ‘’Aah why are you speaking these to me today? For every child to grow well, it’s because of these things.’’

**All:** cross-talk

**02:** so you can see there are things that people take them as part of life, they say God protects, so they take no responsibility for caring for a child and the result, a child plays with dust, at the end of the day, flue starts. Most of the children play together, they transmit that flue

**09:** we have mentioned dust because we are passing through summer. During the cold weather, the flu is mixed with pneumonia because we have been meeting the children going to school wearing uncovered shirts, so cold weather makes children sick because that weather affects their lungs. If parents hesitate to cloth their children adequately during the cold weather, flue is common

**Facilitator:** what makes them hesitate?

**09:** they just don’t pay attention, as long as they have bathed them, children are not bathed

**0:** sometimes it’s because of hesitation, children have jerseys (not clear) so when talking, look at both sides. I did not comment on that issue of flue because…

**Facilitator:** (you should comment

**0:** there is a climate change, 100 people can get sick from the flu at once. It’s not that you are sleeping on duty floors, it’s because of climate change, and that’s why rich countries are taking part so much, replacing trees and the like. Some people have blankets when you see them, eish (not clear)

**Facilitator:** how climate change is related to flue?

**0:** our air is polluted, that’s why these things happen

**02:** to add on that, what we said had caused COVID-19? Climate change, can we mention how it started? Scientists said it was an issue of climate change, and that’s why I agree with him. I forgot to comment on the issue of overpopulation. I am a teacher, and there is congestion in the classes, so children easily transmit the flu. With limited spaces available, it is not possible for them to protect themselves

**09:** I want to comment on that issue, as he said children transmit flu. When the children come back from school, bathe them before they sleep. Remove the clothes they wear because some just go to bed with those clothes they wear

**Facilitator:** how do you make sure children prevent flu at school?

**02:** it is difficult for us because it is a public school, unless the time COVID-19, we could force every child to wear a face mask. We cannot force a child to wear a face mask now, at the end, you will just (49:26 not clear) we are thankful because when you cough, you should not do it on your friends, of which we hear that (49:44 not clear) once my child has flue, I don’t allow him to go to school (59:56 not clear) ‘’you are not going to school, sit down here.’’

**00:** just to comment on that issue, yesterday I had a chat with one of the teachers from here Namatapa. We consider it a school which has many learning blocks and some of them are not used. When we take the government teacher-student rate, how many children per teacher?

**02:** 60

**00:** one teacher is supposed to have 60 learners, and not more than that. When I was chatting with that teacher, I noted that one teacher is manning 160 learners, adding 100 on top of that 60. How can a teacher manage such a class? The teachers have no alternative because the government has already failed, ‘’what should be done?’’ ‘’They want auxiliaries.’’ But auxiliaries take longer, in the process, children have flue because they are in one place

**Facilitator:** I did not want you to tell us that you are failing, I just wanted to know what you do, okay let’s proceed. It looks like there are disease risks here, I would like to know, when someone gets sick, where do they access the treatment, and what do you do?

**0:** when a child or an adult gets sick, we have a public clinic where we go to access treatment, as we go along, we will discuss the challenges that we face at the hospital because these diseases we are discussing, diarrhoea, malaria and the like, Bangwe has a big catchment area which does not fit that clinic. When we go to Queens, they tell us, ‘’Go to Bangwe clinic first!’’ it looks like this hospital is not enough, maybe I should comment on the issue of the vaccine because that clinic is small, there are any people who should be accommodated, let’s say dental issues, it used to be an issue for the elderly people, now if you got to dental department, you will find notice many people than any other department, and there is one doctor from Queens who comes once in a while and when that doctor starts working, he/she does not follow the guidelines, maybe he might help the first 10 patients following the guidelines, the rest will not be treated as it was supposed to be. When we go there this time, you will see that those who are assisted are those who went in the morning because health care workers do book drugs for that time, and when the drugs are finished, for that day they are done. If you go after that drug limit, you are told to buy them from the pharmacies yet I have nothing. In the process, the condition is worsening, death rates are high. If it’s diarrhoea, when I am told to buy from the pharmacy yet I have nothing, things get worse. So we have problems, and we depend on the government to fix the hospitals for us, and if there are some initiatives that we do like the first aid that I mentioned, people say, ‘’We didn’t know,’’ If you ask what they do if a child has a maize grain in his/her nose, they have what they do even though those actions are not recommended, so when they are not assisted, there are things that they try to do, and in the process, they worsen the condition

**Facilitator:** you go to the hospital of course, but you are saying there are things that you do on your own, what are they?

**09:** like first aid

**Facilitator:** whether the first aid or treating the patient

**02:** if we are able to, we take a child to the private hospital, most of the time we go to the pharmacy because we know this is flue or diarrhoea, we just go to the pharmacy and say I want medication for diarrhoea or flue, that another part that we do

**09:** if diarrhoea is just too much, we dilute salt and sugar to make thanzi, and then we take him/her to the hospital. We also administer rice water (madzi a mpunga) to prevent lethargy, so we do these things when going to the hospital. If the child breastfeeds, we breastfeed him/her every now and then because it helps the child. So we force him to eat porridge instead of forcing the child to breastfeed

**Facilitator:** is it every time and everyone who thinks of going to the clinic when they are sick?

**00:** aah no, there are people with different beliefs in the community, some don’t go to the hospital. Since we are saying there is a congestion in the public health facilities, and we go there if we don’t have anything. So when we go, we are not assisted, we think of a plan B he was talking about. ‘’the child seems to have malaria, let me buy drugs,’’ we have experienced this in the past few days. There was a child who had diarrhoea. After that, she thought it was teething. At one point, I should be helped, what is the connection between teething and diarrhoea? So she had a child, 5-6 months old, and the child had diarrhoea. Her friend advised her, ‘’that diarrhoea is a result of teething.’’ She had that mentality, as we know when women say it is teething, they don’t take it seriously. ‘’it’s teething, I should be giving him/her some things.’’ When the child got hydrated, she thought of going to the hospital, the child was so weak, ‘’What happened?’’ ‘’The child has diarrhoea from yesterday.’’ ‘’why didn’t you come?’’ ‘’aah, I thought it was teething.’’ When the child was examined, it was not the issue of teething, he/she was really sick. So I wanted to answer the question of whether we go to the hospital whenever a child gets sick, sometimes we don’t go to the hospital due to some advice that we get from our friends. So, we have friends who give us some advice and when they tell us what they know, you think going to the clinic is not necessary, but you should do what they want. Most people have died because of such things, that’s what I wanted to comment

**05:** When commenting, we should think. When a child is teething, he/she feels itching, maybe the child touched something, he/she scratched him/herself. When a child is teething, he/she doesn’t have diarrhoea, ask healthcare workers, and they will tell you. These children have tough things that have germs. I will give you an example of my friend, an engineer, he/she was hurt by a nail at work, and it pricked her /his foot within a short period of 2 months (not clear). One night when he/she slept, he/she started feeling hot and went to the hospital where he/she was told that the foot had to be amputated

**Facilitator:** was it amputated?

**05:** it was amputated. That nail had germs and we should be careful, some germs just wait to access our bodies, so his foot was amputated and it was too late because the whole leg got amputated. So things that we don’t discuss properly, we cheat each other, ‘’ooh when a child is teething, he/she has diarrhoea,’’ the child has diarrhoea when he/she is alone because of wanting to scratch the gums when he/she is with his/her mother, it does not happen. Our Bangwe clinic is one of the facilities that are pathetic because there are always no drugs. People living around the clinic don’t mind. Bangwe is different from Ndirande. Drugs that are supplied at Ndirande Health Centre, are not available at the Bangwe clinic

**Facilitator:** what do you mean?

**05:** people’s attention, so if you have a car, you need to pay attention to it, don’t just give the driver a car without checking, the driver will go with it on the railways, it will be destroyed. Similar to this clinic, we were supposed to have a very good committee that checked what drugs were being supplied to the hospital. They can supply drips only yet people have diseases that don’t need drips, then drips for what

**Facilitator:** Okay

**05:** let me proceed. There is a committee in Ndirande. Do you know that if there are no drugs at Queens, they get them from Ndirande? If you get sick here, you cannot access the treatment from Ndirande

**Facilitator:** from Bangwe

**05:** from Bangwe to Ndirande, you will not access the treatment

**Facilitator:** unless I say I am from there

**05:** you should provide them with evidence that you come from Ndirande because people pay attention to the facility. Last time, there was a shortage of anaesthetic drugs, they were taken from Ndirande, but Ndirande refused and said,’’ these are ours, we signed for these.’’ Until the chairman said, ‘’Guys let’s give them to save lives.’’ So the drugs were taken from Ndirande to Queens. The same drugs are not available at Bangwe, where do they go, there is no attention, as I said, we should change out mid-set, we should be paying attention to details, whether its home or community. In all aspects, it seems there is a lack of ownership, just as he said, we think it’s for the government, we don’t take the facility like ours, ‘’aah this is government road’’ we don’t say it’s ours and when it is damaged, we don’t fix it, ‘’government should fix it,’’ so we don’t pay attention to such things

**Facilitator:** are there things that make people not go to the hospital on top of a lack of drugs and beliefs?

**03:** people don’t go to the hospital because of abuse that happens at the hospital. People are abused sometimes. You can go to the clinic, sometimes people wake up with tempers, like teachers, they quarrel with children and when they go home, once his/her child is wrong, they treat the child differently from how he/she was supposed to treat a child. Sometimes people don’t want to go to the hospital because of a lack of attention and the abuse. Some people don’t know where to report such issues, so when you get sick next time, you decide not to go. There are some people who made up their minds not to go to the hospital because they once got abused. For the under-5 issue that we talked about, you can take your child to the clinic for the vaccination, and you will meet with a doctor who vaccinate the child until the child’s buttock gets swelling, The next time you see her not coming, she thinks of going to another hospital and if that hospital needs transport, obvious she will not go, because she was abused last time

**04:** prayers are good, but sometimes we are confused. There are some people who think that if I am praying, I will not use any medical means. With all these vaccines going on, there are some parents who decided not to have their children vaccinated, so every vaccine that may be introduced, they don’t receive it. They don’t go to the hospital, ‘’we will stay indoors and pray.’’ We have heard some people died. We heard what happened in Mulanje, it was closed because of the faith that they have, believing when they are taking part in medical things they are sinful. The way we understand the bible, there is nowhere written if we use medicine, we are sinning. We are cheating each other here on earth don’t receive the vaccine, and your children will die. ‘’if you are going to the hospital, you will be forced to receive this and that.’’ Such issues have contributed so much that when a child gets sick, people should not seek medical treatment, rather they should try home remedies because we are going to meet the vaccine issues

**Facilitator:** this issue of vaccination is one of the issues that I wanted us to discuss, he has already said, ‘’How they injected my child, I will not go next time,’’ and this could be a reason why people don’t go to the hospital and the same reason why people don’t receive the vaccines. Let’s proceed, are there other reasons why people don’t receive the vaccines? Maybe beliefs, what you experienced when the child was receiving the vaccine, is there any other reason why children don’t receive the vaccines?

**09:** people started getting afraid of the vaccines when the COVID-19 vaccine was introduced. From that time, people started to have bad perceptions towards vaccines, but this is out of ignorance because vaccines have their work in the bodies. For example, children, when a child is born, he/she has to receive the tetanus vaccine and the other oral vaccine that is dropped into the mouth. These vaccines help children’s bodies but people don’t know. Because of such rumours, when the health care workers come with the vaccines in schools, some parents refuse their children to receive the vaccine, ‘’don’t vaccinate my child,’’ these vaccines protect children from diseases. Most of the vaccines were damaged from that time of COVID, even Queens itself, it is not congested like before, people are afraid of the rumours

**Facilitator:** what are people afraid of Quuens?

**09:** they think their children will receive the COVID vaccine and from that time till today, people are tarnishing the image of the vaccines

**00:** even the recent oral vaccine, people were hiding their children, locking them up indoors, ‘’don’t receive it’’

**Facilitator:** what was the vaccine?

**00:** polio vaccine, it was oral one

**0:** there are language issues because vaccine means cutting yet the polio one is not like that

**09:** they say, ‘’Mine already received the vaccine!’’ yet they did not, and such people need advice and sensitization on how the vaccines work. I think when we go to the under-5 clinics, they tell mothers, ‘’We are giving this child oral vaccine which will protect the child from this and that. We are giving your child this vaccine to prevent his/her organs from getting weak.’’ So it depends

**Facilitator:** why people were afraid of COVID vaccine?

**05:** it was because of religious and other beliefs, and also, were you diagnosed with COVID-19?

**Facilitator:** myself?

**05:** yes

**Facilitator:** I should answer at the end

**All:** laughing

**Facilitator:** I will answer you, please proceed

**05:** people who were testing COVID during the early days made the mistake, they were inserting a pipe in the nose, it pains so much! So when such a person goes back home, he/she could report by twisting the face, ‘’What’s wrong my father?’’ ‘’they tested COVID, it was so painful.’’ People then thought, if testing is so painful, what if they inject you? That’s how it started

**00:** I tested too, it’s painful

**05:** not what they did to you, that’s not COVID testing, they insert a pipe

**All:** laughing

**Facilitator:** so, beliefs including religious and testing process, was there anything else that prevented people from receiving the COVID-19 vaccine?

**10:** Another thing was the rumour, people said that the COVID-19 vaccine was a robot. Once you receive the vaccine, and when the time comes, you will be controlled by a machine. When people thought of that, they were not going

**00:** people brought misconceptions

**Facilitator:** through what?

**00:** people were saying that this vaccine is very dangerous, ‘’it came from America.’’

**0:** there was a sort of sabotage, at one point, people were not sleeping apart from me because I knew it was not true. There came the bloodsuckers

**All:** laughing

**00:** I knew for sure that’s not true because I have never met one ever since. I wrote my examination in this classroom

**Facilitator:** here

**00:** yes, in 1994. So nobody has ever seen these things, there are some people who use such things for the (1:14:06 not clear), people became rich because of such things. We are not mentioning the names here. So they give this one 200, ‘’say this and that’’, some people were killed, like in Mulanje, because of bad behaviour. Those things are not available, but they create them

**Facilitator:** what is the purpose for doing that?

**00:** just to confuse people, for them to raise money

**02:** Although we are out of our topic, I would like to agree with my brother. When there was blood sucker rumours, I was there. I was just sleeping and my neighbours could wake me up, ‘’Don’t you hear what is happening outside hear? When they will come to your house, we will leave them to kill you!’’

**All:** laughing

**02:** they were calling me by my name, ‘’Why are you not coming out of your house?’’ because I knew those were things that aimed at creating fear in people but they didn’t exist. Secondly, it’s some people’s opportunity to steal from you, so if you are not careful, ‘*’mumazavina nyimba osadziwa kuti yachokera pati.*’’ (you can dance to a tune that you don’t know its source, meaning you can join things that you don’t know) another thing is for political mileage. They want to get popular politically or to advance their motives, it happens as well. Let’s get back to the issue of vaccination, I think the issue is with school. If education is insufficient, the understanding is insufficient too, and it’s very difficult because if we look back at where we are coming from, as far as vaccination is concerned, only the vaccines children receive at birth. That does not happen because of any awareness, but we are used to that. We found them, and they still continue. These vaccines are coming when life is going, we are ageing. There is a need for a sensitization team to come, ‘’when did this vaccine start? When was the research conducted? And to whom the vaccine was tested? What were the results after testing?’’ people are not told these things and the result is that people are not aware of the whole process for the vaccine to be administered to people, so people have fears, ‘’they want us to become barren, they want us not to overpopulate, they are few people in Europe.’’ We are referring to disease, there are countries where malaria doesn’t exist, and here in Malawi we have it. I was researching the issue of malaria. In 2007, malaria had over 236 medicines. Ever since malaria started up to 2007, all malaria medicines 236 and out of 236, there was no tablet that could cure malaria because the time they collected malaria samples and took them to India or Kenya, it was discovered that plasmodia had changed into another shape because those countries have no malaria cases. Same as this vaccine, we are receiving because we are the ones at risk more than our friends, so they did not test or give us the testing tools. So people think it’s obvious they want us to die, they want us to become barren, and they want to give us something so that we should not reach there, so they should be ruling this world by themselves. So I have talked of school, secondly, every research should be given to people, how it was conducted and the results of the research, so people will have confidence in the vaccines.

**Facilitator:** Alright. He said that one of the reasons why people receive the vaccine is because most of the vaccines have been there ever since, and our parents received the vaccines, and our children are receiving them too. We don’t understand the vaccines that were introduced when we grew up. I want us to discuss what was motivating people in the past to receive the vaccines.

**000:** a child never gets sick every now and then

**02:** I think it has to do with the leadership that you have. I will give an example of Nigeria, the time the polio vaccine was given, (1:20:20 not clear) Nigeria refused that people from Nigeria should not receive the polio vaccine, and as a result, the majority of people in Nigeria are disabled due to polio. The time they accepted the vaccine, polio was so common. Here in Malawi, the leadership that was there was MCP, the government accepted the vaccine at that time and many people were saved from polio. Most of the time, it has to do with leadership, if the leader says, ‘’Learn from me, don’t get afraid. If it kills, I should be the first one, but I am assuring you that if you allow this, it will help you.’’ Everyone will receive it.

**Facilitator:** leadership and sensitization, anything else that you think can make people receive the vaccine easily

**000:** yes, approaching people first, and after that, people will receive it because of the detailed explanation

**Facilitator:** what do you think can be the approach here in Bangwe?

**000:** first of all, they will tell the community members. During the second visit, people are aware of it, so when they come with the vaccine, they will not struggle because people know

**04:** if the religious leaders here in Malawi worked with the health care workers, religious bodies would have helped so much because there is an emerging spirit of listening to the religious leaders. If such leaders do sensitize people besides ministering to people spiritually, they should also tell people how to take care of their lives physically, it can help so much because people don’t mind what the village headman speaks at the funeral or community gathering. Even if the health care workers come, what people care about is, ‘’What does my pastor say? What does Papa say?’’ we should help such people because they are revived so much that they cannot go to the hospital, and that’s why, fellowship prayers are full of women, and once women get the message of not bothering with vaccination issues, it’s done. ‘’bring your children for vaccination.’’ So they tell each other, ‘’I cannot go, I cannot do this and that’’, in the process, we are damaging health workers’ jobs, even the issue of hygiene, you will notice that those who pray, don’t practice hygiene, they don’t mind. They pray too much, they even speak tongues, but they cannot take care of things. I was passing somewhere and someone spat saliva

**Facilitator:** those who speak tongues

**04:** our conscious should be convicting us not to spite saliva where people are passing by, do it where you can manage to cover them for others not to see. Some people don’t think, ‘’as I spite, someone will use the same path’’ (cross-talk) but we go to the mountain for prayers, and we think of spiritual things only, abandoning the fresh. There is a certain church that I am not sure of the name of, they have health talks besides the spiritual teachings. They mainly teach how to eat and care for the bodies, and after that, they teach spiritual things. Most of the preachers are destructive, so that people should not receive health services

**Facilitator:** why do people listen to the religious leaders more than the other groups of leaders? There are people who should be listened to; teachers, village headmen, healthcare workers and politicians, why do they believe so much in the religious leader?

**04:** mmh

**00:** there was an organization that was doing research on who is most trusted. First, it was religious leaders, second was the village headmen and politicians came third

**Facilitator:** mmh, who did that research?

**00:** I have forgotten, but it was here in Malawi during the time of the election. Social commentators were asked, ‘’why do people trust religious leaders than politicians?’’ they said that politicians are deceivers, and village headmen are used by the politicians’’ so they can trust religious leaders because their principles are not from humans, they are from what they believe in, whether it’s Quran or the Bible. So people believe in them because they don’t take their principles from a human being

**0:** things are complicated now

**All:** laughing

**0:** because when she was asked who to believe in, she said religious leaders have negatives, this other side there are negatives as well. I have examples too, there was an issue of sabotage, when the rumours were too much, and they affected some people’s mindset. I was thinking of what to do if the rumours are affecting the vaccine intake, where do these rumours come from? How can they stop spreading? It is difficult to come up with an answer. Let me follow what this sister said, maybe we front religious leaders too much, so we take everyone as a Christian or a Muslim or Napuse-Napuse

**All:** laughing

**0:** just an example. You will see that everyone values their faith, which means if we take such messages to the church, people will believe and say our church does what God wants, so they will take such messages seriously, I agree with her. On the other hand, I have seen people here in Banagwe migrating to Nkhataby or hilly areas, ‘’let’s go there, we don’t want our children to receive the vaccine,’’ such things are coming from people who go to churches. If we believe in the religious leaders, we may damage some things, so churches have some beliefs that are not good. Let’s go to the village headmen, they are our go-between, but they depend on politicians. If we believe in what they say, there are negative issues, that’s why I am saying, our problems cannot be sorted overnight. There are things that are destroyed from the past, they cannot be fixed in a day but we should know that these problems do exist and for them to come to an end, it is us. So every topic here is debatable

**Facilitator:** maybe time is not enough for us to sufficiently debate every theme (cross-talk) but it gives a starting point for the discussion, so it’s good to discuss the emerging issues further, so its good to look into these things deeply and say even though we are saying this, there is this problem, that’s very important

**02:** we live in a global community today, though I am Malawi, I am in America, I receive information from America, and I receive information from China. You will see that many things that are confusing today, it’s because of social media. In some countries, when they see that what is been discussed on social media brings negative effects, social media is banned to protect some things from spreading so that the government can disseminate the right information. You asked why people don’t go to the hospital, social media told people to join herb groups and the like, so read herbs that can cure flue, and such things are linked to the vaccines as well, ‘’people had a reaction in China after receiving the vaccine, I may react as well.’’ Forgetting that we are living in different environments and what someone can experience is different from another one. Allergies come as a result of receiving the vaccine which is not suitable for your body, so if we bring such things here in Malawi, we rule out that this is not good because I have seen it somewhere, it had such a reaction, so social media should be controlled so that wrong information should be banned instantly before it reaches to people and make them not receive necessary thing.

**Facilitator:** Okay, we are going towards the end of our discussion. Let’s get back to what number 3 said regarding what parents experience on their children after they receive the vaccine, maybe the child is not properly vaccinated. Are there any other concerns that parents experience following the vaccination that affect their decision as to whether they should receive the vaccine or not?

**03:** I am the one who said it, I saw with my eyes. A child received the vaccine, the vaccinated area got swollen. We hear when a child receives the vaccine, he/she has a fever and all that, but for that child, it was worse, some people say when that happens, we should be wiping with salty water, but it never worked out. Some say such children should be bathed with water boiled in a pot in which nsima is just cooked (*nkhoko za nsima* madzi a), ’’is that so?’’

**09:** ooh?

**Facilitator:** with what?

**03:** after we have cooked nsima, we pour water in that pot and the child should bathe in that water. I have no experience, I do visit homes

**Facilitator:** did the child do that?

**03:** yes, but after some time, the area got swollen like this. When I saw that child, as a first aid officer I touched him/her. In the first days, inspection was difficult because parents didn’t know why the child was crying, so the area became so stiff, so it was not a good vaccination. We tried to handle it unkindly sort, of because the child was in pain. After that, pus came out 3 times. So the positioning of the syringe was not that good, and it caused a wound inside. The problem came due to an injection and a mother had something inside, thinking that this was a result of an injection, so she developed a negative perception toward the injection. We could encourage her sometimes, telling her that this happens sometimes because those who do the work get tired, it’s only one person doing the job but there are many people to be attended to, many children are born and under 5 clinics are always full, yet it’s only one person working. There are newly trained people who have no experience, so there is that feeling that when I go back there, I will experience the same. ‘’I did not sleep these days, should I go there for a second time?’’ so they withdrew the idea of going to the hospital, so I just wanted to speak this

**Facilitator:** is there anything different from what he has said?

**09:** I just wanted to comment on the same, I once experienced it. Whenever I went to the clinic, there was something stiff inside the child, so I was sent to Queens. When I went there, they told me that the child was not vaccinated properly, so they removed pus and fatty-like things. I asked them, ‘’does this happen to every child?’’ they answered that not every child, the problem is with who injected that child, he/she did not inject him/her well, but you should not be discouraged, the child will never experience the same with the remaining vaccines

**03:** the language they use is that those who injected the child had bad hands, they don’t say he/she did not inject the child well

**09:** they say warm hands

**00:** my child got sick from the tonsils, and his/her mother took him/her to the hospital. The child refused to be injected, so they were wrestling there. The type the medicine was that if a child has hidden the body, drugs could not get in the body. So they called me from work and when I came, just as you said, some people don’t know how to inject a patient. So I told them, give me that child, and you will inject him/her, after that the child was flexible and got injected. That health care worker didn’t know what to do for a child to receive the injection. So our friends should be trained, they don’t know everything but if they are trained, they can do it

**0:** children don’t receive the vaccine because of the mindset that parents have. I work with both parents and children. There is a time when the government distributes albendazole. You are my witness here, you heard that children will receive drugs that will knock them out. ‘’you are not going to school today, you will stay here.’’ All this because children were told to get ready for the drugs and they should eat enough food. People say that children will become barren when they receive the drugs. We all received those drugs, who is barren here?

**All:** laughing

**0:** we are telling our children that if they receive the drugs, they will become barren. I tell my children that they are told not to receive this vaccine because they will become barren, are you not going to get pregnant right from this school? If we were barren, would you have been born? Because it is us who first received the drugs but you were born, which means whatever is said is not true but people take it as true

**Facilitator:** finally, let’s talk about cholera vaccine. You said everyone heard of it, how was it distributed here in Bangwe?

**04:** I may not know because I go to the clinic with my child, so I cannot know who else went after me to know whether there are many or fewer people

**05:** this vaccine came in 2012 and before COVID came, most parents were receiving this vaccine, but after COVID, those who received the vaccine, I don’t know. It was the cholera vaccine that I received, and I know what it was doing. What people were saying in the drinking joints, you would wonder, is it the same vaccine? So when such people go back to their homes, it is every vaccine including polio, if many children receive it, it will be fortunate. I heard from my own tent saying (1:42:31 not clear) I heard children saying, Dad, I will not go to school’’ their religious faith does not allow them to take medication

**Facilitator:** people were receiving the vaccine but COVID has disrupted everything

**All:** yes

**09:** I heard men talking when the health care worker came. ‘’how can we help you?’’ ‘’I have a message for you.’’ ‘’don’t bother! Take your box and go! You want to vaccinate us with cholera vaccine, why don’t you give us a vaccine that we should not feel hungry?’’

**All:** laughing

**09:** the health worker left, so even in schools when many children heard about this vaccine, they were absent from school because the COVID-19 vaccine had bad rumours. It will take another sensitization for people to be aware of the newly introduced vaccines

**02:** I once contacted COVID and I got sick with it. When I recovered, I made up my mind to get vaccinated in town because felt the pain of it, so what happens with cholera, people at our workplace refused to receive the cholera vaccine because of traditional beliefs. I told them there is a cholera vaccine and according to the situation, people should get vaccinated to prevent it from spreading. The politicians that we had said none should receive the vaccine. Was that a school meeting?

**02:** yes, after 4 days, we heard that the same politician and his/her mother had cholera. So that politician took the whole family in a car to go and receive the vaccine, and you know the health workers, they came and told us that those who were leading you have received the vaccine, so they came back and mobilized people, from that time, people started receiving the vaccine, that’s why I am saying, it’s up to the leadership

**Facilitator:** the politician told people not to receive the vaccine for what reasons?

**02:** they said that if they are failing to give us maize, should they give us the vaccine?

**00:** there was a group of people doing the registration of the names, when they approached me, I refused because I didn’t receive things, there were the right people to receive the coupons. First, a man came, and he was chased away to say we don’t receive this thing. ‘’when you want names for free cash, you go to those whom you know, for the vaccine you just vaccinate everyone’’

**All:** laughing

**00:** one day I was laughing when I received a message about polio vaccine and I said to myself, ‘’so tnm know my number only for polio, but they don’t know my number for Mtukula pakhomo’’

**All:** laughing

**00:** when you go through that situation, you understand it better because how critically ill I was that time, it was by the grace of God for me to be alive. Those who died are not stupid either. If the vaccine kills, let it kill me because I already died. Such things made me fear, like what my brother said, I did not receive the cholera vaccine because I ask myself, ‘’What causes cholera?’’ I prevent this and that so that I should have diarrhoea, and it depends on what you are exposed to, that can put you at risk or protect you. So it depends on the awareness of the environment that we are living in

**0;** at least you contacted COVID here in Bangwe, I got it from Nkhatabay- Chintheche (cross-talk)

**00:** I was alone in-doors, ensuring that I did not transmit to anyone but it was painful

**Facilitator:** Alright, this is what I had, we have discussed for 1 hour and 30 minutes

**00:** has it not exceeded? This is 12 o’clock

**Facilitator:** Okay, that’s true, we have discussed this for about 2 hours. Thank you for your coming and your attention. I would have loved we could still be discussing but, no matter how good is a song, it comes to an end. So thank you for your coming

**All:** thank you

**09:** thank you for your coming so that we should be aware of some things and learn from each other

**Facilitator:** thank you.

**End of interview**
