# Supplementary material for: Exploration of the social determinants of diarrhoea, rotavirus vaccine uptake, and vaccine ‘fatigue’ in Ethiopia, Kenya, and Malawi
Source: PLoS One. 2025 Sep 9;20(9):e0319691. doi: 10.1371/journal.pone.0319691 (PMC12419581; doi:10.1371/journal.pone.0319691)
Supplement: S1 Data — (ZIP) [file pone.0319691.s001.zip › Supporting Information Files/MW_8FGD.docx]

**F:** Alright, there are some few things that I want to discuss with you people from Bangwe. I would like us to discuss about diseases that most of the people here suffer from, we are also going to discuss about Diarrhea especially about its causes, how we can or we prevent it. We are also going to discuss about different vaccines that are mostly administered to children, we are also going to discuss about your experiences with Rotavirus vaccine as well as what people talk about this vaccine. Of course, we are going to discuss about other things as well but mostly, our discussion with dwell on the issues that I have mentioned. so, I will ask you to raise your voice as you are speaking so that your voice should be recorded clearly.

**00:** (All) Silent.

**F:** In your own opinion, what diseases do you think are common amongst the children here in Bangwe? Anyone can start.

**P 7:** The disease that is common among the children here is Diarrhea.

**P 4:** Malaria is the disease that is common among the children here in Bangwe.

**P 5:** Chicken pox.

**P 2:** Most of the children here suffer from Scabies.

P 1: [Not clear]

F: Welcome, you have found us in the midst of our discussion. We are discussing about the diseases that are common amongst children here in Bangwe and our friends have mentioned Diarrhea, Malaria, scabies and chicken pox. P 2, you want to talk?

**P 2:** I said that the disease that are common amongst children here in Bangwe is Scabies and the other one is Pneumonia.

**F:** Alright, Pneumonia.

**P 3:** Asthma is also another disease that is common among most of the children here.

**P 6:** Malnutrition due to lack of food that contains essential nutrients.

**F:** It is also a problem here in Bangwe?

**00:** Silent.

**F:** Your number is P8 and you P 9. you are going to use this number when you will be giving us your views.

00: Ok.

**F:** Are there other diseases that you think are common amongst children here in Bangwe apart from the ones that others have mentioned?

**P 8:** Diarrhea is a common problem among the children here because most of the people do not practice hygiene. For instance, most of the people do not wash their hands before feeding their children.

**F:** This one is telling us on ways of preventing Diarrhea, we are going to discuss about this later. Are there other diseases that are common amongst children here?

**00:** No

**F:** I know that each and every disease is dangerous but I want us to think, out of the diseases that we have mentioned, which ones do we consider as a burden?

**P 6:** The disease that we consider as a burden is Diarrhea, a lot of children from Bangwe suffer from Diarrhea.

**P 4:** It seems that Diarrhea is a disease that is common among the children here in Bangwe.

**00:** It’s is due to the fact mostly we use water from the rivers for domestic purposes and this is why most of the children now suffer from Scabies here.

**F:** P 5.

**P 5**: I would say that the disease that we consider as a burden here is Diarrhea.

**P 4:** The disease that we consider as a burden when it comes to children is Malaria, this is due to the fact that most of the children do not sleep under a treated mosquito nets and due to the fact that there are a lot of places with stagnant water that acts as bleeding grounds for mosquitos.

**F:** Alright, we started with the diseases that are common amongst the children here and we have reached a point at which we are able to say that these diseases are the ones that we consider as a burden among us, right?

**00:** Yes.

**F:** Now out of the diseases that you consider as a burden, what makes Diarrhea as a burden here?

**P 4:** Because we don’t have access to clean and safe water in our households. We don't have boreholes here in our communities and we usually fetch water from the rivers where a lot of things happen.

**F:** What things happen in those rivers?

**00:** Some people defecate in the rivers and there are some who drink water from these rivers.

**P 7:** I would say that Diarrhea is a problem because the water that we used in our household is usually fetched from the rivers that are polluted.

**P 2:** Aaah most of the children suffer from Diarrhea here because they don’t prepare and handle their food with hygiene. There are some women who are used not to wash their hands after visiting the toilet. Such kind of women they just breast feed their children without washing their hands after visiting the toilet. So such kind of behaviors are the ones leading to more cases of Diarrhea among the children here in Bangwe. This is due to the fact that in most cases Diarrhea is caused by lack of hygiene in households and when children eat unwashed fruits, it means that they eat together with other germs that are responsible for causing Diarrhea.

**F:** Lack of hygiene in our households.

**P 5:** When children eat the food that is not properly cooked, they suffer from Diarrhea, even the adults can suffer from Diarrhea by eating such kind of the food.

**P 1:** Us women in most cases allow our children to eat food before washing their hands.

**F:** In terms of the water, you have said that you usually fetch water from the rivers, right?

**00:** Yes.

**F:** I just want to know, are there no sources of clean and safe water that people use for domestic purposes here?

**P 9:** If we could have had boreholes in our communities, this problem could have been solved even if people would be required to pay something for them to fetch water from such sources.

**P 8:** There are more cases of Diarrhea among the children here because we don't have access to clean and safe water here. Due to this most people are forced to fetch water from sources whose water is polluted.

**00:** We don't have boreholes here a thing that is making people not to have access to clean and safe water here.

**F:** Rivers, wells where else do people fetch water?

**P 5:** Wells, people fetch water from the wells.

**00:** Wells that are dugged out by ordinary people or experts?

**00:** There are some people who has the wells in their compounds and they sell water from these wells to other people.

**F**: Where do most of the people fetch water used for domestic use from?

**P 6:** Most of the people here in Bangwe fetch water from the boreholes and wells. Of course, we have a water kiosk in our community but the problem is that people are only able to fetch water from source once in a week and the other days taps are always dry. In most cases people fetch water from the wells and boreholes.

**F:** But you have said that there are few boreholes here?

**00**: Yes.

**F:** Let us now look at Malaria, you have said that Malaria is an issue here, right?

**00:** Yes.

**F:** In your opinion, why is Malaria an issue here?

**P 6:** There are a lot of places with stagnant water that acts as bleeding ground for mosquito and due to this, mosquito's bites here happens both during the day and night.

**P 7:** Most of the people do not have Mosquito nets, hence they don’t sleep under the mosquito nets and this exposes them to Mosquito bites.

**F:** What makes people not to sleep under mosquito nets?

**P 3:** In the past we were being given two nets by the government and but it’s been three years now without us getting the nets from the government hence the nets that we got last time from the government as worn out now.

**F:** How do people use the mosquito nets here in Bangwe? Do people use these after receiving them from the government?

**P 6:** Of course, people use these nets accordingly but not all, some of the people use the nets in making there gardens and some use the nets in drying maize. This frustrates those who give us the nets as they spend a lot of money in buying the nets for us and it is very painful to them to see people using the nets for other purposes rather than what they are intended for. I would say that maybe this is the reason why the government is no longer providing us with the nets now.

**00:** I will say that these nets were given to us a long time ago.

**00:** It should be 3 or 4 years ago.

**00:** We got them in 2016.

**F:** Three years ago?

**00:** Yes, since the change of the regime, we haven't received the nets.

**00:** 4 years.

**00:** Is it 4 or years?

**00:** [Not clear]

**F:** You were told that you should use the net for how long?

**00:** When one washes his/her net frequently, it is recommended that the net should be used for a period of two years because after this period the nets are worn out but if that is not the case, the net last for three years or more years.

**P 4:** It just depends on how one is taking care of the net and in some family two or three people sleep under one mosquito nets and in this case net does not last longer.

**00:** It also depends on the type of bedding that one is uses, when one is using a mat, the net does not last long as compared to when one uses a bed, the net last for a long time.

**F:** What prevents people from using the mosquito nets to reach a point of selling or using the nets in their gardens?

**P 6:** Some of the people believe that the mosquito nets attract and encourage the bleeding of bed bucks hence they use the nets in their gardens or selling it to the fishermen.

**P 4:** Some people have skin burns when they sleep under a mosquito net maybe it’s due to the chemicals that are applied to the mosquito nets. I would say that this is one of the factors that prevents people from sleeping under mosquito nets.

**P 2:** Some of the people say that when they sleep under mosquito nets, works of the bed do not go well.

**F:** What kind of bed works?

**00:** Sexual performance is decreased when one sleep under a mosquito net that's why most people sell the nets after receiving them.

**F:** Who complaints about this? Is it the man or the woman?

**00:** Mostly it is men who complain about this.

**F:** Men where do you get this idea?

**P 5:** These are some of the beliefs that people hold but I would agree with what some of the people say that they usually have difficulties in breathing when they sleep under a mosquito net because of the chemicals that are applied to the mosquito nets, that really happen. If by accident one covers his/her face with the nets while sleeping, he/she experiences difficulties in breathing for 2 or 3 days, I once experienced that and those who experienced this claim that mosquito nets are not good.

**F:** What did you do for you to get better?

**00: It** only happened once and I was cleaning my face with plenty of water frequently.

**F**: Alright, what other diseases did you mention?

**00:** Sores.

**00:** Chicken pox.

**00:** Pneumonia.

**F:** Did we say Pneumonia or Chicken pox?

**00:** Both were mentioned.

**F:** Let's start with Pneumonia, what makes you to think that Pneumonia is an issue here?

**P 2:** Some parents in cold season, aaah I would give an example, there are some women who earn a living through sand mining and they does that in the rivers that are found in our communities. Such women when they are mining the sand they usually leave their young children on the river banks where they are exposed to cold weather and due to this, children of this women suffer from Pneumonia very often.

**F:** Let me hear from others, what do you think causes to more cases of Pneumonia here in Bangwe?

**P 6:** The major contributing factor is the one that my colleague has mentioned that Pneumonia in children is caused when the children are exposed to cold weather especially when they are not clothed with specific cloths to protect them from cold. I would say that most of the children here are suffering from Pneumonia because they are exposed to cold weather.

**P 4:** It is not true that Pneumonia is caused by cold weather. The microorganisms that are responsible for causing Pneumonia are already in our bodies but they are activated when one is exposed to cold weather, Pneumonia is not caused by Cold weather because we are born with the organisms that causes this disease, it’s just a matter of being exposed to cold weather or not.

**F:** In which months of the year do cases of Pneumonia increase?

**00:** During cold months.

**00:** I would say that there is no specific time for this disease, some people we hear people saying that their children are suffering from Pneumonia but in hot season. Meaning that there is no specific time for this disease. The thing is we are born with the organisms that are responsible for causing this disease in our bodies and they are active in cold weather.

**F:** But someone else said that this disease is common in cold season?

**00**: Yes I said that this disease is common in cold season because my child was once suffered from this disease and it was in cold season, I took him to Luwanda health center since that time I was staying in Machinjiri. At the clinic I was told to make sure that my child is not exposed to cold weather and that I should ensure that he wears cloths to protect him from cold. I was told that if the child will be exposed to cold, he will be always suffering from Pneumonia.

**F:** Alright, what about Chicken pox? What makes Chicken pox a problem here?

**P 4:** I would say that in this community most of the children suffer from Scabies and Chicken pox because of shortage of water clean and safe water. Due to scarcity of water, most of the children bath in rivers which are already polluted and the germs that causes both chicken pox and scabies are found in the rivers but most of the people are not aware of this.

**F**: What else?

**P 5:** Scabies is a contagious disease, most of the children suffer from Scabies not because they bath in the rivers that are found here but due to the fact that they are fond of touching or coming in contact with their fellow children suffering from Scabies as well as exchanging clothes with such kind of children .

**F:** Like I said each and every disease that you have mentioned here is a problem, right?

**00:** (00) Yes.

**F:** But if we could be given a chance to choose among these diseases that you have mentioned, which diseases can you say are very common here in Bangwe or Malawi as a country?

**P 1**: Diarrhea.

00: Malaria.

**00:** And Scabies.

**00:** Chicken pox.

**P 5:** I would say that the diseases that are very common here are Diarrhea, Malaria and Pneumonia and only few children suffer from Scabies.

**00:** Scabies.

**00:** Chicken pox.

**00:** Pneumonia.

**F:** Now, you can agree that the diseases that are common here are Malaria and Diarrhea, right?

**00:** Yes.

**F:** What is the third one?

**00:** Scabies.

**00:** Pneumonia.

**P 2:** Pneumonia because Scabies is a just something that is common within a specific period of time and this is disease is caused due to unhygienic places at home meaning that it can easily be prevented by practicing hygiene at our homes whereas Pneumonia is a dangerous disease and it cannot be prevented in a simple way just like scabies.

**F:** Maybe if we can compare these two diseases (Scarbies and Pneumonia) which one is responsible for more deaths here?

**00**: Pneumonia.

**F:** Meaning that we can say that Pneumonia is a third one?

**00:(**All) Yes.

**F:** Am not trying to say that the other diseases are not dangerous but I want to know about the diseases that people of Bangwe consider as a burden. It is clear that the risk of suffering from these diseases is high here in Bangwe, right?

**00:** Yes.

**F:** What do you do when you are not feeling well for you to get better?

**P 6:** When one is sick, we take him/her to the hospital so that he/she gets should assistance, if there are no drugs at the hospital, doctors usually prescribe drugs and refer us to by drugs in the private pharmacies.

**F:** Mmmh, what else?

**00:** Silent.

**F:** We always go to the hospital when we are not feeling well?

**00:** Mostly we go to the hospital.

**F:** Are you trying to mean that each time when one get sick, he or she goes to the hospital?

**00:** (All) Yes.

**F:** You mean everyone?

**00:** There are some whose religion or beliefs restricts them from going to the hospital.

**F:** Religion?

**00:** Yes.

**F:** Which religion is that?

**00**: Apostolic church.

**00**: Apostolic church members are prohibited from going to the hospital, they believe that when a child sick, they should pray for him/her.

**P 5:** Some of the people use herbs.

**00:** They go to the herbalists.

**P 5**: They don't go to the herbalists; they just buy and herbs.

**F:** What the difference?

**P 5:** The herbalists are those who practice witchcraft but the herbs are usually bought from people who have knowledge of the herbs but they don't practice witchcraft.

**F:** We are learning a lot, some sought assistance from the religious leaders, others use helps.

**00:** Some go to the hospital.

**F**: Is that all? Are there some places where people go when they are not feeling well?

**00:** Some goes to fellowships (prayer gatherings).

**F:** At the churches?

**00:** Yes.

**F:** You also mentioned that when there are no drugs at the hospital, you are told to buy the drugs at the pharmacy. Now I just want to know which drugs do the doctors mostly prescribe for us to buy at the pharmacies.

**P 5:** They only drug that is always available at Bangwe health center is GV but not other drugs. The only bad thing at Bangwe health center is that whenever they have run out of the drugs, they don't tell you in good time and they keep you waiting for some hours only to tell you that they don't have drugs at last.

**00:** The only drug that is always available at Bangwe health center is Panado.

**F:** Only Panado?

**00:** Yes they give you three tablets of Panado and ask you to buy the other the drugs on your own.

**F:** Apart from shortage of drugs at the hospital, are there other factors that prevents people of Bangwe from going to the hospital?

**00:** At this clinic, the doctors do not welcome us with respect. Sometimes they shout at us without any proper reason.

**P 8:** The other thing is that in most cases there are no drugs at the hospital and most of the people are discouraged knowing that even if they can go to the hospital, they will not get the drugs.

**P 5:** To say the truth, the clinic serves a lot of people from this community and that's why there are not drugs at the clinic in most cases. I would say that the high population growth has created some pressure on the drugs at this clinic.

**P 6:** When you go to the ho, they tell you that there are no drugs, and they give you the prescription of drugs to buy at private pharmacies. The funny thing is that they usually direct us to buy those drugs from such and such pharmacy. So I just wanted to know, how do they (health workers) know that such drugs are found in such and such pharmacy?

**00:** Are you trying to ask a question?

**00:** Yes.

F: We are discussing and it ok to have questions in a discussion like this, she is asking that when she goes to the hospital, she is told that there are no drugs but she should buy the drugs from a specific pharmacy, so she was not as regards to how the health care workers so that the drugs are available at that pharmacy?

P 5: They know the owners of the pharmacies and they know the drugs that those people sell, I must say that there is a good relationship between the health workers and the owners of the pharmacies. This is common to us who runs businesses.

**F:** What does this mean to the people from our communities when doctors refer them to a specific drug store for them to buy drugs?

**00:** This makes us to be suspicious that there is something going on between the doctors and the owners of the pharmacies.

**00:** Just be open that the doctors steal drugs from the public hospitals and sell them to the owners of the pharmacies.

**00:** Thus right and we are able to buy some of the drugs from the clinic from some people in our communities.

**00:** Sometimes we are told that there are no drugs at the hospitals but when someone else who knows the doctors goes to the same hospital, he/she is given the drugs.

**F:** Apart from the pharmacies, are there other places where people go and buy drugs?

**P 12:** If there are no drugs at the hospital, we sometimes go and buy the private clinics.

**F:** Where else?

**00:** Some think of the distance that they travel to get to the hospital whereby for them to travel to long distance to get to the hospital only to be told that there are no drugs they consider that as something bad, as such, such kind of the people do not like going to the hospital.

**00:** It is only good to those who have money as it is difficult for those who don't have money to go and buy drugs at private clinics.

**00:** When one goes to the private clinic first and then later decides to go to the public hospital, the health care workers shout at him/her and sometimes refuse to assist such people. Some doctors in public hospitals feel that when one goes to the private clinic it means that he/she does not trust them but when you go to the public hospital, you are always told that there are no drugs and that you should buy from a pharmacy.

**F:** What do you do when you don’t have money and you don't want to go to the hospital at the same time?

**00**: I just go to QECH.

**P 3:** Here in Bangwe most of the people does not go to Bangwe clinic but Limbe health center. This is due to the fact that at Limbe health center, we are always given the drugs and its very rare not find drugs at this clinic and if it's something serious they usually refer you to QECH easily.

**F:** Meaning that drugs are usually available at Limbe health center unlike at Bangwe?

**00**: For one to receive drugs at Bangwe health centre, it means that one has to be there when the drugs are being offloaded by the authorities from Central Medical Stores.

**F:** Meaning that you only receive drugs during the time when the authorities from central medical stores are offloading drugs at the pharmacy?

**00:** Yes.

**F:** Why does it happen like this?

**00:** I don’t know why things happen like this but the truth is that we are able to get the drugs only when they are offloading the drugs at the facility.

**00:** A lot of people now goes to this clinic during days that the drugs are offloaded by the authorities from the central medical stores and sometimes we are not given proper drugs.

**F:** When you say proper drugs what do you mean? what drugs do you consider as proper?

**00:** Amoxicillin and Panado, when we are given these drugs, we are glad that we have been given proper treatment.

**F:** What about others, what do you consider as proper treatment when you go to the hospital?

**00:** Most of the parents likes to go to the hospital when their children are sick unlike a situation where they themselves are sick and when we get to hospital with children who are suffering from cough, instead of giving us Amoxicillin that cures cough, we are only given Panado. There are times when one whose child is suffering from cough is given Panado and another person whose child is having the same problem is given Amoxicillin. To me the treatment that I consider as proper, it’s when my child who is suffering from cough is given Amoxicillin syrup and not Panado . This is due to the fact that Panado does not cure cough in children.

**F:** She said that in most cases us, adults we don't like going to the hospital and they usually go there when their children are sick, why is it like this?

**00:** Yes that's true in most cases we usually go to the public hospitals so that the doctors should have evidence that sometimes we go to the public hospitals because if the doctors check in our health passports and discover that we usually go to the private hospitals, they don’t assist us and they tell us to go and seek for assistance at the private hospitals. So, we usually got to the public hospitals so that the doctors should have evidence that sometimes we trust them as well by going to the public hospitals.

**F:** Does this happen very often? that sometimes the health care workers are not willing to assist you due to the fact that at some point you sought help from the private clinics?

**00:** Yes that is happening.

**F:** Aaaah we have mentioned about the drugs that you are prescribed to buy at the private clinics, now I just want to know about the distance that you travel for you to get to the hospital. Basically, I want to know about the communities that are very far from the Bangwe health centre and in terms of money that they use for transport to get to the clinic.

**00:** Most of the people here in Bangwe stay in areas that are very far from the clinic. For instance, there are some people who stay at Chiswe, Mbalame villages and it is hard for someone from those areas to get to the hospital especially when their children are suffering from Diarrhea. It is very painful for someone living in such areas to get to the hospital and to be told that there are no drugs.

**F:** How much is the transport from these villages to Bangwe health centre?

**00**: Most people from these areas walk on foot to get to the clinic but for one to use a motorcycle, it cost him/her K1,500.

**00:** From my community, the transport is K 1000 but for someone from Chiswe let's put it at K 4000 to and from the clinic.

**00:** But during the night from Chiswe to the clinic, they charge K 2000 one way.

**F:** Where exactly is Chiswe?

**00**: It is close to BCA here in Bangwe.

**F:** As for me from Mpingwe primary to this place, I pay K 1000.00.

**00:** It is where cheap from that place to this place but from here to that place they charge K 1,500.00.

**P 5:** The motorcycle business operators are business oriented and they know that when one is stranded due to sickness of the child, he/she can pay any amount. In addition to this, someone who stay at Mtopwa and someone who stay at Chiswe cannot pay same amount of transport to this clinic. This is due to the fact from Mtopwa to the clinic, it is not more than 1 kilometer, so I would say that the amount of money people from different villages to get to the clinic is not the same. so it becomes difficult for someone whose is suffering from Diarrhea and stays very far from clinic to get to the hospital. I understand without getting proper treatment, someone suffering from Diarrhea can die within a period of 30 minutes.

**F:** What would happen to someone who is suffering from Diarrhea and takes 30 minutes without going to the hospital?

**P 5:** When one is having Diarrhea, he/she loses a lot of body fluids and there is this other disease that one vomit and has Diarrhea at the same time.

**00:** Cholera.

**P 5:** Sorry I forgot that it is Cholera, when one who is suffering from Cholera does not go to the hospital immediately let's say within one hour, he/she can easily die.

**F**: In a situation where a child is suffering from Diarrhea, what do we do?

**00:** In the past the health care workers were providing 'Thanzi ORS' to each and every family with young children in our communities.

**00:** In our community there are community health workers who provide us with ORS during the times when cases of Diarrhea are high in the country so that when a child start having Diarrhea during the night, he/she should be given ORS.

**00:** We don’t receive that.

**00:** Maybe they give you that because they are your friends.

**00**: [Unclear noise]

**00:** They were proving Thanzi to families with young children Mrs. Robert, that's not right.

**F:** When were they distributing ORS?

**00:** During the time when this area was hit by Cholera.

**00:** ORS is not only distributed during the time when cases of Cholera are high.

**00:** They were distributing Thanzi when cases of Cholera were high, that was the time they were providing us with Thanzi.

**P 2**: During the time when Cholera cases were high, we were being given Chlorine not ORS.

**00**: Yes, that what am aware of as well.

**F:** Meaning that people in the communities are given different things?

**00:** Even in our communities some people are given these things while others not.

**P 2:** Let me explain, in the past when we approach the health care workers to give us ORS, they were giving us but now they tell us that they are no longer distributing ORS to the people in the community because as of now ORS is scarce and it is therefore found at the hospital.

**F:** Are there other things that we do to help our children who are suffering from Diarrhea apart from taking them to the hospital?

**P 9:** We make a solution of sugar and salt and give it to them.

**P 2:** We were told by the HSAs that we cannot know the appropriate amount of salt and sugar that is required to make home-based ORS but they always encourage us to take the one who is suffering from Diarrhea to the hospital. In addition to this, they usually encourage us to give the one who is suffering from Diarrhea protected water as we are taking him/her to the hospital and that if we can afford to buy ORS on our own, we should do that.

**F:** What do you mean when you say protected water?

**00:** Protected water is the one that is treated with Chlorine or water guard.

**F**: Alright, are there other things that us parents does on our own to help the child who is suffering from Diarrhea, of course you have said that sometime you use herbs, right?

**00**: Yes.

**F:** What else do we do to help the child who is suffering from Diarrhea before making a decision of going to the hospital.

**P 4:** We give them water that is extracted from rice when it is being cooked and give it to the child. In addition to this, like what my colleague has already said, we make a solution of sugar and salt and give it to the child.

**F:** We have discussed about the causes of Diarrhea, what we do to cure this disease, right?

**00:** Yes.

**F:** What about in terms of prevention, how do we prevent Diarrhea? Let's start with how we prevent Diarrhea at our household level.

**P 6:** Taking care of the house like sweeping the house and disposing garbage in the dust bins, mopping the floor and ensuring that the kitchen utensils are clear always. Water that is used for cooking should be kept in a clean and covered bucket and making sure that we bath properly always.

**P 1:** There is a need to clean the pit latrines with Chlorine or ash on daily basis and making sure that the hole of the pit latrine is always covered with a lid.

**P 12:** There is a need rubbish pits at each and every household to be used in disposing garbage and we should always make sure that we wash our hands before preparing the food, breast feeding the child as well as after changing the baby diaper.

**F:** You said that there is a need for us to have a household rubbish pits, right?

**00:** Yes

**F:** In your opinion, are there waste bins in our respective communities?

**00:** There are no waste bins in our households and community, we usually dispose our garbage in rivers.

**F:** What prevents us from having rubbish pits in our households?

**00:** Shortage of land.

**00:** Due to population growth, there is shortage of land.

**00:** Some people are discouraged from digging rubbish bins in their compounds as a safety measure for children.

**00:** Some people dispose their garbage in the sacks and when the sacks are full, they dispose the sacks containing rubbish in the rivers.

**F:** What about in the communities, are there any other strategies that are used in dealing with Diarrhea at community level?

**00**: Silent.

F: Let me ask in this way, as a community, how do you prevent the spread of Diarrhea?

P 4: After preparing the food, we always make sure that the food is covered appropriately so that it should not be exposed to the flies that are responsible for causing Diarrhea and before our children eat, we always make sure that they wash their hands. We always teach our children that before eating the fruits, they should wash them with clean water, that's what I encourage my children to do but not all parents does this. In addition to this, there is a need for the community health care workers to teach us how to practice hygiene in our households as well as other ways of preventing the spread of Diarrhea at household and community level.

**P 5:** The major problem in this community is the way how people build their pit latrines, most of the people build their pit latrines in places that are not recommended for that. In addition, these days due to modernization, people are now using disposable baby diapers and most of the people who uses these diapers, dispose them in the rivers and some of the people use water from these rivers for domestic purpose.

**F:** If people can stop using the disposable baby diapers and use the cloth diapers, what can happen?

**00:** Our communities would be clean because it is impossible for a woman to dispose used cloth diaper in rivers, mostly these kinds of diapers are washed and reused.

**P 8:** There cannot be any changes because when women wash the cloth diapers, they don't dispose the water containing children’s excreta properly and some of them they don’t wash their hands after removing the cloth diapers from their babies.

**F:** So, we can say that the disposable diapers prevent women from touching their children’s excreta directly but on the other hand due to illegal disposal of these diapers people face problems. The cloth diapers are good because women can dispose their children’s stools in places like pit latrines but the problem is that when using this kind of diapers, they touch their children’s stools directly.

**00:** The use of cloth diapers also contributes to non-hygienic practices in our communities, these is due to the fact that most of the women consider rivers as appropriate places of washing cloth diapers and some people use water from the same rivers for domestic purposes.

**P 6:** There is no difference because the disposable baby diapers are disposed in rivers and the cloth diapers are also washed in the same rivers, so it is just the same.

**00:** But to me I feel like there is a big difference, apart from disposing the diapers in the rivers, people can set them ablaze but most of the women choose to dispose them in the rivers deliberately and some people disposer the diapers anyhow such that some children sometimes to play with the used baby diapers when they come across them. As with cloth diapers, women can wash these diapers at home and dispose the wastes in their pit latrines.

**F:** Who is responsible for disposing the baby diapers, men or women?

**00**: Women.

**00:** This is the responsibilities of both parents.

**00:** But in most cases it is women who are found at home.

**00:** But men can also teach women about hygiene practices.

**00:** When it comes to taking care of the children, that's the responsibility of the women.

**00:** Let me remind you, raising and caring the child are two different things.

**00:** In most cases men are busy with income generating activities while women are the ones who are have the responsibility of taking care of the children, so if the house is in unhygienic state. Can we say this is the problem of the man or woman?

**00:** Women.

**00:** Because they are the ones that are involved in taking care of the family.

**00:** It is usually women who dispose diapers in the rivers.

F: Alright, what strategies do you think can be used to eliminate the problem of disposing the diapers in the rivers?

**P 4:** There is a need to locate a special place that we should use in disposing the diapers and there is also a need to construct more boreholes in our communities.

**P 5:** Hygiene needs to start with oneself because the government can provide us with the facilities for disposing the used baby diapers but when one is used to dispose the diapers in places like rivers nothing can change. As such there is a need for us to do something on ourselves.

**F:** What do you mean when you say that there is a need for us to do something?

**P 5:** For instance, if there are more crimes being committed in a community, we are usually told that we should be a guard of one another, the same can also happen with women, when one woman is doing something wrong, her fellow women can advise her against that and other women can learn from her fellow women about issues to do with hygiene.

**F:** Alright, now let’s discuss about the vaccines that are administered to children.

**00:** Yes.

**F:** What do people say about the vaccines of children here in Bangwe?

**P 6**: When we allow our children to get vaccinated, people say things like our children will not be able to bear children and that the vaccines are being given to people so as to reduce population. Due to this some people are discouraged but still others are able to allow their children to get vaccinated.

**F:** Some say that those who got vaccinated won't be able to give birth, what do others say about the vaccines?

**P 2:** Before the coming of COVID19 and its vaccine, people were willing to get their vaccinated but a lot of people changed their perception towards the vaccines due to the coming of COVID-19 vaccine, people now say that the vaccines that are provided to the children are mixed with COVID-19 vaccine and that when children get these vaccines, they are going to join satanism. This discouraged a lot of people from getting their children vaccinated.

**P 7**: Some people are lazy when it comes to going to the hospital, it could be for the vaccines to be administered to people in their respective homes and not at the hospital.

**F:** What discourages people from going to the hospital?

**00:** People say that those who got vaccinated will die since the vaccines are meant to reduce human population and people regard this as true.

**P 4:** The major setback is that the vaccines are given to us before providing us with enough information on about these vaccines.

**F:** Alright.

00: If they could have been giving us all the necessary information about the vaccines before administering the vaccines, it could have been easy for people to accept that their children should be vaccinated because they would know the advantages as well as the disadvantages of the vaccines in advance. I would say that it is difficult for one to accept that his/her children should be vaccinated if he/she doesn't know have adequate information about the vaccines. To me I feel like there is a need to educate us about the advantages and disadvantages of the vaccines before they are administered to people.

**00:** Just want to answer the issue that has been raised by my colleague here that most of the people are not aware of the vaccine when it is being administered to people. Before the vaccines are administered to people in our communities, they there are always community awareness campaigns that are conducted by community health workers and we also receive the messages about these vaccines in our phones, churches, posters, hospital and the groups where we meet. They usually tell us that on a specific day, they are doing to start administering a specific vaccine, I remember before administering Polio vaccine they told us that they want to administer Polio vaccine to our children because polio causes deformities in children’s limbs. So, this issue that the health care workers should be moving around the communities telling people that they want to administer vaccine, that is already happening.

**P 9:** It is true that one cannot allow his/her child to get vaccinated without knowing the vaccine and its functions. if they are going to teach us about the vaccines before administering them to our children, it would be easy for us to accept that our children should be vaccinated. Most of the parents are not willing that their children should be vaccinated because they don't have adequate information about the vaccines.

**P** 2: I remember at some point in time I was called at a school where my child was learning, my child came home and told me that the authorities at her school wanted us parents so that we should discuss about a vaccine that was supposed to be administered to the girl at this school. When we went there, they told us that they wanted to administer that vaccine to the girls only from 9 years so that they should be protected from cervical cancer and that that vaccine is administered to young girls who are virgins and they gave us all the necessary information about that vaccine. However, most of the parents were not willing and they refused that their children should be vaccinated. They refused because we were thinking that if their children get vaccinated, won't be able to bear children, only few parents accepted that their children should be vaccinated and I was one of those who refused.

**F:** Mmmmh

**P 2:** When I got home, I told my daughter that she should not try to get that vaccine but after some time, one of the girls who got vaccinated became pregnant and this made us to reflect on the thoughts that we had that those who got vaccinated won't be able to give birth but now this girl conceived. And when ones who were administering the vaccine came to the school for the second time so that those who got the first dose should get the second dose, I accepted that my daughter should be vaccinated as well. Most of the children who got vaccinated that time are now married and they have children. I would say that it is important for us parents to understand that when the vaccines are being introduced, we should be curious to know the importance of the vaccine because sometimes we deny our children from getting useful vaccines due to some myths and misconception that are associated with the vaccines. My child got vaccinated and she has kids now, there was a time when people were refusing that their children should be given polio vaccine because most of the them believed that Polio cease to exist but as of now some children are suffering from Polio. I can conclude that there are awareness campaigns that are being conducted but most of the parents chose to believe that whatever they tell us is false.

**F**: I just want to know, where you get these messages that where you when your children get vaccinated, they won't be able to bear children, that they will join satanism. Where do you get these messages from?

**P 2:** I heard these from my friends, we were in a group and we were discussing about this and some were saying that they were being suspicious with these vaccines that are administered to children and some were saying that these vaccines are being given to people as a way of reducing human population as a result some of the parents do not want their children to be vaccinated.

**F:** What about others, where do you get these messages that when children get vaccinated, they won't be able to bear children and that they will join satanism, where do you get these messages?

**P 6:** There are some people who don’t want their children to get vaccinated so they came up with a plan to convince others that they should follow what they think is right and they spread such messages so that they friends should not get vaccinated their children as well and due to this some of the parents do not allow their children to get vaccinated.

**F:** What about others, where do you get this information from?

**P 4:** Mostly it’s because of the messages that circulate on the social media platforms. There are some people who circulate messages on social media platforms about different types of vaccines even before the vaccines are administered. When one has come across such kind of messages, he/she tells his neighbors about this and their neighbors develop negative perceptions towards the vaccines as well and the other thing that I can say is that rumors spread fast than something what is true.

**F:** What is the name of the vaccine that people were telling you that if your children get vaccinated, they won't be able to bear children?

**P 2**: HPV vaccine.

**F**: Alright, we have talked of Polio vaccine, what other vaccines are administered to children here in Bangwe? You have been quit, what is your number?

**P 3:** P 3.

**F:** This side you are quit as well as well, we are about to finish this discussion.

**00:** Ok.

**F**: Can you tell me the names of the vaccines that are administered to children here in Bangwe?

**P 5:** Malaria vaccine.

**P 6**: Rotavirus vaccine.

**P 2:** Polio Vaccine?

**F:** Cholera vaccine?

**P 2:** No, Polio vaccine.

**F:** Our colleague is thinking about this [laugh]

**00:** Chicken pox vaccine.

**00:** At the antenatal clinic children are given different types of vaccine.

**F:** Before the coming of COVID19, what was the perception of the parents towards the vaccine?

**P 2:** Before the coming of COVID-19, people were willing to get their children vaccinated. When we went to antenatal clinic at Namatapa, we could spend about 6 hours waiting for our children to be vaccinated but when COVID19 came, people are not allowing that their children should be vaccinated.

**F:** Why do you think those days most of the parents were motivate that their children should be vaccinated? Am asking everyone not only P 2.

**00**: (Alright).

**P 4:** Those days when one starts to attending the antenatal clinic, she was taught about all the vaccines that are administered to children and due to this, it was easy for the parents to allow their children to get vaccinated. However, with the coming of COVID19, most of the people deliver at the hospital but when it comes to go and get their children vaccinated at the antenatal clinic, they refuse for fear that their children will be given COVID-19 vaccine.

**F:** What makes people to think that their children will be given COVID19 vaccine at the antenatal clinic?

P 4: What makes a lot of people to think like this that I don’t know, for instance when COVID19 virus came, me and all my children got vaccinated, our neighbors used to come to our house and frighten us that after 6 months all of us will die. I was just telling them that it if was meant to be like that, then let the will of God be done. After 6 months to date no one from my family has died. Some were saying that there is a cross inside the injection that is in administering COVID19 vaccine.

F: [Laugh}

**00:** The one that was looking likes like a cross is Cholera vaccine not that of COVID19.

**P 5:** I remember this woman mentioned that some of the people do not go to the hospital because of their religious beliefs, the same also happen with the vaccines. Am not trying to blame those people who believe in prayers but they are the ones who tell people that vaccines are associated with Satanism. For instance, with COVID-19 vaccines, most of the religious people were saying that COVID19 was deliberately created and due to this most of the people were afraid to go to the hospitals as well as get the vaccinated. I would say that most of the people were afraid to get vaccinated because their religious leaders used the scriptures in warning their members about COVID-19 and vaccine. Secondly, the health care workers come in our homes with their cooler boxes and tell us that they want to administer a vaccine to our children without informing us of their coming and when you try to ask them, they don't provide adequate information about the vaccines and this discourages most of the parents to get their children vaccinated. Therefore, I will say that it would be good that before the health care workers come with the vaccines in our respective households, they should inform us in advance about their coming and that they should have adequate information about the vaccines so that when they are asked, they should be able to answer and that they should not be rude when are trying to ask them questions.

P 4: Before the coming of COVID-19, a lot of people were going to antenatal clinics with their children where they were being vaccinated without any problem but after the coming of COVID-19, people changed their attitudes towards other vaccines. This is due to the fact that after COVID19 vaccine was introduced, a lot of vaccines has been introduced as well and this made a lot of people to think that such vaccines are other forms of COVID-19 vaccine.

**F:** Let's assume, from last year to date, how many vaccines have been administered to your children or children of the people that you are aware of?

**P 4:** there are a lot of vaccine.

**P 9:** I would say that 8 or 9 vaccines, this due to the fact that few months after birth, the child is given 5 vaccines, after 9 months and a year, they are given other vaccines.

**F**: How many vaccines have been administered to your children or children of the people that you are aware of?

**00:** There is now Polio and Cholera vaccine on top of the 9 vaccines.

**P 5:** Since am not a woman, I don’t go to the hospital with my children but during the period that you have mentioned, I have seen that my children have been given vaccines for Malaria, Polio and Cholera.

**F:** What does it mean to us when our children are being given the same vaccine for the second time, what does this mean to us parents or the community?

**00:** It means that the lifespan of the first dose in the body of the child has elapsed and the other one is given to boost the immune system of the child again.

**P 2:** The health care workers told me that when they do that they try to boost the immune system of the children

**F:** What else the health care workers say?

**00**: Yes.

**F**: What about you parents, what does this mean to you?

**P 6:** To me I feel like without getting the second dose, it means that the children could suffer from those diseases that were vaccinated against at first but when they are vaccinated for the second time, it means that they can't suffer from those diseases anymore.

**F:** Someone said that children are given a lot of vaccines these days, right?

**00:** Yes.

**F**: Now I want to know, what are the problems that children are facing as a result of receiving a lot of vaccine.

**P 4:** Let me give an example of my children because I always make sure that they receive and every vaccine that is administered to children at a particular time. There is no problem that my children experienced as a result of getting vaccinated, I know a lot of say negative things but there is nothing bad that has happened to my children as a result of receiving the vaccines. I don't know if other people's children experience problems as a result of getting COVID19 vaccine.

**00:** The same also with my children, they have never experienced any problems as result of getting vaccinated.

**P 7:** There is nothing wrong with the vaccines and these vaccines are helping us a lot only that some people have negative attitudes towards the vaccines.

**P 2:** The problem that I experienced with the vaccine is……, aaah are you trying to mean about the children?

**00:** Yes.

**P 2:** My children have never had any problems with the vaccine.

**F:** But we can say that what is being said on the social media is the one that brings fears among the people when it comes to getting vaccinated, right?

**00:** Yes.

**00:** And what the churches teaches us.

**F:** I wanted to ask something about the religion, what does other churches like CCAP, Seventh Day Adventist church, Assemblies, Roman catholic say about the vaccines?

**00:** I would say that they encourage us to get vaccinated because we hear from our respective churches that a certain vaccine will be administered at a specific place and time. We are usually encouraged to vaccinate our children at church.

**00:** Not all churches encourage people to vaccinated their children, some have got nothing to do with the vaccines. I would say that mostly we get information about the vaccines from different social media platforms and not churches.

**00:** I can say that churches are playing a vital role in encouraging people to vaccinate their children, some people who do not allow their children to get vaccinated are the ones who doesn’t go to church and they therefore have limited information about the vaccines. I would say that most of the people who don't allow their children to get vaccinated are the one who are illiterate as compared to those people who are educated.

**F:** Where do you think you can learn appropriate information about COVID19 vaccine?

**P 4:** From the chiefs because when we hear that there is something that we should attend at the chief’s house, a lot of people are motivated to go there. When the community health workers are introduced at the chief’s compound, it is easy for us to accept them in our respective households. Most of the people are willing to vaccinate their children if they heard from their chiefs about the vaccines.

**F**: When I was asking about the vaccines, you mentioned about Rotavirus vaccine, are we aware of this vaccine?

**00:** Yes.

**F:** Is there anyone who has never heard anything about Rotavirus vaccine?

**00:** We have heard about it.

**F:** What do people say about Rotavirus vaccine?

**00:** (All) Silent.

**F:** In your opinion, what was the reaction of the people to Rotavirus vaccine?

**P 6:** Rotavirus vaccine was accepted positively because it was introduced before COVID-19 came and a lot of people accepted it but if it could come after COVID19, people could have had negative reactions towards it just like any other vaccine.

**F:** Are you trying to mean that most of the children here got vaccinated?

**00**: (All) Yes.

**F:** Where were they getting vaccinated?

**P 1:** Bangwe health center.

**00:** Antenatal clinic at Bangwe health centre.

**F:** If you can compare between the vaccines that are administered at the clinic and the ones that are administered at the households, which ones to most of the people likes most?

**00:** The one that is administered at the clinic.

00: The one that is administered at the household level since most some don’t like going to the hospital so it is easy for such kind of people to accept that their children should be vaccinated when the community health workers bring the vaccine in their respective homes.

**F:** Are there other people who refused that their children should not be vaccinated?

**00**: Yes.

**00:** For instance, at the clinic now people are being given drugs for gastrointestinal worms that are common among the children and there are some who refuse to get those drugs for their children and others accept.

**F:** Is there anything that you feel like should be changed in the way how Rotavirus vaccine is administered?

**P 9**: I feel like there is no problem with this vaccine and our children do not experience any problem after getting this vaccine.

**F:** The aim of the government is to ensure that each and every child should be vaccinated, right?

**00:** Yes.

**F:** But you have said that there are still some who don't want their children to be vaccinated. Now I just want to understand from you, what should be done for the vaccines to be administered to everyone?

**P 7**: I feel like there is a need for more awareness campaigns so that people should be given adequate information about the vaccines.

**P 5:** There is a need for the government to collaborate with various stakeholders such as chiefs, religious leaders and the teachers. It is important to collaborate with the chiefs because they are found in each and every community and the religious leaders can make it easy for their members to have a better understanding of the vaccines and within the school setting, it is easy for the teachers to explain to their students about the importance of the vaccines.

**F**: What else, I know in most cases as we are discussing like, us (researchers) cannot tackle everything that you have experienced or maybe you might have had something that you wanted to talk about but we did not ask questions on that. We are approaching the end of our discussion, if you have anything to say please this is the time.

**00**: (All) Silent.

**00:** Can you come again?

**F:** I was asking about the strategies that can be employed to ensure that all children should have access to vaccines and some have mentioned community awareness campaigns as well as collaborating with other stakeholders such as chiefs, teachers and religious leaders.

**00:** Most of us women know and get vaccines of our children at the antenatal clinic and if the antenatal clinic is very far from us, women are discouraged to go and vaccinate their children at the antenatal clinic. it is easy for women who stay close to the antenatal clinics to go and vaccinate their children as compared to those who stay very far. For instance, at first, we used to go and access antenatal clinic services at Hajra which is very far from this area and as of now, due to distance a lot of women are discouraged to go there. I would therefore suggest that, there is a need to have antenatal clinic close to us in our communities,

**F:** How far it is from where you stay to Hagra?

**00:** It’s a walkable distance but it is a bit far.

**00:** It is not very far but most of us we are lazy to go to Hajra, but it is not very far from us.

**00**: At this clinic there are usually a lot of people meaning that we have to wait on a queue for a long time before being assisted. This means that when we go there, we waste the time that we could have use for other productive activities. so if they the antenatal services can be brought close to us in our communities, it would be easy for us to go and vaccinate our children.

**F:** Meaning that the mobile antenatal clinics are very helpful?

**00:** Yes, rather than for us to go to the health center

**F:** If there nothing else, this marks the end of our discussion, thank you very much for being open and the interest that you have shown in the discussion that we had. When you heard that two of us were here, you made sure that you should come, I am impressed because we had a nice discussion as compared to the discussion that I have had with other groups. Once again thank you very much.

**00:** (All) Thank you.
