# Supplementary material for: Exploration of the social determinants of diarrhoea, rotavirus vaccine uptake, and vaccine ‘fatigue’ in Ethiopia, Kenya, and Malawi
Source: PLoS One. 2025 Sep 9;20(9):e0319691. doi: 10.1371/journal.pone.0319691 (PMC12419581; doi:10.1371/journal.pone.0319691)
Supplement: S1 Data — (ZIP) [file pone.0319691.s001.zip › Supporting Information Files/ET_4FGD.docx]

I: Thank you for your participation. What are the common diseases affecting infants in your village?

P3: I came from zone 2; the most common disease affecting the infants is something like common cold. In addition to that diarrhea is also common among children below the age of five. Currently, they are being affected with a flu pandemic that causes them to coughing.

I: You may also consider the common diseases in general, not only diseases on this particular time?

P3: Fever, common cold and diarrhea are common here.

I: Did you say diarrhea in?

P3: Yes, coughing and fever are also common. They frequently affect and most of the time they don’t became severe.

I: Thank you, any other?

P8: Most of the time, infants below the age of two are frequently affected with diarrhea. In time of ጥርስ ማብቀል, fever and diarrhea are common symptoms. As we usually take this kind fever and diarrhea are caused by ጥ.ማብቀል, we don’t visit health facilities for treatments. The health workers also tell us those are just the symptoms. In addition to these, we also take them to health facilities for treatments when such diseases happen.

I: So, what are the common diseases affecting children in your village?

P8: Common cold and tonsillitis are very common.

I: Very good. As you all came from different zones, you may tell different opinions.

P10: I think it is diarrhea, and the cause for this common disease on infants is bacteria.

I: Some of you have mentioned Tonsillitis, and some of you have mentioned common cold, diarrhea and fever. Which of these diseases is a threat in your village and becoming more dangerous?

P7: It is common cold. Infants like my four -Years -old daughter have being affected with common cold in kindergartens. As their immunity is less and the transmission is high, they have been affected with that frequently. Fever and coughing happens frequently and the medicine we usually give them as a treatment doesn’t give a cure. So, we can conclude common cold is very common than diarrhea and any other diseases.

I: Very good, any other?

P9: My daughter is four years old, I have brought her to health center and I have learned that, the disease was caused by bacteria and we need to keep our children's personal hygiene by washing hands, to prevent such disease. We need to make sure we serve children with fresh food and make sure their hands are clean before having meal and after they come from play.

I: So, the most common disease that has been a threat in your village is bacterial disease?

P9: Yes.

I: Any other, please keep participating? You have mentioned common cold, diarrhea and fever. Even the later is a symptom of a disease, please rank these diseases as First, second and third, in order of their threat they brought to your village and their danger?

P3: In addition to those diseases mentioned, there is also some diseases that create rash on children's face. That is because children in our village play near polluted rivers. Fever is the most common here. I have brought my child to private clinic and the doctor has told me it was just a common cold.

I: Any other?

P6: In our village, it is just a common cold.

I: Is that on infant children?

P6: Yes.

I: That is what has observed in general, not only from recent times?

P6: Yes.

I: Please, put them in order, from one to three?

P8: I think the common cold is seasonal here. But, the tonsillitis is a disease that affects children throughout the year. It happens more frequently even after three or two months after taking medicines. So, tonsillitis has become more frequent in my children’s case. The fever and pneumonia caused by the tonsillitis are the most dangerous in my experience, and I have been challenged with these.

I: Any other?

P1: In our village, diarrhoea is the most common. I think the cause is environmental pollution.

I: Would you rank them as first, second, and third?

P1: Yes, diarrhea is the first. Children with normal immunity can resist diarrhea. Some children can be more affected than others.

I: Very good, please mention the health facilities available in this village?

P9: There are just two health centres, including Teklehaimanot Health Centre.

I: Okay, you use the services of these health centers?

P8: Yes.

I: You may also mention any other health facilities you know; even you are not using the services from them.

P6: We only use health centers like Teklehaimanot because that is where we can afford the services.

I: How about the facilities serving the society?

P4: The health workers give home to home health services and health education about the prevention of diseases for infants. We were also told there would be a research on this, and we have had One-day training on Meskerem.

I: Are you talking about the orientation I had given you?

P8: Yes.

I: Just mention the health facilities available in this village?

P2: Ginbot Haya Health Centre.

I: Okay. Isn’t there any other health facility other than health centres?

P8: Can we mention private health facilities?

I: Of course, you can.

P8: We usually get the services from Teklehaimanot Health Centre and Tikur Ambessa Hospital when referred. There is no private hospital nearby. There is actually… Chechila hospital and some hospitals called Vision. The health centers are closer to us.

I: Is there any other health facility you get health services from, other than health center?

P1: There are private clinics and pharmacies, but we usually get health services for infants from Tekle Haimanot Health Centre and from health centres nearby for diseases like a common cold.

I: Any other different idea?

P5: We also get the services from Tikur Ambessa Hospital.

I: Good.

P7: I usually get health services from Coronel Mekonen. I sometimes get cured when he just touches me without any medication.

[All Other Participants]: *[Laughing]*

I: Okay, another?

P9: There are also health workers who regularly provide us home to home health services.

I: How much do they charge you for the services? You have told me that most of you use the health centres’ services. So, how much do they charge you?

P6: I have health insurance, so I can use that to get the health services at health centres for free. But, we can’t get all the medicines available from the health center, and we have to purchase these medicines from private pharmacies.

I: Any other?

P10: We just pay only for registry cards; we can get the services and medicines and vaccine services from health centers for less.

I: How much do they charge you for a registry card for infants?

P4: I had paid 40 birr for that, but I am still using that.

I: How about payments for laboratory and other related services to test?

P4: I have brought my child with a high fever and swelling on his body. Then, the doctor has just diagnosed the child and referred him to Tikur Ambessa hospital. The doctor I know in this health center have prescribed him some medicine before I went to the hospital and he get cured on the morning I had had the refer schedule, after I had applied the medicine at night. I have never had the service after that.

P3: Thank you, as they have told you we may get the services at the health centers for free. But, mothers may need to pay up to 1,000 for city scans and other services we can’t get in the health center.

I: At health centers?

P3: There is only some medicine available in health centers. The only medicine I had gotten from the health center for my child was Paracetamol; we had to pay 1,000 and more for any other medicine from private pharmacies.

I: Any other? How much do you pay to get the card and health services at private clinics?

P6: In Mekonen's clinic, you have to pay 200 birr for a card, 150 birr for children. You may also need to pay 400 birr for laboratory service, and I usually pay 2500-2700 birr for health services of children.

I: Any other?

P8: It is almost the same here. We usually go to Mekonen, as it is the closest private clinic we know. We don’t pay for the card and laboratory services we have at the health centre and Tikur Ambessa Hospital. It is because we have health insurance; we have to buy medicine that is not available here from private clinics.

I: Has the distance of the health facility from your residence been issues? How far are the health facilities like Mekonen? Perhaps you live in different zones.

P9: Most medicine we need have not been available here in the health centre, especially in 2016. I have had a postnatal checkup in Tikur ambessa Hospital. I have been very challenged to find a medicine for her. I had to go to Bole Arabssa to find that medicine for her. The health insurance is not providing medicine nowadays, we can’t even get medicine for money. The doctor has recently prescribed medicine that helps the baby to get strong, but I couldn’t find that. The prices for medicines are also not fair; we need to pay 4,000 or more for medicine. I don’t know what people with low income are doing for this!

I: Any other pleases?

P1: The health Centre is very clothes for us, and the health workers' integrity and services are also very good. The only problem is the unavailability of medicines; we have to buy medicine from private clinics.

I: So, even the health centers are close to you. You sometimes need to go far to buy the medicine you don’t get from health centers, right?

[All of them]: *Yes*

I: Have there been situations you couldn’t go to buy medicine because of the distance?

P3: Governmental health centers are built by considering the people living around them. But, the private clinics are like far from residents. For example, abera's clinic is found at Berbere Berenda, and D.r Mesfin's clinic is now at Mesalemiya. The private clinics are far from residents.

I: Okay, any other?

P8: It is not easy to get medicines; there was a time when I couldn’t find medicine for over a month. Pharmacies had been telling me the 600 grams of the drugs on the prescription were wrong, and the amount is very high for children, and the Tikur Ambessa doctors were insisting it was right. Finally, I found a professional from Tikur Ambessa who provided me with the exact drug. The health centre and Tikur Ambessaa hospital is closely located to us, but medicines are not available.

I: Good. Is there any other means you get medicines other than pharmacies?

P3: Yes, we also buy from Kenema.

I: That is also a pharmacy, right?

P3: Yes.

I: Thank you. How do you treat your child at home when they get diarrhea?

P6: I treat my child with a honey-lemon solution and with garlic.

I: Okay.

P2: We usually treat them with Tenadam with boiled water, and milk with garlic is another treatment we provide.

I: Okay, any other?

P9: I treat her with lemonade.

I: Okay.

P8: I treat her with lemonade with hot water. I let my children take that in when it tastes like glucose.

I: How about you guys, don’t your husbands help your family with treatments?

[All participants]: *laughing*

I: What changes do you see in children after those treatments at home?

P8: It usually gives them a cure. If that doesn’t work, I take them to health facilities. If they ask me why I didn’t bring them on time, I tell them I had been treating the child at home.

I: Okay, any other?

P8: Most of the time, it doesn’t stop diarrhea but stabs pain in the stomach. As long as they are eating, diarrhea is inevitable. I just treat them with Tenadam before I take them to health facilities.

I: Okay, good. Do you know any other treatments for diarrhea you heard from the community?

P5: Tenadam treatment is what most people recommend. They tell you to boil the Tenadam and let the patient drink the solvent with no sugar in it.

I: Any other?

P4: Most of the time, when children get diarrhea, we tend to think it is because of and we go to Wegesha ( people who give physiotherapy treatment traditionally). But, I don’t recommend that people use that.

I: Please elaborate on that?

P4: When there is diarrhea, some say it is a symptom of Kichit (traditional name given for vertebral subluxation) is believed that it is caused when their intestine is twisted. So, take them to wegesha.

I: Okay, any other?

P9: I personally know some elder women who provide traditional treatment at home. She had treated my child with ashes from charcoal and lemon when she had had a diarrhea. The women have told me the diarrhea is caused because her tonsil had inflamed. The lemon on my child had started to boil over, and the woman told people the bubbles from the lemon were indicators to show the patient had been cured.

I: Thank you so much. I have heard new treatment methods I have never heard before. Any other?

P2: I also took my child to my mother, and she treated him with lemon and ash.

I: Just like the person before said?

P2: Yes.

I: Where do you bring children when they get diarrhea? Tell me if there is somewhere you take them other than the facilities you mentioned earlier. You have told me you either took them to health facilities after three episodes of diarrhea or to traditional treatments. Are there any other ways you get the treatments for diarrhea from?

[All participants]: *Silent*

I: What are the opportunities and challenges that allow you to bring your child for diarrhea treatment?

P4: Sometimes, I need to bring the children to the health Centre at midnight. At that time, the doctors were unavailable and can’t serve the child timidly. That is the challenge I have experienced. In addition to that, they sometimes just refer patients without careful diagnosis.

I: Okay, any other?

P8: There are good opportunities in the health center. But there was a time when the health workers treated you wrong and don’t identify the disease wrong, like they didn’t diagnose the pneumonia case of my daughter. They just have told me it was a flu first. Finally, her noses started to discharge blood, and they referred her when they saw that symptom. The day I brought her was a holiday, and the health workers in the health centers recklessly told me to bring her to Tikir Ambessa the next day. That was irresponsible. The doctors in Tikur Ambessa, on the other hand, are very cooperative and take action immediately. The biggest challenge in health centers is the fact that there is no responsible person for consequences like death. So, I usually don’t bring my child to health facilities.

I: Okay, any other? What are the challenges and opportunities?

P3: What made us go there is the severity of the diseases after trying treatments at home. One of the challenges is the knowledge gap of workers. There was a time when they diagnosed my daughter with just a simple cough when I brought her after she had had a fever and diarrhea. But I learned it was pneumonia later. They just had been prescribed me syrup, and my daughter had almost died. I brought her to Dr. Mesfin he criticized me for not bringing her to him when I saw no change. He diagnosed her with pneumonia and prescribed me a medicine, and then she was cured. The workers in health center try their best, but they don’t exactly know the best cure for patients. That is what happened to my daughter.

I: Any other different idea?

P4: As he mentioned earlier, the attitude of the health workers at the health center is not good. There was a time when they prescribed me the wrong medicine, and my daughter's case became severe. I brought her to a private clinic, and she was immediately cured. The doctor in private told me I was wrong to take her there instead of bringing her to him or Tikur Ambessa. The workers in health centers should not be ignorant as they are dealing with lives.

I: Good, any different opinion?

[All participants]: No

I: What do you think is the cause of diarrhea in infants?

P5: I think it is caused by poor personal hygiene. Children may feed something infected, and that may cause the disease.

I: Any other?

P4: Our poor living style is one cause. We live in very low standards and scattered ways that pave the way for infections to be transmitted easily. Another cause is insufficient water supply of water; we just get water in terms. Some toilets don’t even have doors.

I: Good, any other?

P9: Not getting vaccines on time can be the cause. In addition to that, not serving them fresh meals is another cause of disease.

I: Any other? Please keep participating; you are becoming cold now

[All participants] : *[ laugh]*

P1: Some children may not like the meals we provide, and we sometimes tend to give them fruits like bananas. That may cause them the disease.

I: Okay, any other?

P8: Packed juices and sweets given to children by ignorant mothers may cause children diarrhoea.

I: Good, any other causes mentioned than poor hygiene and low living standards. You can also elaborate how low living standards are the cause?

P8: In our living area, the playing grounds of children are closely related to latrines and trashes. In some Addis Abeba villages, toilets and homes are built closely, and that makes children to be vulnerable to diseases. In addition to that, children may become infected because of the contaminated food we serve them. Amoeba and like diseases can be caused by such contaminated meals like tomato, so I usually refrain from serving them such like foods.

I: That’s good. What have you been going to prevent diarrhoea at home? You have mentioned the treatments earlier; tell me the prevention methods now.

P3: We usually boil and freeze water for our child to drink. The cause for the disease is the drain running over house, which is because of broken ditches. So, we usually don’t let her go outside home. There is also a packed juice sold for 5 birr; whenever she feeds that juice, she gets diarrhea, which is like a white bubble. So, I prevent the disease by not buying her such foods and restricting her not from getting out of home and reaching the ditches.

I: Good, any other?

P1: I don’t let my children feed chips and other meals bought from outside. That is because these chips are made from expired oils. The second protection method I use is to let my child eat fresh meals and make sure to wash them regularly as they are always touching dirty.

I: Okay, how about the other prevention methods you use to prevent your children from diarrhea?

P6: It is the same here. I boil water and disinfect the water from taps. I also regularly wash them before meal.

I: Any other?

P9: I regularly clean the house and make sure they their hands are clean before feeding them.

I: Very good. These were prevention methods you do at home. What do you do to prevent this diarrhea disease at the community level?

P8: It is not regular, but we have a trend of cleaning the environment every 15 days, especially the playing ground of children. Most importantly, I advised my child not to touch a speck of dirt and threaten her with injections by telling them she would get injections if she kept touching dirt.

I: Very good. What other prevention methods do you use?

[All participants]: *[silent]*

I: Why aren’t you participating? Are you tired?

[All participants]: *[It is just because it is the same method we all use]*

I: Okay, share your experience anyway let’s now discuss the vaccination. What is the understanding of the people in the community towards the vaccination for infants?

P4: Honestly speaking, the people are willing to take vaccination and know it is very useful. The health center is also doing great on vaccination.

I: Any other?

P3: I don’t think the community has got enough understanding of vaccinations. The people get their vaccination because they can’t get vaccination certificates unless they get their child vaccinated. Most mothers in my village think the vaccination is against religion and faith.

I: Any other, tell me what’s on your mind?

P8: The reason most mothers don’t go for vaccination, in my opinion, is because the children don’t get cured even after the vaccination for diseases like tonsillitis. For example, people get the vaccine for measles but still get their child affected with that disease. So they think it is futile to get a vaccination for the prevention of diarrhoea and vitamins. I also doubted that and asked the health workers about that, what is the purpose of the vaccine if the diseases still occur? That is useless. That is why most of us don’t give vaccination that much focus.

I: Any other?

P4: I believe vaccination is very important and I take my children myself if my wife can’t make it. It doesn’t work like the participant earlier mentioned; the vaccination for measles and others don't enable the children not to get affected by the disease at all. It just helps them to be more resistant to diseases because vaccines weaken bacteria. I have this awareness, and I know how it works, and there may be some acne on their face. It is like a war; our body fights with the external body, and that may cause some illness, but the vaccination made that easier. I know that, and I never let my child skip vaccination programs.

I: Very good, we are getting different ideas, one is saying it is more accepted in the community and the other is saying it is not. Do you have any different ideas, or which one do you agree with?

P5: I don’t want my children to skip vaccination programs; even if my wife is not that concerned about this, I always bring my child for vaccination. They wouldn’t get them vaccinated if that doesn’t help. So, I want to argue that vaccination is very vital.

I: Why is your wife not concerned about vaccinations?

P5: She doesn’t think it is that important, so I have to take that responsibility because it is very important.

I: Any other?

P1: Not only the parents, but the health workers are also making sure everyone gets the vaccination. They always follow up and check home to home if there are children who doesn’t get the vaccine. There is better implementation of vaccination now than before. The people have accepted it well, and the health workers are also doing great. The people’s awareness is now better now.

I: Good, every one of you represents your own village so you all have different experiences. His experience may not be the same as you. So, do you have any different ideas on this matter?

[All participants]: *[silent]*

I: If there is not, let’s discuss the Rota virus vaccine now. Is there anyone who knows about the Rota virus vaccine?

[All participants]: *[confused, and nod to say no]*

I: You have been taking the vaccination, though.

[All participants]: *[no]*

I: You are taking the vaccination as you do it for vaccination for vitamin.

P8: Most of us don’t ask about the vaccines when getting vaccination services.

*[Laughing]*

We just ask about the vaccines when our children get sick. We just brought them till they are five, because we are told to do so. The health worker at Tikur Ambessa once asked me about the type of vaccine my child had taken, but I couldn’t specifically tell him which vaccine it had had. The people identify the vaccine with their vaccination schedule; we know them as “the vaccination on 9th month, or 8th month” and like that. We don’t usually ask what the vaccine specifically is.

I: Okay, I can’t elaborate you about the vaccine. But it is the vaccine you had had in the form of droplets, to prevent diarrhoea. So what is the attitude of the community about this vaccine? It is the vaccine that is introduced after 2,000 E.C unlike Polio vaccination which had been introduced very early. It is given for three times at the time you go for the vaccination you take in through tighs. What kind of attitude does the community has over this vaccine?

P8: I have had a chance to round with the nurses. What I observed from that is the mothers don’t specifically know the name and purpose of the vaccines. They just refer the vaccine by using the timing they take it. But they all are aware of the benefit we’ll.

I: You may heard about the side effects of the vaccine and related comments from mothers, what are they and what is the community’s attitude towards the vaccine?

P3: I have a different opinion. I just recognised the rota vaccine when you have told us it is given in form of droplets. Most people know only the droplets on eye before experiencing this. The community doesn’t know the purpose of the vaccine and don’t know the side effects and that is because the health workers don’t explain the side effects. For example, I have knowledge of the same method of disease prevention on hens. The side effects are that they feel tired and feel dizzy for three days. We are explained this before treating the hens with the vaccine like droplets. So, the health workers should explain in the same way and let the people know the side effects like the acne and feeling of dizziness. The people just get their child vaccinated and go home.

I: Where is this vaccine given?

P2: They give our child the droplet vaccines on the 45th date. We don’t know the purposes, but we know it’s important. After the day my daughter gets sick, I have started to ask the benefits of the vaccine like the vitamin. Most people don’t know the purpose and what the vaccine is because no one explains that.

I: So, where is this vaccine given?

P1: In the health centres,

I: Have you ever gotten these vaccination services at home?

[All participants]: *[yes, it is given]*

I: You get the services for free, right?

[All participants]: *[Yes]*

I: What are the opportunities and challenges that enable you to take the vaccination service?

P1: What made us vaccinate our children is the need to see changes in children health. Traditional methods of treating children had been very dangerous, and many had died because of that. This vaccination has solved this problem. The people don’t know the exact purposes of each vaccination, but we all are committed to getting the vaccination services because it helps us to prevent children from diseases.

P8: I am well aware of the benefit of the vaccine now. I have learned which vaccine is given for what. So, it is a good experience we have got and it will be very useful for the next time we get new-born children.

I: What religious and cultural beliefs prevent the people from getting vaccine services?

P3: I think vaccination is a prevention given beforehand to prevent disease that possibly affects children. The Corona virus vaccine, for example, was not accepted by many people and was considered as some work of devil or illuminate, and it was taken as something against religious teachings. It affects the vaccination services of other types.

I: Okay, so the vaccination for Covid 19 has an impact on other vaccinations, right?

P3: Exactly, there were beliefs among believers about the vaccine.

I: What kinds of beliefs?

P3: The religious fathers were not appreciating the people to go for vaccines; later, they started to do that. They didn’t keep doing that for long. In general, the peoples’ attitude towards vaccines is better now.

I: Any other opinion please? If there is none, is there a person who is concerned about the safety of a vaccine? It is the last question, so please participate.

P9: We regularly take the vaccine because we want our children to be safe and don’t want to be affected by the disease

I: She was mentioning that some believe the vaccine as something futile and being affected by the disease is inevitable. Do you have similar opinions on the safety of the vaccine?

P3: Some people complain about the vaccine given as injection because they fear not to get injected on the wrong blood vessel. As I had a chance to round some patients with the health workers, I have heard many complaints about this.

I: Okay, do you have any other opinion? You may have come up with ideas when listening to others. So, do you have any?

[All participants]: *[no]*

I: Okay, if not, I am done asking now. If you have anything to add, let me give you the chance.

P3: We don’t have.

I: Okay. Thank you all for your participation.
