# Supplementary material for: Exploration of the social determinants of diarrhoea, rotavirus vaccine uptake, and vaccine ‘fatigue’ in Ethiopia, Kenya, and Malawi
Source: PLoS One. 2025 Sep 9;20(9):e0319691. doi: 10.1371/journal.pone.0319691 (PMC12419581; doi:10.1371/journal.pone.0319691)
Supplement: S1 Data — (ZIP) [file pone.0319691.s001.zip › Supporting Information Files/MW_1FGD.docx]

**F:** Thank you very much, I would like to thank you for accepting to participate in this study. Aaaah let’s start our conversation with what you know about Diarrhea and Rota virus vaccine, your experiences when you go to seek for assistance at the hospital as well as when you are seeking assistance for your children aged below 5 years who suffering from Diarrhea . So, I just want you to explain to me about diseases that are very common among the children in this community (Mpingwe/Bangwe).

**P3:** The diseases that are commonest among the children in this community are Pneumonia, Diarrhea, flue and cough. These are the diseases that most of the children suffer from here.

**P 6:** Skin sores.

**F:** What?

**P 6:** Skin sores.

**P 9:** Don't forget Polio.

**F:** What?

**P 9:** Polio.

**F:** Is Polio one of the diseases is that is common amongst children here (In Mpingwe)

**P 9:** Yes.

**P 10:** High body temperature (fever).

**F:** What kind of disease is this?

**P 10:** It is usually associated with Malaria and skin sores in children.

**F:** Is Malaria a big problem here?

**00: (**All participants) Yes.

**F:** Mmmmh.

**P 1:** The disease that is more common amongst children here is Diarrhea.

**F:** You have mentioned a lot of diseases such as Diarrhea, fever which some say is associated with Malaria and others diseases such as Polio, Flue, cough, Pneumonia, Sores, right?

**00:** (All participants) Yes.

**F:** So out of these diseases that you have mentioned, which diseases do you think are more prevalent (common) in this community?

**00:** Diarrhea.

**00:** Diarrhea

**00:** Diarrhea and Malaria.

**00**: Pneumonia as well.

**F:** Pneumonia, Diarrhea Malaria and what?

**00:** Skin sores.

**F:** We are taking of diseases that are common amongst the children.

**00**: Skin sores.

**F:** Meaning that the diseases that are commonest among the children are these four?

**00:** (All).... Yes.

**F:** Let's start with one disease at a time.

**00:** Alright

**F:** Let's start with Diarrhea or else let me hear from you, out of these diseases which one is very common amongst children here?

**00:** (All) Diarrhea.

**F:** It is seconded by which disease?

**00:** (All) Pneumonia.

**F:** The third one?

**00:** (All) Malaria.

**F:** So, we can say that the fourth one is skin sores?

**00:** (All) Yes.

**F:** Now, let us discuss why do you think Diarrhea is a big problem here? Why do you think Diarrhea is a big problem here in Mpingwe?

**P 6:** What happens is that since the children are young, they just eat anything that they come across with and this end up in causing Diarrhea among most of them.

**P 3:** The other problem why most of the children suffer from Diarrhea it’s because of the water that we drink which are not clean. In most cases, this water is consumed before it is treated.

**F:** Why do you think the water that you drink here is not clean? what makes the water to be in such state?

**00:** Since we fetch water from the wells or boreholes.

**F:** Just a reminder, we agreed that we should give each space when he/she is taking, right?

**00:** (All) Yes.

**F:** Eheee,

**00:** We have a challenge of shortage of water here, most of us fetch water from the wells that are not usually covered.

**00:** Sometimes we fetch water from the boreholes with uncovered water buckets which we carry on our heads to our respective homes, meaning that on our way some germs can get into the water and once we get home, our children consume that water before it is treated and this exposes them to Diarrhea.

**F:** What sources of water do most of the people use here?

**P 6:** Wells that are mostly found along the river banks.

**00:** These are the rivers that some people use to dispose different baggage/wastes such as baby diapers.

**F:** Which water do you consider as clean?

**00:** Water from the boreholes and taps.

**F:** So, there are no taps and boreholes here?

**00:** We have boreholes but are usually found very far from our households.

**00:** There are a lot of people here but the sources of clean water are very few hence a lot of people use water from the wells. This is the reason why most of the people use water from the wells.

**F:** Alright, what about Malaria, what makes Malaria a big problem amongst the children in this community?

**P 8:** Most people do not sleep under treated mosquito nets.

**P 10:** People do not sleep under mosquito nets.

**F:** Let us proceed, you are saying most of the children suffer from Malaria because they do not sleep under Mosquito nets, right?

**00:** In a situation where one does not have a mosquito net, children are exposed to mosquito bites. Mosquito bites are usually common in hot seasons like this (September) and there are stagnant waters in most parts of this community.

**F:** So, you are taking of lack of mosquito needs as well as presence of stagnant waters in this communities?

**00:** (All) Silent.

**F:** Where do most of the people from this community go to seek assistance when they are not feeling well?

**00:** Bangwe health center.

**F:** Let’s start with P 2, you are not talking what’s wrong with you (laugh).

**P 2**: Most people go to Bangwe health center.

**F:** What do most of the people do before going to the hospital?

**00:** (All) Silent:

**F:** Can we say that each individual goes to the hospital when he/she is not feeling well?

**00:** No.

**F:** So, I would like to know what we usually do when we are not feeling well.

**00:** We usually go and buy medicine at the pharmacies.

**00:** We usually go and buy drugs at the grocery store or pharmacies.

**00:** Some go and seek assistance at the private hospitals.

**F:** You have mentioned about pharmacies, private clinics. We are going to look at these separately later on.

**00:** (All) Alright.

**F:** Let us start with buying drugs at the pharmacies, can you tell me the types of drugs that you are able to buy at the pharmacies?

**00:** Caffeino.

**F:** Mmmh.

**P 3:** Since we are used that when we go to the hospitals with a child who is having flu, we are used that we are given Amoxicillin or Bactrim. As such when a child is having cough, we just decide to go and buy Amoxicillin and Bactrim because we are used that when we go to the hospital with a child having flu, we are given such kind of drugs.

**P 10:** One of the reasons why we don't usually go to the hospital is that when we get there, we normally go through all the steps that are usually followed at the hospital but the problem is that when go to the pharmacy, we are usually told that they don’t have drugs and doctors usually refer us to buy drugs at the private pharmacies.

**F:** This one is explaining about factors that prevents people from going to the hospital, right?

**00:** Yes

**F:** But we were discussing about the drugs that we usually buy like Amoxicillin, Bactrim. Do you buy RA at the private clinic?

**00:** (All) Yes.

**00:** Flagyl, Caffeino.

**F:** So where exactly do you buy these drugs?

**00:** Pharmacies and groceries.

**F:** I also want to know about the amount of money that you spend for you to access medical services? But before we look into that we should look at other factors that prevents people from going to the hospital, this one has mentioned that most times when she goes to the hospital, she is told that there are no drugs and that she should buy the drugs at the pharmacy. What else do you think prevents people from going to the hospital?

**00**: Long distance to the health facilities.

**00:** The behavior of the nurses, in most cases nurses are rude when you approach them at the hospital and sometimes this discourages us to go to the hospital and this makes some of the people just to go and buy drugs at the pharmacies.

**00:** Some people are just lazy to go to the hospital because the treatment that they get when get to the hospital is just the same in several occasions. As a result, most of the people just decide to go and buy drugs at the pharmacies.

**F:** When you say treatment are you trying to mean the rudeness of the nurses or something else?

**00:** Yes, and sometimes when you go to the hospital, they just give you the prescription of the drugs and refer you to buy the drugs at the private pharmacy. As such most of the people just like to go and buy drugs at the pharmacies when they fell sick.

**F:** In terms of the distance to the hospital, how far is the hospital from this community?

**00**: (All) eeeeeh its very far.

**F:** One by one please, you are so quit this side are we together (Laugh)?

**00**: (All) Laugh. We are tired.

**F:** Are you tired, we are just starting our discussion (Laugh).

**00**: (Laugh)

**F:** Please raise your voice as you are taking.

**P 1:** The clinic is very far from this area and sometimes it is very disappointing to travel long distance to the hospital only not to be given appropriate treatment as such most of the people are discouraged to go to the hospital. Sometimes we are just not willing to go to the hospital when we fell sick but to me it is good to go to the hospital when one is sick because after the diagnosis at the hospital that's when you know about the appropriate drugs that you can buy at the pharmacies than just going straight to the pharmacies to buy drugs without knowing the real problem as well as proper dosage. Sometimes we us parents, we fail to explain the real problem that is in our children and in most cases, we usually provide drugs to our children before seeking advice from the doctors.

**F:** Mmmm, what else. We were discussing about long distance to the hospital.

**00:** (All) Yes.

**F:** What about in terms of the money that you spend when travelling to the hospital, how much do you use as money for transport to travel to the hospital?

**P 7:** 1000.

**P 1:** The motorcycle taxi operators charge K 1,500.00 one way.

**F:** One way?

**00:** (All) Yes.

**00:** Go and Back its K 3,000

**F:** If you compare that with buying medicine at the pharmacy, what is more costly between buying drugs at the pharmacy and going to the hospital?

**00:** It just depends with the distance from your house to the hospital since some stay close to the clinic.

**F:** But just comparing the amount of the money that you spend when travelling to the hospital and buying drugs at the pharmacy, where do you spend most?

**P 1:** Most of the private hospitals now requires that one should have a health passport before being assisted and in the pharmacies, they also demand the health passport so that they should know the drugs that the doctors prescribed for one to buy. In the past most of the pharmacies were just allowing people to buy drugs in small amount but as of now they require one to buy full or half dose. For instance, one can be having flu and at the pharmacy they require him/her to buy a full dose of Erythromycin at K 4,000 or the full dose of Panadol at K 1, 500 and as of now Amoxicillin for children is at K 2,200 but they usually allow you to buy the drugs when you have the health passport.

**F:** Why do you think the pharmacies are now demanding the health passport so that they can sell one medicine that are prescribed by the doctors here in Mpingwe?

**00:** They are afraid of selling drugs that are contrary to the disease that one is suffering from.

**00:** It is very cheap to go and assisted at the public hospital but when one goes to the hospital, he/she is recommended to buy the drugs at the pharmacy.

**F:** How many there pharmacies here in Mpingwe who does not sell drugs to the people without prescription from the doctors?

**00**: Only few but most of them just sell drugs freely.

**00:** Most of them just sell drugs because they are after making money and they does not prioritize the health of the people.

**F:** How do people react when they are told that they won't buy drugs when they decide to go straight to the pharmacies? Let’s explain what is happening here.

**00:** (All) Silent.

**F:** What would be your reaction if the government would come up with a provision that those who do not get prescription from the doctors should not buy drugs at the pharmacies.

**00:** It can be very painful.

**F:** How painful can this be?

**00:** It can be very painful because we usually go to the pharmacies to get assisted so going to the pharmacies and without getting any assistance at the pharmacies as result of not getting prescription from the doctors that would be painful because in most cases people do not get proper assistance at public hospitals.

**F:** You mentioned about vaccines for body sores, how do you know about the type of drugs to buy at the pharmacy?

**00:** We just look the signs and symptoms and from there we make a decision to buy such and such drugs.

**F:** What signs do you observe for you to come up with a decision to buy Amoxicillin?

**00:** When the child has flu and cough, that’s when we decide to buy Amoxicillin.

**F:** What about Erythromycin, what signs and symptoms do you observe for you to come up with a decision to buying this drug?

**00:** When we usually go to the hospital with sore throat, they mostly give us or tell us to buy at Erythromycin, the pharmacy. As such, when one has sore throat, he/she can go straight to buy at Erythromycin at the pharmacy.

**F:** Now you are telling me that people from Mpingwe when they are not feeling well, they usually go to buy drugs at the pharmacy when they decide not to go to the hospital? There is nothing else that people do?

**00:** Some go and seek assistance from the witchdoctors and we have other people who are members of Apostolic church who does not go to the hospital and some believe in witchdoctors so they usually go to get assistance from these people. For instance, some associate diseases like headache with witchcraft so they usually go to the witchdoctors to seek for assistance when they experience that.

**F:** You have mentioned about Apostolic church, that they have their own ways dealing with diseases, right?

**00:** Yes, they have their own ways since they usually don't go to the hospitals when they fell sick. They just follow what they are taught at their church that whenever they are not feeling well, they should just drink a lot of water as such they don't go to the hospital and some of them usually go to the witchdoctors.

**F:** How do you come up with a decision to buy a particular drug? Of course you have mentioned that you usually make such decisions based after the signs and symptoms, so what else do you look for?

**P 3:** When I go to the hospital with a child who has sores, they usually told us to buy Bactrim and when there are no changes after taking Bactrim, they usually recommend that you should buy Amoxicillin and when that does not work, they also told you to try Bactrim again and whenever I see that my child has cough, I usually go and buy Bactrim at the pharmacy.

**F:** Let me hear from others?

**00:** My child had sores that were itching and I went to buy drugs at the pharmacy but to no avail and then I went to the hospital but there were no changes so I just decided to leave it as it was and the problem was dealt with on its own. At the hospital the child was given an injection but didn’t work.

**F:** Are we aware of the drugs known as anti-biotics?

**00:** (All) Silent.

**F:** Are we aware of these drugs?

**00:** We have heard about those drugs before that they are used to cure skin sores.

**F:** Out of the drugs that you have mentioned are there any anti-biotics?

**00:** Bactrim.

**F**: No, I just want to learn from you, out of the drugs that you have mentioned, are there any anti-biotics?

**00:** Yes, there are.

**F:** What are they?

**00:** Bactrim.

**F:** Let me hear from others or we don’t know?

**00:** We don't know.

**F:** Alright, let us proceed, we were taking about what people usually do when they are not feeling well in general. Now let us talk much about children, when a child start experiencing Diarrhea what do you do first before going to the pharmacy or hospital, I just want to know what you do at home?

**P 4:** Aaaah,

**F:** Sorry, please raise your voice?

**P 4:** When we are at home and the child is having Diarrhea, we give him/her solution made up of sugar and salt locally known as *'Thanzi'*

**F:** What about others, what do you do?

**P 1:** When a child is having Diarrhea especially during the night, most of the women are reluctant to do anything until the next day for them to go to the hospital and this makes the condition of the child to become worse but I believe that when the child is having Diarrhea is better to take him/her to the hospital while we are giving him/her solution made up of water and salt on the way. At the hospitals, women who brought children suffering from Diarrhea are not allowed to be on the queue, they just went straight to meet the doctor. But in most cases, most of the women wait until the condition of the child become worse for them to go to the hospital.

**F:** She has explained what we are supposed to do.

**00:** (All) Yes.

**F**: But I would like to know what usually happens, you should remember that we have mentioned that most of the times we are deliberately not interested to go to the hospital due to the fact that there are no drugs at the hospitals and that they do not welcome you in a good way. What do we usually do at home? I know we cannot always have money to buy the drugs when the child got sick.

**00:** Yes.

**F:** Now in a situation where you don’t have money and considering the long distance to travel to the hospital where there are no drugs, what do we do on our own apart from making salt and sugar solution

**00**: We usually prepare soft porridge.

**F:** Anyone here whose child have had Diarrhea before?

**00:** All of us.

**F:** Everybody?

**00:** Yes.

**F:** Now I will ask everyone to tell me what they did or usually do (Laugh)

**P 6:** In a situation where the clinic was very far and the condition of the child was worse, I just bought rice and prepared a soft porridge for the child and when I did that I observed that the condition of my child for some time and when I was that it was not improving, that's when I decided to go to the hospital. At the hospital I was asked what I did to the child before going there and I told them that I gave the child soft porridge of rice and they said I did well.

**F:** That's number 6, now each one of us is going to tell us what they did, P 10?

**P 10:** Some say that......

**F**: What you did.

**P 10:** When my child had Diarrhea, I was told to soak tea into warm water and give it to the child to drink.

**F:** Where did you learn about this?

**P 10:** From my friend.

**F:** What happened when you gave that to your child?

**P 10:** His/her condition improved and I didn’t take the child to the hospital

**F:** Alright, let me hear from others? P 9.

**P 9:** I prepare a solution of salt and sugar and give it to my children.

**F:** Is that all?

**P 9:** To date whenever my child has Diarrhea, I just make a solution of salt and sugar.

**00:** I was told to make a paste of guava leaves and I mixed it with waste and after that I gave it to my child who was suffering from Diarrhea to drink.

**F:** A paste and solution of Guava leaves?

**P 9:** Yes.

**F:** P 2?

**P 2**: I usually make a soft porridge and buy Doxycycline.

**F:** Why do you give your child Doxycycline after giving him/her soft porridge? what else to you add to the porridge?

**P 5:** We usually add some salt and sugar.

**F:** It is made from Ngaiwa flour?

**P 5:** Yes.

**00:** I have already said that I usually give my children soft porridge.

**F:** P 2 what do you do?

**P 2:** I usually make a solution made up of salt and sugar.

**F:** You don't do anything apart from that?

**P 2**: Yes.

**F:** Lets go back to what we discussed earlier about factors that increases the risk of Diarrhea but now let's focus on children, you have mentioned about unclean water and this applies to the whole community. Now I just want to know things at household level that increases cases of Diarrhea amongst the children?

**P 3:** Unhygienic practices at household levels increases cases of Diarrhea.

**F:** Can you explain clearly what you mean by unhygienic practices?

**P 3:** Maybe let's say after changing the baby diaper, it is recommended for one to wash his/her hands with soap but some women do not do that, they just go straight to breast feed their children without washing their hands after changing the baby diaper. In addition to this, some women do not wash their hands after visiting the toilet before feeding their children.

**F:** P 4 it looks like you have something to say.

**P 4:** I wanted to mentioned what P 3 has just said.

**F:** In your opinion, what do you think are the main contributing factors of Diarrhea, especially at household level?

**P 1:** Most of the women do not give foods that are recommended for children who are young, for instance some women guy *'Thobwa'* that is sold along the roads and give it to their children, some guys some snacks such as *'Zibwente*' along the roads that are not well covered and these exposes children to Diarrhea.

**F:** I want to hear from others, please let's all participate so that we can finish in good time. What other factors do you think contributes to more cases of Diarrhea among the children from this community?

**P 7:** When a child starts to craw, we (women) just left him/her to eat anything that he/she come across with since we believe that young children are fond of eating anything.

**F:** Alright thanks, you have explained about factors that contributes to more cases of Diarrhea in this community, what about strategies that people use to prevent Diarrhea among both the children and the adults?

**P 1**: I believe that it is difficult to prevent Diarrhea when it comes to children.

**F:** Let's talk consider these as a separate topic, let us look at it from home and community setting. I would like to know what you do at your household and what people from your community does to prevent Diarrhea? Let's start with strategies at household level, you have said that it is difficult to prevent Diarrhea among the children, why?

**P 1:** In most cases when a child is having Diarrhea based on our tradition, we believe that the child is developing the teeth, I can say that we associate Diarrhea with teeth development in children and these prevents some women from seeking the remedies for Diarrhea at the hospital. According to my observation, as of now cases of Diarrhea has been reduced unlike it was in the past when a day couldn't elapse without hearing that someone has died of Diarrhea.

**F:** Let me hear views from others on this issue that cases of Diarrhea are not common these days.

**00:** Yes, it is true that cases of Diarrhea are not common as they used to be in the past.

**00:** It is true due to the fact that when we went to the hospital, we are given some tips on how we can prevent Diarrhea and due to these cases of Diarrhea has been reduced.

**F:** What advises do you get from the hospital that helps in preventing Diarrhea?

**00:** We are usually told to monitor our children so that they should not eat anything they come across with when they are moving around and this helped in reducing cases of Diarrhea.

**P 3**: I can say that cases of Diarrhea has been reduced due to the fact that most of the children are now getting Rota virus vaccine. Since children started receiving this vaccine, most of them are not suffering from Diarrhea anyhow.

**F:** Apart from following advices from the doctors and Rota vaccine, what else do you think has helped in reducing cases of Diarrhea?

**P 1:** I would say that most people now are practicing hygiene because there are HSAs who move around the communities and sensitize people on things to do concerning hygiene which was not the case in the past and due to the Rota vaccine study, most of the children get vaccinated now.

**F:** This side you have been quiet, our friends have said that cases of Diarrhea among the children are not common as they used to be in the past and they have mentioned some things that has helped in reducing these problems. Are there other things that has contributed to these?

**00:** (All) Silent.

F: Alright let us proceed, some have mentioned about Rota virus vaccine which they say has contributed a lot in reducing cases of Diarrhea. Now I just want us to discuss about this vaccine, firstly I would like to know what people from this community say about this vaccine?

**P 1:** At Bangwe health center there was a study for Rota virus vaccine in 2020 if am not mistaken, when we were attending antenatal clinic, we were told about this study and the vaccine, some people were interested to join the study and others not some were withdrawing from the study after they were enrolled. For those who participated in the study, they benefited a lot not due to the fact that they were getting money but due to the fact that their children are no longer suffering from Diarrhea after being vaccinated. Some people were discouraging their friends from joining the study as they associated the study with things to do with underwater powers.

**F:** We are discussing about what people in the community say about the vaccine, right?

**00:** (All) Yes.

**F:** Our friend has talked about advantages of the vaccine as well as other things that people say about the vaccine, what else do people say about this vaccine?

**00:** Some people say the vaccine is associated with satanism.

**F:** What?

**00:** Some people say that the vaccine is associated with satanism.

**F:** Can you elaborate more?

**00:** Some people say that it is associated with satanism because a blood sample was being collected during the time when children were getting the vaccine and some people do not have a better understanding of the vaccines including this one.

**F:** Alright, let's focus on what people say about the vaccine? You have said that people say that the vaccine is not good.

**00:** Yes.

**F:** What problems do people experience with the vaccine?

**00:** Let me speak, on my side I have a neighbor who is a member of Apostolic church and she usually say that based on the scriptures, the introduction Cholera or Polio vaccine is a sign of end times and at the antenatal clinic most of the women usually say that this vaccine is associated with satanism and this prevented a lot of women from participating in the study.

**F:** Are there other factors that prevents people from getting Rota virus vaccine that you are aware of? before we look at factors that encourages people to get the vaccine because regardless of factors that prevent people from getting the vaccine, there are also other factors that persuade people to get the vaccine.

**00:** A lot people say that this is associated with satanism.

**F:** Let me just ask, is there anything bad that happened to someone here in Mpingwe as a result of participating in a study or allowing his/her child to get vaccinated?

**00:** (All) nothing at all.

**00:** One day as I was going to the clinic, I met a woman who was coming out of the room where the study was being conducted and after some few days after participating in the study, she lost her child and some people were saying that she sacrificed her child by participating in the study.

**F:** Did this happened when the child got vaccinated, can you explain to me clearly?

**00:** After getting the vaccine that was being provided to the children as part of the study, the child got sick and buy the time the woman was going with the child to the hospital it was too late and people started speculating that this vaccine is associated with satanism.

**F:** What else bad happened to someone else as a result of participating in any study?

**00:** Thats all.

**F:** Now let us look at the factors that encourages people to get vaccinated, of course you have said that some people were refusing to get their children vaccinated while others were getting the vaccine. What factors do you think encouraged most of the people to get the vaccine?

**00:** It was due to the fact that the when children get vaccinated, they were not suffering from Diarrhea.

**00:** It was also due to the fact that whenever a child who got vaccinated fell sick, we were not going to the general section at the clinic but we were going to the section where the study was being conducted for assistance and there, people were getting adequate assistance and advise as compared that that is provided at the general section of the hospital.

**F:** What advice were you getting when you were Rota virus vaccine.

**P 1**: I was one of the people whose children got Rota virus and my child was fond of suffering from flu and one of the doctors who was conducting the study asked me about the baby powder that I was using and when I told him he explained that most of the powders causes infection in babies hence he advised me on appropriate powders that I should be using for my baby not to have flu and we were learning a lot about personal hygiene as well as food that is recommended for babies.

**F:** Some mentioned that this vaccine is also being administered by the government, right?

**00:** Yes.

**F:** What do you think are the factors that are encouraging people to get Rota virus vaccine that is being administered by the government?

**00:** It’s because of the fact that cases of Diarrhea among the children have been reduced as a result of this vaccine.

**P 1:** We are encouraged to get our children vaccinated because people conducting the study treat us with respect and they give us proper help unlike the nurses who work under government at the clinic.

**F**: What about the vaccine that is being administered by the government, what do you think are the factors that are encouraging people to get Rota virus vaccine that is being administered by the government

**P 1:** It’s because of the fact that cases of Diarrhea among the children have been reduced unlike in the past.

**F:** What about distance, how does distance affect people's ability to get the vaccine?

**P 1:** When we went to meet the research staff, they were offering us money for transport and they were giving us some drugs that were not available at the government clinic.

**F:** Are children still getting Rota virus vaccine?

**P 1:** Yes, in the government clinic people are still getting the vaccine.

**F:** In what way does distance affect the accessibility of Rota virus vaccine?

**00:** This vaccine is very good and it is only available at the public clinic unlike other vaccines.

**F:** This vaccine is only found at the clinic?

**00:** Yes.

**F:** Is Rota virus vaccine administered to children orally or through injection?

**00:** It is administered orally.

**00:** Sometimes we fail to go to the hospital because of factors such as distance but when it comes to getting our children vaccinated, we try our best to go to the hospital

**00:** Yes.

**F:** Why do you always make sure that you should not avoid getting your children vaccinated?

**00:** Vaccine for children is mandatory and that each and every child is supposed to get vaccines that were recommended by the government that they should be administered to children.

**F:** Is there any woman whose child missed some doses of vaccine here?

**00:** Yes.

**F:** Can you tell me what happened for that to happen?

**00:** It was due to the fact that I was not willing to go to the hospital due to long distance and lack of money for transport.

**F:** Is this the same case with Rota vaccine or any other vaccine?

**00:** Any other vaccine.

**F:** What factors do you think can encourage women to go and get their children vaccinated at the hospital?

**P 1:** May if the vaccine is administered at household level by the HSAs

**F:** What else? what role to men play in ensuring that children get vaccinated?

**P 1:** Most men do not play any role when it comes to children vaccine.

**F:** What factors prevents men from playing any role in ensuring that their children get vaccinated?

**00:** Most of the men are shy and do not feel comfortable to go to the hospital with their children or wives to the hospital.

**00:** Most of the men are not concerned about the wellbeing of their children as they are usually engaged in income generating activities.

**F:** What do you think should be done for men to play a role in ensuring that their children are vaccinated?

**00:** Some men do not like the vaccine and are usually violent to their wives when they heard that their children get vaccinated.

**F:** Do you think unwillingness of the men to take part in making sure that their children get vaccinated affects the health of the children?

**00:** Yes, because sometimes a woman may fell sick and due to this the child can miss the dosage of the vaccine as most of the men are not willing to take their children to the hospital as that is regarded as one of the roles for women.

**F:** What do you think should be done for men to play a role in ensuring that their children are vaccinated?

**00:** There is a need to conduct civic awareness campaigns with the men aimed at sensitizing them about their role in as far as growth and development of the children is concerned.

**F:** What kind specific topics should be shared to men in such campaigns?

**00**: They should take proper care of children.

**00:** They should be working in hand with their wives in any matters concerned health of their children.

**F:** We are approaching the end of our discussion and I feel like we have discussed a lot that I wanted to hear from you, now in general I just want to hear from you what you think can help in encouraging people to ensure that they should make sure that their children get vaccinated in the communities?

**P 1:** Us women we should not get carried away by rumors that people spread in the communities about the vaccines especially those that are spread by those who don't believe in seeking health services at the hospital.

**F:** How can these messages be spread to other women who are not present here that they should not get carried away by the rumors?

**P 1:** Maybe telling the women about this when they are attending antenatal clinic.

**00:** Maybe us (women) who are present here we can carry this message to other women in their respective communities.

**00:** Maybe the health care workers should be moving around the communities telling people about this.

**00:** It would be better for the health care workers to spread these messages because most of the people trust them as compared to other people.

**00:** Some people believe that the vaccines are responsible for causing impotence in people at a later stage.

**00:** Most of the people are willing to get vaccinated when they experience a problem

**F:** Meaning that people require specific evidence for them to get vaccinated?

**00:** Yes, I was one of the people who are afraid of getting Cholera vaccine but when I saw someone suffering from Cholera, I decided to get the vaccinated and when I told my husband he was motivated to get vaccinated too because each one of us is afraid of death

**F:** What should be done for people to be willing to get vaccines without any evidence?

**00**: It would better if the health care workers to move and spread the messages about the goodness of the vaccines in the communities.

**00:** These messages can be spread through radios.

**F:** Alright, thank you so much. I have learnt a lot of things. Now I would like to give an opportunity to each one of us to talk about anything they wanted to talk but I was not able to ask on that, P 2.

**P 2:** My last word is that there is a need for the health care workers to move around the communities and encourage us in any issues concerning health because this can enable us to share such kind of information with our friends, thank.

**F:** Thank you, anyone else who want to talk, sorry if I mention you it does not mean that there is any other reason but I just want all of us to be active.

**00:** I have a question, for us to meet again and have the same discussion, are you going to call us again?

**F:** What we have discussed today was scheduled for today only but in a situation where we did not, capture some useful information, we usually make phone calls to discuss that with you or we can approach one person from this group. This does not mean that there is something else with that person but we just want to get some information from that person that was not capture well during a discussion like this. So, our discussion is over today but if any need arise that we should meet and discuss, we are going to call you. Anyone else who want to talk.

**00**: My last word is that I just want to ask the health care workers I remember in the past they were checking in the health passport for children if they come to the antenatal clinic on regular basis. There are instances where people do not visit antenatal clinic once the child is born and the health care workers do not follow up on that unlike in the past and this was encouraging people to take their children to antenatal clinic on regular basis.

**F:** May the health care workers are afraid of being insulted by the parents (Laugh)

**00**: (All) Laugh.

**F:** Alright that's noted that’s a good point. Anyone with a comment?

**00:** (All) No.

F: Alright, ladies am really grateful and we had a nice discussion, thank you very much.
