# Supplementary material for: Exploration of the social determinants of diarrhoea, rotavirus vaccine uptake, and vaccine ‘fatigue’ in Ethiopia, Kenya, and Malawi
Source: PLoS One. 2025 Sep 9;20(9):e0319691. doi: 10.1371/journal.pone.0319691 (PMC12419581; doi:10.1371/journal.pone.0319691)
Supplement: S1 Data — (ZIP) [file pone.0319691.s001.zip › Supporting Information Files/KY_05FGD.docx]

**FOCUS GROUP DISCUSSION 5**


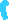


**NUMBER OF RESPONDENTS- 10 ( 8 FEMALES, 2 MALES )**

**1.Can you please tell us some of the illnesses that affect children in your community?**

R1- Diarrhea

R 2- Malaria
R 3-Common flu, coughing and diarrhea.
R 4- Chicken Pox
R 5- skin diseases

**2. Which of these illnesses do you consider to be a burden in this community? Why do you say**

**so?**

R1, it is diarrhea because it happens every day, people have died because of it

R 2- Mostly diarrhea is a big problem, we can not control or prevent it.

R3- Common cold is another one, it keeps on recurring. Almost every child in the plots suffer from it at the same time.

R 4-For me it is diarrhea, if one child gets everyone in the house start diarrhea, it is costly to treat the whole family.

R 5 Measles can make a child not to sleep, if the child doesn’t sleep, you will not sleep too.

R6- if your child gets sick, mentally you are affected too, for me diarrhea and flu is the biggest problem

**3. How do most people respond when a child has diarrhoea in the home?**

R 3- I give the child a slice of bread without any fluid, the stool will harden eventually
R 4 -Pawpaw and yoghurt can help stop diarrhea, that is what I was told by mum
R 5-I buy some ORS from the chemist and give it my child

R 6 - In our community we mostly believe that diarrhoea is caused by plastic teeth in children. When they get diarrhoea, we take them to a man in our community who rubs the teeth of the child, and it stops

**4. Can you tell me some of the enablers and challenges that people experience to access treatment for diarrhea diseases?**

1. **Challenges**

**R1.** The first obstacle is our environment, reinfection will keep on occurring, It would be like good if we organize for proper cleaning of the places. Because the disease is still there with us. Even if you take him to the doctor, he will be okay for two days but will diarrhea again. It would be good if we could organize, even if it's a toilet, I hear that there is a round substance that can be used in the toilet to kill germs.

**R2.** when you go to the hospital, you get a queue. You can go to a hospital like Njenga, you are told there is no medicine, you go and get the medicine. Even people have started to do trial and error with drugs. If I give him this tradition medicine or Maasai medicine can it treat this. We go ahead and ask tradition healers, what medicine can give my child. These tradition healers are not educated in this concoction. They normally just guess roots and we give them to our kid because at the hospital we are not treated the way we are supposed to.

**R3.** There is a long queue. When you reach to the hospital you are told to wait for the doctor and that day you might not have a chance to see the doctor. Then you are given an appointment of a different day and the illness is getting worse day by day.

**At community level**

R1. The one who have NHIF card, at least, he can help the child. Now, you get someone who has no job or business, he doesn't even have money for food, how will that person take the child to the hospital. He might even don’t have fare to take the child to the hospital. So, he keeps on guessing drugs and painkillers in the house. After some time, you hear that the kid died in the house or on the way to hospital.

**How do you prevent childhood diarrhea at household level?**

**R1. I** observe cleanliness like if I clean my child, before eating I ensure proper handwashing. Boiling of drinking water or medicine that kills germs.

**R2.** We should observe cleanliness and minding the food that we eat. Proper cleaning of plates and cups. For children we ensure we maintain short nails.

**R3.** For me, in my house we try to avoid roadside food vending because it can be contaminated with waste water and houseflies.

**R4**. We should observe cleanliness so much especially when you buy fruits should be cleaned and handwashing of children. Mostly diarrhea is brought about by dirt. In my location food is prepared near waste water drainage and houseflies come from there and contaminate the food. Personal cleanliness is very important to prevent diarrhea

**R5.** I can say even giving children one type of food can cause diarrhea, but we should uphold cleanliness and try to change food type.

**R6.** We can remove dirt from our houses by cleaning utensils, covering of food, baby’s clothes, proper disposal of pampers and getting rid of all flies in the house

**What are the preventive measures at community level**

**R1.** At our residential plot we have a waste collecting sacks that are disposed daily. We also have a cleaning plan around the waste collecting sack. When we do laundry we clean around the toilet and bathroom properly even if a child comes to play they are safe to. We do regular cleaning of drainage, we do get all the waste from the drainage dry them and dispose them to allow proper flow of water. A child can fall in the drainage and when its blocked the child will carry the dirt and contaminate everywhere.

**R2.** Where I live is a bad place, people don’t dispose waste properly instead of putting waste in the waste collection sack they dispose them on the walking pathway.

**R3**. I can say mostly diseases like cholera are caused by fecal material. When you touch it causes diarrhea. For instance, some come with their fecal waste in a polythene bag and throw away not minding where it will fall. Especially when it’s raining, it is not advisable to go out of the house. It can land on you. You will never know where it comes from. So, I suggest we improve on things like toilets. We can have pit latrine or toilets or anything that is safe.

**How do people in this community perceive childhood vaccines**

**R1.** There is a church here where children get sick and they are not given any medication, Even vaccine, there are others who have refused it. Just because the pastor said… the pastor said, if you get polio vaccine, don't come,

**R2**. These vaccines are too much, many people say they don't like polio vaccine because they give it out every time

**R3**. Obviously before vaccines for children are brought research has been done on them. This is round one of Polio vaccine and we have round two this month. And after this month there is round three they said. If a child misses one of these vaccinations and something happens to your child, you will blame yourself. So, we as parents, if doctors and government have approved the vaccines, we need to allow our children to get vaccinated. Like corona vaccine people were saying that we are used in testing the vaccine to see how many dies. If your day has reached, that is it. We need to get full responsibilities of our own children.

**R5.** These vaccines are not bad for instance vaccine for Polio before it starts there are people who announce and create awareness. We should ensure a child gets all the doses required.

1. **why do you think childhood vaccines are widely resisted?**

**R1**. Majority rejects vaccination.

**Who rejects the vaccines the most between male and female?**

**R1.** Most of people who reject vaccination are male as they say my friend’s child got vaccinated and his limbs rotten. They normally use friendship. Husband mostly say children are for mother especially if there is a problem. Husband rejects vaccination in most cases.

**What is the Impact of COVID-19 on childhood vaccination**

**R1.** They reduced the number of patients being vaccinated. For instance, we used to go 30 patients per day but during the pandemic they reduced 15 morning and others 15 afternoon not as we used to go all us once. They were dividing by two, mornings and afternoon.

**R2**. There were those children that were taken to hospital for weight measurements. The doctor refused to measure their weight, they only allowed those getting the vaccination.

**R3**. Some people were afraid of the vaccines so they didn’t take their kids to clinics. They used to say when they get vaccinated, they get sick and some weak. So many children during the pandemic did not receive their scheduled vaccines since it was mandatory to get vaccinated to get into the healthcare facilities.

**R4.** Majority of the children did not receive vaccination.

**R5**. Some said this vaccination affects brain and others said it brings death

**What makes majority of people accept clinic vaccination**

**R1.** You know when children are still young every parent wishes all the best but when they get a little older. Mothers start becoming ignorant but when they are young, they follow up each vaccine they require for their upbringing.

**R2.** Once they are 1 to 2 years old, they start ignoring clinics. They assume are out of danger zone. Once they are done with the first 3 vaccinations they are done. They even forget about the 1.5year injection. If you do research from out of 100 children 20 children didn’t get 1.5 injection.
