# Supplementary material for: Exploration of the social determinants of diarrhoea, rotavirus vaccine uptake, and vaccine ‘fatigue’ in Ethiopia, Kenya, and Malawi
Source: PLoS One. 2025 Sep 9;20(9):e0319691. doi: 10.1371/journal.pone.0319691 (PMC12419581; doi:10.1371/journal.pone.0319691)
Supplement: S1 Data — (ZIP) [file pone.0319691.s001.zip › Supporting Information Files/ET_6FGD.docx]

I: Thank you for your participation. What are the most common diseases in children under the age of five?

P1: Thank you, it is usually pneumonia, flu and vomiting.

P2: Fever and vomiting are also common here.

I: are the causes for those symptoms?

P2: I don’t know.

I: Please, participate.

P3: Diseases caused by bad smell.

I: Can you give me examples?

P4: Diarrhea and tonsillitis are other common diseases.

P5: Fever and having interrupted sleep. In addition to that, fever, drying of lips and daydreaming are some common diseases.

I: From the diseases you have listed, which disease is more dangerous?

P6: I think it is pneumonia and it is caused by the absence of environmental hygiene. Cherkos, the village we live in, is a village with a common latrine for many households. For example, I have twin children and they are often affected by pneumonia. I usually complain about the toilets, but no one can hear me.

I: You have listed diseases like pneumonia, diarrhea, flu and other diseases. Which of this disease is more dangerous?

P7: In our village, there is a flu. My daughter is usually affected by flu or tonsillitis. They also say it is pneumonia.

P2: It is pneumonia, the coughing usually changes to pneumonia.

I: Which one is the most dangerous?

P4: It is flu.

P1: Diarrhea and vomiting are the most common in my child’s case. It is because he is taking in the dirt, even if they give us medicines, that don’t help cure the disease. It is better nowadays, there is better sewage disposal now and they pick up trashes two days per week.

I: Good. You have listed flu, diarrhea and pneumonia as the most common diseases. Would you rank them in order of their dangerousness?

P2: It is pneumonia first, diarrhea and the tonsil in order.

P3: We are in the same village.

P5: As she said, tonsillitis couldn’t have gotten a cure.

I: Can you rank the diseases in order?

P5: Tonsillitis and diarrhea, respectively.

I: Any other?

P1: It is pneumonia and then infection from wounds on the head of our daughters.

I: Any other?

All participants: *[silence*]

I: What health facilities are there here, facilities you get health services from?

P1: There are health centers, private clinics and traditional health facilities.

I: Okay, any other?

P2: Some workers provide a home to home health services. There is Cherkasy Health center we use the services from.

P7: We usually bring our children to health center and go to private clinics if they don’t get better.

I: Any other.

P4: I bring my family to police hospital as my husband is a staff there.

I: Any other?

P6: People use governmental health facilities or private clinics in search of more available medicine. We can’t get medicines available in governmental facilities as we only use health insurance.

I: How much do you spend for the services in the health center?

P2: We pay 30 birr for the registry card and we pay different for different medicines and laboratory services. We at least spend around 2,000 Birr.

I: Any other, how about the payments in private clinics?

P3: There are private clinics called Amaresa and Birhan. Those are found around Bulgaria. We spend based on the disease, we may spend 2,000 or 3,000. It is hard to tell the prices.

I: How about you, how much do you pay for health services at the Police Hospital?

P4: It is free for us.

I: How much do you have to travel to reach the health facilities?

P6: It takes 20 minutes to reach the health center.

P5: It takes me around 30 minutes.

I: Has the distance of the facility from your residence been an obstacle to using the health services?

P7: Yes.

I: What do you do if you don’t want to travel that, what option do you take?

P7: Noting, we must do that.

P4: It takes me 40 minutes for me.

I: So, has that 40-minute walk become an obstacle to getting the health service?

P4: No.

I: What do you do at home to treat your children before taking them to the hospital for treating diarrhea?

P2: I pour salt into hot water.

I: Any other?

P1: I take him to the hospital and give him more liquid if it happens during the day. If it is at night time and I can’t get him to hospital, I give him a lemonade.

I: Any other?

P5: I only take them to the hospital.

I: Any other?

P2: We also treat them with Tenadam and water solution.

I: What results do you observe after these treatments at home?

P2: They are curing sometimes, and don’t give a cure sometimes. If they don’t, we go to health centers.

P4: We just use these treatments as a temporary solutions. We might put a towel on head of patients, it doesn’t mean that can cure.

I: So, you are saying that you must go to health facilities anyways?

All participants: Yes.

I: Okay, you have told me what you do. Now, tell me what the people in your community do at home to treat diarrhea? Haven’t you ever purchased medicine without out prescription?

P2: We only buy medicine with a prescription.

I: What are the challenges that made you not use health services in health facilities?

P2: Shortage of money.

I: Please, elaborate on that.

P2: We need money to purchase medicines.

I: How about the opportunities that made you use the health services?

P2: The health facilities are all nearby, this is one good thing.

P5: The health insurance system provides suitable situations for people with no money.

P5: Most of the time the medicine may adapt to the diseases and may not give us the cure. So, unless it is severe we shouldn’t use the medicine.

I: In your opinion, what are the major causes of diarrheal disease?

P6: Not washing hands before having meals.

P7: The changing weather can also be the cause.

P5: It is because of poor personal hygiene.

I: Any other?

P3: Temperature variance can also affect children's health.

I: What do people do at home to prevent diarrhea?

P1: We keep their hygiene, making sure they don’t take in their hands to their mouth before washing.

P4: We should also clean our environment.

I: What methods are you using at home?

P4: We only have to feed our children fresh food to avoid germs.

I: How about the methods that have been applied at the community level?

P5: We have to bring our children to health facilities and check for their health.

P1: We clean our environment every Saturday in groups.

I: Do the workers collect the trash bags you accumulate?

P1: Yes, they come twice a week to collect.

P3: The Edir organization are responsible for the supervision of the cleaning programs with Wereda leaders. Everyone must keep cleaning their environment within 50 meter radius from their house.

I: What is the community's perception of vaccination?

P1: It depends on the people. Some people properly take their vaccination services. Some skip some vaccinations with ignorance.

P5: Some people think children are only people below the age of nine months. Some mothers may forget vaccination schedules as they are usually busy. The extension workers usually get mad.

P2: I get the vaccination service every three months.

I: What is the purpose of the vaccination for you?

P2: It prevents from diseases, from diseases like polio.

I: Is there any other idea?

P7: Most people in Addis Ababa get the service for their children under the age of five, and even the people in the country do the same.

I: Do you think vaccination is well accepted by the community? Or it is just because it is mandatory?

P3: I think it is because it is Mandatory.

P2: I don’t agree with that, it is because it is useful.

I: Have you ever heard of a vaccine called ‘Rota Virus’? Let me give you a hint. It is the vaccine in form of droplet, in three rounds. What is the perception of the people regarding this vaccine? You may tell me what the people know about its uses, or things like that.

P2: I hear from people that the vaccine in the form of droplets is useful for measles. That is all I know.

I: Any other?

P4: I don’t think the people know about that vaccine?

I: The people have been taking that, though.

P4: You are right, but most of them don’t know that. They don’t specifically know the nama e, they might know the purpose.

I: What do they know about the uses?

P2: They know it prevents diseases.

P4: It prevents from osteoporosis.

I: Any other.

All participants: *[Silence*]

I: What triggers people to get the vaccine services for the rotavirus?

P1: It is because it is necessary for their health. They should teach us which vaccine is what other than just telling us to come on the schedule.

I: Where did you get the service from?

P1: From health center.

I: How about in campaigns done home to home?

P1: They do that for measles vaccination.

I: Is the health center close for all of you?

P1: Yes.

I: Is there any challenge from religious thought, or cultural barrier for the vaccination?

P5: I have never heard of anything about that in religious thoughts. It has never been a challenge.

I: Was the COVID-19 pandemic situation an obstacle to the vaccination?

All participants: No, it wasn’t.

I: Have you ever been concerned about the safety of the vaccine?

P5: I have a year and eight months and a two year and six month children. They are both healthy, so I think they are safe.

I: How about the perception of the people in your community?

All participants: *[silence]*

I: Thank you, I am done asking. You can add if there is anything you want to add.

P5: I want the health extension workers keep up doing what they are doing on this matters.

I: Okay, thank you.
