# Supplementary material for: Exploration of the social determinants of diarrhoea, rotavirus vaccine uptake, and vaccine ‘fatigue’ in Ethiopia, Kenya, and Malawi
Source: PLoS One. 2025 Sep 9;20(9):e0319691. doi: 10.1371/journal.pone.0319691 (PMC12419581; doi:10.1371/journal.pone.0319691)
Supplement: S1 Data — (ZIP) [file pone.0319691.s001.zip › Supporting Information Files/KY_07FGD.docx]

**FOCUS GROUP DISCUSSION 7**

**13/03/2024**

**NUMBER OF RESPONDENTS-10(5 MALES,5 FEMALES)**

**1. Can you please tell us some of the illnesses that affect children in your community?**

**R1-**Diarrhoea, Sneezing and common flu, white patches on the body that we can refer to as a skin disease.

**R2-**Cholera, measles and diarrhoea during teething in babies.

**2. Which of these illnesses do you consider to be a burden in this community? Why do you say**

**so?**

**R4-**Another burdensome illness that affects children in this community is common flu especially during the morning and evening hours. You might even find a child facing breathing difficulties because of this. Eating becomes difficult for them and their fever rises and it is during this time when we rush the child to the hospital.

**R5-**Another illness that affects children in our community especially from Ruben is respiratory diseases. This is because there are many factories close to us and the smoke from these factories affects children causing respiratory complications. It affects many children and you may find these children losing weight. This is a challenge to many parents especially those living next to those companies.

**R3**-To add on what my colleague has said, there is a lot of environmental heat at the moment. A child might be crying a lot and refusing to eat and you are unable to understand what the problem is I think that this heat is the cause of that.

**R1-**Personally, a lot of the time children complain about stomach aches. They do not diarrhoea but they do suffer from stomach aches. The food that they consume is what they eat regularly and not something foreign so you wonder what might be the cause. I later came to realize that where we live we are surrounded by sewers and dirty water. My son contracted diarrhoea and when he was taken to the hospital, he was suspected of having typhoid so I think that the environment and our surrounding is likely the cause of this problem.

**R6**-On my part, I would say typhoid which is water borne. In our community, there are taps that go through sewer lines. You might find out that a broken tap that is exposed to the sewer is what is used to supply water to you. We buy the water from these taps at high prices sometimes paying twenty shillings for a twenty-liter jerrycan of water. And because not everyone has access to water treatments or cannot afford to boil water due to fuel costs small children that drink this water end up falling sick and contracting typhoid.

**3. If you were to rank these illnesses in order of priority, what would you rate as the top three**

**diseases affecting children**

**[If diarrhoea is not listed in the top three priority problems -Do you think diarrhoea diseases**

**are a big problem in this community or not? Why do you say so?]**

**R2-**The first is cholera and diarrhoea, the second is fever and the third is respiratory diseases. Respiratory diseases are caused by climatic changes that are brought about by the factories in our community which release smoke into the air thereby affecting our children.

**R4**-The first is fever, the second is common flu and the third is diarrhoea. I am particularly saying fever because I have witnessed a child suffering from fever to the point that they cannot wear clothes. Their body temperature and fever rise and they become weak. For example, if you have five children four will be affected with this fever. It stays for about fifteen minutes until the child becomes unconscious. However, after being tended to they become okay.

**R7-**The first is diarrhoea. This is because in our area sewers are all over. There is no place where there is no sewer so you find that there is exposure to a lot of dirt. We also do not have a proper waste disposal system and people dispose of their waste anywhere. The dirt is not collected on time so there are many flies surrounding the disposed waste. The second illness is fever and the third is measles.

**R5-**The first is diarrhoea because the water that we use as a community comes from taps which are exposed to sewers. Not everyone has access to water treatments or boiling the water and the bacteria causing cholera is a lot in the water. When children use this water, they become affected by diarrhoea. The second illness is respiratory diseases caused by the smoke from the factories in Ruben. This affects children a lot because their immune system cannot be compared an adult’s. The third is common which is caused by climate changes in the environment and poorly disposed waste.

**R8-**The first is fever and the second is diarrhoea. My child used to complain about stomach aches and suffered from diarrhoea a lot I however took him to the hospital and he is now fine. The third one is tonsillitis because the child complains of pain in the throat while swallowing food.

**R1-**According to me, I think that because of the environment we live in and the water we drink diarrhoea is likely to be the first. The second one is stomach aches because of drinking water. You might boil your drinking water at home but your children may end up drinking unboiled or untreated water in school. The third is common flu caused by air that comes from the waste disposal sites and dirty environment.

**4. Can you tell me the health services/facilities available in this community? Where do you**

**access health services? [Probe: how much does it cost to access these services, how long do**

**people have to travel to access the services?**

**R8-**I usually take my child to Our Lady of Nazareth or City Council when they are sick. I walk to these facilities because they are nearby and do not incur any transport costs. Both hospitals do not charge you for treatments apart from times when you are prescribed medicines to go and buy from the chemist.

**R3**-When my child is sick I usually take them to Government hospitals like Njenga Level Four and Mukuru Health Center. I walk to these hospitals because the are not far from where I live. For most of the times you are not charged anything for children under the age of five but you might have to buy medicines from the chemist.

**R9-**I take my child to Ruben Center because it is close to me and I am used to it. I walk there and do not use a car or boda-boda. There, I pay fifty shillings for the card and other charges may depend on what the doctor will say for instance you might be told to go to the lab and therefore you will have to incur some costs for that. There are however many organizations there like children organizations which can help cater for medication costs of the child if you are financially unable to do so. You talk to a CHP who takes you to a social worker who will in turn take you through the process and cater for the costs.

**R10-**I take my child to Ruben Center which is a faith-based organization because that is where they were born and where they attended all of their clinics. It is nearby so I walk there. You are usually charged for the card and medicines.

**R4-**For me It depends on when the child falls sick. If the child falls sick during the day, I take them to Maendeleo Hospital which is just here in Ruben but if the child falls sick at night, I cannot wait until the morning to take them to hospital so I take them to a chemist nearby called Kwa Dennis to get treatment as we wait for the morning hours to come. We still feel that the chemist is good because there are times when you go to the hospital and don’t find medicines there so you will still end up going to the chemist to buy the medicine. I walk in order to get there since it is not a long distance.

**R5-**Hospitals within walking distance are Maendeleo and Ruben Center. This is where we take children if they fall sick during the night and if they are not in critical conditions we wait until the morning to take them to the hospital to get treated. If the medicines are unavailable there then we get a prescription to buy them from the chemist. I cannot go to the chemist to buy medicine without a doctor’s diagnosis since I cannot guess what a child is suffering from.

**R7**-I take my child to MMM but that is if it is during the day if it is at night, I might buy them paracetamol to relieve pain and take them to the hospital in the morning. I buy paracetamol from a nearby chemist. I use a boda-boda to get to the hospital and it costs me one hundred shillings.

**R2**-Depending on the hospitals that are near me, I can walk to Kware and can also walk to MCC but it takes me a bit longer so I take a motorbike in order to get there faster. Sometimes a child might fall sick during the night so I buy some medicines from a nearby chemist and when symptoms persist I take them to the Government hospitals the next morning. At the Government hospitals, you are not charged anything for children under five years.

**R1-**I take my children to Our Lady of Mercy which is accessible even during the night since there are doctors there anytime you go. I do not buy medicines from the chemist despite being surrounded by many because maybe the one selling you the medicine does not even know the child’s diagnosis. I take my children to city council hospitals because they have good doctors.

**5. How do most people respond when a child has diarrhoea in the home? [Probe: What do**

**people do at household level? at community level? Where do they go to access treatment? Do**

**they take antibiotics? Where do they access antibiotics? Why do they access antibiotics?]**

**AT HOUSEHOLD LEVEL.**

**R4-**If the child falls sick during the time which I can easily access the hospital then the first action I would do is take them to the hospital but if they fall sick during times I cannot access the hospital then I give them warm water mixed with a little bit of sugar and salt.

**R3-**I first asses their condition and if I am able to take them to the hospital I do so. If I am unable to, I take some water and mix it with some sugar and salt and give it to them

**R1-**It depends with the time they start diarrhoea. If it is during the night then I go to a nearby chemist, explain the child’s condition to a doctor there and get some medicine to help the child. The child becomes weak when they diarrhoea so I cannot stay with them in the house so I rush them to the chemist instead because the doctor there might have some knowledge on how to help me even if their knowledge is limited.

**R1-**The first thing is that as a parent you are not a doctor so you do what you think will help the child before you get to the hospital.

**AT COMMUNITY LEVEL.**

**R7-**There is a mother who told me that she mixes uncooked rice with water and gives the water to the child. This water immediately stops diarrhoea.

**R9-**There are some that give bread to the child. They cut it into small pieces and give the child. This then stops their diarrhoea. Others also crush Panadol mix it with water and give it to the child. There are many doctors and funny home remedies.

**R6-**There is a woman who told me to mix wheat flour with warm water into a smooth paste and give it to the child. This stops the diarrhoea though I have never personally tried it.

**R3-**I have also heard of the bread method and it works even for adults. I have also heard of the flour and water mixture and that it works too.

**R1-**There is a tree that has roots back in my rural area. The roots of the tree are taken and crushed. It is then mixed with water and another herbal medicine is chewed by someone and then applied on the child. This then stops the diarrhoea.

**R2-**In my rural area I was taught by my grandmother to use roots from a mango tree. You peel the first layer off it to reveal another layer beneath the root. You then crush it and mix it with some warm water. This stops the diarrhoea in both children and adults.

**R9-**I have heard that sometimes diarrhoea is caused by the evil eye. You apply a certain oil on the stomach of the child and it releases sand, sometimes hair or funny things. The evil eye is associated with different tribes like luhya and kisii.

**R8**-I have also heard of the evil eye theory and know that sometimes it is associated with Luhya‘s,Kamba's or Kisii‘s. It is said that the Kisii‘s is the worst. The child has severe diarrhoea and fever and if you take them to the hospital to get an injection they die. They should not be injected or taken to the hospital. We take the child to a traditional healer who knows how to deal with such things.

**R1-**This evil eye theory is different in many tribes. For the Kisii, we were taught that if you are walking and suddenly feel dizzy, you look for dried precious stones and hit the woman until she bleeds, then you get better. For children, you boil a shilling in water and give the child that water to drink. Thereafter, you apply some oil on their stomach and after that they get better.

**WHERE DO YOU GET ANTIBIOTICS?**

**R2-**We get them from the chemist.

**R4**-I know antibiotics but I am afraid of using them because they affect your immunity if you do not use them properly.

**R10**-I cannot buy antibiotics unless I get a prescription to buy them

**R9-**As for me, I cannot wait for a doctor’s prescription to buy them because sometimes you are busy and cannot go to the hospital so you just buy painkillers from the chemist and take them.

**R7**-I also buy from the chemist because there you are given according to the money that you have.

**R3**-The biggest mistake is that when we go to the chemist to buy medicine the first thing that they do is give you antibiotics for however much of money that you have, it will still fit within your budget.

**R1-**If I am lucky to get it for free from the hospital then that well and good but if I have been given a prescription then I go to a nearby chemist and buy it from there. However, that is where the problem occurs because if I do not have enough money to cater for the whole dosage then I am given a dosage that fits within my budget. If I get better before completing the doctors prescribed dosage, I do not bother to buy the medicine again and if I don’t I continue buying within my budget.

**R8-**Sometimes I might borrow from my neighbor if I need the medicine at night. If I get better in the morning then I di nit bother about it but if I don’t I go to the hospital.

**R2-**For antibiotics most of the time people do not finish their dosage so you will find that they store it and use it later on when someone becomes sick.

**6. Can you tell me some of the enablers and challenges that people experience to access**

**treatment for diarrhoea diseases?**

**ENABLERS IN ACCESSING DIARRHOEA TREATMENT.**

**R9-**The hospitals are nearby, the roads are good, the doctors at the hospital are available and there are CHVs who are allocated a household each, go by giving out water treatments and show you how to treat your water. They also visit you each month to track your progress and give you medicines or any coming from the Government.

**R1-**When my child contracted diarrhoea, I took them to the hospital together with my wife. There we were educated on water treatments and boiling drinking water in order to prevent diarrhoea in the future. We never used to previously boil the drinking water because my wife felt like boiled water was not as satisfactory or thirst quenching as unboiled tap water however, the doctor advised us to start boiling our drinking water. We took this advice and started boiling our drinking water and even putting some in bottles for our children to take to school with them. We also have CHVs who walk around in the community and give us water treatments like Aqua taps which we use to treat our water.

**R2**-The hospitals are nearby and there are CHVs who help us and sometimes advise us to go to the hospital. The roads are also good and accessible.

**R5-**The doctors at the government hospitals follow up with us in case our child has diarrhoea until they get better, the roads are good and that makes it easy to get to the hospital at any time and there are CHVs who guide and follow up with us incase our child has diarrhoea to ensure that they do not spread the disease and also give us advise on water treatments. The doctors who follow up with us are from KEMRI and they attach a CHV with the sick child. They buy medicines for the child and follow up to ensure that the child gets better.

**R3-**There are labs at the hospitals and doctors who follow up with you incase your child has diarrhoea. They run tests for you and sometimes offer to give you free medicines in case they find the child with diarrhoea. However, because we cannot rely on hospitals during the day only and we need such services at night too, I would request that the doctors available at the chemists to be trained so that when they attend to us they do so in the right way.

**R1**-Here at Mbagathi South, there are many KEMRI officials who help curb the issue of diarrhoea by attaching CHVs to households affected by diarrhoea. This helps a lot since those CHVs follow up with the households to endure that the child gets better.

**CHALLENGES IN ACCESSING DIARRHOEA TREATMENT.**

**R2-**There is stigmatization of households in case one of the children suffers from diarrhoea. This makes you feel like you are no longer part of the community.

**R5-**The water that we use from the City Council comes untreated and during the handling process, the water might become contaminated. We urge the Government to distribute water treatments through the CHVs to us to ensure that this diarrhoea issue is kept under control.

**R6-** When you take your child to the Government hospitals there are long ques there so it might be a while before you are attended to and if you opt to go to private hospitals, the treatments there are costly.

**R9**-Time is a challenge, this is because you might go to the hospital early with intentions of leaving early but the doctors might take tea breaks in between and go up to one hour or more during those tea breaks. They take more time on their tea breaks than the time used to attend to you.

**R7-**Long ques, a lot of people at the hospitals and sometimes there are no medicines at the hospital so you have to buy them outside. Because you do not have money to buy the medicine, you go back home and give the child warm water with sugar and salt. This becomes a waste of your time since you stayed for a long time in the que and were still unable to get the treatment or medicine that you needed for your child.

**R8-**The challenge at the public hospital is the long ques. If you do not go early enough, you will not be attended to early.

**R1-** The challenge that I face is where I take my child for daycare, the carer might not be able to take the child to the hospital incase they are sick and you are also unable to because you are at work. In case you get the chance to take the child to the hospital the que is long and maybe the doctor is not in a good mood or is tired and talks to you the way they want to. This becomes very challenging for us.

**R2-**Povetry contributes to accessing medical care because a lot of people in the community are not financially well off so they are unable to pay for the medication offered by private hospitals. Religion also contributes because there are some churches that discourage or don’t allow people to go to the hospital when they are sick. This affects many children because the parents believe that they should be prayed for and not taken to the hospital when they fall sick.

**R1-**There is no time for doctors to explain to you as a parent what your child is suffering from as they hurriedly diagnose the child and write down prescriptions that you may not understand because the patients are many and time is limited.

**7. What do people do to prevent diarrhoea? [At household level, at community level?]**

**R9-**Personally, I will ensure that the child completes the dosage that they were given at the hospital and follow the guidelines given in the hospital by the doctors like ensuring the child is in a clean environment, they drink boiled water and cooking their food until it is well done.

**R2-**I educate my family on ways of preventing diarrhoea like boiling drinking water, proper hand washing and covering food.

**R1-**I ensure that our drinking water is boiled, that we wash and clean our vegetables before cooking and wash hands before eating any food.

**R2-**Mostly on Sundays I inspect the children in my church and advice their parents on how to care for the children like ensuring that they wash their hands with warm water, they check their body temperature and proper breastfeeding procedure to prevent them from being sick.

**R4-**Mothers should be advised to ensure that their children receive all the vaccinations that they are supposed to get especially the one that will prevent diarrhoea which is Rotavirus. For older children, the mothers should ensure that they finish their prescribed dosages to prevent the problem from re occurring.

**8. How do people in this community perceive childhood vaccines [Probe: why do you think**

**childhood vaccines are widely accepted? Why do you think childhood vaccines are widely**

**resisted?**

**R7-**People are serious about vaccines especially the polio vaccine. They are mostly afraid of their children becoming handicapped so they ensure that they are vaccinated.

**R4-**In our community most parents who have children usually get their children vaccinated because the Government instructs them to do so. They do not know the vaccines given or how helpful it is They lack the knowledge of the importance of every vaccine a child gets from birth.

**R2-**For the vaccines that are taken around in our community, there are many thoughts on that. There are some who think that they are family planning schemes and many funny theories. It is because of this that many children end up not being vaccinated.

**R3**-Parents have a good attitude towards vaccines and take their children to be vaccinated. The problem comes when the adults are to get the vaccines.

**R10-**There was a case in the community where a parent was refusing to get their child vaccinated or even taking them to the hospital because of their faith. There are also some parents who only acknowledge the vaccines that are given at the hospital and does not want the ones being taken around in the community by vaccination officers.

**9. How about rotavirus vaccines? What do people think about rotavirus vaccines? Where do**

**they access rotavirus vaccine? [Probe: What do they think are the benefits of rotavirus**

**vaccines? What concerns do people have with rotavirus vaccines?**

**R2-**Three quarters of the mothers with young children do not know exactly what rotavirus is. Personally, I know what rotavirus is and it protects a child from diarrhoea.

**R7-**A lot of people in the community do not know what rotavirus is and what it does. I know that it is a vaccine that is given to children to stop them from having diarrhoea and my children have received this vaccine.

**R5-**I know rotavirus helps many illnesses in the body especially diarrhoea and builds the child’s immune system however, not many people in the community know of it or of its importance.

**R1-**Not many people in the community know it because the city council hospital does not educate us on it. I however came to know of it when I went to a private hospital and the doctors there explained it to me.

**10. What are the enablers and challenges for people in this community to access rotavirus**

**vaccines? [Prove: cost, distance to access services, cultural/religious beliefs, impact of**

**COVID-19, perception of vaccine safety]**

**R5-**The first and biggest challenge is lack of knowledge because a parent may go to the hospital to take their child but they do not know anything about the child’s diagnosis or what the doctor gives the child. This is because they have not been educated on what rotavirus is and how it helps.

**R7**-I did not face any challenge in accessing the rotavirus vaccine. The vaccines are easily accessible at the city council hospitals and you do not pay for them if you go in time.

**R1-**I can say that even though there are challenges, once you understand about the importance of vaccines it becomes easy for you to comply because it helps the child and protects them illnesses.
