# Supplementary material for: Exploration of the social determinants of diarrhoea, rotavirus vaccine uptake, and vaccine ‘fatigue’ in Ethiopia, Kenya, and Malawi
Source: PLoS One. 2025 Sep 9;20(9):e0319691. doi: 10.1371/journal.pone.0319691 (PMC12419581; doi:10.1371/journal.pone.0319691)
Supplement: S1 Data — (ZIP) [file pone.0319691.s001.zip › Supporting Information Files/MW_3FGD.docx]

**F:** Thank you so much, I will ask you to raise your voice as you are taking.

**00:** (All) Alright.

**F:** I just want to start our discussion by looking diseases that are common among the children here in Bangwe or in your community. In your opinion, which diseases are more common amongst the children?

**P 5:** The disease that is common among the children is Diarrhea and it is common among the children who are developing their teeth.

**P 6:** Flu and cough.

**F:** Mmmmh.

**P 5:** Pneumonia, most of the children suffer from Pneumonia as well Diarrhea.

**P 4:** Most of children suffer from Diarrhea.

**P 2:** Most of the children are suffering from Diarrhea and they also vomit because of what we are going through.

**F:** What do you mean when you say ‘what we are going through’?

**P 2:** It’s because of the scarcity of water and the foods that they eat, these are some of the factors that are increasing cases of Diarrhea among the children here. Mostly, I would say lack of access to safe water is the major factor contributing to more cases of Diarrhea amongst the children here.

**F:** We are still discussing about diseases that are common among the children here in Bangwe and you have mentioned of Diarrhea, Flu, cough and Pneumonia what are others diseases?

**P 9:** The diseases that are common among the children are Flu, Fever and Malaria.

**P 6:** Skin sores

**P 3:** Scabies that are caused due to shortage of clean water and there is a need for the mothers to practice hygiene in their respective homes because scabies are easily transmitted from one person to another. This scabies is also associated with fever.

**F:** Welcome to this discussion madam

**00:** Thanks.

**F:** Since you have found us discussing, your number is seven and each time you are speaking, you should mention your number first. Thank you, you have mentioned of scabies and Measles. Are there other diseases that you are thinking about. We are discussing about the diseases that are common among the children here in Bangwe.

**F:** Measles is also another disease that is common among the children here.

**P 1**: Malnutrition is also another disease that most of the children are suffering from here and it is manifested through stunted growth among most of the children.

**P 6:** Most of the children here suffer from Diarrhea especially when they are about to develop teeth for the first time and this delays some processes such as motor development as well as stunted growth in children.

**00:** Bilharzia.

**F:** What are other diseases that are common among the children apart from those you have mentioned already?

**P 6:** Ringworms.

**F:** That attacks children’s head?

**P 6:** Yes

**P 10:** Some children have ringworms in their faces as well.

**F:** You have mentioned many diseases such as Malaria, Pneumonia, Diarrhea, Measles, Ringworms, Flu, Cough, you have mentioned a lot of diseases including Bilharzia, right?

**00:** (All) Yes.

**F:** In your opinion, which diseases do we consider as burden among these ones?

**P 2:** Persistent Diarrhea among most of the children.

**P 5:** Vomiting, most of the children also vomit when they are having diarrhea.

**F:** Meaning that they vomit when they are having Diarrhea?

**P 10:** In the past few months most of the children were suffering from Measles.

**F:** Is Measles the same as chicken pox?

**00:** (All) They are different.

**P 4:** The diseases that are commonest among the children are Diarrhea and Measles.

**F:** Is chicken pox common here?

**00:** (All) Yes.

**00:** Scarbies because they are easily transmitted from one person to another.

**F:** What else?

**00**: (All) Silent.

**F:** What about Malaria, is malaria not a big issue here?

**00:** It is an issue but not that much as compared to other disease.

**F:** Maybe let’s start with Malaria. What makes you think makes that Malaria is not an issue in this community?

**00:** I believe it’s due to the fact that most of the people are now aware that Mosquitos are responsible for causing Malaria and they protect themselves from the Mosquitos hence Malaria is not a problem but we are having more cases of Diarrhea. This is due to the fact that most of the people here are not aware of what exactly causes Diarrhea.

**00:** Diarrhea is commonly most among the children of the women who does not attend antenatal clinics.

**P 6**: Most of the children are having Diarrhea because of they were not vaccinated. There is a vaccine that each and every child is supposed to receive and it is administered in form of injection in both legs. Most of the parents are not willing to get their children vaccinated against Diarrhea and this is causing more cases of Diarrhea among the children here.

**F**: What's the name of that Vaccine?

**00:** We are told that it prevents children who are developing teeth for the first time from having Diarrhea.

**00:** (All) It is called Rota virus vaccine.

**00:** Most of the children who got this vaccine do not suffer from Diarrhea when they are developing teeth for the first time.

**P 2:** I just heard that children usually have Diarrhea when they start to develop teeth for the first time but I did not experience this with my child because she got that vaccinated and she is now healthy.

**P 5:** Me as well, my child got vaccinated with Rota virus vaccine but she didn’t have Diarrhea when she was developing teeth for the first time.

**00:** When children get vaccinated, they do not have Diarrhea when they are developing teeth for the first time and I would therefore say that Rota virus vaccine is very helpful.

**F:** P 8, we haven’t heard your voice today.

P 8: I would say that this vaccine is very helpful.

F: Please raise your voice.

P 8: This vaccine is very helpful because it helps children not to suffer from Diarrhea, my four-year child did not suffer from Diarrhea when she was developing teeth due to this vaccine and the doctor told me that teeth development in children is not correlated with Diarrhea but is something to do with hygiene. So, I can conclude that Rota vaccine is very important.

**F:** Let's go back to the point that P 1 raised that cases of Diarrhea are common among the children of the women who do not attend antenatal clinic and we have heard about the advantages of the Rota virus vaccine. Now I just want to hear about factors that prevents women from getting their children vaccinated. Of course, one of you highlighted that some women complain that nowadays children are subjected to different types of vaccines. What other factors do you think prevents women from getting their children vaccinated?

**00:** Some religious beliefs.

**F:** Make no noise please, what factors do you think prevents most of the women from getting their children vaccinated?

**P 2:** Some people do not vaccinate their children because of their religious beliefs but to me this is infringing the right of the children to get vaccinated since each and every child has the right to get vaccinated. So, I would say that most of the parents we are behind increase in cases of Diarrhea among the children here. This is due to the fact that some of us we do not allow our children to get vaccinated and I would say that the religious beliefs that restricts people from getting their children vaccinated are not good.

P 5: It’s not only religious beliefs but also some people believe in rumors about the vaccines that are speculated by their neighbors. Some of the people claim that this vaccine (Rota virus vaccine) is associated with Satanism because it was not there before in the past.

**F:** Each one of us should hold the recorder in her hands so that the voice should be recorded clearly.

**00:** In the past we used not to have these kinds of vaccines why now as such they associate the vaccines with 666 concept and when one is not strong enough she decide not to go to the clinic to get her child vaccinated.

**P 5:** I would say that another thing is lack of information about the vaccines among the women with under 5 children here, if women could have enough information about these vaccines that are administered to children, it could be easy for them to allow their children get vaccinated. But in most cases, most of the women do not have adequate information about these vaccines and they are only asked to get their children vaccinated at the hospital.

**P 6:** Lack of understanding whereby each and every woman has her own understanding of information about the vaccines, some of the women are able to get correct information about the vaccines while others not. For instance, during the time when COVID-19 vaccine was introduced people were saying that once one get vaccinated, he/she was dying and the same was also being spoken about Cholera vaccine. As such most of the people believe that vaccines are introduced to kill and not heal people and these prevents parents from getting their children vaccinated. Let me add something on the issue of the religious beliefs, most of the people including the health care workers restrict their friends from getting their children vaccinated. There are some health care workers who usually tell people in their communities that they just place their children where they are exposed to sunlight for and they get healed from different diseases and it becomes difficult for someone who got such information from the health care workers themselves that the vaccines are bad and that is not good children to get vaccinated.

**F:** What do you mean when you say they expose their children to sunlight?

**P 6:** Some of the health care workers claim that vaccines are developed from sunlight and that sunlight is responsible for boosting immune system of each and every child and not these vaccines that are administered to the children.

**F:** Some people expose their children to the sunlight for them to get healed?

**P 6:** Yes because they have heard from the health care workers themselves that children ought to be exposed to the sunlight for them to healed from different diseases and this is also common among the people from other religious groups.

**F:** Religious beliefs, lack of information and ignorance, are there other factors, we are not trying to mean that those who do not accept vaccines are wrong, right?

**00:** (All) Yes.

F: Like I said earlier, each one of us has freedom but we should look at some of the factors that prevents people from getting their children vaccinated. We are not here to judge people but we just want to explore some of the factors that restrict people from getting their children vaccinated.

00: (All) Silent.

F: What about factors that encourages people to get their children vaccinated?

**00:** It depends on how the HSAs convince and interacts with the people in their areas of jurisdiction. This is due to the fact if there no good working relationship between the HSAs and people in the communities, of course it is difficult for people to accept that their children should get vaccinated but when there is good working relationship, it means that the HSAs can tell the people about the importance of the vaccine and disadvantages of not getting vaccinated. In turn this can make it easy for people to accept that their children should get vaccinated.

**F:** Our friend has pointed our relationships between the HSAs and the people in the communities, others what can we say about this issue?

**00:** (All) Silent.

**F:** Or are there other factors that encourage people to get vaccinated apart from good relationship between the HSAs and the community?

**00:** (All) Silent.

**F:** You have said that it is in the recently people are having negative attitudes towards the vaccines, right?

**00**: (All) Yes.

**F:** And you also mentioned about the coming of COVID-19?

**00:** (All) Yes.

**F:** I just want us to go back, what were people saying about vaccines before the introduction of COVID-19 vaccine?

**P 2:** Before COVID-19 vaccine was introduced, people had no issues with vaccines, I would say that before the introduction of COVID-19 vaccine, people were able to allow their children to get vaccinated different types of vaccines that are administered to children easily. However, after the introduction of COVID-19 vaccine, people are having negative attitudes and fears towards any vaccine. For instance, someone else mentioned that the HSAs do come in our respective homes, yes, they are coming and inform us about vaccines but still most of the people have negative perception towards the vaccines especially vaccines for children.

**P 5:** In the past the vaccination was not an issue and people had no problem with the vaccines but when Polio vaccine was introduced, most of the parents are not willing to get their children vaccinated because they don’t understand how the vaccine is administered. Polio vaccine first dose requires that children should receive 4 injections and the second one also involves 4 injections but most of the people in the communities do not have adequate information about this and most of the parents are suspicious about this. Some of the parents are willing to allow their children get vaccinated when they have adequate information the same also happened with Rota vaccine.

**F:** What questions do most of the people have or ask about Rota virus vaccine?

**00:** (All) Silent.

**F:** What do most of the people say about Rota virus vaccine? I mean questions, comments, worries, etc.

**P 6:** Mine is just a comment, most of the people claim that because of Rota vaccine, most of the children who got vaccinated are not suffering from Diarrhea and that most of the parents are not worrying that their children will suffer from Diarrhea when they are developing teeth for the first time which was the case before this vaccine was introduced.

**F:** P 7 do you have anything to add.

**P 7**: I have nothing to say.

**F:** This one said that in the past before the coming of COVID-19 vaccine things were not difficult in terms of vaccine for children that are administered to children.

**00:** (All) Silent.

**F:** Now I just want to know what was happening that was making it easy for people to accept the vaccines for children in the past that is not happening now?

P 6: Like with the coming of chicken pox here in Malawi, most of the health care workers were thinking that it was just like any other sores but when they realize that it was a disease they came up with a vaccine. Before the vaccine was being introduced to the people, they were mobilizing together people in and informing them about the importance of the vaccine but as of now such messages are being disseminated through social media platforms and radios but the problem with is that not everyone has access to these. As such now it is a problem for people to accept the vaccine because they don’t have adequate information before these are vaccines and most of the people are surprised that as of now each and every year, a new vaccine is introduced to the people which was not the case in the past. It could have been better if the people could be informed about the importance of the vaccines before they are introduced to the people and I have just heard from this meeting that there is a new Polio.

**00:** Lack of civic education because if the people have adequate information about the vaccines, it can be easy for them to accept the vaccines.

**F:** Here in Bangwe, where do you gets vaccine for your children?

**00:** At Bangwe health center but sometimes the HSAs bring the vaccines in our respective communities but Rota virus vaccine is only administered at the antenatal clinic only. However the some women do not allow their children to get vaccinated at the antenatal clinic because they have limited knowledge about this vaccine.

**F:** I just want to know other factors that encourage women to get their children vaccinated.

**00:** Some people are aware of the advantages of the vaccines so they allow their children to get vaccinated on voluntary basis while some are persuaded by the health care workers or their friends to get their children vaccinated.

**F:** How far is the health center from your community?

**00**: I stay near the clinic.

**F:** How much does it cost to travel from the areas that are far to get to the clinic?

**00:** From Mpingwe to the clinic, it is very far.

**00:** Sometimes the HSAs bring the vaccine in our respective homes.

**00:** Sometimes the health care workers bring the vaccine in primary schools and people bring their children in these so that they should be vaccinated.

**00:** In most cases the HSAs bring the vaccine in our homes and if the child is not vaccinated, it means that it is a parent who has a problem.

**00:** To say the truth the HSAs here in Bangwe are trying their best because sometimes they find us with the vaccines in the places where we fetch water and they tell us that we should be free to approach them if we face any problem with the vaccines or if we want other things like ORS anytime.

**F:** Alright, like what you have said about the vaccines, it is really nice to hear your experiences with the vaccines. There is something that I want to hear, as parents do we have any worries or concerns when our children are getting vaccinated? Please, let’s be open

**00:** The worry that we usually have is that some of the children cry the whole day after being vaccinated that’s what I see as the major problem but it is not that big issue.

**00:** Some of the children's arms swell after getting the vaccinated.

**00:** For instance, my second born child was given an injection at Limbe health center and the one who was administering the vaccine was a trainee and when he did that, my child was bleeding heavily and the in charge of the nurses at the clinic came and apologized to me for what happened. After that my child developed something that looks like a ball when she was injected and that thing is still visible to date. To me the vaccines are not bad but the way how some of the health care workers handle our children when they are administering the vaccines.

**F:** Making children to cry, causing swelling of the arms and legs, what other else?

**00:** Fever.

**F:** What else?

**00:** Mine is just a comment, these days teenagers are the ones who are active in as far as child bearing is concerned, so such kind of people doesn't know how to handle a child when he/she is being given an injection and this is one of the major factors why children bodies swell after being vaccinated through injection which is different from the adults.

00: I just want to add that it is not recommended that a woman/parent should hold his/her child when he/she is being given an injection because that’s the responsibility of the health care workers. However, nowadays, most health care workers usually instruct parents to hold their children when they are injecting the children.

**F:** What do you do when your children are having fever, having their arm or regs swelling after getting the vaccine?

**P 2:** We usually buy pain killers such as Caffeino to deal with any side effects of the vaccine.

**P 1:** I ensure that I get the child bathed in warm water.

**F:** I just want to be clear, after getting the vaccine, some of the children experience side effects and these makes us parents to have some fears and worries. That's what we experience in some instances, how does this influence your decision to get your children vaccinated later? I just want to hear your honest views on this.

**P 6:** It’s really something that is worrisome when dates that your child expected to get vaccinated again is approaching , some parents are worried that when their children are vaccinated, they don’t sleep because children usually cry during the night after getting vaccinated.

**00:** Everything has its own advantages and disadvantages, the way how children react to the vaccine is different, some children don’t experience any problem after getting the vaccinated while others experience side effects. My child usually experience fever after getting vaccinated but I have no worries with this because I know that after sometime, he will get better.

**F:** I want to hear from others, P 7 and P 9, I would be happy to hear your views on this, you have been quit for a while. There was an issue about beliefs that was raised and I have heard that some people expose their children to the sunlight, right?

**00**:(All) Yes.

**F:** Are there other beliefs that prevents people from getting their children vaccinated?

**P 7:** There are some people who doesn’t take their children to the hospital and they don’t allow their children to get vaccinated because of their religious beliefs and some of our friends who belong to these religious they speak about this when we are interacting. However, I heard on the radio that there was a certain couple that was not allowing it’s children to get chicken pox vaccine and children from this family became seriously ill until the government intervened and this made their neighbors took the child to the hospital but it was too late and the child died while in transit. So, I would say that there are other beliefs that prevents people from vaccinating their children.

**F:** P 9?

**P 9:** Sometimes it’s because of the beliefs that us parents hold, we believe that there is nothing wrong in getting our children vaccinated or not. However, as parents there is need to know that each and every child deserves to get vaccinated because that's one of their rights and by not allowing them to get vaccinated, it means that we are violeting their right to get vaccinated and sometimes us parents we get carried away by what is speculated on social media that the vaccines were made to kill people so as to reduce human population. For instance, there were some of the messages that were circulating on social media that Polio vaccine was introduced to kill children. However, there is a need for us parents not to believe whatever is said on the social media.

**F:** Apart from what is speculated on social media, you have said that us parents we have our own beliefs in our households. What are those beliefs that discourage you from getting your children vaccinated?

**P 9:** In most cases, some of the parents just believe that there is no need for their children to get vaccinated and some of the parents hold a belief that in the past they were not giving any vaccine to the children and children were able to grow without any problem. I would say that there are some of the religious and traditional beliefs that discourages parents from getting their children vaccinated.

**F:** For those who do not allow their children to get their children vaccinated, do their children grow healthily?

**P 2:** Most of the children from the families that restrict their children from going to the hospital do not grow healthily such that their children are characterized by stunted growth and that their bodies usually have sores and scars. For instance, there was a certain family which had seven children and they lost 5 children because they were not willing to allow their children to go to the hospital and when one of their children got sick, the village head threatened them that he is going to report what they are doing to the police and the police were involved. When the man heard that the police were informed about what his was doing, he had to run away leaving his wife behind and the wife took the child to the hospital. At some point in time the man got sick and he was not going to the hospital and he died, as of now wife is left with 3 children and she now goes to the hospital with the children whenever they are not feeling well. This made the woman to realize that it is good to take the children to the clinic when they are sick.

**F:** We have discussed a lot about the vaccine, I remember at some point we were discussing about the diseases that are common among the children here in Bangwe, so I just want us to pick three diseases that you think are a burden among the people here in Bangwe.

**00:** Diarrhea.

**00:** Measles.

**00:** Pneumonia.

**00:** Scarbies

**F:** You have mentioned four diseases, so out of these diseases which one is our number 1?

**00:** Diarrhea.

**F:** Diarrhea.

**00:** (All) Yes.

**F:** So apart from the fact that some of the people do not allow their children to get Rota virus vaccine, what other factors do you think contribute to more cases of Diarrhea among the children here in Bangwe?

**00:** (All) shortage of clean and safe water.

**F:** One by one please.

**00:** Because of scarcity of safe and clean water.

F: What do you mean when you say lack of safe and clean water?

**00:** Here water that is fetched from the wells and it is consumed by the children before it is treated, that's what we call unsafe water.

**00:** There are some areas here in Bangwe with taps, boreholes and covered wells but the problem is that water that us fetched from the wells and boreholes is not safe and it needs to be treated before it is consumed. And there is a need for the children to know places where safe water for drinking is found in the household and this is the responsibility of the parents and this will ensure that the children are drinking safe water always. in addition to this, if people don’t have money to buy chemicals that used in treating water, it is important that the water should be boiled and sieved using a clean cloth before it is consumed.

**F:** Lack of safe and clean water is one of the contributing factors, what else apart from this?

**00:** Most of the children consume untreated water.

**P 2:** Just to add, most of the people dispose the used baby diapers in the rivers where most of the people fetch water that is used for different domestic from and this water is used before it is treated. This is an issue here in Bangwe, most of the people use rivers as dumping sites for baggage include used baby diapers.

**00:** Some of the people use buckets that they use for bathing to fetch water that is used for drinking.

**00:** Eating unclean food is one of the contributing factors among the children here in Bangwe. Most of the parents are lazy in preparing food for their children at their households such, they just buy food such as *'Mandasi'* (locally made snacks) that are usually sold uncovered along the roads.

**F**: We need to finish this discussion; we are approaching the end.

**P 5:** Most of the women especially the lactating mothers do not practice hygiene when preparing food for their children and most of these women do not wash their hands when they want to breast feed their children. Some women do not wash their hands after visiting toilet hence there is a need for lactating mothers to wash their hands with soap before breast feeding their children.

**00:** Just to add on these, what is more important is to ensure that we practice hygiene, we (women) don’t take a responsibility of taking care of our families properly, the problem of Diarrhea will still persist in our families. We need to make sure that our children are always eating warm and clean food and we should make sure that the water bucket that is used for bathing and washing should not be used to fetch water meant for drinking.

**F:** You have highlighted things that we need to do to avoid Diarrhea but, in our opinion, what strategies do you follow to prevent Diarrhea? measures that are used at home as well as community?

**P 6:** During the past, we didn't have boreholes in our communities such that we were relying on the water from the wells that are found along the rivers but as of now at least we have boreholes that are found here and there. Most of the people now opt to fetch water in these boreholes and not in the wells and this has really helped in reducing cases of Diarrhea among the people.

**00:** The major contributing factor of cases of Diarrhea here in Bangwe is lack of safe and clean water for drinking, if most of the people could have access to clean and safe water, cases of Diarrhea could have been few and there is also a need for people to treat water with Chlorine or water guard before it is consumed.

**F:** What do you think should be done to prevent disposal of garbage including baby diapers in places where most of the people fetch water here in Bangwe?

**P 1:** It can be better in people can dispose used baby diapers in the waste bins at their households or set them ablaze. It could also be better if the Blantyre city council could be providing people in the communities with the waste bins so that people should throwing their wastes there.

**F:** Are there any efforts being made by the Blantyre city council in as far as waste management is concerned here in Bangwe?

**00:** Nothing is being done.

**00:** And they haven’t reserved any place to be used as a dumping site for garbage here.

**F:** Where do most of the people dispose their wastes?

**00:** (All) Rivers.

**F:** In the same rivers where people fetch water for domestic purpose?

**00:** (All) Rivers.

**00;** That's why we are still having more cases of Scarbies among the children here because water from the same rivers is used to bath the children.

**F:** Alright, what strategies do you use to help/cure those people who are suffering from Diarrhea?

**P 2:** In a situation where a child is refusing to be breast fed, we make a solution of sugar and salt and give it to him/her when he/she is having Diarrhea. We also practice excessive breast feeding if the child is refusing to be breast milk. In addition to these, there is also a need to get children immediately to the hospital when he/she is having Diarrhea.

**P 1:** In a situation where a child is having Diarrhea during the night, are encouraged to go and seek for assistance in form of ORS from the HSAs who works and reside in our communities and we need to take the child to the hospital the first thing in the morning.

**P 3:** There is a need for the parents to keep ORS in their house to give it to their children whenever they start having Diarrhea.

**F:** Can we say each and every parent manage to get his or her child to the hospital when his/her child is having Diarrhea?

**00:** Not everyone takes his/her children to the hospital when they are not feeling well to the, some just believe that there is no cure of Diarrhea and that the children get better on their own but when it comes to the adults, most of them go to the hospital when they are not feeling well.

**F:** What makes some of the parents to rash to the hospital when the child is having Diarrhea?

**00:** In most cases, most of the parents takes the child to the hospital when his/her condition becomes worse especially when the child is showing serious signs that's when they decide go to the hospital.

**F:** Meaning that it's the severity of the illness that prompts most of the parents to go to the hospital with the child but when the child is not showing serious signs it means that they cannot take the child to the hospital?

**00**: (All) Yes.

**00:** It depends but sometimes it’s good to take the child to the hospital before the condition becomes worse and if the child died, the parent is responsible for that.

**F:** Now I just want us to discuss what normally happens when the condition of the child becomes worse. What do you?

**00:** Some of the people believe also believe that when a child is having Diarrhea, it means that his/her parent stepped on a milk at the graveyard from another woman who is breast feeding. In such the societies who believe about this, women whose child are suffering from Diarrhea are instructed to go and pour milk from their breast at the graveyard for the child to be cured from Diarrhea and am one of the people from such societies, I remember when I was at the village we were told that whenever we are going to any funeral, we should apply some breast milk on our feed so as to prevent our children from Diarrhea in case we step on the breast milk from another woman.

**F:** (Laugh).... We are not here to judge anyone but to learn from one another (laugh).

**00:** Some traditional believe (Laugh)

**P 2:** There are some of the traditional beliefs, some of the people believe that when a child is suffering from diseases such as fever and Diarrhea, it simply means that the woman has Chlamydia locally known as 'Mauka' and Diarrhea simply shows that the child has acquired the disease from his/her mother. In a situation like this, the mother is forced to take some herbs so that the child should stop suffering from Diarrhea, this is mostly done when the child producing green stools.

**F:** Does that help the child from having Diarrhea, I mean after the mother take some herbs?

**P 2:** This does not help but some people believe that when a child is having Diarrhea, it means that his/her mother has Chlamydia and some believe that it is normal for a child to have Diarrhea when he/she is developing teeth.

**00:** When a child is having Diarrhea, some of the people believe that the woman have had sex with another man who is not the biological father to the child and that for the child to get healed, there is a need that after having sex, the woman should wipe the sperms from another man with a cloth and use that cloth to wipe the child after bathing him/her, in that way the child cannot have Diarrhea the same day(Laugh).

**F:** A lot things are happening to our children (Laugh).

**P 2:** Some people also believe that when a child is having Diarrhea, it means that the father of the child had sexual intercourse with another woman.

**F:** Alright thanks, we are about to finish this discussion, apart from Diarrhea, what other disease can you say is more common here?

**00:** Measles

**00:** Malaria not measles.

**00:** Malaria.

**F:** Why do you think Malaria is a serious problem here in Bangwe?

**00:** Believes also play a role, some people refuse to sleep under a mosquito net because they believe that there are some chemicals that are applied to the mosquito nets that are responsible for causing impotence and some people believe that the it is hot to sleep under a mosquito net.

**00:** Some of people believe that mosquito nets attract bed bugs especially the ones that are distributed for free by the government.

**00:** Some of the people believe that sleeping under mosquito nets causes them to have difficulties in breathing.

**00:** In most cases, Mosquitos are able to multiply easily when there are some stagnant waters around our households hence there is a need to practice hygiene in our households.

**00:** Just to add, the presents of shrubs in our households encourages the spread of Mosquitos since they act as the breeding grounds for the mosquitos.

**F:** You are telling me that here in Bangwe there are more stagnant water and shrubs that acts as bleeding grounds of mosquitos, right?

**00:** Yes.

**F:** And that some of the people are not willing to use some preventive measures such as sleeping under a treated Mosquito net?

**00:** Yes.

**F:** What else do you think contribute to more cases of Malaria here in Bangwe?

**00:** Some of the people do not have the nets and do not want to buy the nets on their own and they just wait to receive the nets that are distributed for free. Some people stay far from the hospital so it’s not easy for them to go and get the nets at the hospital.

**F:** How often do the government distribute the nets?

**P 2:** Mostly people are given the nets at the antenatal clinic and it is usually women, sometimes we are told to go and get the nets at the hospital on a specific date but when we get there, we are told that they are run out of the nets. Due to lack of money, most of the people does not manage to buy the nets.

**F:** You have said that women are given the nets whenever they are pregnant?

**P 2:** Yes.

F: What about others who are not pregnant?

**00**: Yes, are not considered, consideration is only to the pregnant women.

**F:** Aright, meaning that apart from the believes and unwillingness to use the nets, some of the people are not able to use the nets because of the scarcity of the nets?

**00:** Yes.

**F:** Which disease do you consider as third after Diarrhea and Malaria?

**00:** Measles.

**00:** But it is not that common and there is a vaccine for this disease.

**P 1:** Measles as a disease somehow brings some unhygienic practices among the people. This is due to the beliefs that some people have regarding the disease. Some of the people believe that for the child to get healed from the measles, the child should not get bath until he/she is healed.

**F:** They should not be bathed after they have been given some medication or not?

**P 1:** After they have been given some medication.

**00:** Some of the people apply ash on the whole body of the child as a cure for measles.

**F:** We all agree that Measles is a third one or there is another disease?

**00:** (All) it should be measles.

**F**: Why do you think there are more cases of Measles here in Bangwe?

**00:** It is caused by changes in weather patterns because even those children who are vaccinated against this disease also suffer from this disease.

**00:** Some of the parents restrict their children from getting vaccinated because they don't know how the vaccine works.

**F:** Meaning that most of the people do not have adequate information about the vaccine for Measles?

**00:** Yes.

**F:** Why do you think most of the parents do not ask the health care workers on information about the vaccine or any other health issues?

**00:** Sometimes it’s the attitude of the health care workers towards us, some of the health care workers are rude and some are not usually willing to provide answers to the questions that the patients have.

**00:** Yes, I would say how the health care workers respond when we approach them discourages us from asking some questions that we might have. For instance, I was called at a primary school where my child is learning and I was asked if I was willing that my child should get vaccinated and I accepted. There were some people who were trying to ask the people who were administering the vaccine about the important of the vaccine but they health workers were telling them that if they were not willing that their children should not get vaccinated, they could go.

**00:** Some people are afraid to ask the health care workers because they are afraid of the health care workers while some it’s because of ignorance while others do not ask because of the physical appearance of the health care workers.

**F:** Alright, now I just want to hear from you. What do most of the people here in Bangwe when they are not feeling well for them to get better? be it children or adults.

**00:** For us women and children, in most cases when we get sick, we usually rash to the hospital but this is not a case with men who are usually reluctant to go to the hospital when they fell sick.

**00:** Most of the men are not willing to go to the hospital when they get sick and even it is really difficult for men to get tested for HIV. I remember there was a certain couple in our community who agreed to go and test for HIV at the hospital but when they got to the hospital, it was the woman who got tested because the man escaped at the hospital. The woman tested positive but the man was not willing to get tested. So, I would say that most of the people are not willing to go to the hospital as compared to the women.

**F:** Now, what do most of the men do for them to get better when they are sick since they don’t like going to the hospital?

**00:** They just buy Indocin.

**00:** Some just buy Brufen.

**00:** That's what most of the men does, they just buy drugs at the private pharmacies and it’s not easy to convince men to go to the hospital. For instance, during the early dates of COVID-19, my husband fell sick, he was having difficulties in breathing but he was not willing to go to the hospital and he had to go to the hospital when I report about this to his sister and we went to the private hospital and he was given an injection and he got better after that.

**F:** What kind of drugs do most of the people buy whenever they are not feeling well?

00: Most of the people usually buy Erythromycin and Panado whenever they are having Diarrhea.

00: Some people buy Doxycycline and Panado and when some people are experiencing signs of Malaria, they usually go and buy Fansidar even before testing for Malaria at the hospital.

**F:** Why do you think some people prefer to buy drugs at the pharmacy when they get sick rather than going to the hospital?

**00:** Some people they don’t consider their diseases as serious and due to the fact that most of the people get better after taking some drugs.

**00:** Some people do not want to be on the queues at the public hospitals since there are a lot of people at these hospitals.

**00:** Scarcity of the drugs at the public hospitals is also one of the contributing factors, in most cases when people go a public hospital, they are told to go and buy drugs at the pharmacy.

**F:** What are the most common drugs that the doctors recommend you to buy at the pharmacy?

**00:** Amoxicillin, Panado, Erythromycin these are the most powerful drugs

**00:** Bactrim.

00: Erythromycin,

**F:** Someone else mentioned about powerful drugs.

**00:** It was me, I was taking of the drugs such as Panado, Amoxicillin.

**F:** Why do people consider these drugs as powerful?

**00:** Whenever most of the people have cough and take Amoxicillin, they usually get better easily.

**F:** My last question, do we know drugs called Anti-biotics?

00: Amoxicillin.

**00:** Bactrim.

**F:** Are we able to buy the Anti-biotics on our own?

**00:** Yes.

**00:** These days yes but in the past, it was somehow difficult because they were not found in the grocery stores. Those people who had them in their groceries, they were usually selling them secretly.

**F:** Why do you think such drugs are now readily available such that most of the people are able to buy easily?

**00:** In the past, it was prohibited for people to buy anti-biotics at the private pharmacies and groceries and those people who were found selling them were arrested but although it was like this, those drugs were not available in the hospitals.

**00:** Those people were arrested because they didn't have adequate knowledge on prescription and they didn’t have proper storage facilities for the drugs.

**F**: Now I understand that most of the people are now aware of the disadvantages of buying drugs on their own and from the unauthorized people but they still buy drugs from those people, right?

**00**: Yes.

Why?

**00:** Most of the drugs in the pharmacies are expensive but they are able to sell the drugs in full, half and quarter doses based on the money that one has.

**00:** Another thing is that some of the drugs that are found at such places are not available at the government clinics that’s why most of the people prefer to go and buy the drugs at the private pharmacy. In addition, due to the fact that some of the drugs are expensive in the pharmacies, some people prefer to buy the drugs from the illegal dealers who usually sell such drugs at a cheap price.

**F:** Anyone with a comment?

00: Yes, the government usually has money because it collects tax from the people who are in formal employment as well as those who do businesses but the government does not prioritize in buying essentials drugs which is wrong because for country to develop, it requires people who are health. Of course, there are some people who have money are able to buy the drugs on their own but it could better if the government could have also prioritized the poor people are well so that people should be able to get essentials drugs in the public hospitals.

**F:** This marks the end of our discussion; we had a nice discussion and I would have loved if we could discuss these the whole day. I would like to thank you for being open and whatever you have said, I have learnt a lot of things. Thank you very much.

**00:** Thanks.
