# Supplementary material for: Exploration of the social determinants of diarrhoea, rotavirus vaccine uptake, and vaccine ‘fatigue’ in Ethiopia, Kenya, and Malawi
Source: PLoS One. 2025 Sep 9;20(9):e0319691. doi: 10.1371/journal.pone.0319691 (PMC12419581; doi:10.1371/journal.pone.0319691)
Supplement: S1 Data — (ZIP) [file pone.0319691.s001.zip › Supporting Information Files/KY_06FGD.docx]

**FOCUS GROUP DISCUSSION 6. MUKURU**

**10 PARTICIPANTS, 5 FEMALE& 5 MALES**

1. Can you please tell us some of the illnesses that affect children in your community?

R1-My child was afflicted with cholera, the common flu, and diarrhea.

R2-Mostly, our children are affected by diarrhea, high body temperatures, and chickenpox.

R3- My children have been affected by high body temperatures, diarrhea, and vomiting.

R4-My child is affected by a swollen face, asthma, and diarrhea.

R7-Our female children are also affected by infection, mostly by UTI.

2. Which of these illnesses do you consider to be a burden in this community? Why do you say

so?

*R 10 Diarrhea and coughing are significant concerns in our community due to the risk of contamination from burst pipes. The virus responsible for diarrhea may contaminate the water pipes, posing a threat to the health of everyone in the community. This is particularly concerning when it comes to washing groceries and consuming untreated water.*

3. If you were to rank these illnesses in order of priority, what would you rate as the top three

diseases affecting children

*R6 - Diarrhea and Cholera

R3-Common flu/cough

R2-Malaria*

4. Can you tell me the health services/facilities available in this community?

*R3 - We have Njenga Level 4 public facility.

R7 - Mandeleo public facility.

R2 - Ruben center, a private facility.

R10 - Mukuru health facility, public.

R5 - Our Lady of Nazareth, public.

R6 - Quarry Dispensary, public.*

5. How do most people respond when a child has diarrhoea in the home? [Probe: What do

people do at household level? at community level?

***At the HH level*** *R6 - Most of the time, we take a sick child to a health facility, but at times we take them to a nearby private chemist to get over-the-counter prescription.

R5 - We take the child to the chemist, and if the sickness persists, we take the child to a health facility.

R7 - We give the sic****k*** *child drugs at home, and if the sickness persists, we take him to the facility.

R4 - At public health facilities, they don't charge any fees for medication/antibiotics, but for private facilities, th****ey charge highly.

At the community****R3 - We give the diarrhea child a loaf of bread or boiled bananas to help the child.

R2 - We give the sick child yogurt.

R5 - Other community members go to the chemist and purchase ORS drugs to stop diarrhea.

R8 - We access antibiotics at the facility level, but most of the time, we buy from private pharmacies since the public facilities do not have enough stock of antibiotics****.***

6. Can you tell me some of the enablers and challenges that people experience to access

treatment for diarrhoea diseases?

***Enablers****R9-Facilities (Maendeleo and Mukuru) are easily accessible; we are able to seek professional medical advice and treatment.

R10-Linda Mama card, M-Tiba, and NHIF enable us to access medication for diarrhea for our children at public facilities at almost no cost.

R6-The availability of community health volunteers who refer us from the community to health facilities for diarrhea treatment.* ***Challenges****R4-Long queues at the health facility, and after diagnosis, you get referred to a private pharmacy to buy drugs.

R7-Public facility medical staff are at times rude to patients, and they close offices in between for lunch hours, leading to congestion. Additionally, in private facilities, the treatment of diarrhea is relatively expensive.*

*.*

7. What do people do to prevent diarrhoea? [

***At the HH Level****R3-Washings hands after visiting the toilet and anytime the child comes from outside the house.
R2-Washing fruits,vegetable and boiling drinking water.
R4-Cleaning the HH floor always.
R1- Ensuring the utensils are cleaned and dried.
R3-Cover food properly to keep away virus transmitting insects
R5-Ensure the household have proper waste disposal mechanism****.
At the community****R3-At the plot/HH we ensure that we dispose waste in designated places and not in open fields.
R5-Ensuring that we unclogg blocked trenches and having a cleaning campaign on weekly basis done by Kenya Redcross Volunteers*

8. How do people in this community perceive childhood vaccines [Probe: why do you think

childhood vaccines are widely accepted? Why do you think childhood vaccines are widely

resisted?

*R2- Some individuals resist childhood vaccines due to concerns about potential side effects..*

*R4-. There are some who refused the polio vaccine because you do not take the child to hospital rather the vaccinators come to your house,I vaccinated my children, and they have been healthy. But I understand some parents who had negative experiences or heard stories of adverse reactions might be hesitant.*

9. How about rotavirus vaccines? What do people think about rotavirus vaccines? Where do

they access rotavirus vaccine?

*R9- Community leaders and elders who understand the importance of vaccination can positively influence opinions.

R8- Parents usually get information about rotavirus vaccines from healthcare professionals during routine check-ups. However, there's room for improvement in disseminating information through community awareness campaigns to address concerns and misconceptions.

R10- Many community members believe that rotavirus vaccines are effective in preventing serious gastrointestinal infections. Parents who have vaccinated their children often mention fewer instances of severe diarrhea, hospitalization, and overall better health outcomes.

R2-All the vaccines for children are mandatory from the first one to the last one.*

*R3-Most community members are not aware of the vaccines available for children.*

*R4-We have a nearby church that has barred its members from getting the vaccine.*

*R 5-Adverse effects or doubts about the vaccine's efficacy also deter community members from getting vaccinated.*

*R9-The COVID-19 pandemic introduced additional challenges. Fears of contracting the virus deterred individuals from visiting healthcare facilities for routine vaccinations, leading to a decline in vaccine coverage within the community*

**10. What are the enablers and challenges for people in this community to access rotavirus**

**vaccines?**

***Enablers for access to Rotavirus vaccine***

*R4-Rotavirus is affordable and easily accessible at public facilities since it is free of charge.*

***Challenges***

*R6-Some communities have reservations about the rotavirus vaccine due to cultural practices or religious ideologies.*

*R8-Fears surrounding adverse effects, fueled by misinformation, discourage our community from seeking the rotavirus vaccine*.
