# Supplementary material for: Exploration of the social determinants of diarrhoea, rotavirus vaccine uptake, and vaccine ‘fatigue’ in Ethiopia, Kenya, and Malawi
Source: PLoS One. 2025 Sep 9;20(9):e0319691. doi: 10.1371/journal.pone.0319691 (PMC12419581; doi:10.1371/journal.pone.0319691)
Supplement: S1 Data — (ZIP) [file pone.0319691.s001.zip › Supporting Information Files/MW_2FGD.docx]

**F**: Let's start our discussion with diseases that are common amongst children here in Bangwe, let’s start with that. Can you tell me diseases that mostly affect children here in Bangwe?

**00:** (All) Silent

**F:** Let us start with you, what is your number?

**P 4:** P 4 Diarrhea and Pneumonia are the diseases that are common amongst the children here, some children are losing their lives due to these diseases especially when their parents does not seek medical services in good time at the clinic. In case of Diarrhea, when a child is having this disease and his/her parents goes to the hospital in good time, the child is able to recover easily but if the child is not taken to hospital, he/she can easily die.

**F:** Diarrhea and Pneumonia, apart from these diseases, what other diseases are common amongst children here?

**P 8:** Fever that is manifested through high body temperature and cough, when a child has fever and cough, we are required to go to the hospital with the child in good because these diseases can result into death if the child is not taken to the hospital in good time by his/her parents.

**F:** Now you have added cough and fever?

**P 8**: Fever and cough.

**F:** What other diseases are common among children here in Bangwe apart from these four?

**P 9:** Skin sores and chicken pox.

**F:** Are there other diseases that are common to children apart from these, now we have 5 right?

**P 5:** Vomiting, most of the young children here vomit and this them to have weak bodies.

**F:** This is also a problem among the children here in Bangwe?

**P 5:** Yes.

**F:** Are there other diseases that you are thinking about?

**P 10:** Chicken pox and skin sores.

**00:** There are also some sores that are usually attacks children’s heads and they cause loss of hair in young children.

**F:** Are these sores different from those that are found on the skin?

**00:** These are different from the skin sores because they are usually found in children’s heads and with time if they are not treated, they cause open wounds in their heads.

**F:** Are they different from ringworms?

**00:** Yes, they are different because these ones are accompanied with pus.

**00:** My child had this kind of sores and I had to go to QECH where I was given some drugs but when I used those drugs, nothing was changing and I had to go back to the hospital again and I was given different type of drugs but no to avail. I then went to the hospital for the third time where I was given an injection but that did not help as well, I would say that my child got healed naturally. Some people say that these sores are common to children below 9 years of age and that no child beyond that age can suffer from this disease.

**F:** What ages of children are prone to this disease?

**00**: Like mine is 4 years old.

**F:** Out of the diseases that you have mentioned, which ones can you say are giving you more troubles in this community?

**P 6:** Malaria.

**P 1:** Diarrhea which is common among the children who at crawling stage as well as those who are learning to walk. This is due to the fact that these children are fond of eating anything that they come across with. I would say that this disease is common among the children between 1 to 3 years.

**P 7:** When a child has fever, his/her body temperatures increases and if the child is not taken to the hospital in good time, he/she die easily.

**F:** You have mentioned Fever and Diarrhea, what other diseases do you think are commonest among the children.

**00:** Malaria and Pneumonia, in most cases when the child is not taken cared of like inability to provide the child with warm clothes cold weather, it means that the child at risk of Pneumonia.

**F:** In your opinion, what makes Diarrhea an issue here in Bangwe?

**P 6:** Here in Bangwe, Diarrhea is an issue problem because of scarcity of clean water. In this township, it is very hard to fetch clean water and due to the fact that when a child reaches 6 months of age, we usually introduce them to some hard foods including water. Sometimes children are given water from wells to drink before the water is treated and this place children at the risk of Diarrhea.

**P 1:** Just to add on this, due to scarcity of water we are forced to go and wash cloths and kitchen utensils in the rivers whose water are full of worms and this exposes children to Diarrhea.

**F:** Are you able to see these worms?

**00:** Yes, they are visible, they are usually red in color.

**00:** Apart from these, some people who have built their houses near these rivers and connects their sewer system into these rivers.

**P 7:** Some people also plant vegetable in their gardens and uses water from these rivers in watering their plants. Sometimes people throw garbage from their waste bins into these rivers and some people wash the vegetables in these rivers before selling they are sold at the market. We have some people who does not cook the vegetables properly before eating them and due to this, there are more cases of Diarrhea among both the children and adults here in Bangwe.

**F:** Like Salad?

**00:** Yes.

**P 8:** Most of the cooked food that is sold along the roads and markets is not properly covered.

**F:** Are you trying to mean that most of the people likes to buy cooked food that is found along the roads?

**00:** Yes.

**P 9:** I feel like the food that has been prepared properly cannot have any problems but when the food is not covered it is exposed to some germs and other micro-organisms that are responsible for causing Diarrhea.

**F:** You have mentioned shortage of clean water, unhygienic practices, what else do you think increases cases of Diarrhea among the people of Bangwe?

**P 2:** I would say that congestion of people is also one of the contributing factors towards unhygienic practices among the people in this community. This is due to the fact that in a congested community even when one practices hygiene and others not, it is very difficult for the diseases not to spread easily because the presence of those who does not practice hygiene.

**P 9:** Just to add, I stay at a congested compound and I always make sure that my children defecate in a pit latrine but my neighbors allow their children to practice open defecation meaning that although am practicing hygiene, me together with my children we are at the risk of Diarrhea due the other people who do not practice hygiene.

**F:** Alright, we have discussed about Diarrhea, what about in terms of Malaria, what makes Malaria an issue here?

**P 10:** Most of the people including children do not sleep under treated mosquito nets and these exposes them to mosquito bites. Some people do not have mosquito nets, at the clinic it is only pregnant women who are privileged to receive mosquito nets but it is still a challenge because some pregnant women do not attend antenatal clinic.

**F:** Shortage of the mosquito nets, what else?

**00:** In most of the communities there are stagnant waters especially from the bathrooms which acts as breeding grounds for mosquitos and some of the houses do not have proper ventilation which makes it easy for mosquitos to breed easily.

**P 4:** In addition to this, there are some people who do not cut grass surrounding their households and this makes it easy for mosquito to breed as such there is a need for people to cut grass surrounding their homes.

**F:** Let's move on to Pneumonia, I understand someone else mentioned about this disease, right?

**00:** (All) Yes.

**P 1:** Pneumonia is an issue here because Bangwe is the coldest township in here in Blantyre, so when children are exposed to cold, it is easy for them to suffer from Pneumonia.

**00:** Sometimes parents do not have money to buy warm cloths and beddings that can protect their children from the cold.

**F:** In your opinion, which time of the year is Pneumonia common in this township?

**00:** June, July.

**F:** From January to December, which months do you experience more cases of Pneumonia.

P 6: As for me it would be difficult to mention the exact months when Pneumonia cases are common among the children because as of now (September) children are still suffering from Pneumonia such that some children from this community are being referred to QECH to get better treatment for Pneumonia. This means that it is difficult to determine the months when Pneumonia cases are common among the children here. This is due to the fact that every child can suffer from Pneumonia he/she is exposed to cold weather regardless of the time of the year.

**F:** Is there something else that you think leads to more cases of Pneumonia here apart from what you have mentioned?

**P 6:** Most of the children also suffer from Pneumonia because of the food that we usually give to our children. Sometimes children are given food that is cold and this place them at the risk of Pneumonia.

**F:** The fourth disease that you mentioned was Fever, but we are going to look at it later. Now among the three diseases that you have mentioned, which one can you say is the major issue here?

**P 2:** Diarrhea.

**P 4:** Pneumonia.

**P 7:** Malaria.

**00**: Wanted to say Malaria.

**F:** Now which among these is the very common here?

**00:** Malaria.

**00:** Pneumonia.

**00:** Diarrhea

**00:** Malaria.

**F:** I just want us to agree on one disease that is very common here.

**00:** Diarrhea.

**F:** The second one?

**00:** Pneumonia.

**F:** Which is supposed to be number two between Malaria and Pneumonia?

**00:** Malaria.

**F:** Do we all agree with this?

**00:** Yes.

**F:** Now I just want us to discuss on how we access health care services, what do most of the people do when they are not feeling well do for them to get better here in Bangwe?

**P 10:** Here in Bangwe when one gets sick, it is difficult for him/her to go and get assistance at the public hospital. This is due to the fact that one can goes to the hospital, he/she is told that there are no drugs and he/she is referred to the private pharmacies to buy the drugs that the doctors and it worse when one does not have money to buy such drugs.

**F**: What else do most of the people does for them to get better apart from going to the hospital?

**P 9:** In most cases when we go to the public hospital, we found that there are no drugs even drugs such as Panado. As such in most cases when we are not feeling well, we just to straight to the pharmacies to buy the drugs. However, the problem with going straight to the pharmacies is that one can buy drugs that are contrarily to the disease that he/she is suffering from where as the hospital, doctors are able to prescribe appropriate medication after their diagnosis and I remember at some point in time I went at a certain pharmacy where I was sold drugs that were contrary to what I was suffering from.

**F:** You have said that when one has money, he/she can go to the pharmacies to buy drugs, right? What about for someone who doesn't have money?

**P 9:** Those who don't have money they don’t have a choice and they are forced to go and seek for assistance at the public hospital.

P 6: In a situation where, one is told by doctors to buy drugs at the pharmacy and he/she does not have money, there are some alternatives that people use such as use of herbs that are readily available in our communities. For instance, when one has Diarrhea, he/she just get Guava leaves, boil them into water and there after drink the water, this really helps.

**P 5:** One can also make a solution of sugar and salt.

**F:** What do you call this?

**P 5:** *Thanzi* (Oral Rehydration Salts).

**F:** At what point of sickness do most of the people go to the hospital to seek for assistance?

**P 1:** At any point when one is sick, he/she goes to the hospital but the problem is that at the public hospitals we mostly spend a lot of time like spending the whole day at the hospital only to be told that there are no drugs and they tell us to go and buy the drugs at the private pharmacies.

**F:** What modes of transport do you use to get to the hospital?

**P 9:** I just want to understand, are you trying to mean that private or public hospital?

**F:** Any hospital where you go when you are not feeling well.

**P 9:** Public hospital?

**F:** Let's start with public hospital.

**P 9:** For those who stay close to the hospital they usually walk but those stay from far they need some money for transport.

**F:** How much does it cost for someone who stays very far to come to the clinic (Bangwe health centre)?

**P 9:** One can spend approximately K 3000 to and from the clinic.

**00:** Someone from an area known as K requires about K5,000.00 to and from the clinic.

**F:** Is this the only public clinic here in Bangwe?

**00:** Yes, and someone does not come to the hospital because of lack of money.

**F:** How much does it cost for those who stays near the clinic for them to get to the clinic?

**00:** K 1000.00

**00:** Some who stay close to the clinic usually walk but those who stay a bit far from the clinic spend about K 1,000.00 to and from the clinic.

**F:** What about the private clinics, are there differences on the amount of money that is spend to get to the private and public clinic?

P 3: At the public hospital we are only required to buy health passport at K300.00 while at private hospital clinic such as Hajra you have to pay K 2,500 as consultation fee and they usually charge based on the disease that you are suffering from. In addition, for one to get an injection at a private clinic like Hajra, it requires an additional money. I remember at some point when I went to Hajra private clinic and I was charged K 2,500 as the consultation fee, K7,500 for a drip of glucose and K 5000 for drugs but nothing was changing in me so I decided to go QECH where I was only required to buy health passport at K 300 and I was given the same treatment for free.

**F:** Between private and public hospital, where do most people go when they are not feeling well?

**P 3:** In most cases, a lot of people go to public hospital.

**00:** Most people are forced to go to the private hospital because for one to get medicine at this hospital (Bangwe) it means that you have to be known by someone else who works at the hospital while at the private hospital it depends on the money that one has.

**F:** This one is claiming that a lot of people here usually go to private clinics as compared to public hospitals, P 10 what is your stand on this?

**P 10:** Am against the idea that most of the people are now preferring private clinics. Regardless the fact that the help that most of the people get at the public hospitals is inadequate, most of the people are still opting to go there and not private clinics. If you can go to the public hospital, you are going to find long queues and this is due to the fact that most of the people here are poor and they cannot manage to and pay at the private clinic. I can conclude that a lot of people goes to the public hospitals but they are not given enough assistance at these public hospitals.

**F:** What difference is there between those who seek assistance at the private clinic as well as those who seek assistance at the public hospital in terms of economic status?

**P 10:** The difference is that there are some who believe that the only hospitals where they can get proper assistance are those owned by the government and not the private regardless the fact that they can manage to go and pay at the private hospital but there are also some usually go to the private clinics because they have a lot of money.

**P 7:** Some people who don’t have money they don't have a choice to go to a private clinic hence they prefer to go to the public hospital.

**F:** This side you have been quiet, we can't hear your voice (laugh).

**00:** What is being said here is true, in most public hospitals’ drugs are somehow scarce hence some people just go straight and buy drugs at the private pharmacies.

**F:** Where else do people buy drugs apart from the pharmacies?

**00:** Groceries.

**F:** Are they special groceries?

**00:** Any other grocery.

**00:** Some of the pharmacies do sell drugs at a retail price as well.

**00:** Some people are given drugs at the public health centers because of the relationship that they have with the healthcare workers who works there.

**F:** Sorry for interruption, how do you know that this one has received drugs at the public hospital because of the relationship that he/she has with the health care workers?

**00:** We usually know about this because when are coming out of the hospital on our way to our respective homes we are sometimes together with some people who sometimes reveal to us that they got the drugs at the clinic because they know someone else there..

**00:** I remember at some point in time I was not feeling well and I went to the clinic and while there, I met someone I know who took me to the clinician and he told him that I am one of his relatives so I should be assisted quickly and I remember that that day I was given special treatment and I was given a referral letter for me to go QECH within a short period of time which normally the case at the clinic.

**F:** What other drugs are you able to buy at the pharmacy apart from Panado?

**P 1:** Since we already know that when one has Diarrhea, he/she needs to take Fragile, this means that when one has Diarrhea, he/she go and buys Fragile at the pharmacy or we buy the drugs that are green in color because they are also used to treat Diarrhea.

**F:** Fragile, green green (laugh).

P 9: Amoxycillin, Bactrim, whenever the child is coughing, we usually buy amoxycillin. Sometimes the drugs that are prescribed at the clinic are not found in the pharmacies and in such situations like these, we are recommended to buy drugs with similar use by the pharmacy owners.

**F:** Do we go to buy drugs at the pharmacies before getting prescription from the doctors?

**00:** Yes.

**F:** Can you tell me examples of the drugs that you usually buy on your own without getting prescription from the doctors especially when you are having Diarrhea, Pneumonia or cough?

**00:** If it's Diarrhea we usually buy Magnesium because it is used to cure Diarrhea,

**P 10:** Doxycycline.

00: We also Buy Thanzi when the baby has Diarrhea unlike going to the hospital with a child who is having Diarrhea.

**00:** Diclofenac, I gave birth to this child am carrying through Caeser and I was therefore instructed to buy Diclofenac at QECH and when I went to QECH I was told to go and buy the drug at the pharmacies and I bought it at K 5000.

**00:** We also buy Indocin when we are experiencing some pains in the arms or joints.

**F:** In your opinion, what do you think are the factors that influences people to buy drugs on their own without getting prescription from the doctors?

**P 10:** What makes most of the people to go straight to the pharmacies is that some people have experiences of some diseases such that they are aware that when am suffering from this disease, I should buy this drug and they only go to the hospital if they are no changes after taking the drug.

**P 1:** What makes most of us not get prescription from the doctors is that we are used to believe that most public hospitals do not have drugs and we know that even if we go to the public hospitals, we will not be given the drugs. If the public hospitals could be having drugs readily available throughout the year, we could have been going to the public hospitals and not the pharmacies.

**F:** I just want to hear from you about the income generating activities that most of the people engage into here for them to find money to be used for them to get to the hospital.

**00:** Some people do piece works such as washing in our people’s houses while some get loans from their friends.

**F:** Apart from these, other people do what?

**00:** That's all.

**F:** Only these?

**00:** Yes

**F:** Do you think there is any advantage or disadvantage of buying drugs on your own without getting prescription from the doctors?

**00:** There is no advantage of this because buying drugs on your own can make one buy drugs that are contrary he/she is suffering from.

**P 6:** Buying drugs on your own is very dangerous because in some cases one can buy drugs that are expired at the pharmacies and here in Bangwe we are forced to buy drugs at the pharmacy based on the conditions and treatment that we are given at Bangwe health centre.

**F:** Alright, you have only talked about the disadvantages, don't you think there are some advantages of this?

**00**: This has its own disadvantages (laugh).

F: Then why have you focused on the disadvantages only?

**00**: You only asked about the disadvantages.

**F:** No, I asked about both advantages and disadvantages.

**00:** There are some advantages of buying drugs on your own. For instance, at some point in time I was having stomachache and I went to the hospital where I was given some drugs but they were not helping and then I went to the pharmacy where I bought different drugs and these ones worked. So, I would say that the goodness of buying drugs on your own is that in most cases when you go to the public hospitals you are not assisted, you just waste your time but when you buy drugs on your own you are able to get better.

**F:** Now let us move back to the topic that we were discussing about Diarrhea; we have talked a lot about factors that contribute to the increase in cases of Diarrhea in this community. Now I just want us to discuss on ways of preventing Diarrhea, what do you do to prevent Diarrhea? I would like to know what you do at home and community to prevent Diarrhea.

**P 8:** We are supposed to practice hygiene in our households like after changing a baby diaper, we need to wash our hands and before cooking vegetables we need to wash them properly with clean water.

**P 7:** We need to make sure that our pit latrines are cleaned with Chlorine so as to avoid breeding of flies that acts as vectors of Diarrhea.

F: Do we clean our pit latrines with Chlorine?

**00:** Yes.

**P 9:** I feel like only few people are able to use Chlorine because not everyone can manage to get Chlorine.

**F:** Where you get Chlorine?

**00:** Let those who said that they use Chlorine answer this question.

**00:** We get it at Bangwe health center.

**00:** We even get it from the HSAs who move around our communities.

**P 1:** I would like to ask the government to provide us with Chlorine through the year not only when our area is hit with Cholera. This is due to the fact that here Diarrhea is experienced throughout the year while Cholera is somehow seasonal. This will help us to treat water that we fetch from the rivers and wells.

**F:** Alright, that's how you can prevent Diarrhea at home, right?

**00:** Yes.

**F:** What about the whole community, how does Bangwe as a community prevent Diarrhea? what do people in the communities do to reduces cases of Diarrhea?

**00:** Like during the time when this area was recording highest numbers of Cholera cases, the government was sending a water tank truck to supply clean water to people so as to prevent people from using unsafe water from the wells and rivers.

**F:** So, what happened when Cholera cases cease to exist?

**00:** The water tank truck is no longer coming.

**00:** Most of the people here uses water from the wells and rivers because it is expensive to get tap water. Here one is required to pay about K 150 for a bucket of 20 liters and the same bucket cost K50 at the well so a lot of people opt to fetch water from the wells and not taps.

**F:** Alright let us move on to the last topic of our discussion about vaccine, what do most of the people from this community say about vaccines for children?

**00:** About what.

**F:** Vaccines for children.

P 10: In terms of vaccines for children, a lot of people are afraid of getting their children vaccinated because there are a lot of vaccines that are given to children and more vaccines are still coming. People say a lot of things about these vaccines they don’t the use of these vaccines. To but my understanding, vaccines are meant to help children’s in boosting immune system so as to prevent them from suffering from different diseases as well as the spread of the virus from one person to another. Some of the HSAs who administer vaccines for children in the communities verbally and sometimes physically abused. So, I would say that people speak a lot of things about the vaccines. For instance, when COVID-19 vaccine came, people were saying that the vaccine is associated with satanism and that those who got vaccinated will have their bodies deformed in later years.

**P 1:** We started healing about Cholera some years ago but there was no vaccine for Cholera and we used to believe that we usually have cholera cases during rainy season but last year we had Cholera cases in hot season and this made a lot of people to be suspicious and a lot of people were saying that Cholera has no vaccine and that the only medication for Cholera is a drip of water that is provided at the hospital. Now a lot of people say that the vaccine is used as a tool of spreading Cholera and not to boost one's immune system against Cholera.

**P 6:** Its true that as of now we have different types of vaccine especially for children, in the past we used to know that a child was supposed to be given two vaccines but as of before the child reaches 5 years of age, he/she is already subjected to 15 different types of vaccines. For instance, this year only children below 5 years have been given 6 different types of vaccines. This makes most of the parents to be suspicious since in the past this was not happening and it is true that sometimes we verbally and physically abuse the health care workers in the community because they just come in our homes without telling us that they are coming as well as the use of the vaccine that they want to administer to our children. I remember some months ago one of the health care workers came to my house with Polio vaccine and I lied to him that my children got vaccinated already because a month before his coming, some people also brough another vaccine.

**F:** What do you think would happen to children if they are exposed to different types of vaccine?

**P 10:** At some point we got a message from Bangwe primary that there is a vaccine that is supposed to be administered to girls below 15 years of age and this made us to be suspicious and most of the parents were angry since there were speculations that the vaccine was meant to cause sterility in these children at a later stage and therefore the vaccine was not administered to children at the school.

**P 2:** I just want to emphasize that it could be better for the government to conduct community awareness campaigns before administering vaccines in the communities so that people should know the importance of the vaccines. So, it could be good for the government to conduct the awareness campaigns before the vaccine is administered in the communities.

**F:** Apart from this, what do you think should be done to ensure that people have a better understanding of the vaccine?

**P 1:** There is a need to disseminate these messages on the radios, TV stations but the problem is that some of the people do not have access to the TVs and Radios hence it could be better for the government to conduct community awareness campaigns at community level so that even those who don’t have access to the radios should get the information.

**F:** Alright, we were discussing about vaccines in general, what about Rota virus vaccine? what are people saying about this vaccine?

**P 10:** In terms of Rota virus vaccine, I have never heard anything bad about this vaccine.

**F:** Do people from this community regard this vaccine as useful?

**P 10:** Yes of course. For instance, there is this other girl, she went to the hospital and she got K7500 after her child got vaccinated, that’s the goodness of this vaccine that am aware of.

**F:** I want to hear from others, what do people say as the goodness of Rota virus vaccine?

**P 9:** I feel like this vaccine is doing great job but we don't know much about it because we were not provided with any information regarding this vaccine before it was administered. My child got vaccinated but I don't know the importance of the vaccine in her body.

**F:** So can we say this vaccine has never cause any harmful side effects to the children who got vaccinated?

**00:** Yes.

**F:** Can we say each one of you went to get her children vaccinated when Rota vaccine was introduced?

**00:** Some did while others no.

**F:** What other factors discourages women from getting their children vaccinated?

**P 6:** Not anyone is willing to get his/her children vaccinated because we have different beliefs, as for me am a member of Apostolic church and our church does not allow us to get vaccinated so I can never allow my children to get vaccinated even if after being approached by the health care workers.

**F:** Sorry I will ask you this since you are a member of Apostolic church, regardless of your faith, do you think there is something that can be done that can make you accept vaccines?

**P 6:** I don’t think that there is anything else that can make us change what our faith requires us to do.

**F:** I just want to know, are there any other strategies that you think the government can employ to make you accept to go to the hospital to seek for medical help?

P 6: Maybe if the government reduce the vaccines that are given to children like how it used to be in the past when children were getting 2 vaccines but as it is now, it is impossible for us to allow our children to get these vaccines. For instance, only this September only, children from this community have been given a vaccine and they are expected to receive another vaccine before the end of this month that's what discourages us from allowing our children to get vaccinated.

**F:** According to your religion, the presence of a lot of vaccines means what?

**P 6:** It’s a sign of 666.

**F:** Alright, meaning that there are some beliefs that prevents people from getting vaccines, right?

**00**: Yes.

**F:** What other factors?

**00:** (All) Silent.

**F:** You have said that long distances to hospital is one of the factors that prevents people from going to the hospital. Do you think this also prevents people from getting vaccinated?

**00:** Distance is not something that can prevent one from getting his/her children vaccinated because most of the vaccines are administered to people in their respective communities.

**00:** There is a big problem because the health care workers just approach you at your household with the vaccines without your knowledge that they are coming as well as the function of the vaccines and due to the fact that you were not expecting them at your house you just lie to them that your children got vaccinated already.

**F:** In your opinion, what do you think can be the best approach to be used by the HSAs in approaching you with the vaccine?

**P 6:** My advice to the HSAs is that before administering vaccines in our communities, they should collaborate with the community leaders so that the community leaders can bring the messages to people within their areas of jurisdiction meaning that by the time the HSAs are bringing the vaccines in our household we should be aware of that already.

**F:** Is that anything else you want to talk about that I didn't manage to ask?

**00:** (All) Silent.

**F:** Because I have asked all the questions that I prepared to ask you but maybe you might have something else that you want to say?

**00:** I want your help, I understand that different studies are being introduced. There are some people who come in the hospitals to diagnose ladies for cervical cancer so they tell you that after the examination we are going to give you money. So after the examine you for cervical cancer they did not pay you contrary to what they promised you, what should one do in such situation.

**F:** Is that happening at the clinic?

**00:** Yes.

**F:** So, before you participate in the study, they tell you that they will pay you?

**00:** Yes, they usually tell you that they will pay you some money for taking your time and it happens that in the end after the discussion and everything, they tell you that we are going to send the money through mobile banking platforms.

F: First of all I would say that the researchers when they are departing their offices to meet the participants they usually take money with them. it is impossible for a researcher to leave their offices without carrying money with them so before you provide the consent, they should clarify to you that you are going to receive the money later.

**00:** I mean in a situation where you have already signed the reimbursement form and they are telling you that you will get your money later.

**F:** I know that that happened to you but I just want explain the procedure that is normally followed in research, like since we started our discussion, did I ask anyone to sign the reimbursement form?

**00:** (All) Silent.

F: So, there is no need for you to sign the payment form before you are given money and if they promise to give later, you need to get contact details of their employers. These forms contain contact details of some people who are not my relatives but my employers, you have freedom to call these people and inform where you feel your rights have been infringed, you should have interest to know the exact places where they are coming from and sometimes it is good to ask about them about the ID of the person conducting the study. It is your responsibility to ask about the details of the person conducting the study. Thank you very much
