# Supplementary material for: Exploration of the social determinants of diarrhoea, rotavirus vaccine uptake, and vaccine ‘fatigue’ in Ethiopia, Kenya, and Malawi
Source: PLoS One. 2025 Sep 9;20(9):e0319691. doi: 10.1371/journal.pone.0319691 (PMC12419581; doi:10.1371/journal.pone.0319691)
Supplement: S1 Data — (ZIP) [file pone.0319691.s001.zip › Supporting Information Files/MW_10FGD.docx]

**Facilitator:** thank you ladies and gentlemen for your participation in this discussion, thank you very much. There are several issues that I would like us to discuss and one of them being the health problems that affect children here in Bangwe. Let’s start from there, what are these health problems that affect children here in Bangwe?

**05:** malaria is affecting both children and adults because of not sleeping under the bed nets. It’s a big problem because not everyone receives a net. For example, health workers may come to distribute the nets when I am at work and there is no one at home, which means I have missed it. Sometimes people think sleeping under the net is a burden, and that’s why, malaria is common here. My child has been sick with malaria twice. The first time, he/she fainted. He/she was admitted to Queens for 5 days after 2 weeks, it happened again, so I bought a mosquito net for him/herself

**Facilitator:** on that point, you said people don’t usually sleep under the net, what did you say is the reason behind it?

**05:** people say they feel hot when they sleep under the net. Some people say and others here can agree with me. If you have slept on a mat, mattress or the bed, if you have not hung the net properly on the edge, it will burn you when getting up the following morning, such reasons make people not sleep under the nets

**02:** A health problem that is common here in Bangwe from 15 years ago, is diarrhoea. This is for both children and adults because people use unprotected water. Another reason is that the foods that we eat are not protected. On top of that, he mentioned malaria, is one of the diseases that are common here in Bangwe for both children and adults because we are promised bed nets, people come into our homes to record our ID numbers with the purpose of giving us the bed nets. Time goes by, without us receiving the bed nets. This is the summer season and there is too much mosquito breeding, there is no water, and there are more stagnant waters. Secondly, even though we say that we are sleeping under the nets. When some people sleep under the net, they have difficulty breathing, and they feel like they have slept in a pot and there is no ventilation, to them it’s not good to sleep under the net, and after all, mosquitos' food is humans, that’s why malaria is so common, I should stop here

**07:** diarrhoea is common here in Bangwe as he has already said. Our water is not protected and it can take us 2 weeks without piped water, so we use water from the wells and rivers, so diarrhoea is common here and in the end, flue comes, and the patients are referred to Queens where they are diagnosed with typhoid and after receiving the treatment, they get cured.

**04:** health problems that affect children are flu and cough, as a result of congestion in schools. Because children are overcrowded in schools, they can catch the flu. When they get back home, you may administer the treatment and the child gets better. When he/she gets back to school, the child contacts a classmate who has flu as well, and he/she will transmit the flu. You may be administering the medication without an improvement, and then the child starts having difficulty breathing, so you stop sending the child to school for a while

**Facilitator:** they transmit because they are over-crowded

**04:** yes

**Facilitator:** what causes flu here in Bangwe?

**05:** what happens with flue is that when parents are away like this, they leave children alone playing with dust, so these things cause flue to children. So, after playing, they sleep without taking a bath, and all the dust remains in the blanket when they use that blanket the following night, they swallow the dust, that’s what normally happens to children

**Facilitator:** you have talked of diarrhoea as one of the health problems here in Bangwe, you have said it is caused by the unsafe water from the river. Did you say both water from the rivers and boreholes?

**All:** yes

**Facilitator:** how unsafe is the water?

**07:** our rivers are also the bins as well, so we have unsafe water. Most of the bins are closer to the boreholes, it is not safe because one borehole is been used by many people and everyone aims at getting water quickly, so they don’t wash the utensils, and the boreholes are not for free, people pay. So we use unsafe water

**04:** in addition, we have a borehole down here, and up there is a toilet. Underneath waters are mixing, and in the end, we have unsafe water

**Facilitator:** number three, we have talked of flu, malaria and diarrhoea, are there other health problems that affect children here?

**03:** no, it’s malaria and diarrhoea, when they go to the hospital, we are given only panado. The problem is what my colleagues said about unsafe water, we don’t have safe water here in Bangwe. We are using rivers which are dumping areas too, people dump papers in the rivers, others are drawing water for bathing, because of lack of water

**Facilitator:** people do know that there is a water problem and the only water source they depend on is a river, why do they dump wastes in the rivers here in Bangwe?

**08:** lack of understanding by people living in Bangwe. So what people do, when they have waste, is to hire a manual worker ‘’dump these ones.’’ Because it is piecework, he/she just dumps the waste anyhow. If it was the previous time we had metal bins here, the city council was taking them out when they were full, and that was better. We don’t have such bins here in Namatapa, so people just dump waste anyhow, they say should I get there, it’s too far, so they dump them under a bridge at night so that when it rains, they can be washed away but where the wastes go, there is a well, and the following morning, a child draws water for him to bath and go to school, there is no hygiene practice obvious. Pampers are too much here in Bangwe. People are dumping them in rivers and if a dog picks them it will dump them on the veranda. Because of busy, you just put them behind the house so that when you are free, you just dump them near the well. If a visitor does not know where to dump pampers and has seen someone dumping them under the bridge, will he/she not do the same? We should have counselling for us to understand the disadvantages of dumping trash everywhere, it contributes to diarrhoea

**02:** just to add what he has said. What I discovered here in Bangwe is that most of the people here in Bangwe have no garbage dump despite staying in rented houses. These pits help because if the pit is full, we burn it, so we reduce future problems. We are dumping trash in the rivers instead. If we pass by the rivers, there is a bad smell, and we cannot eat food, that’s not possible. In addition to the diseases that affect children here, most of the children here have ringworms. So, you are here for this discussion, we will see how you are going to help

**00:** I suspect that it’s because of the animals that we keep like dogs. I was reading somewhere that if you are keeping animals and you are not cleaning them, they can transmit diseases to a child. We thought it was a dog, but we take it to the dip at all times, so I think it’s just an issue because it’s one after another

**06:** in the recent past, there was a type of fish that was coming from Mozambique called Makerere, people were blaming that fish but, it’s no longer coming

**Facilitator:** what people were saying regarding that type of fish?

**06:** it has scales of course but, it has fewer bones, people blamed the fish for causing this illness, the fish is not available, but we still have the disease

**Facilitator:** Okay, were people blaming the fish for the ringworms?

**06:** yes, it starts like skin rash, then it affects the whole skinhead, looking like ashes and it affects the hair

**00:** it produces pus

**Facilitator:** ooh

**0:** that depends on the body

**Facilitator:** I think every disease is a health problem

**All:** mmh (yes)

**Facilitator:** of all the diseases that you have mentioned, what are the most serious health problems?

**01:** the most serious health problem here in Bangwe is diarrhoea because of the erratic water supply as well as the use of unsafe water from the rivers. A dead dog may be dumped in the river yet people are using the same water and as a result, we get sick with diarrhoea. So the most serious problem here is diarrhoea because of unsafe water

**Facilitator:** if you are to select 3 serious health conditions from what you have mentioned, what could be these three?

**00:** the first one would be diarrhoea

**Facilitator:** do you all agree?

**All:** yes

**00:** the second one would be malaria

**Facilitator:** do you all agree?

**All:** mmh (yes)

**0** third one would be flue

**Facilitator:** flue?

**0:** we can say they are flue, diarrhoea and malaria. Malaria drugs are not available at the hospital, you go to the hospital in the morning, and by noon malaria drugs are finished. The following morning same things happen. It’s because many people go to the hospital to get flue, diarrhoea and malaria drugs

**Facilitator:** when someone gets sick here in Bangwe, what do they do to get treatment?

**06:** if you want to access quality treatment, go to the private clinic. If you go to the public health facility, they will just prescribe and tell you to buy the medication

**02:** we are discussing so let’s be open

**Facilitator:** yes, let’s be open

**02:** previously, that clinic was good, but the staff that is working there now is diverting the drugs and selling them in private clinics. When we get sick and go there, what they have is panado and Bactrim, and that panado is not even enough, you are given maybe 4 tablets on paper, the rest you will buy. For someone whose survival is hard, it is difficult for them to buy enough drugs because even though they have prescribed the treatment for you to buy, what are you going to buy with if you don’t have cash? That’s why people here in Bangwe suffer from different diseases

**Facilitator:** you have said that for you to get quality treatment, you go to a private hospital

**06:** yes

**Facilitator:** when you say ‘’quality treatment’’ what do you mean?

**06:** it is a treatment suitable for the illness. If you have a headache and are given treatment which is not for the headache, are you going to recover? You cannot, so if you want to get quality treatment, go to the private clinic. If you go to the public facility, they will prescribe the treatment for you to buy, yet you have no money. So they give you 4 tablets of Panado for morning and evening, what about the following day? Are you going to be okay? That’s not possible, and the treatment is not enough, they tell you to buy other drugs.

**00:** I am very bitter with this issue of drugs, if this discussion was aired on the radio, they could have heard what I am saying. The day my child fainted, I was working. My wife called me at around 2 or 3, ‘’we are referred to Queens! Our child is unwell and still unconscious!’’ I asked her, ‘’what did they say?’’ ‘’We should find our means of transport,’’ I told her to hire a motorbike and get me in town and she came. We went to QECH, I was disappointed with what happened, but I was so happy with the warm welcome doctors from Queens gave us. About 6 doctors cared for our child, someone did this, another one did that, and someone collected blood samples, in a short period of time, everyone was coming up with results and records, that’s important. What happens at this facility is that when you go like today Saturday, you will find that they haven’t started working. As we are getting in, before sitting, they have already started asking, ‘’What’s wrong with you?’’ before I explain, they have already started writing. You have no time for questions, they have a target, and they just want to knock off, maybe they do business as well, they sell second-hand clothes, so they want to rush to the business

**All:** laughing

**00:** so, they rush to do some things. I have got evidence, but I cannot mention a name. Some doctors live with us in the communities. Family planning methods are obtained from the clinic, some have family planning drugs, and they go out to the community to inject women in return for 1000

**Facilitator:** the facility one

**00:** that one, and you know this is a healthcare worker from this facility, my wife once did it, got injected and paid 1000. When the women go to the clinic, they don’t get the family planning injections

**06:** I went to the private clinic when I got sick. I was told that we have no medication for this condition, ‘’if you have 5000, give us.’’ I gave paid 5000. I was disappointed to see that doctor sending his boy, after contacting a health worker from the public clinic. The boy went by the motorbike, he just took the things and came back. When the drugs are delivered at the hospital, what should someone do, we don’t know what someone can do to receive the right treatment. Doctors work in shifts, there are day and night shifts. I have been there with a patient and was told to come tomorrow morning, what is their work yet they are deployed on the night shift? I called my friend, ‘’this is what has happened.’’ ‘’aah it's okay, tell a doctor that you want to call for a councillor to come and sort it out.’’ I put that call on the louder speak, and I asked a friend, ‘’Do you have a contact number for the councillor?’’ when a doctor overheard that, he was sleeping, but he woke up, ‘’What’s really wrong with a patient?’’ he started treating the patient because he had heard that the issue was going to be reported to the higher authority, so what is needed is the use of power, otherwise, you cannot receive the treatment.

**07:** I have got evidence on this issue of drugs, things are bad. Drugs from the Namatapa clinic are available in private clinics. When you go there, they just prescribe the treatment without giving you the drugs. I went there just yesterday with my children, they just prescribed the treatment without giving us drugs, just diarrhoea drugs. The child was given thanzi only, the child I have registered here, ‘’Give him/her 2 litres of than for the whole and the following day, ‘’What about other drugs?’’ ‘’We don’t have drugs, buy them if you want.’’ I didn’t have money, how could I buy the drugs

**Facilitator:** how did you feel after receiving thanzi only?

**07:** it was painful to me, I felt not assisted, and that’s why I rushed to come here

**All:** laughing

**07:** so that my child’s health should improve

**Facilitator:** did you do anything when you got back home?

**07:** I did not do anything because I had no money

**Facilitator:** what would you have done if you had money?

**07:** I would have gone to the private

**00:** or else he would have bought the drugs

**Facilitator:** if you had money, what kind of drugs would you buy for your child?

**07:** I would have gone to the private clinic for them to see what was prescribed and also a further examination of a child if she/he has other illnesses. I could not just buy drugs because I could buy expired drugs

**Facilitator:** if drugs are not expired, what drugs could you have bought?

**00:** Okay, when we take a child to the hospital, they do their work. They give you insufficient drugs, then they tell you to buy the rest. If we have money, we buy what’s on the prescription, we can go to the pharmacy and buy

**0:** most of the time when they have prescribed the treatment, you just go to a pharmacy and show them a card because most of the time, what the doctors write can never be seen by an ordinary person. When you show the pharmacists, they too are the doctors, and sometimes you just explain to them, that they give you the right treatment, so when they see a book, they know what treatment to give

**Facilitator:** is it at all times when people go to the hospital first before going to the pharmacy?

**00:** most of the time we use Flagyl when we have diarrhoea or green-green, they are available in shops

**0:** doxycycline

**00:** they are green capsules, so we use Flagyl and these drugs when someone has diarrhoea, you buy thanzi and Flagyl, it works

**Facilitator:** you have mentioned green-green and doxycycline, is it the same drugs?

**00:** yes, green-green is doxycycline

**FP1:** if my child has diarrhoea and I don’t have thanzi, I make sure I have a zinc tablet

**Facilitator:** where do you buy them?

**FP1:** we buy them from the pharmacy

**Facilitator:** what is the use of zinc?

**FP1:** if a child has an infection in the stomach, it helps. Just to add to what happens at the health facility, the problem is that they don’t give you a chance to explain. After the explanation, they say that they have diagnosed you with this problem, so they don’t give you the chance to ask what to do for you to not experience the same condition, they just tell you to go to the pharmacy and receive the drugs. So, they don’t give you time to ask, for example, it’s an infection, they don’t tell you how it is caused, they just prescribe and tell you to go. When you go to the pharmacy, they just tell you, ‘’This is for morning, noon and afternoon, ‘’ sometimes they give you insufficient dosage

**Facilitator:** number 2, I interrupted you

**02:** I would like to ask you a question and this question came to me in a tricky way

**Facilitator:** Okay

**02:** you asked how we get treatment when we get sick

**Facilitator:** mmh

**02:** at some point, you asked how we are assisted, and everyone responded how they understood the question. I am getting back to the question. In order for me to prevent that problem, I should have several prevention methods. We are talking about diarrhoea, it is unsafe water that causes diarrhoea. We are told to boil water from the river, borehole or from the well if we are not sure, and when you boil the water and follow the process of sieving, you are assured that the water you are drinking is safe. Secondly, the food that we eat. You know there are house flies out there and you are buying child flitters that have been uncovered since morning, let us be honest, can this not cause diarrhoea

**FP1:** it can cause

**02:** for us to prevent diarrhoea, we should eat safe foods. When you are late in the morning, you just eat food that remained yesterday, yet you don’t know what spoiled the food. First of all, we should prevent problems before they come, and when the problem comes, that’s when we go to the hospital and I cannot comment on it, I already put the blank

**All:** laughing

**02:** when things are not alright, I just go to the clinic to avoid the disappointment, ‘’iih we don’t have medication for this illness.’’

**Facilitator:** which clinic do you go to?

**02:** there is a private clinic at Tadala, some are in Namiyango. When you go there, ‘’iih just pain killers,’’ yet I have gone there to receive the treatment for my child and you are giving me tablets to relieve pain, after the pain is been relieved, the illness will resume. All I am saying is that we should be preventing the problem first before it comes, and when we have a problem, that’s when we rush to the hospital, that’s what I wanted to add

**FP1:** he explained about the food

**Facilitator:** mmh

**FP1:** you give a child food, like flitters and after a short period of time, you give him/her another food. As a result, a child eats a mixture of food that can affect the child’s stomach. It’s only that we have got poor financial statuses that’s why we pick this and that but, we should be monitoring the foods that we are giving to our children, we can then prevent the illnesses because if we just mix the foods, some may not be suitable for the child’s healthy

**Facilitator:** on prevention, he talked of boiling the water, how else do you prevent diarrhoea at home on top of what is been said?

**04:** we should start practising hygiene, just like what others have said. You know that I use this basin for washing or bathing, you should not use the same basis for washing the plates. So it happens that the same basin is used for bathing, the same one is used to prepare the food and the result, we cannot prevent the illness. Children do not know how to wash their hands, they just pick food and start eating. So, you need to call the child and wash his/her hands

**Facilitator:** is there any other prevention that you know? Number 3…..number one is silent

**03:** Another prevention is to make sure you have a rubbish dump so that when you have used things like diapers, instead of dumping them in the rivers, you should dump them in that rubbish dump and in so doing you prevent illnesses. If you have a rubbish dump, you will get petrol, paraffin or diesel for burning the rubbish when they are dry, we can then prevent diseases

**Facilitator:** we have been talking about this, do you have a rubbish dump here in Bangwe such that we can burn when they are full? Does this happen?

**06:** it is difficult here in town because the landlords have no space for that, all they have are spaces for building houses as per their business. We just have an opportunity to have a trash sack and that trash is removed by a manual worker

**00:** as said, it is removed at night, and it is dumped everywhere on someone’s veranda

**All:** laughing

**06:** the houses are for business, so the space they keep is for the toilet and bathroom, that’s all, not space for the rubbish dump

**04:** with the modern housing styles, they just plaster the whole space without leaving a space for a rubbish dump, in the end, they are the ones who come out of the fence and dump the trash outside because they did not leave the space for the rubbish dump

**07:** when they have finished washing their plates, they dig a hole along the fence and water goes to someone’s plot outside, all the trash goes out there and all the dirtiness happens outside there

**00:** (those outside the fence are affected

**Facilitator:** Alright, when a child gets sick, apart from buying medication or rushing to the hospital, is there anything that we do back home apart from these?

**02:** we call it first aid, the hospital is too far and you don’t have money. We take sugar and salt to make thanzi, so we use a teaspoon for measurement. Then we administer to a child up until he/she improves, beyond that, that’s when you take a child to the hospital

**07:** We have friends who are herbalists, they use aloe vera to stop diarrhoea. If a child is constipated, aloe vera helps, that’s what our friends say. People have aloe vela in their homes and it’s working sufficiently

**Facilitator:** how do you use aloe vera?

**07:** Cutting the pieces and adding water, then start administering to a child, so most people do that because they don’t get medication at the hospital, when they do that, the following morning they say, ‘’Should I go to Limbe (health centre)?

**0:** another thing that people do before going to the hospital is they take guava leaves and add water, then wait for some for 10 minutes, then drink the water, and diarrhoea stops instantly

**Facilitator:** ooh, instantly?

**0:** yes

**Facilitator:** fresh leaves

**0:** yes, put them in water for 10 minutes, diarrhoea stops

**00:** if guava leaves fail, use flour, just add water and stir a bit, then drink it, it stops as well

**Facilitator:** you take guava leaves…..

**00:** in the absence of guava leaves and you have got flour

**Facilitator:** which flour?

**00:** the one used for baking flitters

**Facilitator:** Okay, did you all know these?

**All:** laughing (cross-talk)

**06:** I knew peaches leaves, if someone has malaria, take peaches leaves and mix water, then administer them to a patient, malaria cures

**Facilitator:** how long does it take for someone to get healed?

**06:** you boil the mixture and put the liquid in a bottle, and when the time comes for a patient to drink, he/she does so, within 2 days malaria disappears

**02:** I have a question but I would like to add on the malaria treatment, some people were talking about grinding pawpaw leaves and water, don’t boil them. I am a witness, I have a friend who has never gone to the hospital, he just uses pawpaw leaves and within 2 days, he gets better. My question goes to number 7, we are talking of the under-5 diarrhoea and you have talked of aloe vera. Can we administer aloe vera to a 6-month-old baby?

**07:** it’s not necessary

**00:** the child is too little for that medication

**07:** we are talking of the under-5 children, even a one-year-old cannot drink aloe vela because when adults are drinking aloe vela, they have sugar behind, so children cannot drink aloe vela

**0:** a child cannot drink aloe vela, we just hear people talking, so the advantage of going to the hospital is that they prescribe the dosage according to the illness and patient's age, you will never hear them ‘’Ooh let’s try this and that’’, he asked how long does it take for guava leaves to heal, you have answered 2 days yet you supposed to start improving within 3 hours, it depends on the dosage, we just heard these things and we make illness to continue because we administer the doses that do not suit the patient age, so we either worsen the condition or improve the condition

**Facilitator:** we are discussing whatever you do whether they are wrong or not, and I have already said earlier, what we do. Let’s get back to the issue of hospitals, there are several things that were raised as barriers to accessing health services—drug shortage, how people are welcomed and the long distances. I would like to ask if there are other barriers apart from these.

**02:** beliefs hinder people from accessing medical services. There are some religious groups that I cannot mention the names, they don’t believe in going to hospitals

**Facilitator:** mentioning them is not gossiping but for us to have a picture

**02:** Alright, churches like Jehovah’s Witness, if there is a member here please you will forgive me. There are Apostles, who don’t go to the hospital, then there is Zion church, members don’t go to the church, we stay with these people in our communities, is there any other church? Remind me, please?

**00:** there is a modern church movement, that tells their members that if they pray, God will answer them instantly, just like what the Universal church does, they say give whatever you have, and God will bless you. So that’s what they do, they say don’t go to the hospital, let’s pray now, and God will heal this child right now because if you go to the hospital, you will meet a Satanic doctor who will finish your child, that’s what people are doing and one of them is my wife, I force her to go to the hospital because of the same issue of religious beliefs. I try as a father to pray for my child, but it does not work

**All:** laughing (cross-talk)

**00:** eeh because I know it’s my money, when I go working, he should be eating the tithe money (cross-talk) go to the public hospital first and if that fails, go to a private clinic (not clear) they have no testing methods. These religious groups who don’t go to the hospital are making diseases increase because they don’t follow the world counselling, they only want their things to be done

**04:** most people don’t go to the hospital because of the coming of the herbs. People do believe that if I use herbs that are sold, I will be okay

**Facilitator:** herbs, mmh

**04:** they drink herbs but they don’t ask for the dose. They can just be drinking, looking like they are improving yet they are causing another problem to their health, so most people believe that ‘’I was told that if I mix this and that, I will produce medication. ‘’ but they are destructive

**Facilitator:** On the issue of distance, I would like to know, how far is Bangwe clinic.

**00:** Bangwe is big, and the facility has not only been used by the people of Bangwe. For example, the facility is here, there are railways, and they are far from the clinic. People from Ntopwa are closer to the clinic. Namatapa is very close to the clinic. There are people who are living in K, there are some numbers I don’t know

**Facilitator:** but they are in town

**00:** exactly, they depend on this facility

**0:** there are others from Mondiwa

**00:** (not clear) They come here, people from Chiladzulu, St. Patricks come here as well

**Facilitator:** Those who come from far areas, how much money do they use for transport?

**00:** they can use K2000 for them to get to the facility

**Facilitator:** is that one way?

**00:** For those who come from K in Mpingwe, it’s K2000 one-way

**0:** those who come from Ntenje in Chigumula, if you are two people both adults, you can pay K3000 to be dropped off at the clinic one way

**Facilitator:** K3000

0: that’s fair and he has done you a favour because in some of the areas, minibuses don’t go, only motorbikes do, so you hire it as an individual, unlike minibus, you contribute K500 each

**Facilitator:** do you know the drugs called antibiotics?

**04:** yes

**Facilitator:** can you mention the types of antibiotics that you know?

**04:** drugs like amoxicillin (cross-talk) Bactrim

**0:** panado

**Facilitator:** is panado antibiotic?

**0:** aspirin

**Facilitator:** mmh

**0:** I have replaced Panado with aspirin

**All:** laughing

**Facilitator:** so, amoxicillin, panado, aspirin, what else?

**All:** Silent

**Facilitator:** I am asking this because we may use antibiotics as a treatment when a child gets sick

**04:** amoxicillin and panado can be available

**Facilitator:** apart from the health facility, where else do you access the antibiotics?

**04:** from the pharmacies

**02:** we are not the experts, we just guess. ‘’the child has high body temperature, let me buy Panado.’’ So, you just go straight to the shop. ‘’aah I will buy Cafenol’’, or those capsule drugs called…

**Facilitator:** green-green

**02:** not green-green, they are available in shops

**Facilitator:** are they capsules as well?

**02:** yes

**04:** parapain and brufen

**02:** parapain and Brufen we trust it too much

**Facilitator:** buffen?

**02:** yes, it’s just within a short period of time

**00:** there is panado extra

**04:** Panado and brufen are taken together, they are all pain killers but someone comes and says, ‘’I want brufen and panado’’ Someone knows he/she is sick, it has happened to me, someone was coming to our shop, ‘’I want brufen and panado’’ I was like, ‘’aah, these are pain killers, why does she want to mix them?’’

**Facilitator:** I want you to refer to children, the antibiotics that buy when the children get sick. Do we buy green-green for them?

**All:** (cross-talk)

**00:** not green-green, it is bitter

**0:** you open and then you reduce the amount

**Facilitator:** you open and reduce

**0:** yes, I have never done it before, I just see people doing it

**00:** I remember long ago, parents were peeling the outer off (not clear)

**Facilitator:** (laughing) so that it shouldn’t be known

**00:** After peeling, they put it in a teaspoon with water, and then we stir with another spoon. We then give administer to a child, we can administer it 4 or 5 times

**Facilitator:** Okay, let’s proceed and continue with the issue of vaccines. What do people say about the under-5 vaccines?

**04:** there are beliefs that we talked about, some people don’t allow their children to receive the vaccine, and they don’t believe in vaccination. We have seen healthcare workers being sent back. In the recent polio vaccine, people living in fenced houses didn’t allow their children to receive the vaccine, so healthcare workers were been sent back, they believe their children should not receive the vaccine

**05:** people started distrusting the vaccine during COVID because people believed was COVID man-made, it’s some people’s business, so everyone speaks what they think but the coming of COVID destroys issues of vaccines. People say it’s Chakwera who wants to reduce our population, such things have damaged vaccination issues. There are more vaccines coming and people say, ‘’Aah they failed us during COVID, then they have introduced this.’’. everything now has a vaccine, cholera has a vaccine, now people think they failed us during COVID, so they have come here, they want to reduce us. These things are discouraging. Another thing they say is, ‘’Receive this vaccine to protect you from such and such diseases, ‘’ when they have vaccinated you, you get sick from the same disease as well. I have an example. During cholera, I went to the village and when I came back, upon arrival, I met with a health care worker who was distributing cholera vaccine. As I was getting in the house, I was told, ‘’This is your vaccine’’ It was an oral vaccine. I said that I didn’t take that vaccine. Her relatives came, and she gave that vaccine to her relative’s child. The following morning, that child had severe cholera and we visited a child at the clinic, we were surprised because the child had received the cholera vaccine the day before yesterday. The child got sick for 3 days and such things I was like what would benefit me if I got vaccinated and maybe it could be even this vaccine that has caused this child to get sick from cholera. Things like these make us not believe in the vaccines, and let me tell you, if men were taking children to the under-5 clinics, most children would have not been receiving the vaccines, we would have refused children to receive the vaccines but, women just sacrifice children anyhow. When they say, ‘’Bring your children next week for vaccination!’’ we would not have allowed it because vaccines are still coming and children are vaccinated, ‘’eeh this vaccine, that vaccine.’’ There is that organization for civic education, it has a lot of work but it is failing in its duty. Just like how you have come, you would have been telling us, ‘’There is this coming, the issue is this and that.’’ Things are been introduced before people are sensitized and that’s why people are refusing the vaccine. So, it is discouraging people to receive the vaccine

**06:** COVID issue affected the vaccination, someone could get vaccinated, meaning he/she has no COVID. You could see him/her, after 2 to 3 days, you could hear that person has what, if you investigate it, you hear it was COVID. So, someone had COVID and yet he/she received COVID vaccine. Most people stopped trusting the vaccines

**02:** on this issue of the vaccination, what people believed was that we are overpopulated in this country. The vaccines that are coming, aim at reducing the future generation and in short, these vaccines aim at killing the human organs so that people should not reproduce in future, that’s what we think. Some of these vaccines are not coming in good faith, they want to make children barren so that the population should be reduced

**Facilitator:** vaccines like what?

**02:** in the recent past, there were 5 vaccines, one after another. Oral vaccine, another one here, another one this area, then you wonder, all 5 vaccines on one child, why?

**FP1:** that’s how we get children vaccinated

**0:** there was another vaccine recently

**02:** the strange one (cross-talk)

**0:** that one, children have received 3 doses, so it came again. Children were receiving the vaccine in school and there was chaos in schools as a result of that vaccine, the issue is that he had said that they wanted to make children barren because that vaccine was for girls only, not boys, girls only (cross-talk). They were receiving 3 doses. First dose this month, after months or weeks, another dose up to 3 doses, and children should explain these things to parents after hearing them from school. Of course, it was announced on the radio but, not everyone listens to the radio. Children could tell you that you don’t know anything, that’s why people thought it was Chakwera who wanted to reduce our population and he wants us to become barren, ‘’Don’t receive this vaccine,’’ so, the vaccine is not going on well

**Facilitator:** It’s not everyone who has a radio, what do you think can be the method for everyone to receive the message?

**0:** community sensitization. Through meetings like this one, you should train us so that when we get back home, we should call for the community meeting and tell people or else, after this meeting, we can go back home and tell a friend, in the process, a message will be disseminated. We cannot rely on the radio, the issue is community meetings

**04:** On top of that, the village headmen are important because when they have a message, they disseminate it at the funeral and everyone hears for themselves.

**00:** everyone who has gone to the funeral, if there is a message, for example during a cholera outbreak, messages were disseminated at the funeral and everyone who goes to the funeral could hear the message. If you are a married man and you have gone to work, if your wife heard the message, she was going to tell you

**Facilitator:** Okay. Apart from community meetings, is there another method that you think can be used for people to receive the messages easily?

**00:** I think through schools, people can receive the messages easily but, vaccine messages should not be disseminated to school-going children, it’s difficult. Messages should go to parents first, then parents should be telling their children because most of the time, children don’t trust teachers, they trust their parents because there were rumours in the past and you might be aware of. People were saying that teachers have shut the doors giving way to the bloodsuckers, some people could run away from school because of distrusting their teachers, and maybe a teacher has just closed the door to prevent children from just moving ups and downs when I am in the office. So, most of the time messages here in Ntopwa are disseminated through the funeral because most of the time here in town, people don’t come when you call for a meeting unless you want to register them for something, but still, some go to work. So, messages are disseminated through the funeral, that’s the most effective one

**Facilitator:** what about….

**04:** messages can be disseminated through cell phones because many people have cell phones, and we see messages saying, ‘’polio vaccine team will come on a such and such a day.’’ Some of us didn’t know the messages through the radio, we knew it through the phone, so we prepared the children to receive the vaccine

**Facilitator:** what about the use of churches and mosques, can they be used as one way of disseminating the messages?

**0:** Yeah, it is the most effective one, same as funeral and church methods, no matter what, people go when a day comes

**00:** issues of polio vaccine, I have been hearing the announcement at our church

**0:** The vaccine team came to the church before finishing the service to offer the vaccine

**Facilitator:** sometimes we do have a political rally

0: those are difficult ones, for example, if someone from the ruling party comes and start talking about the issues of vaccine, people will never agree with him/her. What people want now from the government is money for maize, people will then agree with him/her, but if they come and say, ‘’We have come to tell you about the issues of vaccines,’’ that meeting will end amidst the way because people don’t want it. Maybe if it is the opposition party, and not the ruling party to come at that ground and say, ‘’we would like to tell you about this and that, ‘’ aah it will not work because….

**07:** (if this meeting was announced in the community, ‘’all under-5 children should come!’’ People would not have come because they believed that the vaccines were bad, they wanted to reduce the population

**Facilitator:** Among the groups that I have mentioned like churches, politicians and the village headmen, who can be trusted with this issue of vaccine message dissemination?

**0:** Churches

**All:** (cross-talk)

**Facilitator:** why churches?

**0:** churches are spiritual, even if the pastors are deceiving us, we will believe them. Sometimes village headmen are corrupted, and they can be given a bribe, but most of the time we trust the village headman more than the politician. Additionally, the village headman tells people, ‘’If you don’t get your child vaccinated, and if that child dies, I will not entertain you.’’ (sindidzakutulutsirani makasu) so people go because of fear, if the village headman has spoken, if I don’t do it, he/she will refuse me when things are not okay, (1:3:18 not clear). For every passport or ID to be done, the village headman should be available, so people are afraid of the village headmen and they are known by many people

**02:** we trust the village headman for we know that if there is a plot to kill people, the village headman cannot allow his/her people to die. If people die, is he/she going to be a village headman?’’ who will he/she be governed? None, so we believe that the village headman cannot betray us

**Facilitator:** Alright. Let us finish up with the issue of the Rota vaccine, I think issues are mixing, aren’t they?

**All:** mmh (yes)

**Facilitator:** I would like to hear your views on how people received the Rotavirus vaccine here in Bangwe. We have said the Rota vaccine was introduced in 2012. We have heard about other vaccines

**05:** my friends came earlier than me, but, I just saw a poster at the clinic when I went on that day, but I don’t know if it’s important. If you had explained to us, then ask us later. Maybe my friends, do you know it?

**04:** I have never heard about it

**02:** I came earlier here, but I had never heard about this vaccine and somehow I was asking, ‘’What does this Rota mean?’’

**Facilitator:** laughing

**07:** I have many children and I know this vaccine, but most women refuse it because they start the Rota vaccine when they are pregnant. So blood sample is taken for testing, so women say that they take a lot of blood. After that, when a child is born, he/she is taken a blood sample too. So, they say, this child is born with someone’s blood and is taken the blood sample, do they want him/her to be alive or not? Children whose parents receive this vaccine die below 4 years, so many people hate this vaccine, saying it is not good

**Facilitator:** May you explain, how do you know that children below 4 years die after receiving this vaccine?

**07:** I have said that I have many children..

**Facilitator:** (how many children?

**07:** 4 children, the fifth one died

**Facilitator:** sorry

**07:** she goes to the under-5 clinics every now and then, so women do discuss, ‘’This vaccine is good, it has helped my child to do an omphalotomy (1:6:23 minutes), so they discuss like that. They are given the same date for the next visit, so they discuss again, ‘’My child is sick’’ ‘’Mine is done’’

**0:** I think that’s what we discussed earlier,

**Facilitator:** mmh

**0:** about the collection of blood samples, is this what you are saying?

**07:** yes

**0:** that’s what I said earlier

**Facilitator:** did you have something to say?

**FP1:** no, I don’t know this vaccine

**Facilitator:** Rota vaccine

**FP1:** yes, I don’t know it

**Facilitator:** you never even heard about it

**FP1:** Okay, I heard that children receive different vaccines at the under-5 clinics, so I was thinking, ‘’Did my child receive this vaccine or not?’’ so I don’t know

**02:** Since we are here to know, when you know, you have a real picture. This vaccine, I don’t know if my children received this previously when they went to the under-clinics with their mother. Does this vaccine have no other name? Maybe we know it by another name

**Facilitator:** If it has another name, then I don’t know. Maybe I have missed that name too, but the name that is used is the Rota vaccine or Rotavirus vaccine, so I should go and find out the other name what we are discussing here is research and we know that maybe many people don’t know about this, that’s another message, most people don’t know that there is Rota vaccine

**0:** I will ask when I go home what this means

**07:** I repeat the question, what do you know about this vaccine?

**Facilitator:** I will explain, for now, we are discussing, at the end, we will have time for that because if I explain amidst the discussion, you will feel like your views are not important because I have already explained, let’s finish first.

**07:** Alright

**Facilitator:** what could facilitate the Rotavirus vaccine awareness in the community?

**02:** I am one of those who don’t know this vaccine, but I come to tell people the advantages because everything has advantages and disadvantages. Community members should be told about the advantages and disadvantages of this vaccine, but I do believe that when you are selling the plot, you don’t review how you got that plot, and then people won’t buy it. So when coming, you will come with tangible messages that everyone should be aware of, though there is something behind the vaccine which you know that although this vaccine has this advantage, there is this disadvantage too

**Facilitator:** Okay, I got you. Before we discuss the vaccine advantages and disadvantages, I want us to get back to the point that someone talked, saying that before COVID, people were receiving vaccines

**All:** yes

**Facilitator:** I would like to know, what motivated people to go and receive the vaccine in the past.

**07:** I believe that what was motivating them was that health care workers were telling people that everyone who has never received the polio vaccine when getting the heath passport book, if he/she has taken another health passport book and go to receive the vaccine, so parents were keeping the health passport book for a first child so that if t is polio vaccine, this child should receive it. The coming in of these things, pregnant mothers don’t start the ante-natal clinic for up to 7 months to avoid being vaccinated again. So, they can be encouraged by telling them to follow such messages and healthcare workers should tell people the advantages of the vaccine before they get vaccinated, but when they tell them that we are going to vaccinate another one because they have lost their health passport book, they say, ‘’not me, I better terminate the pregnancy because they want to kill me, so I better die myself.’’ These things make hospitals have too much work because people are refusing the vaccine. The vaccines have advantages in the human body but, we experience adverse events because of our blood culture, but some have advantages when they get vaccinated. So, the advantages he experienced, to some it’s a problem, so we think that I should not face a similar problem. Therefore, people should be encouraged but then I don’t know the approach because people lost trust

**0:** May you repeat the question because I am lost

**Facilitator:** you have said that the vaccines were interrupted because of the coming in of COVID. Then I asked, what was motivating people to receive the vaccines in the past?

**0:** aah okay, I am repeating. People had trust in the vaccines before COVID. I remember when COVID had just started, politicians who are in government now said there was no COVID. When Peter was president, these ones were saying there was no COVID. These ones were in government, if there were COVID benefits, they were benefiting and they knew. When these ones started governing, they saw that there were benefits if they agreed, and they started agreeing that there was COVID. So people started thinking that this politician wanted to hurt us and when the time came for people to start receiving the vaccine, with COVID vaccine, it was compulsory and nearly manhandled us for a jab. For us to reach this day without receiving the vaccine, it was our courage but there were many ways. You could not get in the office if you were not vaccinated, so there were many ways to ensure that we had received the vaccine and we were wondering, why they were forcing us. Such things discouraged people. The biggest number of those who got vaccinated are those living in cities because they have time to go to different offices where such things are demanded. Most of the people living in typical rural areas, I don’t think there is anyone who received the COVID vaccine, and if you go there, out of 200 people, maybe you will find 5 of them got vaccinated, in cities, you can 50 to 100 people who received COVID vaccine because they have time to go to the different offices where it was a compulsory

For people to be aware of the Rota vaccine fast, when a pregnant mother is starting the ante-natal clinic or during the ante-natal clinic, there is a time for counselling, there should be a healthcare worker offering a health talk special for the Rota vaccine, telling people that this vaccine helps in a such and such a way, then people will become aware. Secondly, tell all people at the under-5 clinics, I think it can be better. Another area can be the general patients' waiting area, they have time for the announcements, ‘’This and that!’’ I think in these three areas, at the ante-natal, general patients waiting area and the under-5 clinics, if these three areas are reached out to, churches and community meetings may not be effective

**Facilitator:** mmh

**0:** everyone going to the facility will get the message about the Rotavirus vaccine, until now, women like these would have known it, and it cannot take a year without us going to the hospital, we would have heard of it this vaccine

**Facilitator:** If I may use a language that he used, like benefits and disadvantages, what benefits did people see with the vaccines before COVID-19?

**04:** the benefit was that when they received the vaccines, they had no health problems, than this vaccine. When someone receives the COVID-19 vaccine, he/she gets sick with COVID-19, that’s the difference. During that time, when a child receives polio vaccine, he/she was not diagnosed with polio

**Facilitator:** so, people had trust

**04:** yes

**07:** just to comment on what she has said. Another example is malaria, when you give a patient Fansidar, the patient could stay for 6 months without getting sick from malaria. That meant Fansidar was a malaria vaccine on its own. People do believe that if they give me 3 tablets of Fansidar to take instantly, I will not get sick. When a child receives the polio vaccine, that child will grow up, so people believed in the vaccines because of such things, when the COVID vaccine came, eeh it was fire

**05:** there are different businesses today, people are establishing organisations maybe to manufacture vaccines. Telling people that we are giving this vaccine, so they receive money because they are distributing the vaccine. We don’t trust each other because there is too much gambling

**Facilitator:** some people are benefiting

**05:** Exactly

**Facilitator:** we are going towards the end, what’s your final comments? Is there any other benefit apart from that? Or maybe regarding the disadvantages of the vaccines

**05:** another problem that we experience is that you can take a child for vaccination and those who vaccinate are not serious, they haven’t been injected at the right place, there is a bulge up to one year, so you don’t blame health workers, rather you blame a vaccine itself yet it was a health care worker who did not vaccinate well

**0:** additionally, certain children around Luchenza received the vaccine, and we don’t know if the health worker who was giving the vaccine was a learner but after a day, a child’s buttocks started swelling and after a week, there a growth with pus when the child was taken to the hospital, ‘’this child not vaccinated well.’’ As I am speaking now, bad things were sucked out of the wound and the child’s buttocks changed shape until now. When people saw that, they did not think of a healthcare worker, but rather the vaccine. With the Rota vaccine, we know it has been there since 2012, yet people could not identify it, it is your duty to sensitize to people about this vaccine. This vaccine should be with the experts, and not just pick anyone out there who is tired, ‘’go and vaccinate’’

**Facilitator:** laughing

**00:** to add on that, most men should take part because when I have a child, I do challenge myself that I will be responsible for feeding the child, then someone telling me, ‘’Practice the family planning.’’ Yet it will be my responsibility to feed the child. Men tell their wives, ‘’Is he/she going to feed a child?’’ I am not the only one there is a group of men, so the woman practices family planning without the knowledge of her husband, fearing that when he knows, he will shout at her. If the woman does not know anything, he shouts indeed, ‘’How come you obtain the family planning method! Please go!’’ we are saying that these family planning methods women are using are decreasing our manhood in bed. No man allows his wife to go there, and if there is a man who allows his wife to go there, he doesn’t care even if his child dies, he will be eating his money alone

**Facilitator:** how the vaccine is decreasing manhood?

**00:** manhood just disappears

0: to be honest, this vaccine makes sexual feelings disappear, when you do one, you stay for a week (cross-talk) she can be walking naked but you cannot run after her, the manhood is dead. So the vaccine is hurting us, to be honest.

**00:** that is why, people are afraid of most of the vaccines, thinking that they aim at reducing our population. So, if people are not told of the advantages of this vaccine, people will think it’s the same thing. So men are missing that, we should know the advantages of vaccines

**07:** I heard that the vaccine research experts like yourselves, don’t allow your wives to be injected with the family planning methods, and you have your own methods that you use. So you want to hurt us with these other methods, and I think it’s true. It’s germs that are available in the drinking water, they are not visible, but when you use a microscope, you see that ‘’eeh! There are germs.’’ So, you see that these are the germs but, we cannot see them because we have no that lens

**0:** let’s be honest, I stay in Lilongwe and I will not mention the health facility where he is working. He called me, ‘’Don’t allow your child to receive that newly introduced vaccine.’’ I was like ‘’aah! if one at the pot speaks like this, who am I?’’

**Facilitator:** laughing

**0:** I never got my child vaccinated

**Co-Facilitator:** which vaccine was that?

0: the final one

**Facilitator:** there were many vaccines, there was the cholera vaccine, polio…

**0:** the final one

**Facilitator:** oral vaccine?

**0:** last week one, polio vaccine (cross talk)

**00:** what is surprising is that this lady here received the polio vaccine before the child was born. After a child was born, he/she received a vaccine and now, she is told that the child should receive another one. The child was injected in the buttock, then there was another one, an oral vaccine. So polio vaccine has three doses. Oral one, then there is malaria one, add up to 4

**0:** This one has been introduced because polio disease has come back, I heard that 4 people were diagnosed at that time. So they want this not to become worse like cholera or other diseases. So, the government has risen up so that it should not reach that extent. If this attention was given to the hunger that people are experiencing, we would have no hunger in our homes

**03:** I think drugs have overstayed in the warehouses, they have expired. Since they have stayed for about 50 years, they are injecting people. So they have to take these drugs out of the warehouses and replace them with new ones so that they should be injecting people like before, just like the polio vaccine, I think it cannot come again

**Facilitator:** On the issue of the family planning vaccine, I think ladies' views are necessary too because they are the ones who receive it. Ladies, what do you experience after receiving the vaccine in line with what men are saying regarding the reduction of manhood in bed?

**0:** do you experience what we do or not?

**00:** set us free

**Facilitator:** laughing

**04:** what you may experience may be different from what we may experience because those drugs are in our bodies. Sometimes you may feel backache, yet you were okay before obtaining this

**Facilitator:** after the vaccine

**04:** exactly, women say that after starting receiving the family planning methods, they experience backache

**FP1:** some change their menstruation periods

**04:** some even stop

**Facilitator:** does it happen to some?

**04:** Exactly

**0:** when the women are courageous enough, they tell us that there are some women who continuously do their menstruation period for up to 2 weeks

**03:** even the whole month or 2 months. They even go to the hospital for treatment. Pills family planning method makes women not to do their menstruation periods

**Facilitator:** these family planning experiences, do they affect our vaccine choices?

**07:** they should not affect, but because of our understanding, we think they are similar, so we think every vaccine is like that. So there are many vaccines like polio, ever since, there has been no parent that refuses it. The measles vaccine, people do trust it and they believe that the vaccine really functions

**Facilitator:** let me finish with this question, it’s the final one. This one said that one child receives 5 vaccines, for what, and this one answered, ’’That’s how we have been receiving.’’ Although it was low tone, I was listening

**04:** (1:28:12 not clear) 14 days, it was her child who started

**Facilitator:** mmh

**04:** after 6 weeks, the child receives 2 vaccines. The following month the child received another 2 vaccines and the next month, the child received 2 vaccines, then the child finished the vaccines (cross-talk)

**07:** that’s the normal vaccine she is referring to. I was taking my child to the vaccination site after 2 to 3 months until the child finishes the vaccine, but we are referring to these vaccines that have just popped up from nowhere, (cross-talk) I am not pleased with these vaccines because (1:28:58 not clear)

**Facilitator:** Okay, what are the normal vaccines?

**07:** the vaccines that you have seen recently (cross-talk)

**04:** the old vaccines that children receive, when the child is born, we take him/her to the under-5 clinic and we know when a child is 1 year, at 15 months old the child is supposed to receive the measles vaccine, so we know it’s normal, that’s why people say previously, people had trust in the vaccines because they knew that when the child receives the vaccine, he/she never gets sick every now and then, it was because of the vaccine he/she was receiving, that’s where the issues of trust came about

**Facilitator:** mmh

**0:** the child is injected on both sides, and there is another one

**04:** mmh

**0:** not the oral one then this one

**All:** laughing

**Facilitator:** Okay, Alright. This is what I had for this discussion. I have learnt a lot, and I would wish this discussion could continue but, every song must come to an end even if it is good...

**04:** (you haven’t explained to us regarding the Rotavirus

**Facilitator:** I will explain. Let me give you a chance because when discussing, someone may have an issue that he/she expects us to discuss, and yet it is not mentioned. This is the time, if there is something that we have skipped, you can raise it before we start answering the questions that you asked.

**00:** I would like to ask and everyone will take it the way he/she understands it. We are talking about diarrhoea and there are many types of diarrhoea, especially children. They might eat something that is not suitable for his/her health, so the child has diarrhoea. Then we are talking hygiene issues, you have just removed a pamper and thrown it away, then you come back without washing your hands, and two to three days you see a child having diarrhoea or else, you have eaten yesterday’s food, ‘’you are crying, just have this food.!’’ And within a day, he has diarrhoea. So we don’t know which diarrhoea can a child receive this vaccine.

**Facilitator:** before I answer you, let me ask you, when you say a child has diarrhoea, what do you mean?

**0:** diarrhoea is just diarrhoea, there is bloody diarrhoea that a child can get sick with. There is cholera, a child can get sick with, and then there is another normal diarrhoea the child can get sick with. Bloody is abnormal, cholera is abnormal, but there is another one in which you may say the child has just eaten something like flitters, and the digestion has not happened well, so the child is releasing the food, so within two or three hours, the child is okay

**07:** I think there are different diarrhoea regardless of their names because with my age, I know that I have overeaten, I am not sick but what I have eaten has affected me

**Facilitator:** mmh

**07:** then there is sometimes when I only eat porridge for two days, maybe sometimes I don’t eat, so have diarrhoea, I go to the hospital. I think that’s when this vaccine is important. You don’t see anything wrong but you have diarrhoea, that one needs a vaccine, not when you have eaten and get constipated, aah you just say I will drink ashes and will get okay

**Facilitator:** ashes

**07:** based on the beliefs

**All:** laughing

**Facilitator:** people do drink ashes

**07:** yes

**00:** we grind the charcoals and mix them with water, even poison, if someone has eaten poison, just give him/her that mixture, then a second treatment may follow

**0:** (1:33:46 not clear) that’s a very powerful medication

**00:** this very flue that we are drinking the warm ashes, you can sleep without problem

**Facilitator:** thank you very much, we have discussed this for a long time. Let me respond to that question, what was it?

**00:** it was about the Rotavirus vaccine (cross-talk)

**Facilitator:** Okay fine. I will somehow be repeating what you have said, there are different diarrhoeas like bloody cholera and all that…

**00:** including the diarrhoea that starts when a child is teething

**Facilitator:** Exactly, there are different types of diarrhoea. What happens is that every disease has the sources, and the sources may be bacteria, viruses or protozoa. Otherwise, they are microorganisms. Rota is one of the microorganisms that causes diarrhoea, do you get me?

**All:** mmh (yes)

**Facilitator:** of all microorganisms that cause diarrhoea, there is Rotavirus, just like COVID, it is a virus, okay

**All:** eeh (yes)

**Facilitator:** there are now different viruses that cause COVID, so there are different viruses that cause diarrhoea amongst them, there are very difficult ones, so much so that if someone has diarrhoea, it’s them that has caused the sickness. We are talking of diarrhoea that happens every now and then, maybe from morning, every 30 seconds you have diarrhoea, so we know through that, just like what someone said that you may have diarrhoea because of the food that you have eaten, but, there is a virus that has contacted and contaminated the food because food on its own cannot cause diarrhoea because it is food. When it is causing diarrhoea, it’s because the food has been eaten together with a virus that causes diarrhoea. In short, Rota is a virus that belongs to the viruses that cause diarrhoea and there are different viruses. This vaccine was introduced to deal with Rotavirus because most of the time, it is this virus that causes diarrhoea

**00:** Okay, when a person is supposed to receive this vaccine?

**Facilitator:** this vaccine is normally given to the under-5 children, it is given at the under-5 clinics. Here in Malawi, this vaccine was been offered as a trial, so those who received this vaccine at first, were receiving it as an experiment, here in Blantyre, the trial was done in several hospitals and it showed that the vaccine is working. When they discovered that the vaccine was working, that’s when the government took the responsibility to start purchasing the vaccine and delivering it to hospitals for people to start receiving it. So we moved from the trial stage to roll it out, so every woman who has a child who is under 5 is asked to receive the vaccine. As I said, the aim of the vaccines is to do what?

**All:** to protect

**Facilitator:** so diarrhoea normally affects children apart from cholera affects everyone, that’s why we are giving them this vaccine to protect them

**00:** in short this vaccine is on a program that when a child reaches this age, should receive the vaccine

**Facilitator:** yes

**00:** okay fine

**Facilitator:** it’s there on the EPI program that you know

**04:** which means children like ours already received the vaccine

**Facilitator:** I should not say they already received it, then it means they received it without your knowledge. It is possible that they explained but you have forgotten or else, the child hasn’t received it that can be possible. So, as health care workers who are closer, go and check with your child’s health passport book, you may know that my child received this vaccine, because one day, you may say that my child never received the vaccine yet he already received it

**All:** cross-talk

**04:** I think my child received it but, they did not explain

**Facilitator:** it is good for parents to ask whenever they are told that their child is going to receive the vaccine so that they should which vaccines their child is receiving

**0:** I think you are the owner of this vaccine, aren’t you?

**Facilitator:** no, we are not the owners, we are health researchers, so we are doing research on the Rotavirus vaccine. So it is an organisation, it is possible to have another organisation in Lilongwe conducting the same research, but we are here in Blantyre. There can be another organisation in the Northern region doing the same research, but what I am telling you is that our organization, Malawi-Liverpool Wellcome Trust in corroboration with Kamuzu University of Health Sciences are doing this research. There has been diarrhoea research conducted at Bangwe Health Centre all along, that research was conducted before the Rotavirus vaccine research, so they wanted just to see the diarrhoea prevalence here in Bangwe, so the research is well established, and this Rota comes later

**0:** I asked this because the vaccine owners should be explaining to people so that people should be aware of, as some ladies said, maybe others received the vaccine without their knowledge, that’s why they are surprised with the vaccine, and people should be told that as we are offering this vaccine to a 6-month-old child, there is another one, Rotavirus vaccine, eeh

**Facilitator:** mmh another response to that question that you asked if we are the owner of the vaccine, when the research is happening, it is for all of us. There is a committee that oversees and that committee asks, ‘’Who is the first benefit of this research? Or who is going to benefit?’’ So they want every research to benefit community members first, that is why there is a committee to ensure that the research should benefit the intended people. The Ministry of Health ensures that the research benefits people and if the research shows it will benefit, they take over because it is the responsibility of the Ministry of health to ensure every benefit. So when the research is conducted, they have a small sample population to see if there is a benefit. They cannot take it to everyone during the trial period

**0:** Okay

**Facilitator:** Now, the trial period went, now it’s for everyone, and it is with the government now

0: I want to ask before you finish, how many doses a newborn child is supposed to receive?

**Facilitator:** let me ask how many doses, then I will call you

0: Okay

**Facilitator:** I should ask the nurses who are administering the vaccine, I don’t know as well, so I will make a phone call when I have an answer and if the vaccine is been offered, I will let you know where you can access it

**04:** 2012 is a long time ago (cross-talk)

**Facilitator:** maybe it is a name that we don’t know. It is possible that they don’t use this terminology at the hospital when giving the vaccine, that’s where I was not sure of, so healthcare workers are experts, they know the best approach when describing illnesses because they know that when we use this terminology to a community member, he/she will understand it better, so it is possible they are explaining it, but they are doing it the way I am talking

**0:** that’s clear

**Facilitator:** isn’t it

0: yes

00: ‘’aah nyimbo imodzi sachezera gule.’’ (repeating things can be boring)

**Facilitator:** thank you very much

**End of interview**
