# Supplementary material for: Exploration of the social determinants of diarrhoea, rotavirus vaccine uptake, and vaccine ‘fatigue’ in Ethiopia, Kenya, and Malawi
Source: PLoS One. 2025 Sep 9;20(9):e0319691. doi: 10.1371/journal.pone.0319691 (PMC12419581; doi:10.1371/journal.pone.0319691)
Supplement: Attachment — Submitted filename: Review comments.docx [file pone.0319691.s002.docx]

Title: “Exploration of the Social Determinants of Diarrhoea, Rotavirus Vaccine Uptake, and Vaccine ‘Fatigue’ in Ethiopia, Kenya, and Malawi”

Generally, well well-written manuscript that highlights the social determinants that influence vaccine uptake and diarrhoeal illness. Please see my comments below.

Introduction

1. Line 55: I agree with the authors that vaccine hesitancy could contribute to low coverage of rotavirus vaccine, but did the authors also consider gaps in introducing or scaling up rotavirus vaccines?
2. Line 56: Please clarify the specific SAGE working group you refer to? And cite the appropriate source document.
3. Lines 68-69: “For instance, a study in Kenya found that incorporating hygiene education into maternal and child health interventions **increased rotavirus vaccination coverage”-** What was this increase? Increased by what percentage and over what period?

Methods

4. Line 120: On recruitment, is there a definition for hospital catchment area? What radius? Proximity or otherwise can affect care-seeking behaviour, health education, as well as influence positive health-promotive behaviours.

Results

5. For context, can you clarify if the communities involved in this study have had previous experience of cholera or other diarrhoeal illness outbreaks?. This can influence their experience and knowledge as well knowledge of preventive vaccination.
6. Line 326: “…using antibiotics such as Doxycycline, Amoxicillin, and **Flagyl**”. Since this statement is not a direct quote, I will suggest you replace Flagyl with Metronidazole.
